# The fine-scale associations between socioeconomic status, density, functionality, and spread of COVID-19 within a high-density city

**DOI:** 10.1186/s12879-022-07274-w

**Published:** 2022-03-21

**Authors:** Anshu Zhang, Wenzhong Shi, Chengzhuo Tong, Xiaosheng Zhu, Yijia Liu, Zhewei Liu, Yepeng Yao, Zhicheng Shi

**Affiliations:** 1 Otto Poon Charitable Foundation Smart Cities Research Institute, The Hong Kong Polytechnic University, Hung Hom, Hong Kong; 2 Department of Land Surveying and Geo-Informatics, The Hong Kong Polytechnic University, Hung Hom, Hong Kong; 3 Research Institute for Smart Cities, School of Architecture and Urban Planning, Shenzhen University, Shenzhen, China

**Keywords:** Spatial association rule mining, Geographic knowledge discovery, COVID-19

## Abstract

**Background:**

Motivated by the need for precise epidemic control and epidemic-resilient urban design, this study aims to reveal the joint and interactive associations between urban socioeconomic, density, connectivity, and functionality characteristics and the COVID-19 spread within a high-density city. Many studies have been made on the associations between urban characteristics and the COVID-19 spread, but there is a scarcity of such studies in the intra-city scale and as regards complex joint and interactive associations by using advanced machine learning approaches.

**Methods:**

Differential-evolution-based association rule mining was used to investigate the joint and interactive associations between the urban characteristics and the spatiotemporal distribution of COVID-19 confirmed cases, at the neighborhood scale in Hong Kong. The associations were comparatively studied for the distribution of the cases in four waves of COVID-19 transmission: before Jun 2020 (wave 1 and 2), Jul–Oct 2020 (wave 3), and Nov 2020–Feb 2021 (wave 4), and for local and imported confirmed cases.

**Results:**

The first two waves of COVID-19 were found mainly characterized by higher-socioeconomic-status (SES) imported cases. The third-wave outbreak concentrated in densely populated and usually lower-SES neighborhoods, showing a high risk of within-neighborhood virus transmissions jointly contributed by high density and unfavorable SES. Starting with a super-spread which considerably involved high-SES population, the fourth-wave outbreak showed a stronger link to cross-neighborhood transmissions driven by urban functionality. Then the outbreak diffused to lower-SES neighborhoods and interactively aggravated the within-neighborhood pandemic transmissions. Association was also found between a higher SES and a slightly longer waiting period (i.e., the period from symptom onset to diagnosis of symptomatic cases), which further indicated the potential contribution of higher-SES population to the pandemic transmission.

**Conclusions:**

The results of this study may provide references to developing precise anti-pandemic measures for specific neighborhoods and virus transmission routes. The study also highlights the essentiality of reliving co-locating overcrowdedness and unfavorable SES for developing epidemic-resilient compact cities, and the higher obligation of higher-SES population to conform anti-pandemic policies.

**Supplementary Information:**

The online version contains supplementary material available at 10.1186/s12879-022-07274-w.

## Background

Although the COVID-19 pandemic seems to become gradually under control with available vaccines and public health measures, continuous control of COVID-19 and prevention of recurrent outbreaks appears a must. Herd immunity through vaccination can take a long time or even be unachievable [1], current vaccines have reduced effectiveness against certain new SARS-CoV-2 variants [2], including that against both symptomatic and asymptomatic infections [3]. Precise control measures, tailored to specific needs of different neighborhoods and population groups within a city, are pressingly needed if the great socioeconomic and medical costs concerning the long-term epidemic control are to be reduced. Also, urban design needs to be informed about how to make cities more resilient to the prospective future epidemics.

The precise epidemic control and epidemic-resilient urban design depend highly on the understanding of the complex associations between many urban characteristics and the spread of COVID-19. To understand these associations is the key to identify high-risk neighborhoods and population groups, to pinpoint COVID-19 transmission routes causing the high risk, and finally to determine epidemic control measures and urban designs pointed to these risk factors and transmission routes. Here, intra-city COVID-19 transmission routes may be roughly divided into (a) *within-neighborhood* *transmissions*, including those between family members and between residents in the same neighborhood, often due to failing to keep social distance or defected built environment (e.g., ventilation); and (b) *cross-neighborhood* *transmissions*, typically caused by cross-neighborhood activities and interactions between residents in different neighborhoods.

Studies on the associations between urban characteristics and the COVID-19 spread have resulted in many valuable findings [4–13]. However, the following issues are pending to be settled:

- Limited by the granularity of data, most studies on these associations used cities/counties/towns as the spatial units. Only a couple of studies pioneered the intra-city associations [10, 11]. At a city or coarser scale, it is hard to observe the associations between intra-city COVID-19 spread and urban characteristics. As a result, experts consider some socioeconomic, density, and functionality characteristics as jointly and interactively contribute to COVID-19 spread [14], but there is a lack of empirical evidence for this argument at an intra-city scale.
- There are insufficient comparative studies on different waves of COVID-19 outbreaks in specific countries or regions. Also, the associations for the cases imported from other countries/regions and for locally infected cases are not sufficiently compared.
- By mainly using regressive models and cross-sectional analysis, the studies normally evaluated the associations between individual urban characteristics and the confirmed case distribution, or the combined association of all characteristics on the confirmed case distribution. There were few studies on more complex combined associations, for example, a characteristic *A* and the COVID-19 spread can be positively associated when another characteristic *B* has low values, while negatively associated when *B* has high values. These combined associations and the comparative analysis in (b) are very important for inferring the joint and interactive contributions of urban characteristics to each COVID-19 transmission route.

Motivated by these issues, this study investigates the joint and interactive intra-city associations between urban socioeconomic, density, connectivity, and functionality characteristics and the COVID-19 spread, through both within-neighborhood and cross-neighborhood transmission routes. The study took place in Hong Kong, China, a metropolis with the world’s most densely populated neighborhoods. A modified version of the association rule mining (ARM) algorithm DESigFAR [15] was used to investigate the associations between the urban characteristics and COVID-19 confirmed case rate as well as the waiting period (i.e., the time duration between symptom onset and diagnosis). Based on differential evolution (DE), DESigFAR can optimize the resultant rules in terms of the strength of associations and capture combined associations between any subsets of variables. The associations for the first four waves of COVID-19 in Hong Kong and for local and imported cases were comparatively studied.

The results of this study can be used to anticipate the intra-city spread pattern from early increases of the cases, thereby taking pointed countermeasures to prevent recurrent outbreaks. The results can also provide references to the development of precise intra-city anti-pandemic measures and the improvement of urban design corresponding to specific pandemic transmission routes. These results would be particularly useful for high-density cities, which are usually prone to COVID-19 spread and play key roles in the pandemic control, due to their high density, extensive traffic networks, and complex uses of urban space. The ARM method described in this study can also serve to investigate the intra-city epidemic transmissions in other cities.

## Methods

### Data and variables

The study investigated two sets of response variables: the rate of the COVID-19 confirmed cases (in ‰ of the total population), and the median/average waiting period (in number of days) from symptom onset to diagnosis of the symptomatic COVID-19 local cases, at the Tertiary Planning Unit (TPU) level in Hong Kong as of Feb 18th, 2021. The values of both response variables were computed from the government’s open confirmed cases data [16]. In the data, each case had available reporting date; the location the case stayed prior to diagnosis, mostly the residence address; and the type of the case, inluding local case, epidemiologically linked with local case, imported case, or epidemiologically linked with imported case. Most cases were symptomatic and had available symptom onset dates. In this study, the cases epidemiologically linked with local and imported cases were also classified as local and imported cases. The addresses of the reported locations of the cases were transferred to latitudes and longitudes by using Google Maps Geocoding API.

The COVID-19 spread in Hong Kong was divided into four waves: wave 1 and 2 (before Jun 2020), wave 3 (Jul–Oct 2020), and wave 4 (Nov 2020–Feb 2021). In wave 1, there were more imported cases than local ones, and most imported cases had travel histories in developed countries badly hit by COVID-19, for example, the UK, the US, and France [17]. Wave 3 started from the Kwai Tsing Container Terminal Cluster with 77 confirmed cases related to overseas crews [18, 19]. Wave 4 started from the Dancing/Singing Cluster with 734 cases related to visitors to 28 local dancing/singing venues [20]. TPUs with high rates of cases in the Dancing/Singing Cluster had a moderate tendency to have higher income and education level (Additional file 1: Table S1).

To link the confirmed cases to the socioeconomic status (SES) of the residents in the neighborhoods, imported cases that were confirmed on border ports upon entering Hong Kong and cases reported in hotels were excluded in this study. After these exclusions, there were 391 local cases and 442 imported cases in wave 1 and 2, 3305 local cases and 68 imported cases in wave 3, and 4634 local cases and 29 imported cases in wave 4.

**Fig. 1:**
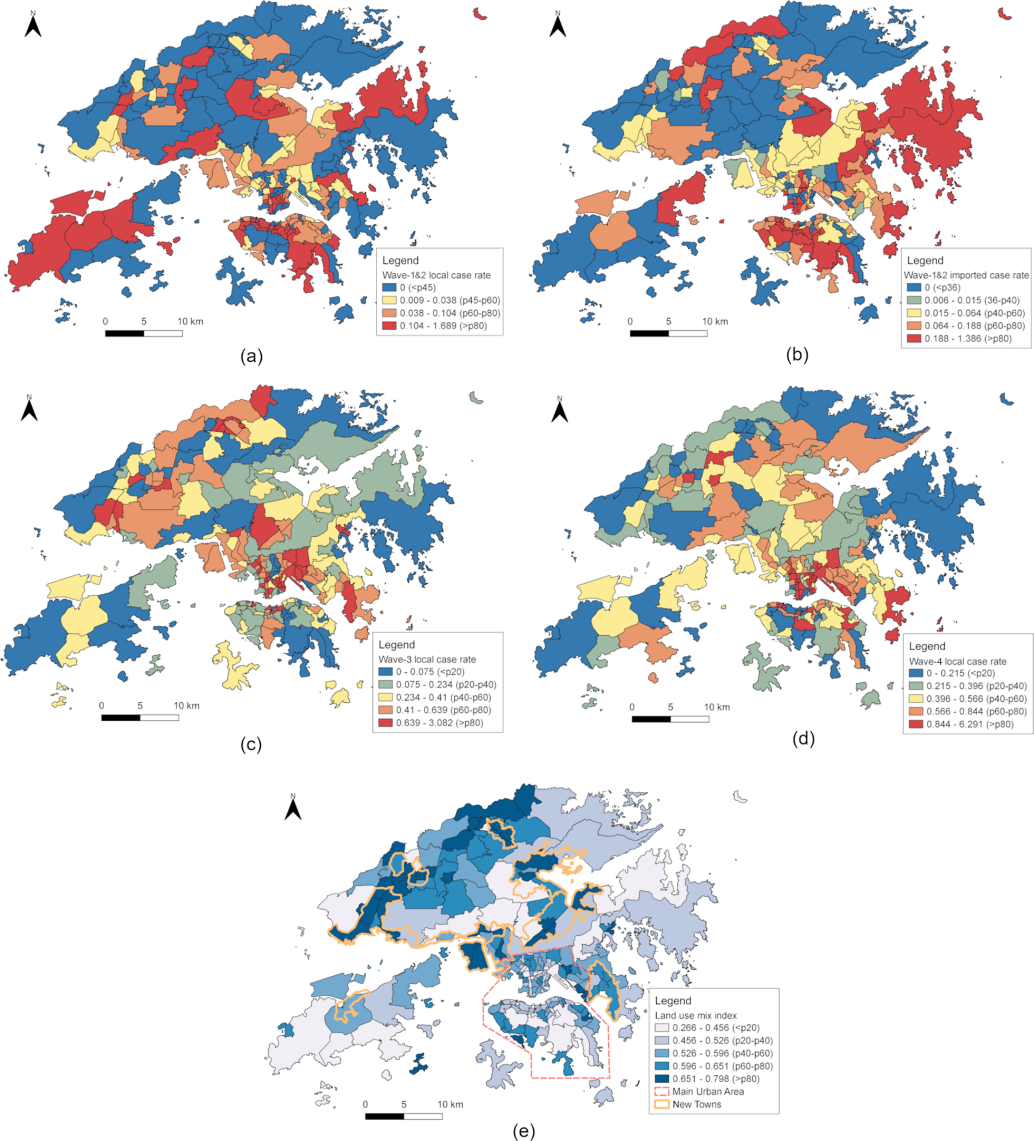
COVID-19 confirmed case rate in Hong Kong as of Feb 18th, 2021. **a** The local confirmed case rate in wave 1&2 of the COVID-19 spread in Hong Kong (by May 2020). **b** The imported confirmed case rate in wave 1&2. **c** The local confirmed case rate in wave 3 (Jul–Oct 2020). **d** The local confirmed case rate in wave 4 (Nov 2020–Feb 2021). **e** The entropy-based land use index value, together with boundaries of the main urban area and New Towns (i.e., satellite towns). The choropleth maps are colored according to quantile classification, and “p” in the legend means the percentile range of the variable values in each class among all TPUs

Then, the confirmed case rate and waiting period were computed for each of the 214 TPU-level areal units covering the entire city defined by Hong Kong 2016 By-Census. The rates for local and imported cases were computed separately (Fig. 1; Table 1). The imported case rates in waves 3 and 4 were not computed, since the cases were too few to be analyzed based the 214 areal units of the study area.

**Table 1:** Explanatory and response variables of the study

Variable name	Description	Min	Max	Median	Source
Explanatory variables					
(a) Demographic and socioeconomic characteristics					
prop_over65	Elderly rate, i.e., proportion of people aged over 65 years	0.02	0.32	0.16	Hong Kong 2016 Population By-census [21]
gender_ratio	No. of males per 100 females, excluding foreign domestic helpers (FDHs)	58.49	329.17	93.14	
prop_preprim_edu	Proportion of population with no/pre-primary education	0.01	0.27	0.10	
prop_higher_edu	Higher-education rate, i.e., proportion of population with post-secondary education	0.06	0.65	0.20	
med_income	Median monthly income from main employment, excluding FDHs (HK$)	7,730	68,000	16,250	
med_area_home	Median floor area of accommodation per person (m^2^)	4.50	56.84	16.90	
ave_household_size	Average domestic household size (person)	1.4	4.7	2.9	
prop_household_3gen	Proportion of three-generation households (with couple, at least one of their parents, and their unmarried children)	0.00	0.83	0.03	
(b) Density and connectivity characteristics					
den_population	Population density (person/Km^2^)	25.81	168,667	18,402	Hong Kong 2016 Population By-census
den_bldg	Building density (Km^2^/Km^2^)	0.00	0.84	0.17	Hong Kong 1:5000 Digital Topographic Map [22]
floor_area_pp	Average floor area per person (m^2^)	20.58	5,595.59	63.01	Hong Kong 1:1000 Digital Topographic Map [22]
den_road	Road density (Km/Km^2^)	0.58	40.72	10.78	Hong Kong 1:1000 Geo-Reference Database [22]
den_public_trans	Density of public transport stations (station/Km^2^)	0.00	271.81	16.43	Hong Kong Transport Department [23]
(c) Land use characteristics: proportion of land use out of TPU area, except for the land use mix index					
prop_private_resid	Private residential	0.00	0.53	0.05	Hong Kong Planning Department [24]
prop_public resid	Public residential	0.00	0.61	0.001	
prop_rural_set	Rural settlement	0.00	0.37	0.004	
prop_business	Commercial/business and office	0.00	0.40	0.004	
prop_industrial	Industrial land	0.00	0.44	0.001	
prop_gov_insti_faci	Government, institutional and community facilities	0.00	0.67	0.04	
prop_open_recreation	Open space and recreation	0.00	0.40	0.03	
prop_transport	Roads and transport facilities	0.00	0.52	0.12	
prop_utilities	Utilities	0.00	0.21	0.01	
prop_vacant _construt	Vacant land/construction in progress	0.00	0.60	0.01	
prop_agricultural	Agricultural land	0.00	0.60	0.004	
prop_woodland	Woodland	0.00	0.67	0.11	
prop_shrubland	Shrubland	0.00	0.45	0.07	
prop_grassland	Grassland	0.00	0.50	0.02	
LU_entropy	Entropy-based land use mix index	0.27	0.80	0.56	
(d) Density and per-capita accessibility of POIs					
POI_den_sports	Density, recreation/sports (POIs/Km^2^)	1.11	12,222	160.27	Hong Kong 1:5000 Digital Topographic Map
POI_den_edu	Density, school/college (POIs/Km^2^)	0.40	5424	96.41	
POI_den_telecom_elec	Density, telecommunication/electric supply (POIs/Km^2^)	0.00	14,343	113.46	
POI_den_transport	Density, transport (POIs/Km^2^)	2.95	11,921	267.41	
POI_den_mall_mkt	Density, commercial center/market (POIs/Km^2^)	0.19	2,942	49.36	
POI_pp_sports	Per-capita accessibility, recreation/sports (POIs/person)	0.62	431.71	16.41	
POI_pp_edu	Per-capita accessibility, school/college (POIs/person)	0.36	281.06	9.32	
POI_pp_telecom_elec	Per-capita accessibility, telecommunication/ electric supply (POIs/person)	0.01	743.25	11.44	
POI_pp_transport	Per-capita accessibility, transport (POIs/person)	0.91	637.60	26.85	
POI_pp_mall_mkt	Per-capita accessibility, commercial center/market (POIs/person)	0.16	152.46	5.12	
(e) Response variables					
rate_local12	Local confirmed case rate, wave 1&2: by May 31^st^, 2020 (‰ out of total population)^a^	0.00	1.69	0.02	Hong Kong Information Services Department [16]
rate_imported12	Imported confirmed case rate, wave 1&2: by May 31^st^, 2020 (‰)	0.00	1.39	0.04	
rate_local3	Local confirmed case rate, wave 3: Jun 1^st^ –Oct 31^st^, 2020 (‰)	0.00	4.72	0.29	
rate_local4	Local confirmed case rate, wave 4: Nov1^st^, 2020 –Feb 18^th^, 2021 (‰)	0.00	6.29	0.49	
ave_waiting_period	Average waiting period of all local cases (days)	0.00	12.5	4.63	
med_waiting_period	Median waiting period of all local cases (days)	0.00	12.6	4.00	

^a^Local/imported cases included cases that were epidemiologically linked with local/imported cases. The rates did not count the cases confirmed in hotels/ports of entry

A total of 38 explanatory variables, including urban socioeconomic, density, connectivity, and functionality characteristics, were also computed for the 214 TPU-level areal units by using governmental open data sources [21–24] (Table 1). For computing the density and connectivity characteristics, areas of TPUs were extracted from the Hong Kong official digital boundaries of TPUs and street blocks [25].

Since TPUs are usually of small areas and specialized functionalities, the daily activities of most people are across TPU boundaries. Thus, the POI explanatory variables (Table 1) for investigating the risk of COVID-19 related to people’s daily activities could not be isolated in a TPU. Instead, following the distance decay law of the trips, the accessibility of POIs of type *O* from a TPU *u*, was computed by:

1 $$accessbility\left( {O,u} \right) = \sum\limits_{i \in O} {p\left( {i,u} \right)} ,$$

2 $$p\left( {i,u} \right) = \left\{ \begin{gathered} 1,\quad i{\text{ is within }}u \hfill \\ \exp \left( { - \beta \cdot 1.3ED\left( {i,u} \right)} \right),\quad i{\text{ is out of }}u \hfill \\ \end{gathered} \right..$$

where *i* represents each POI of type *O*, *β* = 0.3·*S*^−0.17^ = 0.22688 is the empirically most probable value of *β* in a gravity model [26], *S* = 5.172 km^2^ is the average area of TPU-level areal units in Hong Kong. *ED*(*i*, *u*) is the Euclidean distance between *i* and the boundary of *u*, and 1.3*ED*(*i*, *u*) is the approximated road network distance between *i* and *u* [27]. The values of density and per-capita accessibility of POIs (Table 1d) were the value of *accessibility*(*i*, *u*) over the area and over the population of the TPU, respectively.

### Investigating the associations between urban characteristics and COVID-19 incidences

The associations between the explanatory and response variables were investigated by a modified version of the ARM algorithm DESigFAR [15]. ARM aims to discover implicit association rules in the form of “antecedent → consequent” from data. In this study, ARM was used to discover association rules in the form of “interval(s) of explanatory variable(s) → interval of confirmed case rate/waiting period”. For example, a resultant rule “prop_higher_edu > 0.364 (p85) → rate_imported12 > 0.124 (p71)” suggested that TPUs with very high higher-education rates (above the 85th percentile among all TPU-level areal units) tended to have high wave-1 and 2 imported case rates (above the 71st percentile).

The main advantage of the DESigFAR algorithm is that it can discover highly informative rules with strong associations and high interestingness, as a result of optimization based on DE, one of the best-performing evolutionary computing techniques for solving real-world problems [28]. In ARM with numerical data, data discretization is the process to divide the range of each variable into intervals (e.g., the interval prop_higher_edu > 0.364 in the above exemplary rule). Then the association rules will be generated from these intervals. A data discretization scheme includes:

- The number of intervals for each variable, for example, whether the range of higher-education rate should be divided into two or three intervals;
- The numerical data value for each interval, for example, whether the “boundary” of the higher-education rate interval in the above exemplary rule should be at 0.364 or 0.2.

DESigFAR can optimize the data discretization schemes towards those containing strongest associations between the intervals of the variables. Thus, it can discover much stronger rules with summed rule interestingness measure (RIM) values up to 10 times as high as the results of conventional, non-optimized ARM [15]. Also, the resultant rules of DE-based optimization are automatically limited to only those with high RIM values, thus the workload to interpret the rules is greatly reduced, and no attributes need to be precluded to limit the number of rules. Consequently, DESigFAR can address the major challenges in the application of ARM in public health studies, including (a) the discovered rules can be too weak; (b) experts need to conduct tedious manual analysis on the interestingness of the rules; and (c) many data attributes are precluded to limit the number of rules, reducing the chance for obtaining novel findings [29].

The procedure of the modified DESigFAR algorithm in this study are outlined as follows. More algorithmic details can be found from the publication proposing DESigFAR [15].

**Fig. 2:**
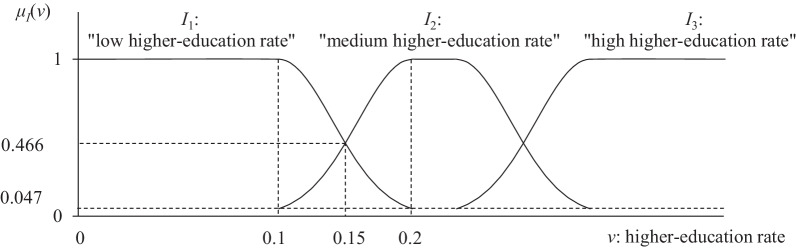
An example of the fuzzy data discretization in the modified DESigFAR algorithm. In each interval (*a*, *c*) of *v* where 0 < *μ*_*I*_(*v*) < 1 (e.g., (0.1, 0.2) between *I*_1_ and *I*_2_), *μ*_*I*_ is a Gaussian curve with a standard deviation equal to (*c*-*a*)/2.473

***Step*** ***1.*** Population initialization. To prepare for the DE, a population *P* with *N*_*P*_ individuals are initialized. Each individual is a vector which encodes a rule template and the corresponding data discretization scheme. The rule template took the form of “any interval(s) of explanatory variable(s) → any interval of the confirmed case rate/waiting period”. For example, the above exemplary rule belonged to the rule template “any interval of higher-education rate → any interval of the confirmed case rate”. DESigFAR adopts a Gaussian-curve-based fuzzy data discretization model (Fig. 2). Under this model, each numerical variable *v* is divided into a number of intervals (e.g., *I*_1_, *I*_2_, and *I*_3_. in Fig. 2). For each interval *I*, a fuzzy membership function *μ*_*I*_(*v*) ∈ [0, 1] is defined to represent the degree to which each value in *v* belongs to *I*. In each interval of *v* where 0 < *μ*_*I*_(*v*) < 1, *μ*_*I*_ is a Gaussian curve.

***Step*** ***2.*** DE. Three operators, namely *mutation*, *crossover*, and *generation* *jumping*, are applied to alter the individuals. Then the *selection* operator is used to select the individual that represents the better data discretization scheme from the original and altered individuals. This step repeats for *G* generations, to let the individuals continuously evolve to containing better data discretization schemes.

***Step*** ***2.1.*** Mutation. Given a mutation scale *F*, *N*_*P*_ mutant vectors *V*_1_, …, *V*_*P*_ are created. Each mutant vector is generated by using three randomly selected individuals, *M*_*a*_, *M*_*b*_, and *M*_*c*_. In the *t*-th generation,

3 $$V_{i}^{t} = M_{a}^{t} + F\left( {M_{b}^{t} - M_{c}^{t} } \right),\quad i = 1 \ldots N_{P} .$$

***Step*** ***2.2.*** Crossover. Given a crossover rate *Cr* ∈ [0, 1], each individual is recombined with a mutant vector obtained from the mutation operation into a trial vector *U*:

4 $$u_{j,i}^{t} = \left\{ {\begin{array}{*{20}c} {v_{j,i}^{t} \quad {\text{if }}rand_{i} [0, \, 1] \le Cr{\text{ or }}j = j_{rand} } \\ {m_{j,i}^{t} \quad {\text{otherwise, }}} \\ \end{array} } \right.$$

where $m_{j,i}^{t} ,u_{j,i}^{t} {\text{ and }}v_{j,i}^{t}$ are the sub-vectors that contain the encoding for the j-th variable in $M_{i}^{t} ,U_{i}^{t} {\text{ and }}V_{i}^{t}$; randi[0,1] is a random number selected from [0, 1]; and jrand is a random index to ensure that the trial vector includes at least one variable from the mutant vector.

***Step*** ***2.3.*** Selection. In each pair of individual and trial vector obtained from the crossover operation, the vector having a higher fitness value will survive to the (*t* + 1)-th generation:

5 $$M_{i}^{t + 1} = \left\{ {\begin{array}{*{20}c} {U_{i}^{t} {\text{ if }}fitness\_value\left( {U_{i}^{t} } \right) \ge \, fitness\_value\left( {M_{i}^{t} } \right)} \\ {M_{i}^{t} {\text{ otherwise}}{. }} \\ \end{array} } \right.$$

In this study, the fitness value was defined as a combination of two RIMs, leverage [30], lev and improvement [31], imp:

6 $$fitness\_value\left( {M_{i}^{t} } \right) = \sum\limits_{r} {\left[ {{{lev\left( r \right)} \mathord{\left/ {\vphantom {{lev\left( r \right)} {30}}} \right. \kern-\nulldelimiterspace} {30}} + imp\left( r \right)} \right]} ,r{\text{ belongs to }}M_{i}^{t} ,lev\left( r \right) > 0,imp\left( r \right) > 0.$$

That is, the fitness value is equal to summed leverages over 30 plus summed improvements of all rules belonging to $M_{i}^{t}$ that have both a positive leverage and a positive improvement. The same applies to $U_{i}^{t}$. The leverage and improvement are computed by:

7 $$lev\left( {r:X \to Y} \right) = supp\left( {X \to Y} \right) - {{supp\left( X \right)supp\left( Y \right)} \mathord{\left/ {\vphantom {{supp\left( X \right)supp\left( Y \right)} {\left| D \right|}}} \right. \kern-\nulldelimiterspace} {\left| D \right|}},$$

8 $$imp\left( {X \to Y} \right) = conf\left( {X \to Y} \right) - \mathop {\max }\limits_{Z \subset X} \left( {conf\left( {Z \to Y} \right)} \right) > 0,$$

where

9 $$supp\left( {X \to Y} \right) = supp\left( {X \cup Y} \right) = \left| {R \in D:X \cup Y \subseteq R} \right|,$$

10 $$conf\left( {X \to Y} \right) = {{supp\left( {X \to Y} \right)} \mathord{\left/ {\vphantom {{supp\left( {X \to Y} \right)} {supp\left( X \right)}}} \right. \kern-\nulldelimiterspace} {supp\left( X \right)}}.$$

*X* and *Y* are the antecedent and consequent of the rule *r*; *supp* and *conf* denote support and confidence, two basic RIMs in ARM. |*D*│is the number of records in the dataset *D*, │*D*│ = 214 in this study. Let $X = \left\{ {{}^\backprime v_{1} = I_{1}^{\prime } , \ldots ,^{\prime}v_{m} = I_{m}^{\prime } } \right\}$, where *v*_1_,…,*v*_*m*_ are a series of variables, and *I*_1_,…,*I*_*m*_ are the intervals of *v*_1_,…,*v*_*m*_ in *X*. Then

11 $$supp\left( X \right) = \sum\limits_{R \in D} {\mu_{{I_{1} }} \left( {v_{R1} } \right) \ldots \mu_{{I_{m} }} \left( {v_{Rm} } \right)} ,$$

where *v*_*R*1_,…,*v*_*Rm*_ are numerical values for *v*_1_…*v*_*m*_ in record *R* of dataset D. The same applies to *supp* (*Y*) and *supp* (*X → Y*).

A positive leverage means that there were more TPUs with the values of all variables contained by the rule falling in their value ranges specified in the rule, compared with if the antecedent and consequent of the rule is independent. A positive improvement indicates that every explanatory variable increased the confidence of the rule. For example, if the rule “prop_higher_edu > 0.364 (p85) → rate_imported12 > 0.124 (p71)” has *lev* = 15.8 and *imp* = 0.49, then among the TPUs with higher-education rate above 0.364, about 15.8 more TPUs had wave-1&2 imported case rates above 0.124‰, compared with if the higher-education rate and confirmed case rate were irrelevant. Also, the probability for TPUs with high higher-education rate to have high wave-1&2 imported case rates was 49% higher than average TPUs. Leverage is a portion of the data size and normally much larger than 1, while improvement is normally only a small fraction of 1. Therefore, leverage was divided by 30 in (6), to balance the weight of the two RIMs in the computation of the fitness values.

***Step*** ***2.4.*** ***Opposition-based*** ***generation*** ***jumping.*** This step is to prevent the population from being trapped in local optima and, thus, failing to search for better data discretization schemes. Each generation has a probability *Jr* to conduct the generation jumping, instead of mutation and crossover. From each individual in current population *P*, an opposite individual is generated, by replacing each number *x* in the original individual with $\mathop x\limits^{ \cup }$:

12 $$\mathop x\limits^{ \cup } = rank^{ - 1} \left( {1 + \left| D \right| - rank\left( x \right)} \right),$$

where *rank*(*x*) is the rank of *x* among all data values of the variable (e.g., elderly rates of all different TPUs); and *rank*^−1^(*r*) is the data value with rank *r* among all data values of this variable. All the *N*_*P*_ opposite individuals form an opposite population *OP*, and *N*_*P*_ individuals with the highest fitness values in *OP* ∪ *P* are selected to survive to the next generation.

The following values of the DE parameters were used in this study: *P* = 300 and 100 for rules about the confirmed case rate and the waiting period, respectively; *G* = 3000; *Cr* = 0.5; *F* = 0.5; *Jr* = 0.04. The *P* and *G* values were such determined that the optimization result generally converged, that is, the number of rules and fitness values almost stayed the same with more generations. The *Cr*, *F*, *Jr* values were such determined to speed up the convergence of the optimization result. The maximum number of variables in antecedent of a rule, *maxL*, was set to 3 to allow the combined association of up to three explanatory variables on the response variables to be analyzed. The minimum fraction of transition in the fuzzy sets, *ft*_min_, was set to 0.5, following the study proposing DESigFAR [15]. The explanation of *ft*_min_ is detailed in [15]. Also, the relative RIM values among the rules were not sensitive to the *ft*_min_ value.

***Step*** ***3.*** Statistical evaluation and result output. After the DE, rules with positive improvement and leverage values are extracted from the optimized individuals as the ARM result. In this study, chi-square test was conducted on the statistical significance of positive improvement of each rule *X* → *Y*, that is, *imp*(*X* → *Y*) > 0. Following [15], a simplified test was conducted with

13 $$\begin{gathered} {\text{Null hypothesis }}H_{{0}} :\exists x_{m} \in X{, }\Pr \left( {Y|X} \right) \le \Pr \left( {Y|X\backslash \left\{ {x_{m} } \right\}} \right) \\ {\text{Alternative hypothesis }}H_{{1}} :\forall x_{m} \in X{, }\Pr \left( {Y|X} \right) > \Pr \left( {Y|X\backslash \left\{ {x_{m} } \right\}} \right) \\ \end{gathered}$$

For each fuzzy value interval of explanatory variable $I_{m} \in X$,

14 $$\chi_{m}^{2} = \frac{{\left( {ad - bc} \right)\left( {a + b + c + d} \right)}}{{\left( {a + b} \right)\left( {c + d} \right)\left( {a + c} \right)\left( {b + d} \right)}},$$

where

15 $$\begin{gathered} a = supp\left( {X \cup \left\{ Y \right\}} \right) \hfill \\ b = supp\left( {X \cup \neg \left\{ Y \right\}} \right) \hfill \\ c = supp\left( {\left( {X\backslash \left\{ {I_{m} } \right\}} \right) \cup \neg \left\{ {I_{m} } \right\} \cup \left\{ Y \right\}} \right) \hfill \\ d = supp\left( {\left( {X\backslash \left\{ {I_{m} } \right\}} \right) \cup \neg \left\{ {I_{m} } \right\} \cup \neg \left\{ Y \right\}} \right), \hfill \\ \end{gathered}$$

and ¬ means to that the corresponding explanatory variable value of the TPU was not in the interval defined in *I*_*m*_ or *Y*. For each $\chi_{m}^{2}$, a *P* value was looked up from the chi-square table with one degree of freedom. The final *P* value of the rule was equal to the largest *P* value resultant from all $I_{m} \in X$.

To make the resultant rules more readable, each fuzzy interval *I* of variable *v* is represented by a crisp interval of *v*, where *μ*_*I*_(*v*) is the largest among the membership degrees of different fuzzy intervals in *v*. For instance, the fuzzy interval for “low higher-education rate” in Fig. 2 is represented as “higher-education rate < 0.15” in resultant rules.

Two datasets were generated to contain each of the five confirmed case rate response variables, one with the POI density variables and the other with per-capita POI accessibility, together with all other explanatory variables. This was to avoid the possible confusion caused by the appearance of both the density and per-capita accessibility of a POI type in the same rule. Also, two datasets were generated to contain the response variables of average and median waiting period, the per-capita POI accessibility, and other explanatory variables. This resulted in a total of 12 datasets. Due to the randomness in DE, DESigFAR results in slightly different rules each time it is applied on the same dataset. Thus, on each of the 12 datasets, DESigFAR was ran for 10 times and output 10 sets of resultant rules. The set of rules containing the largest number of rules for “high confirmed case rate” or “long waiting period” was selected as the final result.

## Results

The rules resulting from the modified DESigFAR algorithm, together with their RIM and *P* values, are shown in Table 2. The strength of the rules was evaluated by two RIMs, leverage and improvement. As stated in Methods, all resultant rules had positive values for both the RIMs. In this case, two variables had an overall positive association, if a high value of one variable was associated with a high value of the other and the same went for the low value. Two variables had an overall negative association, if a high value of one variable is associated with a low value of the other and vice versa.

**Table 2:** Selected resultant rules from the ARM algorithm DESigFAR

(a) Rules for wave-1&2 local case rate; POI accessibility was used							*P*
	Antecedent	Consequent	Supp	Conf	Lev	Imp	
1	prop_higher_edu > 0.361 (p85)	rate_local12 > 0.143 (p86)	10.73	0.33	5.98	0.18	3.34E−05
2	med_income > 18,518.024 (p62)	rate_local12 > 0.136 (p85)	20.40	0.24	7.24	0.09	5.54E−05
3	med_area_home > 16.093 (p47)	rate_local12 > 0.136 (p85)	26.44	0.23	8.43	0.07	6.08E−05
4	gender_ratio > 106.692 (p92)	rate_local12 > 0.107 (p81)	9.88	0.57	6.33	0.36	1.48E−05
5	prop_over65 > 0.204 (p84)	rate_local12 < 0.005 (p45)	25.42	0.76	10.35	0.31	6.96E−05
6	prop_private_resid > 0.101 (p66)	rate_local12 > 0.046 (p63)	39.60	0.52	11.15	0.15	2.84E−06
7	prop_private_resid < 0.011 (p28)	rate_local12 < 0.002 (p45)	43.51	0.74	16.71	0.28	1.14E−07
8	prop_industrial < 0.001 (p47)	rate_local12 > 0.104 (p80)	33.96	0.34	12.94	0.13	3.96E−06
9	LU_entropy < 0.543 (p46)	rate_local12 > 0.106 (p80)	29.65	0.30	9.50	0.10	6.50E−05
**10**	LU_entropy > 0.543 (p46)	rate_local12 = 0.006–0.106 (p45-80)	54.87	0.47	15.52	0.13	1.60E−06
**11**	prop_rural_set < 0.008 (p56)	rate_local12 > 0.038 (p60)	61.34	0.52	13.81	0.12	6.93E−05

**(b)** Rules for wave-1&2 imported case rate; POI accessibility was used							
	Antecedent	Consequent	Supp	Conf	Lev	Imp	*P*
1	prop_higher_edu > 0.364 (p85)	rate_imported12 > 0.124 (p71)	24.89	0.78	15.77	0.49	6.85E−11
2	prop_higher_edu < 0.170 (p37)	rate_imported12 < 0.039 (p50)	57.65	0.73	17.91	0.23	3.65E−07
3	med_income > 19,583.009 (p65)	rate_imported12 > 0.110 (p70)	44.69	0.62	22.24	0.31	1.22E-15
4	med_income < 19,583.009 (p65)	rate_imported12 < 0.110 (p70)	120.22	0.85	22.51	0.16	1.22E-15
5	med_area_home > 25.388 (p86)	rate_imported12 > 0.169 (p78)	19.27	0.66	12.70	0.44	1.69E-12
6	gender_ratio > 94.842 (p64)	rate_imported12 > 0.207 (p81)	26.22	0.32	10.36	0.13	6.60E−05
7	prop_over65 < 0.140 (p35)	rate_imported12 > 0.083 (p65)	38.57	0.53	11.66	0.16	5.07E−05
8	prop_over65 > 0.140 (p35)	rate_imported12 < 0.083 (p65)	100.87	0.72	11.69	0.08	5.07E−05
9	ave_household_size > 3.250 (p87)	rate_imported12 > 0.110 (p70)	20.27	0.76	11.98	0.45	6.18E−09
10	den_population = 673.564–26,483.860 (p13-60)	rate_imported12 > 0.084 (p66)	46.61	0.48	11.11	0.11	2.65E−05
11	den_public_trans < 3.592 (p25)	rate_imported12 < 0.004 (p36)	33.98	0.63	14.57	0.27	1.77E−06
12	den_population < 673.564 (p13)	rate_imported12 < 0.004 (p36)	18.89	0.70	9.22	0.34	3.94E−06
13	build_area_pp > 75.116 (p54) &prop_private_resid > 0.239 (p85)	rate_imported12 > 0.184 (p80)	8.11	0.85	6.08	0.49	6.97E−05
14	prop_private_resid < 0.002 (p19)	rate_imported12 < 0.005 (p36)	30.69	0.75	16.01	0.39	4.10E−09
15	prop_publicResid < 0.034 (p69)	rate_imported12 > 0.103 (p69)	62.20	0.43	15.37	0.11	1.72E−08
16	prop_industrial < 0.000 (p43)	rate_imported12 > 0.097 (p68)	44.29	0.49	13.89	0.15	1.39E−06
17	LU_entropy < 0.561 (p51)	rate_imported12 > 0.099 (p68)	48.70	0.45	12.73	0.12	5.05E−05
18	LU_entropy > 0.561 (p51)	rate_imported12 = 0.001–0.099 (p36-68)	46.64	0.44	13.70	0.13	6.21E−05
19	build_area_pp = 51.859–202.851 (p43-89)	rate_imported12 > 0.105 (p69)	47.53	0.48	15.73	0.16	3.57E−07
20	build_area_pp > 71.028 (p53) &prop_rural_set > 0.026 (p66)	rate_imported12 < 0.004 (p36)	26.61	0.69	12.69	0.22	1.88E−05
21	prop_agricultural > 0.055 (p81)	rate_imported12 < 0.003 (p36)	25.97	0.63	11.25	0.27	2.23E−05
22	prop_gov_insti_faci > 0.030 (p41)	rate_imported12 = 0.003–0.124 (p36-71)	62.21	0.49	16.38	0.13	3.90E−08
23	prop_transport > 0.121 (p50)	rate_imported12 = 0.004–0.080 (p36-64)	43.70	0.40	12.51	0.11	4.34E−05
24	POI_pp_sports > 30.523 (p67)	rate_imported12 > 0.196 (p80)	28.89	0.41	14.60	0.21	5.62E−08
25	POI_pp_telecom_elec > 25.431 (p76)	rate_imported12 > 0.213 (p81)	24.73	0.46	14.51	0.27	5.34E−10
26	POI_pp_transport > 39.200 (p61)	rate_imported12 > 0.188 (p80)	32.28	0.37	14.25	0.17	1.27E−07
27	POI_pp_mall_mkt > 10.941 (p75)	rate_imported12 > 0.202 (p80)	25.16	0.45	14.06	0.25	1.15E−08
28	POI_pp_edu > 22.306 (p79)	rate_imported12 > 0.187 (p80)	22.41	0.48	12.67	0.27	2.78E−08
29	POI_pp_telecom_elec < 6.513 (p36)	rate_imported12 = 0.004–0.091 (p36-67)	48.88	0.64	25.23	0.33	1.78E−15
30	POI_pp_sports < 6.556 (p22)	rate_imported12 = 0.004–0.108 (p36-70)	37.48	0.77	21.29	0.44	1.32E−13
31	POI_pp_edu < 3.533 (p20)	rate_imported12 = 0.003–0.078 (p36-64)	32.83	0.75	20.52	0.47	8.59E−14
32	POI_pp_mall_mkt < 1.922 (p22)	rate_imported12 = 0.003–0.083 (p36-66)	33.73	0.73	20.19	0.44	8.37E−12
33	POI_pp_transport < 4.899 (p9)	rate_imported12 = 0.003–0.084 (p36-66)	18.09	0.89	12.07	0.59	1.59E−08
34	POI_pp_transport = 4.899–8.368 (p9-19)	rate_imported12 = 0.003–0.084 (p36-66)	14.37	0.74	8.63	0.44	1.21E−05

**(c)** Rules for wave-3 local case rate; POI accessibility was used							
	Antecedent	Consequent	Supp	Conf	Lev	Imp	*P*
1	prop_higher_edu < 0.237 (p62)	rate_local3 > 0.335 (p53)	75.42	0.57	14.15	0.11	7.00E−06
2	prop_higher_edu > 0.237 (p62)	rate_local3 < 0.335 (p53)	58.31	0.71	13.93	0.17	7.00E−06
3	med_income < 15,406.084 (p39)	rate_local3 > 0.335 (p53)	57.08	0.66	16.75	0.19	1.49E−06
4	med_income > 24,009.220 (p79)	rate_local3 < 0.335 (p53)	38.57	0.84	13.99	0.31	3.40E−07
5	med_area_home < 13.826 (p27)	rate_local3 > 0.340 (p54)	43.20	0.76	16.91	0.30	1.26E−08
6	med_area_home > 19.838 (p65)	rate_local3 < 0.340 (p54)	56.62	0.75	15.50	0.20	5.08E−08
7	prop_over65 > 0.141 (p36)	rate_local3 > 0.274 (p47)	85.51	0.61	10.65	0.08	8.15E−05
8	prop_over65 < 0.141 (p36)	rate_local3 < 0.274 (p47)	44.95	0.61	10.53	0.14	8.15E−05
9	ave_household_size < 2.580 (p17)	rate_local3 > 0.621 (p79)	18.74	0.43	9.54	0.22	2.66E−05
10	ave_household_size > 2.925 (p63)	rate_local3 < 0.621 (p79)	78.93	0.91	9.95	0.11	8.13E−05
11	den_population > 21,684.808 (p53)	rate_local3 > 0.267 (p47)	68.18	0.69	14.02	0.14	3.31E−05
12	den_road > 9.024 (p43)	rate_local3 > 0.407 (p59)	59.85	0.49	11.28	0.09	5.28E−05
13	den_bldg > 0.136 (p43)	rate_local3 > 0.411 (p59)	59.27	0.47	10.35	0.08	8.32E−05
14	den_population < 21,684.808 (p53)	rate_local3 < 0.267 (p47)	66.24	0.58	14.34	0.13	3.31E−05
15	den_public_trans < 4.083 (p28)	rate_local3 < 0.016 (p15)	23.50	0.40	14.24	0.24	9.33E-10
16	den_road < 9.024 (p43)	rate_local3 < 0.407 (p59)	66.36	0.73	11.35	0.12	5.28E−05
17	den_bldg < 0.136 (p43)	rate_local3 < 0.411 (p59)	64.66	0.72	10.38	0.12	8.32E−05
18	prop_higher_edu < 0.212 (p54) &prop_private_resid > 0.104 (p66)	rate_local3 > 0.372 (p57)	17.56	0.94	9.55	0.39	3.17E−05
19	prop_private_resid < 0.021 (p36)	rate_local3 < 0.041 (p16)	22.79	0.30	10.35	0.14	4.46E−05
20	prop_publicResid > 0.063 (p74)	rate_local3 > 0.295 (p50)	40.41	0.74	12.49	0.23	6.57E−05
21	prop_publicResid < 0.063 (p74)	rate_local3 < 0.295 (p50)	90.45	0.57	12.43	0.08	6.57E−05
22	med_income < 18,723.236 (p62) &prop_rural_set < 0.000 (p44) &prop_industrial < 0.008 (p69)	rate_local3 > 0.428 (p63)	29.28	0.77	15.05	0.15	6.34E−05
23	build_area_pp < 61.380 (p50)	rate_local3 > 0.222 (p38)	75.64	0.75	14.46	0.14	6.15E−06
24	build_area_pp > 61.380 (p50)	rate_local3 < 0.222 (p38)	58.89	0.52	14.37	0.13	6.15E−06
25	med_income < 23,040.421 (p78) &prop_agricultural < 0.028 (p72)	rate_local3 > 0.335 (p53)	72.65	0.64	20.12	0.09	5.81E−05
26	ave_household_size < 2.636 (p29) &prop_rural_set < 0.001 (p46)	rate_local3 > 0.493 (p68)	25.71	0.65	13.15	0.17	5.45E−05
27	prop_shrubland < 0.009 (p30)	rate_local3 > 0.570 (p76)	28.29	0.43	12.25	0.19	1.27E−05
28	prop_woodland < 0.012 (p22)	rate_local3 > 0.409 (p59)	30.91	0.65	12.19	0.26	1.01E−05
29	prop_grassland < 0.000 (p16)	rate_local3 > 0.500 (p68)	21.99	0.64	11.34	0.33	1.08E−05
30	prop_woodland > 0.322 (p82)	rate_local3 < 0.409 (p59)	34.03	0.87	10.45	0.27	3.92E−05
31	prop_shrubland > 0.281 (p89)	rate_local3 < 0.166 (p30)	16.40	0.71	9.31	0.40	1.54E−06
32	prop_open_recreation < 0.002 (p14)	rate_local3 < 0.016 (p15)	13.07	0.44	8.31	0.28	9.22E−06
33	med_income < 17,081.439 (p53) &prop_open_recreation > 0.026 (p45)	rate_local3 > 0.352 (p56)	49.42	0.71	18.23	0.11	1.03E−05
34	prop_higher_edu < 0.224 (p58) &prop_gov_insti_faci > 0.027 (p38)	rate_local3 > 0.297 (p50)	54.45	0.74	17.12	0.12	6.17E−05
35	prop_transport > 0.141 (p55)	rate_local3 > 0.382 (p57)	54.56	0.56	13.89	0.14	4.17E−05
36	prop_business > 0.039 (p84)	rate_local3 > 0.518 (p71)	19.10	0.54	8.80	0.25	2.87E−05
37	prop_higher_edu < 0.222 (p58) &POI_pp_sports < 12.643 (p42)	rate_local3 > 0.277 (p47)	49.14	0.79	16.23	0.13	7.74E−05
38	med_income < 19,861.505 (p65) &POI_pp_edu < 19.291 (p73)	rate_local3 > 0.180 (p33)	94.43	0.80	15.87	0.06	2.84E−05
39	prop_higher_edu < 0.228 (p61) &POI_pp_mall_mkt < 9.663 (p69)	rate_local3 > 0.233 (p39)	75.07	0.74	15.46	0.06	4.66E−05
40	POI_pp_transport < 16.809 (p38)	rate_local3 > 0.191 (p35)	65.98	0.84	14.48	0.18	1.67E−07
41	POI_pp_telecom_elec < 6.955 (p37)	rate_local3 > 0.193 (p35)	65.22	0.82	13.81	0.17	2.01E−06

**(d)** Rules for wave-4 local case rate; POI accessibility was used							
	Antecedent	Consequent	Supp	Conf	Lev	Imp	*P*
1	med_income < 12,559.030 (p7)	rate_local4 > 0.831 (p79)	8.91	0.64	5.86	0.42	2.82E−05
2	med_income < 29,532.689 (p84) &prop_grassland < 0.007 (p31)	rate_local4 > 0.852 (p80)	30.41	0.62	20.17	0.13	4.52E−05
3	med_income < 23,878.185 (p79) &den_bldg > 0.293 (p72)	rate_local4 > 0.774 (p75)	29.22	0.65	17.99	0.13	5.12E−05
4	med_area_home < 15.641 (p45)	rate_local4 > 0.299 (p29)	74.53	0.81	9.64	0.10	7.30E−05
5	med_area_home > 15.641 (p45)	rate_local4 < 0.299 (p29)	45.56	0.37	9.68	0.08	7.30E−05
6	gender_ratio > 109.623 (p94)	rate_local4 < 0.334 (p34)	10.16	0.79	5.84	0.45	5.99E−05
7	prop_over65 > 0.229 (p93)	rate_local4 < 0.299 (p29)	11.05	0.78	6.81	0.48	2.32E−05
8	ave_household_size < 2.713 (p37)	rate_local4 > 0.865 (p80)	27.87	0.38	12.90	0.17	2.34E−07
9	den_road > 16.383 (p71)	rate_local4 > 0.722 (p72)	36.83	0.60	19.40	0.31	4.63E−12
10	den_bldg > 0.226 (p62)	rate_local4 > 0.603 (p64)	50.39	0.60	18.56	0.22	3.09E−07
11	den_population > 26,219.620 (p60)	rate_local4 > 0.466 (p46)	63.71	0.71	16.81	0.19	1.21E−06
12	den_public_trans > 95.120 (p92)	rate_local4 > 0.780 (p76)	12.45	0.67	7.89	0.42	1.19E−05
13	den_road < 16.383 (p71)	rate_local4 < 0.722 (p72)	128.58	0.84	19.36	0.13	4.63E-12
14	den_bldg < 0.226 (p62)	rate_local4 < 0.603 (p64)	99.30	0.76	18.44	0.14	3.09E−07
15	den_population < 26,219.620 (p60)	rate_local4 < 0.466 (p46)	75.80	0.61	16.78	0.14	1.21E−06
16	den_public_trans < 46.130 (p78)	rate_local4 < 0.780 (p76)	136.41	0.85	15.26	0.10	4.31E-10
17	prop_private_resid > 0.087 (p64)	rate_local4 > 0.731 (p73)	36.23	0.45	13.84	0.17	1.00E−06
18	prop_private_resid < 0.022 (p36)	rate_local4 < 0.117 (p14)	22.60	0.29	11.64	0.15	2.50E−08
19	prop_publicResid > 0.042 (p70)	rate_local4 = 0.168–1.073 (p16-87)	56.16	0.86	10.24	0.16	3.66E−05
20	prop_industrial < 0.000 (p36)	rate_local4 > 1.033 (p86)	23.82	0.31	12.74	0.17	2.01E−07
21	LU_entropy < 0.548 (p47)	rate_local4 > 0.863 (p80)	34.55	0.34	14.21	0.14	1.67E−07
22	LU_entropy > 0.548 (p47)	rate_local4 = 0.140–0.863 (p14-80)	90.44	0.80	17.54	0.15	3.55E−08
23	build_area_pp < 58.100 (p47)	rate_local4 > 0.222 (p21)	83.55	0.87	10.09	0.11	1.52E−05
24	build_area_pp > 58.100 (p47)	rate_local4 < 0.222 (p21)	36.65	0.31	9.66	0.08	1.52E−05
25	prop_rural_set < 0.001 (p46)	rate_local4 > 0.630 (p64)	50.56	0.52	15.79	0.16	4.68E−05
26	prop_agricultural > 0.017 (p64)	rate_local4 < 0.430 (p43)	46.73	0.61	13.80	0.18	5.87E−06
27	prop_shrubland < 0.000 (p0)	rate_local4 > 0.785 (p76)	27.11	0.71	17.92	0.47	4.93E−14
28	prop_agricultural < 0.001 (p40)	rate_local4 > 0.766 (p74)	39.90	0.46	17.43	0.20	1.13E−08
29	prop_grassland < 0.004 (p23)	rate_local4 > 0.848 (p80)	28.16	0.56	17.42	0.34	1.39E-13
30	prop_woodland < 0.016 (p25)	rate_local4 > 1.282 (p90)	15.27	0.29	10.03	0.19	3.76E−08
31	prop_shrubland > 0.064 (p49)	rate_local4 < 0.785 (p76)	101.16	0.90	15.67	0.14	2.93E−07
32	prop_grassland > 0.085 (p71)	rate_local4 < 0.188 (p16)	20.40	0.32	9.55	0.15	6.50E−06
33	prop_open_recreation > 0.030 (p48) &LU_entropy < 0.551 (p49)	rate_local4 > 0.804 (p78)	26.92	0.55	15.70	0.18	7.19E−06
34	prop_gov_insti_faci > 0.025 (p36) &LU_entropy < 0.546 (p47)	rate_local4 > 0.765 (p74)	29.27	0.51	14.66	0.11	5.00E−05
35	prop_transport > 0.241 (p71)	rate_local4 > 0.897 (p81)	25.98	0.43	14.55	0.24	3.65E−08
36	prop_business > 0.058 (p86)	rate_local4 > 0.784 (p76)	18.49	0.65	11.61	0.41	1.45E−08
37	med_income < 24,082.337 (p79) &prop_rural_set < 0.001 (p46) &POI_pp_sports > 19.996 (p53)	rate_local4 > 0.827 (p79)	19.48	0.68	13.18	0.22	4.62E−05
38	med_income < 24,082.337 (p79) &den_road > 10.205 (p48) &POI_pp_mall_mkt > 6.459 (p57)	rate_local4 > 0.895 (p81)	18.62	0.63	13.02	0.22	4.56E−05
39	med_income < 23,241.294 (p78) &prop_rural_set < 0.002 (p49) &POI_pp_transport > 32.416 (p54)	rate_local4 > 0.815 (p78)	18.37	0.70	12.49	0.24	6.88E−05
40	POI_pp_sports > 33.191 (p70)	rate_local4 < 0.062 (p13)	22.58	0.35	14.17	0.22	8.48E−12
41	POI_pp_telecom_elec > 30.272 (p80)	rate_local4 < 0.039 (p13)	19.74	0.45	14.04	0.32	3.55E−13
42	POI_pp_transport > 68.907 (p80)	rate_local4 < 0.048 (p13)	19.71	0.45	14.01	0.32	7.44E−15
43	POI_pp_edu > 25.063 (p83)	rate_local4 < 0.061 (p13)	15.92	0.40	10.72	0.27	2.21E−09
44	POI_pp_mall_mkt > 19.264 (p88)	rate_local4 < 0.071 (p13)	12.77	0.48	9.31	0.35	2.60E−09

**(e)** Rules for wave-4 local case rate, excluding cases in the Dancing/Singing Cluster; POI accessibility was used							
	Antecedent	Consequent	Supp	Conf	Lev	Imp	*P*
1	prop_higher_edu < 0.346 (p82) &prop_rural_set < 0.000 (p43)	rate_local4nondance > 0.389 (p53)	48.36	0.76	18.27	0.14	2.36E−05
2	prop_higher_edu < 0.342 (p81) &den_road > 11.532 (p53)	rate_local4nondance > 0.421 (p58)	49.83	0.68	17.91	0.09	2.44E−05

**(f)** Rules for wave-3 local case rate; POI density was used							
	Antecedent	Consequent	Supp	Conf	Lev	Imp	*P*
1	POI_den_mall_mkt > 307.693 (p78)	rate_local3 > 0.547 (p72)	24.39	0.53	12.21	0.26	5.24E−06
2	POI_den_sports > 882.075 (p78)	rate_local3 > 0.528 (p71)	24.72	0.55	11.96	0.26	4.03E−06
3	POI_den_telecom_elec > 540.287 (p78)	rate_local3 > 0.539 (p71)	24.30	0.53	11.95	0.26	2.43E−06
4	POI_den_transport > 1382.534 (p79)	rate_local3 > 0.530 (p71)	24.57	0.54	11.70	0.26	1.18E−05
5	POI_den_edu > 611.274 (p81)	rate_local3 > 0.558 (p74)	21.47	0.51	10.87	0.26	2.04E−05

**(g)** Rules for wave-4 local case rate; POI density was used							
	Antecedent	Consequent	Supp	Conf	Lev	Imp	*P*
1	POI_den_transport > 904.959 (p71)	rate_local4 > 0.726 (p73)	36.27	0.60	19.27	0.32	1.95E−14
2	POI_den_mall_mkt > 208.752 (p73)	rate_local4 > 0.753 (p74)	34.64	0.58	19.11	0.32	1.82E−11
3	POI_den_sports > 598.644 (p72)	rate_local4 > 0.751 (p74)	34.76	0.58	19.11	0.32	9.18E−12
4	POI_den_telecom_elec > 374.340 (p72)	rate_local4 > 0.741 (p74)	34.54	0.58	18.63	0.31	9.93E−12
5	POI_den_edu > 1372.584 (p94)	rate_local4 > 0.751 (p74)	10.59	0.79	7.09	0.53	5.28E−07

**(h)** Rules for average waiting period; POI accessibility was used							
	Antecedent	Consequent	Supp	Conf	Lev	Imp	*P*
1	POI_pp_transport > 39.683 (p60)	ave_waiting_period > 5.790 (p75)	22.98	0.34	9.81	0.14	6.65E−05
2	POI_pp_telecom_elec > 15.395 (p57)	ave_waiting_period > 5.765 (p74)	24.76	0.33	9.65	0.13	7.16E−05
3	POI_pp_mall_mkt > 8.676 (p64)	ave_waiting_period > 5.822 (p75)	20.26	0.33	8.90	0.14	0.0001
4	med_area_home > 17.644 (p50)	ave_waiting_period > 5.807 (p75)	26.33	0.29	8.89	0.10	1.14E−05
5	med_income > 19,979.413 (p60)	ave_waiting_period > 5.751 (p74)	21.18	0.33	8.39	0.13	9.48E−05
6	POI_pp_sports > 29.352 (p65)	ave_waiting_period > 6.163 (p81)	16.16	0.27	8.26	0.14	1.53E−07
7	POI_pp_edu > 14.015 (p63)	ave_waiting_period > 5.788 (p75)	20.68	0.31	7.75	0.12	0.0002

**(i)** Rules for median waiting period; POI accessibility was used							
	Antecedent	Consequent	Supp	Conf	Lev	Imp	*P*
1	med_area_home > 18.602 (p52)	med_waiting_period > 4.392 (p67)	31.27	0.38	10.07	0.12	0.0002
2	med_income > 19,680.813 (p60)	med_waiting_period > 5.030 (p82)	17.80	0.27	7.39	0.11	0.0002
3	build_area_pp > 57.721 (p46)	med_waiting_period > 5.317 (p82)	19.22	0.18	6.54	0.06	0.0002

For clarity, the antecedent and consequent of each rule are put in separate columns. For example, rule 1 in Table 2a is “prop_higher_edu > 0.361 (p85) → rate_local12 > 0.143 (p86)”, where p85 means the 85^th^ percentile of the variable among the 214 TPU-level areal units in Hong Kong. Variables in the rules are described in Table 1. Rules are shown together with the values of four RIMs: support (supp), confidence (conf), leverage (lev), and improvement (imp). *P* is the *P* value of each rule in the chi-square test described in the Methods. The full sets of resultant rules are shown in Table S2 in Additional File 1

### Demographic and socioeconomic characteristics

Among all urban characteristics, high values of higher-education rate, median monthly income, and average accommodation area had the strongest and most significant associations with a high wave-1&2 imported confirmed case rate, in terms of the largest leverage and smallest *P* values (rule 1, 3, 5, Table 2b). Among all explanatory variables, higher-education rate and median monthly income also had the largest positive Spearman rank-order correlation coefficient values between the wave-1 imported case rate, which were 0.52 and 0.48, respectively. Corresponding to rule 5, Table 2b, all 21 TPUs with median accommodation areas over 25.4 m^2^/person and imported case rates over 0.169‰ had median incomes of at least 25,000 HKD/month (78^th^ percentile in all TPUs), showing that this association also came from high-income population, instead of large housings in low-density rural areas. The wave-1&2 local case rate had similarly positive but weaker associations with these three variables, in terms of smaller leverage and improvement values (rule 1–3, Table 2a).

The wave-3 local case rate, on contrary, was negatively associated with higher-education rate, income, and accommodation area (rule 1–6, Table 2c), showing that the cases tended to be occur in lower-SES population. In wave 4, the accommodation area continued being negatively associated with the local case rate (rule 4–5, Table 2d), but the association between income and the local cases rate became much weaker and involved only the 7% TPUs with the lowest income (rule 1, Table 2d). Combined with indicators of urban area (e.g., low vegetation coverage, high building density), income and the local cases rate became negatively associated in more TPUs, as reflected by the larger supports of rule 2–3 than rule 1, Table 2d. Higher-education rate was negatively associated with the wave-4 local case rate only if excluding the cases from the Dancing/Singing Cluster and in urban area (rule 1–2, Table 2e). This shows that the wave-4 local cases were less concentrated in lower-SES population than the wave-3 cases but more concentrated in the urban area.

A high gender ratio of over 94.8, which seemed also related to a high income, was associated with a high wave-1&2 imported case rate of over 0.207‰ (rule 6, Table 2b). In the 26 TPUs fulfilling rule 6, Table 2b, 23 TPUs had the income higher than Hong Kong median of HK$16,250/month (Table 1a). In wave 4, a very high gender ratio was associated with a low local case rate (rule 6, Table 2d), reflecting very sparsely populated TPUs. The 11 TPUs fulfilling rule 6, Table 2d had an average population density of 2,124 persons/Km^2^, much lower than the Hong Kong median of 18,402 persons/km^2^ (Table 1b).

Elderly rate showed a negative association with the wave-1&2 imported case rate (rule 7–8, Table 2b) but a positive association with wave-3 local case rate (rule 7–8, Table 2c). Meanwhile, the elderly rate had a considerable negative correlation with the monthly income, with a Spearman’s *r* value of -0.49 between the two variables. In wave 4, a very high elderly rate was associated with a low local case rate (rule 7, Table 2d), mostly reflecting TPUs with low population densities below 5,000 person/Km^2^.

A small average household size below 2.6–2.7 was associated with high local case rates (rule 9, Table 2c; rule 8, Table 2d). Oppositely, a high wave-1&2 imported case rate was associated with a large average household size above 3.25 (rule 9, Table 2b) which also tended to co-occur with a high income. The income of all 21 TPUs fulfilling rule 9, Table 2b was above the Hong Kong median of HK$16,250/month, with an average of HK$38,952/month.

### Density and connectivity characteristics

All four density and connectivity variables, namely the densities of population, buildings, roads, and public transport stations, showed generally positive associations with the wave-3 and wave-4 local case rates (rule 11–17, Table 2c; rule 9–16, Table 2d). Judged by larger leverage and improvement of the rules, the wave-3 local case rate was more strongly associated with population density, while the wave-4 rate was more strongly associated with road and building densities (rule 11–14, Table 2c; rule 9–12, Table 2d). The wave-4 rate was most statistically significantly associated with road density, that is, connectivity (*P* value in rule 9 compared with rule 10–12, Table 2d).

A high wave-1&2 imported case rate was, instead, associated with a low-to-medium population density. Also, very low density and connectivity were associated with low confirmed case rates (rule 10–12, Table 2b), which represented low-density rural TPUs with few imported cases.

### Functionality: urban residence and related variables

The proportion of private residential LU generally showed positive associations with the confirmed case rates in all waves (rule 6–7, Table 2a; rule 13–14, Table 2b; rule 18–19, Table 2c; rule 17–18, Table 2d). A low proportion of industrial land, which was usually far from major residential areas, was also associated with high confirmed case rates in all waves (rule 8, Table 2a; rule 16, Table 2b; rule 22, Table 2c; rule 20, Table 2d).

Public housings in Hong Kong were reserved for lower-income residents. A high proportion of public residential LU was associated with a high wave-3 local case rate but a medium wave-4 local case rate (rule 20–21, Table 2c; rule 19, Table 2d). A low proportion of public residential LU, indicating a higher income, was again associated with a high wave-1&2 imported case rate (rule 15, Table 2b).

The LU mix index value was negatively associated with the rates of wave-1&2 imported cases as well as wave-1&2 and wave-4 local cases (rule 9–10, Table 2a; rule 17–18, Table 2b; rule 21–22, Table 2d). This echoed the association between residential LU and the confirmed case rate, since TPUs with high proportions of residential area tended to have low LU mix index values. The TPUs ranked the lower half in terms of LU mix index had an average of 17.2% residential area, while the Hong Kong average was 11%. The negative association between the local case rate and LU mix index disappeared in wave 3, during which some New Towns and rural areas with high LU mix index values also had high local case rates (Fig. 1c, e). These areas had high LU mix because they contained both typical urban LUs (e.g., residential and business area) and typical suburban or rural LUs (e.g., rural settlement and agricultural land).

Average per-capita floor area (ave_area_all) was equal to the total floor area of buildings divided by the number of residents in the TPU. In the experimental data, high ave_area_all values appeared in industrial or hotel area, remote rural TPUs, and high-income TPUs, while lower ave_area_all values mainly occurred in densely populated lower-income TPUs. This variable showed overall negative associations with wave-3 and wave-4 local case rates (rule 23–24, Table 2c; rule 23–24, Table 2d). TPUs fulfilling these rules were mainly densely populated lower-income TPUs with higher local case rates. A mid-to-high ave_area_all value was associated with a high wave-1&2 imported rate, while a high ave_area_all value was associated with a low imported case rate (rule 19–20, Table 2b). Looking into the data, rule 19 and 20 largely corresponded to high-income TPUs and remote rural TPUs, respectively.

### Functionality: rural area, urban area, and POI density

In general, the proportion of LUs concentrating in rural area, including rural settlement, agriculture land, and vegetations (woodland, shrubland, and grassland), had negative associations with the confirmed case rates in all waves (rule 11, Table 2a; rule 21, Table 2b; rule 25–32, Table 2c; rule 25–32, Table 2d). As an exception, vegetation coverage had no obvious associations with the wave-1&2 confirmed case rates. Indeed, some high-income TPUs with high wave-1&2 imported case rate, especially those in hilly urban areas on Hong Kong Island, also had high vegetation coverage.

In contrast, the five types of POIs in this study, as well as business, recreation, governmental, institutions and facilities, and transport LUs, concentrated in urban areas. High densities of all five types of POIs and high proportions of all four LUs were associated with high wave-3 and wave-4 local case rates (rule 33–36, Table 2c; rule 33–36, Table 2d; Table 2f, g). Yet since these POIs and LUs also concentrated in densely populated areas, it was not very clear whether these associations were more related to the high level of activities brought by these POIs and LUs, or instead to the high population density.

### Functionality: POI accessibility

**Fig. 3:**
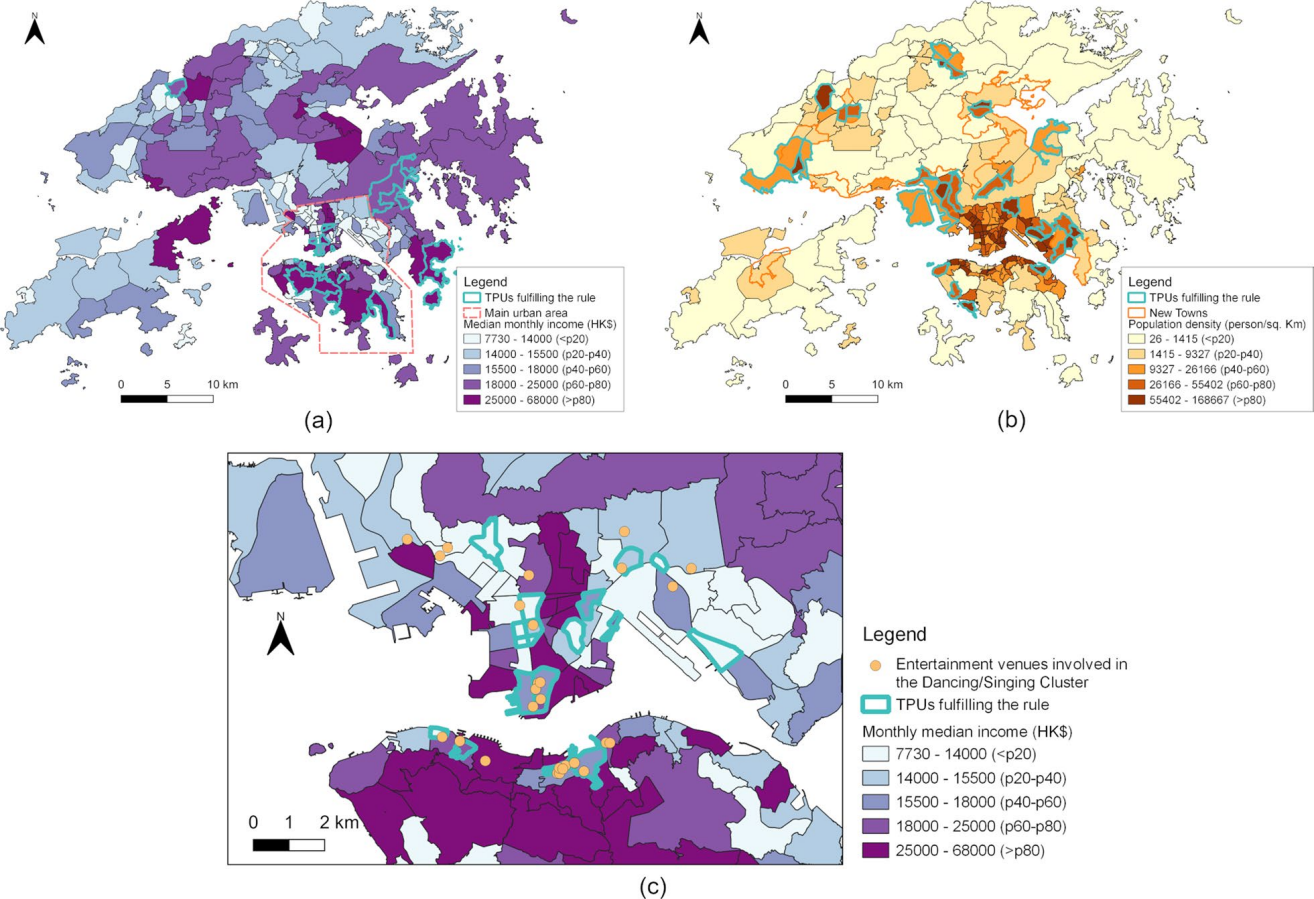
Exemplary rules. **a** TPUs fulfilling rule 27, Table 2b, i.e., TPUs with POI_pp_mall_mkt > 10.941 (p75) and rate_imported12 > 0.202 (p80), together with median monthly income. **b** TPUs fulfilling rule 32, Table 2b, i.e., TPUs with POI_pp_mall_mkt < 1.922 (p22) and rate_imported12 = 0.003–0.083 (p36-66), together with population density. **c** TPUs fulfilling rule 38, Table 2d, i.e., TPUs with med_income < 24,082.337 (p79), den_road > 10.205 (p48), POI_pp_mall_mkt > 6.459 (p57), and rate_local4 > 0.895 (p81), together with the median monthly income and entertainment venues involved in the super-spread of Dancing/Singing Cluster [32]

High per-capita accessibilities to all five types of POIs were associated with high wave-1&2 imported case rates (rule 24–28, Table 2b). These associations mostly reflected the wealthy areas in or around the downtown, which had convenient access to a great number of POIs in the downtown and also mid-to-low population density (Fig. 3a). Low per-capita POI accessibilities were associated with medium wave-1&2 imported case rates (rule 29–34, Table 2b), which mainly reflected some New Towns with mid-to-high population density and relatively limited access to POIs due to the farness to the main urban area (Fig. 3b).

Low or mid-to-low accessibilities to all five types of POIs, alone or combined with a relatively low income or higher-education rate, were associated with mid-to-high wave-3 local case rates (rule 37–41, Table 2c). Looking into the data, TPUs involved in these rules were largely densely populated lower-income urban areas, where the per-capita POI accessibility was low due to the large populations.

In wave 4, mid-to-low-income urban TPUs with high accessibilities to mall and market, sports, and transport POIs, in contrast to low accessibilities in wave 3, were associated with very high local case rate (rule 37–39, Table 2d). The associations mainly reflected the mid-to-low-income TPUs located in major commercial and entertainment areas and in adjacent to high-income TPUs (Fig. 3c). These TPUs also contained most entertainment venues involved in the super-spread of the Dancing/Singing Cluster (Fig. 3c), and their associations with very high wave-4 local case rate disappeared when the Dancing/Singing Cluster were excluded (Additional file 1: Table S2f).

In addition, very high per-capita POI accessibilities were associated with very low local case rates in all waves (rules 222, 324, 326, 329 and 332, Additional file 1: Table S2a; rules 169, 176, 180, 181 and 185, Additional file 1: Table S2c; rule 40–44, Table 2d). These rules mainly covered some remote TPUs with high per-capita POI accessibilities due to their small populations.

### Associations for the waiting period

A high median accommodation area and a high median income were associated with longer average and median waiting periods (Table 2h, i). Long average waiting periods were also associated with high per-capita POI accessibilities (Table 2h). As stated earlier, large median accommodation areas and high per-capita POI accessibilities tended to occur in high-income TPUs and sparsely populated rural TPUs. It is of note that high-income TPUs still contributed to a minority of cases with long waiting periods, since they had much smaller populations and number of confirmed cases than lower-income TPUs. For example, the TPUs with median incomes of at least HK$20,000/month (top 40% TPUs) contributed 1226 out of the 8,238 (14.9%) local cases with available waiting periods, and contributed 76 out of 357 cases (21.2%) with waiting periods of 12 or more days.

## Discussion

Interpreted from the ARM results, main characteristics of the first four waves of COVID-19 in Hong Kong are as follows. The first and second waves (by May 2020) tended to spread among higher-SES population, represented by higher income, higher education level, and more spacious accommodations. The rules related to high income and education level (rule 1 and 3, Table 2b) had higher RIM values and statistical significance than the rules related to other demographic or density characteristics, making SES more likely to be the driving force of the distribution of imported cases. The results, in contrary to some previous results at coarser spatial scales [7, 8] (Table 3), could be explained by that most wave-1&2 imported cases, who had studied or lived in developed countries, tended to have higher SES. Wave-1&2 imported cases also tended to distribute in neighborhoods with mid-to-low population density, agreeing to the fact that higher-SES people in Hong Kong tend to live in mid-to-low-density neighborhoods. The association between a high gender ratio, a low elderly rate, and a high confirmed case rate appeared to link to the higher male proportions and lower elderly rate in higher-SES TPUs, instead of gender or age difference in physiological susceptibility. These high-SES neighborhoods also had high accessibility to POIs, mainly attributed to POIs in nearby higher-density commercial and entertainment areas, since the POI density within high-SES neighborhoods was not usually high. The associations between urban characteristics and the local case rate were similar to but weaker than those for the imported case rate. These weaker associations might be shaped by the distribution of imported cases who were the infection sources for local cases, since local transmission was limited during the first two waves.

**Table 3:** Selected literature on associations between urban characteristics and COVID-19 confirmed case rate

References	Study area	Spatial scale	Study period	Method	Relationship with variables					
					Elderly rate	College-education rate	Economic condition	Population density	Poor housing conditions	Land use/Urban geometry/ Activities
Yang et al. [4]	Massachusetts, US	City/ town	As of Apr 29, 2020	Spatial lag model	-*	- (*P* = 0.05)	+ *	N/A	N/A	N/A
Sun et al. [5]	US	County	As of Jun 28, 2020	Ordinary least square (OLS), spatial lag, spatial error, and spatial autoregressive combined (SAC) model	-* (spatial lag, SAC)	N/A	Nonsig	+ * ~ + *** (all models)	Nonsig	N/A
Zhang & Gary [6]	US	County	As of May 1, 2020	Multiple linear regression	+ *	N/A	N/A	+ ***	N/A	N/A
Hamidi et al. [7]	913 metropolitan counties in the US	County	As of May 25, 2020	Pearson's *r*, structural equation modeling (SEM)	-** (*r*), -*** (SEM)	-* (*r*), -*** (SEM)	N/A	Population + employment density: + ** (*r*), nonsig (SEM)	N/A	Metropolitan area population, indicating connectivity: + ** (*r*), + *** (SEM)
Karmakar et al. [8]	US	County	Mar 25– Jul 29, 2020	Cross-sectional study	-***	-***	-***	+ ***	+ ***	Rate of public transport commuters: + ***
Ahmad et al. [9]	3135 US counties	County	As of Apr 21, 2020	Cross-sectional study	N/A	N/A	N/A	N/A	+ *	N/A
Kan et al. [10]	Hong Kong	Intra-city	As of Apr 14, 2020	Quartile analysis	N/A	N/A	+	-	N/A	Building density, building height, green spaces, public residential, open and recreation land: -; Private residential: +
Kwok et al. [11]	Hong Kong	Intra-city	As of Apr 30, 2020	Logistic regression, case–control, lasso regression	+ **	Nonsig	Male: -**, female: Nonsig	+ **	N/A	Summed building height: + ** (logistic); average street length: -** (logistic, case–control)
Karaye and Horney [12]	US	County	As of May 12, 2020	OLS, geographically weighted regression (GWR)	N/A	Overall SES: global result by OLS: + (*P* = 0.05)		N/A	Housing and transportation vulnerability: global result by OLS: -***, local variation by GWR: -1.10 – 1.53	
Ulimwengu and Kibonge [13]	US	County	May 1 – Dec 15, 2020	Spatial Durbin model	N/A	Overall SES: direct effect: -*** in May 16–Sept 2020, total effect: + *** in May 16–Sept 2020		Direct effect: usually + ***, total effect:-*** in Aug 16–Dec 2020	Housing and transportation vulnerability: direct effect: + ** ~ + ***, total effect: -*** in Oct – Dec 2020; Environment with high epidemic risk (prison, healthcare, and high-risk industries), total effect: + * ~ + *** in Aug–Dec 2020	

The symbols—and + mean negative and positive associations; *: *P* < 0.05, **: *P* < 0.01, ***: *P* < 0.001, and nonsig: not statistically significant; and N/A means that the characteristic was not investigated. Some of these studies investigated other characteristics that are not listed in the table

The wave-3 spread (Jul–Oct 2020) appeared to be more driven by the within-neighborhood transmission that was severer in densely populated neighborhoods, especially lower-SES ones. The wave-4 spread (Nov 2020–Feb 2021) appeared to be more driven by the cross-neighborhood transmission due to high activity level and connectivity. The spread patterns in both waves reconfirmed the vulnerability of lower-SES population against COVID-19 infections. High wave-3 local case rates were associated with high population density and connectivity (e.g., road density), low income and education level, crowded accommodations, small households, public residences for lower-income population, low vegetation coverage, and high density but low per-capita accessibility of POIs. Starting from the Dancing/Singing Cluster which heavily involved high-SES population, wave-4 local cases were less associated with SES or population density than wave-3 ones, but more concentrated in urban area and area with high connectivity and activity level. A high rate of wave-4 cases was most strongly and statistically significantly associated with road density, building density, and a low vegetation coverage (rule 13, 14, 27, 29, Table 2d). The associations for wave-4 cases excluding the Dancing/Singing Cluster became more similar to those for wave-3 cases, confirming that lower-SES population was still more vulnerable. The mid-to-low-income, high-density neighborhoods in main commercial and entertainment areas, with high activity level of lower-income residents as well as higher-income visitors from nearby neighborhoods, was worst hit.

In addition, higher-income population was associated with longer waiting periods between symptom onset and diagnosis, which might be attributed to their higher concern on the economic loss due to seeking medical advice related to COVID-19 and less anxiety for being infected. Since wealthier people normally have better medical resources, their longer waiting periods were likely due to longer delays in seeking medical advice, rather than slower diagnoses. Such delays might not be due to the privacy concern about health data, which were reported to be similar among people in different income levels, or even lower for higher-income people [33, 34]. Instead, higher-income people were reported to concern more about the economic impact of COVID-19 but less about being personally infected [35]. Therefore, wealthier people could have higher concern about the economic loss, such as being quarantined and unable to work, and higher confidence that they were well protected and did not really get COVID-19, which might have delayed their hospital or clinic visits.

Household size played different roles in household and community transmission. A high rate of wave-1&2 imported cases, including the cases directly infected by imported cases, was associated with a large average household size. This might be due to aggravated household transmission in larger households [36], since quarantine at home was allowed until November 2020. Also, high-SES families tended to have larger household sizes by including FDHs. Oppositely, a small average household size below 2.6–2.7 was associated with high local case rates, which might be linked with possibly more activities outside homes for one-person or two-person households (e.g., couples without children).

A high proportion of private residential LU was associated with high confirmed case rates in all waves. Since the reported locations of the cases were mostly their residences, these associations are expected and do not indicate a higher risk of infection in residential area. Rural area was generally associated with lower confirmed case rates, likely because of its much lower density and connectivity.

By comparing previous studies on the same area (e.g., the US) but different time periods, at the city/county scale, an urban characteristic may have variant associations with the COVID-19 spread in different waves of outbreaks [5, 6, 8, 9, 12, 13] (Table 3). This study shows that such temporally variant associations also exist at the intra-city scale, and, further, relates these associations with the intra-city distribution of specific population groups and activities. The study also reveals the intra-city local variations of COVID-19 transmissions in main urban areas with different SES levels and densities, satellite towns, and rural areas. These findings may provide references to investigate the local variations of the associations between urban characteristics and the COVID-19 spread at a coarser spatial scale (e.g., in different counties of a country) [12].

The study results have the following further implications for long-term pandemic control. First, the study reveals the joint and interactive contribution of density, connectivity, and functionality to COVID-19 spread within and across neighborhoods, especially in lower-SES neighborhoods. As a result, to relieve both overcrowdedness and overconcentration of facilities at the neighborhood scale is likely a critical task to improve the epidemic resilience in high-density cities. At the city scale, a significant causal effect of high population and employment density on the confirmed case distribution has been reported unable to be identified [7]. However, within a city, at least a high-density one, the particularly densely populated areas often indicate an overcrowded life with reduced quality. Residents in such areas, therefore, tend to be lower-income ones who do not afford a more spacious, higher-quality life. Overcrowdedness and low SES are linked with multiple conditions which could jointly or even synergistically contribute to extensive within-neighborhood transmission. These conditions include, for example, the difficulty to keep social distance in crowded accommodations and facilities, the tendency to spend more time outside less comfortable homes, the lower feasibility for manual labors to work from home, and the worse ventilation in old apartments.

Meanwhile, concentrated facilities and increased connectivity (e.g., transport hubs, easily accessible locations) can mutually attract and thus tend to co-locate, jointly leading to a high infection risk within the neighborhoods with concentrated facilities and high connectivity. Worse still, in many cities, high-SES people tend to reside in high-income neighborhoods, while the nearby commercial and entertainment areas they frequently visit often have lower SES. Those commercial and entertainment areas can suffer from an extreme risk of infection dually caused by intensive within-neighborhood transmissions due to high density and low SES, as well as intensive cross-neighborhood transmissions due to the high level of activities conducted by visitors of diverse SES. In the Hong Kong case, this was particularly reflected by the suffering of lower-SES TPUs in the major commercial and entertainment areas from the singing/dancing super-spread event. These TPUs also contained most entertainment venues involved in the super-spread of the Dancing/Singing Cluster (Fig. 3c). Their nearby high-SES TPUs contained almost no such entertainment venues but had even higher rate of confirmed cases in the Dancing/Singing Cluster (Additional file 1: Table S1), meaning that the cases in those TPUs should have visited the entertainment venues in the nearby lower-SES TPUs.

Second, higher-SES population, if infected, may have a higher potential to infect others and contribute to super-spread events than the lower-SES one. This brings the wealthy more obligation to conform anti-pandemic policies. In wave 3, higher-income neighborhoods appear less affected by the outbreak initiated in lower-income ones, which might be attributed to the self-segregation of the wealthy in higher-income neighborhoods [37]. Yet the super-spread event in wave 4 which heavily involved high-income population diffused to lower-income neighborhoods shortly afterwards. Such asymmetric effect may relate to that higher-SES people have higher mobility to more diverse area and higher accessibility to POIs outside their neighborhoods. Thus, on average, they contact more persons in a larger geographic scope, leading to a higher risk of cross-neighborhood transmission and super-spread. Lower-SES population, in contrast, tend to contact less diverse people in fewer places, leading to more localized transmission. High-SES population is also obliged to seek medical advice faster when showing COVID-19 symptoms, to avoid infecting others during longer waiting periods.

Third, by referring to the study results, pointed countermeasures to early increases of the cases may be developed to forestall recurrent outbreaks. Intra-city COVID-19 spread patterns, major transmission routes, and their interrelations with urban characteristics varied greatly in different waves of the pandemic. This study has identified such transmission routes and interrelationships for different sources of outbreaks: imported cases from developed countries (wave 1&2), localized transmission concentrating in lower-SES neighborhoods (wave 3), and super-spread events which considerably engage higher-SES population (wave 4). Facing an early increase of the cases, the study result can be used to pre-estimate the confirmed case distributions and transmission routes from the likely sources of such increase. Pointed countermeasures to specific neighborhoods or transmission routes could be further developed to prevent the increase from developing into a recurrent outbreak.

This study has multiple limitations. First, this fine-scale study has an advantage in revealing and reasoning intra-city associations between urban characteristics and COVID-19 transmission. Yet at such a fine scale, reported locations of the cases tended to be their residences and deviated from where they got infected. Such deviation limited the discovery of the infection risk for different activities outside the residences. Massive fine-scale human mobility data which is relatively representative for the whole population, such as smartphone tracking data from the carriers, may help identify people’s daily activity areas and lead to more accurate evaluations on the infection risks for different LUs. Also, while statistical tests have been performed in this and many other studies on the associations between the spatial patterns of various factors and COVID-19 spread, the statistical evaluation results need to be interpreted with caution. Parametric statistical tests generally assume the independence between observations, but spatial autocorrelation is prevalent in geographically distributed data, including the data for the factors investigated and the COVID-19 spread. The authors propose to further tackle this issue by exploring the use of non-parametric tests in future association studies, which may allow the data to be spatially autocorrelated. In addition, this study did not involve factors that were relatively homogeneous within a city at a certain time or had no available data at neighborhood level. These factors include, for example, environmental factors (e.g., relative humidity, temperature, and pollution) [38], non-pharmaceutical interventions (e.g., closure of schools and entertainment venues) [39], human behaviors (e.g., wearing face masks) [40], and COVID-19 testing rate [41]. In particular, the potential impact of seasonal climate and change in non-pharmaceutical interventions on the intra-city COVID-19 spread pattern is very much worth investigation. Effective investigations into these factors at an intra-city scale, again, require these factors to be properly measured for the venue where the cases exposed to the virus, instead of their reported residences.

## Conclusions

This study explores the intra-city associations in a high-density city between SES, density, functionality, and spread of COVID-19. Leveraging the advantage of DE-based ARM in studying optimized and complex associations, the associations were comparatively investigated for four waves of the pandemic in Hong Kong and for local and imported confirmed cases. Further analyzed based on these associations was how the urban characteristics might have jointly and interactively shaped the spatiotemporal patterns of COVID-19 cases, through different epidemic transmission routes within and across neighborhoods.

The study result showed that the first two waves of COVID-19 in Hong Kong (by May 2020) was mainly shaped by imported cases from developed countries. The high confirmed case rate was associated with high SES and related characteristics, such as mid-to-low population density and high accessibility to facilities. In the third (Jul–Oct 2020) and fourth (Nov 2020–Feb 2021) waves, densely populated and built neighborhoods, usually also lower-SES ones, were worse hit. The distribution of the wave-3 cases appeared more strongly shaped by the within-neighborhood transmission and lower SES. The patterns of wave-4 cases showed a stronger link to cross-neighborhood transmission and people’s activity level, likely due to the super-spread in dancing/singing venues. In particular, a diffusion was observed from the super-spread which considerably involved high-SES population to lower-SES neighborhoods and again the within-neighborhood transmission. Also, higher-SES population was found to be associated with mildly longer waiting periods.

The findings of this study provide potentially important references for precise control of COVID-19 at a neighborhood scale, as well as the pandemic-resilient design of compact cities. The usually co-locating overcrowededness and unfavored SES of residents can synergistically increase the vulnerability to epidemic of lower-SES neighborhoods and result in extensive within-neighborhood transmission. Lower-SES neighborhoods with concentrated facilities and non-residential activities can suffer from an extreme risk of infection dually caused by intensive within-neighborhood transmissions as well as intensive cross-neighborhood transmissions brought by visitors of diverse SES. To improve the epidemic resilience in high-density cities, it is, therefore, essential to relieve both overcrowdedness and overconcentration of facilities at the neighborhood scale. Also, higher-SES population is more obliged to conform anti-pandemic policies, due to their higher potential to participate extensive transmission and super-spread events. Facing early increases of the cases in a city, the study results may be used to develop pointed countermeasures against the likely sources of such increase and related specific neighborhoods or transmission routes, to forestall recurrent outbreaks.

## Supplementary Information

**Additional file 1.** **Table S1** (SES of the TPUs with different rates of cases in the Dancing/Singing Cluster) and **Table S2** (Full sets of resultant rules).

## Data Availability

All the dataset used in this study are publicly available and cited in this article. These include: Hong Kong COVID-19 confirmed cases: https://www.news.gov.hk/eng/categories/covid19/index.html, Hong Kong 2016 Population By-census: https://www.bycensus2016.gov.hk/en/bc-dp-tpu.html, Hong Kong 1:1000 and 1:5000 Digital Topographic Maps: https://www.landsd.gov.hk/mapping/en/digital_map/mapprod.htm, bus stop locations in Hong Kong: https://data.gov.hk/en-data/dataset/hk-td-tis_3-routes-and-fares-of-public-transport/resource/ad532643-0b31-4571-93e1-1fd9b1574aa1, Land Utilization in Hong Kong: https://www.pland.gov.hk/pland_en/info_serv/open_data/landu/index.html#!, TPU boundaries: http://www.dupad.hku.hk/cusup/hkugis/html/Data.html. The corresponding author could be contacted if the data needs to be accessed upon reasonable request.

## Abbreviations

ARM: Association rule mining
DE: Differential evolution
FDH: Foreign domestic helpers
GWR: Geographically weighted regression
OLS: Ordinary least square
RIM: Rule interestingness measure
SAC model: Spatial autoregressive combined model
SEM: Structural equation modeling
SES: Socioeconomic Status
TPU: Tertiary Planning Unit

## References

[1] Aschwanden C (2021). Five reasons why COVID herd immunity is probably impossible. Nature.

[2] Bian L, Gao Q, Gao F, Wang Q, He Q, Wu X, Mao Q, Xu M, Liang Z (2021). Impact of the Delta variant on vaccine efficacy and response strategies. Expert Rev Vaccines.

[3] Tang P, Hasan MR, Chemaitelly H, Yassine HM, Benslimane FM, Al Khatib HA, AlMukdad S, Coyle P, Ayoub HH, Al KanaaniAl Kuwari ZKE (2021). BNT162b2 and mRNA-1273 COVID-19 vaccine effectiveness against the SARS-CoV-2 Delta variant in Qatar. Nature Med.

[4] Yang C, Sha D, Liu Q, Li Y, Lan H, Guan WW, Hu T, Li Z, Zhang Z, Thompson JH, Wang Z (2020). Taking the pulse of COVID-19: a spatiotemporal perspective. Int J Digital Earth.

[5] Sun F, Matthews SA, Yang TC, Hu MH (2020). A spatial analysis of the COVID-19 period prevalence in US counties through June 28, 2020: where geography matters?. Ann Epidemiol.

[6] Zhang CH, Schwartz GG (2020). Spatial disparities in coronavirus incidence and mortality in the United States: an ecological analysis as of May 2020. J Rural Health.

[7] Hamidi S, Sabouri S, Ewing R (2020). Does density aggravate the COVID-19 pandemic? Early findings and lessons for planners. J Am Plann Assoc.

[8] Karmakar M, Lantz PM, Tipirneni R (2021). Association of social and demographic factors with COVID-19 incidence and death rates in the US. JAMA Netw Open.

[9] Ahmad K, Erqou S, Shah N, Nazir U, Morrison AR, Choudhary G, Wu WC (2020). Association of poor housing conditions with COVID-19 incidence and mortality across US counties. PLoS ONE.

[10] Kan Z, Kwan MP, Wong MS, Huang J, Liu D (2021). Identifying the space-time patterns of COVID-19 risk and their associations with different built environment features in Hong Kong. Sci Total Environ.

[11] Kwok CY, Wong MS, Chan KL, Kwan MP, Nichol JE, Liu CH, Wong JY, Wai AK, Chan LW, Xu Y, Li H (2021). Spatial analysis of the impact of urban geometry and socio-demographic characteristics on COVID-19, a study in Hong Kong. Sci Total Environ.

[12] Karaye IM, Horney JA (2020). The impact of social vulnerability on COVID-19 in the US: an analysis of spatially varying relationships. Am J Prev Med.

[13] Ulimwengu J, Kibonge A (2021). Spatial spillover and COVID-19 spread in the U.S.. BMC Public Health.

[14] Lakshmi Priyadarsini S, Suresh M (2020). Factors influencing the epidemiological characteristics of pandemic COVID 19: a TISM approach. Int J Healthcare Manage.

[15] Zhang A, Shi W (2020). Mining significant fuzzy association rules with differential evolution algorithm. Appl Soft Comput.

[16] Hong Kong Information Services Department. New.gov.hk – COVID-19. 2020. https://www.news.gov.hk/eng/categories/covid19/index.html (2020). Accessed 26 Mar 2021.

[17] Center of Health Protection, HKSAR Government. Latest situation of cases of COVID-19 (as of 24 May 2020). 2020. https://www.chp.gov.hk/files/pdf/local_situation_covid19_en_20200524.pdf. Accessed 26 Mar 2021.

[18] Siu GK, Lee LK, Leung KS, Leung JS, Ng TT, Chan CT, Tam KK, Lao HY, Wu AK, Yau MC, Lai YW (2020). Will a new clade of SARS-CoV-2 imported into the community spark a fourth wave of the COVID-19 outbreak in Hong Kong?. Emerg Microb Infect.

[19] To KK, Chan WM, Ip JD, Chu AW, Tam AR, Liu R, Wu AK, Lung KC, Tsang OT, Lau DP, To WK (2021). Unique clusters of severe acute respiratory syndrome coronavirus 2 causing a large coronavirus disease 2019 outbreak in Hong Kong. Clin Infect Dis.

[20] Center of Health Protection, HKSAR Government. Latest situation of cases of COVID-19 (as of 12 March 2021). 2021. https://www.chp.gov.hk/files/pdf/local_situation_covid19_en_20210312.pdf. Accessed 26 Mar 2021.

[21] Census and Statistics Department, HKSAR Government. District Profiles | 2016 Population By-census. 2016. https://www.bycensus2016.gov.hk/en/bc-dp-tpu.html. Accessed 20 Dec 2021.

[22] Surveying and Mapping Office, HKSAR Government. Maps and Services. 2016. https://www.landsd.gov.hk/mapping/en/digital_map/mapprod.htm. Accessed 20 Dec 2021.

[23] Hong Kong Transport Department. Coordination of bus stop location in Hong Kong. 2021. https://data.gov.hk/en-data/dataset/hk-td-tis_3-routes-and-fares-of-public-transport/resource/ad532643-0b31-4571-93e1-1fd9b1574aa1. Accessed 26 Mar 2021.

[24] Hong Kong Planning Department. Land Utilization in Hong Kong. 2021. https://www.pland.gov.hk/pland_en/info_serv/open_data/landu/index.html#!. Accessed 26 Mar 2021.

[25] HKU GIS Research Centre. Major Data Sources in Hong Kong: Planning Department - Digital boundary of Town Planning Unit (TPU) and Street Block (SB). 2016. http://www.dupad.hku.hk/cusup/hkugis/html/Data.html. Accessed 26 Mar 2021.

[26] Lenormand M, Bassolas A, Ramasco JJ (2016). Systematic comparison of trip distribution laws and models. J Transp Geogr.

[27] Yang H, Ke J, Ye J (2018). A universal distribution law of network detour ratios. Transp Res C.

[28] Telikani A, Gandomi AH, Shahbahrami A (2020). A survey of evolutionary computation for association rule mining. Inf Sci.

[29] Altaf W, Shahbaz M, Guergachi A (2017). Applications of association rule mining in health informatics: a survey. Artif Intell Rev.

[30] Piatetsky-Shapiro G (1991). Discovery, analysis, and presentation of strong rules. Knowl Discov Databases..

[31] Bayardo RJ, Agrawal R, Gunopulos D (2000). Constraint-based rule mining in large, dense databases. Data Min Knowl Disc.

[32] HKSAR Government. Compulsory testing notice gazetted (November 22, 24, 26, 29 and December 1 2021). 2021. https://www.info.gov.hk/gia/general/202011/22/P2020112200075.htm; https://www.info.gov.hk/gia/general/202011/24/P2020112400025.htm; https://www.info.gov.hk/gia/general/202011/26/P2020112600845.htm; https://www.info.gov.hk/gia/general/202011/29/P2020112900724.htm; https://www.info.gov.hk/gia/general/202012/01/P2020120100972.htm. Accessed 12 May 2021.

[33] Kaufman DJ, Murphy-Bollinger J, Scott J, Hudson KL (2009). Public opinion about the importance of privacy in biobank research. Am J Hum Genet.

[34] Ermakova T, Fabian B, Kelkel S, Wolff T, Zarnekow R (2015). Antecedents of health information privacy concerns. Procedia Comput Sci.

[35] McPhillips D. Coronavirus Survey: Worry About the Economy Is Highest Among the Wealthy. In: U.S. News & World Report. 2021 https://www.usnews.com/news/healthiest-communities/articles/2020-03-21/worry-about-coronavirus-and-economy-highest-among-the-wealthy. Accessed 14th May 2021.

[36] Jarvis CI, Van Zandvoort K, Gimma A, Prem K, Klepac P, Rubin GJ, Edmunds WJ (2020). Quantifying the impact of physical distance measures on the transmission of COVID-19 in the UK. BMC Med.

[37] Ng MK, Lau YT, Chen H, He S. Dual Land Regime, Income Inequalities and Multifaceted Socio-Economic and Spatial Segregation in Hong Kong. Urban Socio-Economic Segregation and Income Inequality. 2021;113.

[38] Fontal A, Bouma MJ, San-José A, López L, Pascual M, Rodó X (2021). Climatic signatures in the different COVID-19 pandemic waves across both hemispheres. Nat Comput Sci.

[39] Cowling BJ, Ali ST, Ng TW, Tsang TK, Li JC, Fong MW, Liao Q, Kwan MY, Lee SL, Chiu SS, Wu JT (2020). Impact assessment of non-pharmaceutical interventions against coronavirus disease 2019 and influenza in Hong Kong: an observational study. Lancet Public Health.

[40] Howard J, Huang A, Li Z, Tufekci Z, Zdimal V, van der Westhuizen HM, von Delft A, Price A, Fridman L, Tang LH, Tang V (2021). An evidence review of face masks against COVID-19. Proc Natl Acad Sci.

[41] Russell TW, Golding N, Hellewell J, Abbott S, Wright L, Pearson CA, van Zandvoort K, Jarvis CI, Gibbs H, Liu Y, Eggo RM (2020). Reconstructing the early global dynamics of under-ascertained COVID-19 cases and infections. BMC Med.

