# Supplementary material for: The fine-scale associations between socioeconomic status, density, functionality, and spread of COVID-19 within a high-density city
Source: BMC Infect Dis. 2022 Mar 21;22:274. doi: 10.1186/s12879-022-07274-w (PMC8936044; doi:10.1186/s12879-022-07274-w)
Supplement: Supplementary file 1 — Additional file 1. Table S1 (SES of the TPUs with different rates of cases in the Dancing/Singing Cluster) and Table S2 (Full sets of resultant rules). [file 12879_2022_7274_MOESM1_ESM.pdf]

The fine-scale associations between socioeconomic status, density, functionality, and spread of COVID-19 within a high-density city

Supplementary Information

Table S1. The average values of the median income, higher-education rate, and population density of the TPUs with different rates of the cases in the Dancing/Singing Cluster. The income and higher-education rate generally increased with the rate of the cases in the Dancing/Singing Cluster. The groups of TPUs with the highest and lowest rates of cases did not follow this pattern, since these two groups contained over 20 TPUs with fewer than 3,000 persons. These small-population TPUs could have either high or low incomes, and they would be in the group with the highest rate of the cases (>0.35‰) by containing only one case in the Dancing/Singing Cluster.

Rate of the cases in the Dancing/Singing Cluster (‰ out of total population)	No. of TPUs	Average med_income	Average prop_higher_edu	Average den_population
0	79	20457	0.219	12,723
(0, 0.1]	60	18238	0.21	50,131
(0.1, 0.2]	29	18081	0.207	36,481
(0.2, 0.35]	28	27434	0.308	36,139
>0.35	18	20944	0.247	33,710

Table S2. Full sets of resultant rules from the modified differential-evolution-based association rule mining algorithm DESigFAR. Rules take the form "antecedent → consequent". For example, rule 1 in Table S1a is "build_area_pp >75.706 (p55) &prop_industrial <0.001 (p47) → rate_local12 >0.112 (p81)", where p55 means the 55th percentile of rate_local12 >0.112 (p81) values for all TPU-level areal units in Hong Kong. The descriptions of variables in the rules are given in Table 1. Rules are shown together with the values of four RIMs: support (supp), confidence (conf), leverage (lev), and improvement (imp). P is the P value of each rule in Chi-square test described in the Methods.

(a) Rules for wave-1 local case rate; POI accessibility was used

No.	Antecedent	Consequent	Supp	Conf	Lev	Imp	*P*
**1**	build_area_pp >75.706 (p55) &prop_industrial <0.001 (p47)	rate_local12 >0.112 (p81)	24.18	0.50	14.71	0.18	2.89E-05
**2**	POI_pp_mall_mkt =4.008-16.323 (p46-86)	rate_local12 >0.103 (p79)	32.90	0.38	14.35	0.16	4.33E-09
**3**	prop_rural_set <0.008 (p56)	rate_local12 >0.038 (p60)	61.34	0.52	13.81	0.12	6.93E-05
**4**	POI_pp_telecom_elec =7.292-35.358 (p38-84)	rate_local12 >0.103 (p79)	33.88	0.35	13.47	0.14	3.13E-07
**5**	POI_pp_sports =10.367-68.635 (p38-87)	rate_local12 >0.106 (p80)	35.21	0.33	13.03	0.12	5.55E-06
**6**	prop_industrial <0.001 (p47)	rate_local12 >0.104 (p80)	33.96	0.34	12.94	0.13	3.96E-06
**7**	POI_pp_edu =10.533-29.153 (p52-86)	rate_local12 >0.103 (p79)	27.64	0.39	12.83	0.18	7.92E-08
**8**	POI_pp_transport =27.804-75.207 (p51-82)	rate_local12 >0.103 (p79)	26.40	0.40	12.38	0.19	1.88E-06
**9**	build_area_pp >76.990 (p56)	rate_local12 >0.116 (p81)	29.38	0.31	11.47	0.12	7.66E-06
**10**	prop_private_resid >0.101 (p66)	rate_local12 >0.046 (p63)	39.60	0.52	11.15	0.15	2.84E-06
**11**	prop_preprim_edu <0.081 (p20)	rate_local12 >0.115 (p81)	18.61	0.42	10.28	0.23	3.09E-06
**12**	LU_entropy <0.543 (p46)	rate_local12 >0.106 (p80)	29.65	0.30	9.50	0.10	6.5E-05
**13**	med_area_home >16.093 (p47)	rate_local12 >0.136 (p85)	26.44	0.23	8.43	0.07	6.08E-05
**14**	med_income >18518.024 (p62)	rate_local12 >0.136 (p85)	20.40	0.24	7.24	0.09	5.54E-05
**15**	prop_transport >0.335 (p87)	rate_local12 >0.086 (p76)	13.08	0.51	6.76	0.26	5.1E-05
**16**	gender_ratio >106.692 (p92)	rate_local12 >0.107 (p81)	9.88	0.57	6.33	0.36	1.48E-05
**17**	prop_business >0.111 (p92)	rate_local12 >0.108 (p81)	9.94	0.55	6.32	0.35	1.48E-05
**18**	prop_higher_edu >0.361 (p85)	rate_local12 >0.143 (p86)	10.73	0.33	5.98	0.18	3.34E-05
**19**	prop_gov_insti_faci =0.026-0.139 (p38-90) &POI_pp_mall_mkt >10.943 (p75)	rate_local12 >0.107 (p81)	9.64	0.49	5.65	0.23	7.82E-05
**20**	POI_pp_edu <19.348 (p74) &POI_pp_mall_mkt =9.973-30.551 (p70-94)	rate_local12 >0.090 (p77)	7.90	0.58	4.66	0.24	2.44E-05
**21**	POI_pp_sports <10.367 (p38)	rate_local12 =0.005-0.106 (p45-80)	58.75	0.73	31.44	0.39	0
**22**	prop_preprim_edu =0.081-0.137 (p20-85) &POI_pp_telecom_elec <13.825 (p56)	rate_local12 =0.005-0.106 (p45-80)	63.55	0.67	31.19	0.08	3.79E-05
**23**	POI_pp_telecom_elec <7.292 (p38)	rate_local12 =0.005-0.103 (p45-79)	58.38	0.72	31.14	0.38	0

**24**	prop_over65 =0.122-0.213 (p20-89) &POI_pp_sports <10.589 (p38)	rate_local12 =0.005-0.116 (p45-81)	53.21	0.84	30.66	0.10	4.84E-05
**25**	den_public_trans =4.269-90.965 (p28-91) &POI_pp_telecom_elec <14.517 (p58)	rate_local12 =0.005-0.117 (p45-82)	64.58	0.69	30.63	0.09	7.26E-06
**26**	gender_ratio =87.668-97.836 (p11-77) &POI_pp_transport <36.170 (p58)	rate_local12 =0.006-0.104 (p45-80)	63.46	0.65	30.57	0.09	7.32E-05
**27**	den_road >5.702 (p27) &POI_pp_telecom_elec <13.365 (p54)	rate_local12 =0.005-0.108 (p45-81)	63.10	0.67	30.53	0.07	2.39E-05
**28**	gender_ratio =87.593-97.312 (p11-76) &POI_pp_telecom_elec <13.575 (p55)	rate_local12 =0.005-0.100 (p45-78)	60.69	0.66	30.51	0.08	5.19E-05
**29**	gender_ratio =87.457-99.480 (p11-79) &med_area_home <19.521 (p61) &POI_pp_sports <28.415 (p66)	rate_local12 =0.005-0.104 (p45-80)	60.19	0.68	30.49	0.07	3.33E-05
**30**	prop_preprim_edu =0.081-0.135 (p21-85) &POI_pp_transport <36.474 (p58)	rate_local12 =0.005-0.103 (p45-79)	62.78	0.65	30.46	0.09	1.66E-05
**31**	med_area_home <17.998 (p55) &den_public_trans =3.985-90.248 (p27-91) &POI_pp_sports <26.288 (p64)	rate_local12 =0.006-0.114 (p45-81)	57.88	0.75	30.44	0.07	1.13E-05
**32**	gender_ratio =87.711-98.931 (p11-79) &med_area_home <19.205 (p61) &POI_pp_mall_mkt <8.837 (p66)	rate_local12 =0.005-0.104 (p45-80)	58.69	0.70	30.37	0.08	1.24E-05
**33**	POI_pp_transport <16.151 (p36)	rate_local12 =0.005-0.103 (p45-79)	56.03	0.73	30.33	0.39	0
**34**	prop_over65 =0.128-0.214 (p24-89) &POI_pp_telecom_elec <8.009 (p40)	rate_local12 =0.005-0.117 (p45-82)	53.36	0.83	30.32	0.11	6.79E-05
**35**	gender_ratio =87.481-99.842 (p11-82) &med_area_home <19.205 (p61) &POI_pp_edu <15.586 (p69)	rate_local12 =0.005-0.111 (p45-81)	60.61	0.70	30.28	0.07	1.54E-05
**36**	gender_ratio =87.457-99.480 (p11-79) &med_area_home <19.520 (p61) &POI_pp_transport <48.280 (p69)	rate_local12 =0.006-0.111 (p45-81)	61.07	0.69	30.14	0.07	3.98E-05
**37**	prop_over65 =0.128-0.214 (p24-89) &POI_pp_transport <17.780 (p39)	rate_local12 =0.005-0.114 (p45-81)	52.11	0.84	30.11	0.12	3.75E-05
**38**	den_public_trans =6.888-92.606 (p35-91) &POI_pp_transport <34.910 (p57)	rate_local12 =0.006-0.114 (p45-81)	60.33	0.71	30.05	0.11	5.22E-06
**39**	den_road >5.586 (p27) &POI_pp_transport <31.516 (p53)	rate_local12 =0.005-0.115 (p45-81)	63.38	0.68	30.02	0.07	5.65E-05

**40**	med_area_home <16.183 (p48) &den_public_trans =4.501-90.772 (p29-91) &POI_pp_edu <18.658 (p73)	rate_local12 =0.005-0.117 (p45-82)	56.19	0.77	30.00	0.08	1.32E-05
**41**	prop_preprim_edu =0.081-0.138 (p20-86) &POI_pp_sports <21.150 (p56)	rate_local12 =0.005-0.103 (p45-79)	61.32	0.65	29.96	0.08	2.66E-07
**42**	prop_gov_insti_faci =0.013-0.144 (p29-91) &POI_pp_transport <33.050 (p55)	rate_local12 =0.005-0.110 (p45-81)	61.32	0.68	29.94	0.09	8.29E-05
**43**	med_area_home <16.183 (p48) &den_public_trans =4.501-90.772 (p29-91) &POI_pp_transport <41.284 (p63)	rate_local12 =0.005-0.117 (p45-82)	55.90	0.77	29.90	0.07	2.7E-06
**44**	gender_ratio =87.457-99.480 (p11-79) &med_area_home <19.456 (p61) &POI_pp_telecom_elec <21.356 (p71)	rate_local12 =0.006-0.103 (p45-79)	59.94	0.66	29.76	0.06	4.24E-05
**45**	den_public_trans =4.218-88.884 (p28-91) &POI_pp_mall_mkt <8.288 (p64)	rate_local12 =0.006-0.124 (p45-82)	66.19	0.68	29.71	0.13	7.13E-08
**46**	med_area_home <17.998 (p55) &POI_pp_mall_mkt <8.471 (p65)	rate_local12 =0.005-0.119 (p45-82)	63.15	0.69	29.68	0.13	1.03E-07
**47**	prop_over65 =0.117-0.212 (p16-89) &build_area_pp <88.565 (p62) &POI_pp_transport <38.267 (p60)	rate_local12 =0.005-0.106 (p45-80)	55.80	0.72	29.64	0.09	2.31E-05
**48**	med_area_home <15.583 (p45) &POI_pp_telecom_elec <13.365 (p54)	rate_local12 =0.004-0.112 (p45-81)	56.13	0.74	29.59	0.13	2.89E-05
**49**	med_area_home <15.505 (p45) &POI_pp_transport <35.608 (p57)	rate_local12 =0.005-0.114 (p45-81)	56.94	0.74	29.58	0.13	1.32E-05
**50**	prop_preprim_edu =0.081-0.135 (p21-85) &build_area_pp <107.300 (p71) &POI_pp_mall_mkt <8.948 (p66)	rate_local12 =0.006-0.114 (p45-81)	61.03	0.69	29.55	0.07	3.45E-05
**51**	den_public_trans =6.736-95.296 (p34-92) &POI_pp_sports <23.417 (p59)	rate_local12 =0.005-0.110 (p45-81)	60.57	0.68	29.51	0.12	3.46E-05
**52**	prop_preprim_edu =0.080-0.138 (p19-85) &POI_pp_mall_mkt <4.227 (p47)	rate_local12 =0.005-0.106 (p45-80)	57.31	0.70	29.47	0.07	8.32E-05
**53**	prop_preprim_edu =0.081-0.137 (p20-85) &med_area_home <17.418 (p52) &POI_pp_sports <26.094 (p64)	rate_local12 =0.004-0.105 (p45-80)	54.24	0.74	29.46	0.08	9E-06
**54**	gender_ratio =87.605-98.187 (p11-78) &POI_pp_sports <23.667 (p59)	rate_local12 =0.005-0.103 (p45-79)	63.70	0.62	29.44	0.08	5.47E-05

**55**	med_area_home <15.461 (p45) &POI_pp_sports <22.789 (p58)	rate_local12 =0.005-0.112 (p45-81)	55.85	0.74	29.44	0.14	1.16E-06
**56**	den_road >5.296 (p26) &POI_pp_mall_mkt <4.041 (p46)	rate_local12 =0.005-0.102 (p45-78)	55.22	0.71	29.40	0.08	2.13E-05
**57**	med_area_home <15.505 (p45) &POI_pp_edu <15.002 (p67)	rate_local12 =0.005-0.116 (p45-82)	57.07	0.74	29.28	0.13	2.8E-07
**58**	prop_preprim_edu =0.081-0.137 (p20-85) &med_area_home <18.212 (p55) &POI_pp_mall_mkt <8.545 (p65)	rate_local12 =0.006-0.098 (p45-77)	53.79	0.71	29.26	0.08	7.14E-06
**59**	prop_preprim_edu =0.081-0.137 (p20-85) &build_area_pp <102.463 (p68) &POI_pp_edu <14.107 (p64)	rate_local12 =0.005-0.100 (p45-78)	56.92	0.68	29.22	0.08	4.94E-05
**60**	prop_over65 =0.117-0.212 (p16-89) &build_area_pp <88.565 (p62) &POI_pp_sports <18.025 (p51)	rate_local12 =0.006-0.115 (p45-81)	54.86	0.76	29.20	0.08	5.98E-05
**61**	gender_ratio =87.587-97.703 (p11-77) &prop_gov_insti_faci =0.016-0.142 (p31-91) &POI_pp_edu <15.454 (p69)	rate_local12 =0.005-0.113 (p45-81)	57.48	0.72	29.15	0.08	4.55E-05
**62**	prop_gov_insti_faci =0.016-0.146 (p31-91) &POI_pp_edu <13.036 (p59)	rate_local12 =0.005-0.113 (p45-81)	61.29	0.67	29.13	0.12	4.3E-06
**63**	gender_ratio =87.162-99.098 (p11-79) &den_road >5.681 (p27) &POI_pp_mall_mkt <9.900 (p70)	rate_local12 =0.005-0.100 (p45-78)	59.79	0.64	29.01	0.08	1.68E-05
**64**	den_road >5.813 (p28) &POI_pp_sports <24.250 (p62)	rate_local12 =0.005-0.109 (p45-81)	64.22	0.63	28.99	0.08	3.06E-06
**65**	prop_over65 =0.120-0.211 (p19-89) &prop_preprim_edu =0.082-0.136 (p21-85) &POI_pp_transport <38.261 (p60)	rate_local12 =0.005-0.110 (p45-81)	56.06	0.72	28.99	0.07	5.45E-05
**66**	build_area_pp <62.130 (p50) &POI_pp_transport <29.120 (p51)	rate_local12 =0.005-0.111 (p45-81)	56.65	0.71	28.93	0.10	6.4E-05
**67**	gender_ratio =87.265-98.353 (p11-78) &POI_pp_mall_mkt <4.554 (p48)	rate_local12 =0.005-0.094 (p45-77)	55.91	0.66	28.92	0.08	3.24E-05
**68**	den_road >5.911 (p28) &build_area_pp <87.159 (p62) &POI_pp_edu <14.977 (p67)	rate_local12 =0.005-0.114 (p45-81)	58.08	0.70	28.82	0.08	4.41E-05
**69**	den_road >5.584 (p27) &POI_pp_edu <12.877 (p59)	rate_local12 =0.005-0.106 (p45-81)	62.14	0.64	28.80	0.09	5.15E-07

**70**	prop_transport >0.055 (p33) &POI_pp_transport <33.033 (p55)	rate_local12 =0.005-0.109 (p45-81)	60.89	0.66	28.79	0.07	8.29E-05
**71**	prop_over65 =0.123-0.208 (p21-87) &build_area_pp <85.101 (p59) &POI_pp_telecom_elec <11.917 (p51)	rate_local12 =0.005-0.116 (p45-81)	52.25	0.80	28.77	0.08	4.46E-05
**72**	gender_ratio =87.824-99.340 (p11-79) &den_road >5.702 (p27) &POI_pp_transport <48.852 (p70)	rate_local12 =0.006-0.108 (p45-81)	61.36	0.65	28.76	0.06	1.99E-05
**73**	prop_over65 =0.117-0.212 (p16-89) &gender_ratio =87.732-98.813 (p11-78) &POI_pp_edu <14.968 (p67)	rate_local12 =0.005-0.108 (p45-81)	58.89	0.67	28.72	0.08	2.12E-05
**74**	prop_gov_insti_faci =0.026-0.139 (p38-90) &POI_pp_telecom_elec <13.575 (p55)	rate_local12 =0.005-0.124 (p45-82)	58.54	0.73	28.66	0.10	7.59E-05
**75**	gender_ratio =87.182-97.302 (p11-76) &prop_gov_insti_faci =0.016-0.142 (p31-91) &POI_pp_mall_mkt <10.308 (p72)	rate_local12 =0.006-0.102 (p45-79)	55.18	0.69	28.61	0.09	5.14E-05
**76**	prop_preprim_edu =0.081-0.138 (p20-86) &prop_gov_insti_faci =0.019-0.133 (p32-90) &POI_pp_mall_mkt <7.643 (p63)	rate_local12 =0.006-0.099 (p45-78)	52.20	0.72	28.58	0.07	4.18E-05
**77**	prop_preprim_edu =0.081-0.138 (p20-85) &build_area_pp <106.529 (p71) &POI_pp_telecom_elec <19.473 (p67)	rate_local12 =0.005-0.123 (p45-82)	62.36	0.68	28.57	0.06	7.19E-05
**78**	prop_gov_insti_faci =0.025-0.148 (p36-92) &POI_pp_sports <24.093 (p62)	rate_local12 =0.005-0.117 (p45-82)	59.66	0.69	28.56	0.12	2.57E-05
**79**	prop_preprim_edu =0.081-0.137 (p20-85) &med_area_home <16.988 (p52) &POI_pp_edu <14.107 (p64)	rate_local12 =0.005-0.104 (p45-80)	52.03	0.74	28.53	0.07	1.38E-05
**80**	den_public_trans =7.565-92.691 (p36-91) &build_area_pp <92.881 (p63) &POI_pp_edu <15.881 (p69)	rate_local12 =0.005-0.112 (p45-81)	55.19	0.72	28.48	0.08	3E-05
**81**	gender_ratio =87.683-99.716 (p11-81) &prop_preprim_edu =0.082-0.138 (p21-86) &POI_pp_telecom_elec <21.311 (p71)	rate_local12 =0.005-0.103 (p45-79)	58.68	0.65	28.46	0.07	1.97E-05
**82**	den_population >14401.549 (p46) &POI_pp_transport <34.255 (p57)	rate_local12 =0.005-0.115 (p45-81)	58.32	0.70	28.45	0.10	3.14E-05

**83**	prop_over65 =0.110-0.204 (p11-84) &med_area_home <19.802 (p65) &POI_pp_telecom_elec <21.288 (p71)	rate_local12 =0.003-0.144 (p45-86)	62.60	0.74	28.40	0.09	1.01E-05
**84**	gender_ratio =87.457-99.480 (p11-79) &prop_gov_insti_faci =0.015-0.133 (p31-90) &POI_pp_transport <48.280 (p69)	rate_local12 =0.006-0.106 (p45-80)	56.25	0.68	28.36	0.08	6.88E-05
**85**	gender_ratio =87.077-98.124 (p11-78) &POI_pp_edu <12.877 (p59)	rate_local12 =0.006-0.100 (p45-78)	61.01	0.62	28.33	0.08	2.24E-05
**86**	den_population >14284.410 (p45) &POI_pp_telecom_elec <12.938 (p53)	rate_local12 =0.005-0.114 (p45-81)	57.05	0.70	28.31	0.09	5.8E-05
**87**	prop_over65 =0.117-0.212 (p16-89) &gender_ratio =87.732-98.813 (p11-78) &POI_pp_transport <50.942 (p71)	rate_local12 =0.005-0.108 (p45-81)	59.50	0.66	28.29	0.08	3.28E-05
**88**	prop_higher_edu <0.334 (p79) &prop_open_recreation =0.017-0.146 (p36-89) &POI_pp_transport <31.516 (p53)	rate_local12 =0.005-0.120 (p45-82)	52.40	0.79	28.23	0.07	2.53E-06
**89**	prop_over65 =0.116-0.211 (p16-89) &med_area_home <20.542 (p68) &POI_pp_transport <57.981 (p76)	rate_local12 =0.007-0.149 (p45-87)	65.83	0.71	28.21	0.09	1.3E-05
**90**	prop_higher_edu <0.310 (p76) &prop_open_recreation =0.019-0.159 (p36-90) &POI_pp_telecom_elec <12.094 (p51)	rate_local12 =0.006-0.119 (p45-82)	50.97	0.81	28.20	0.09	1.55E-05
**91**	prop_over65 =0.129-0.215 (p26-91) &den_road >5.176 (p25) &POI_pp_telecom_elec <15.046 (p58)	rate_local12 =0.006-0.119 (p45-82)	54.47	0.75	28.20	0.06	7.52E-05
**92**	gender_ratio =86.809-98.970 (p11-79) &den_road >5.656 (p27) &POI_pp_edu <18.524 (p72)	rate_local12 =0.005-0.106 (p45-80)	61.01	0.63	28.16	0.07	4.02E-05
**93**	prop_over65 =0.117-0.212 (p16-89) &build_area_pp <89.794 (p62) &POI_pp_mall_mkt <6.720 (p57)	rate_local12 =0.005-0.120 (p45-82)	55.87	0.73	28.07	0.08	5.62E-05
**94**	gender_ratio =87.755-99.437 (p11-79) &den_road >5.702 (p27) &POI_pp_telecom_elec <22.112 (p72)	rate_local12 =0.006-0.107 (p45-81)	61.87	0.63	28.06	0.06	2.37E-05
**95**	prop_over65 =0.106-0.205 (p11-85) &prop_preprim_edu =0.081-0.137 (p20-85) &POI_pp_telecom_elec <21.288 (p71)	rate_local12 =0.006-0.117 (p45-82)	60.67	0.67	28.03	0.06	6.15E-05

**96**	gender_ratio =87.588-98.254 (p11-78) &build_area_pp <88.754 (p62) &POI_pp_transport <57.174 (p76)	rate_local12 =0.005-0.110 (p45-81)	59.02	0.66	28.03	0.08	5.26E-05
**97**	prop_higher_edu <0.320 (p79) &prop_open_recreation =0.017-0.149 (p36-89) &POI_pp_sports <22.889 (p58)	rate_local12 =0.005-0.117 (p45-82)	52.10	0.78	28.02	0.09	1.77E-05
**98**	prop_over65 =0.123-0.211 (p21-89) &med_area_home <20.826 (p70) &POI_pp_mall_mkt <4.366 (p47)	rate_local12 =0.006-0.113 (p45-81)	51.01	0.78	28.01	0.06	7.8E-05
**99**	den_population >14851.623 (p46) &POI_pp_sports <22.924 (p58)	rate_local12 =0.006-0.117 (p45-82)	58.15	0.69	27.95	0.11	2.34E-05
**100**	gender_ratio =87.439-100.096 (p11-83) &prop_preprim_edu =0.081-0.135 (p21-85) &build_area_pp <107.300 (p71)	rate_local12 =0.005-0.103 (p45-79)	57.37	0.65	27.87	0.09	5.93E-06
**101**	prop_over65 =0.120-0.212 (p19-89) &den_public_trans =9.671-95.636 (p38-92) &POI_pp_transport <37.816 (p60)	rate_local12 =0.005-0.119 (p45-82)	51.10	0.80	27.85	0.10	7.97E-06
**102**	build_area_pp <62.130 (p50) &POI_pp_mall_mkt <6.541 (p57)	rate_local12 =0.006-0.111 (p45-81)	56.45	0.69	27.85	0.13	1E-05
**103**	gender_ratio =87.394-100.433 (p11-83) &prop_preprim_edu =0.081-0.135 (p21-85) &med_area_home <17.144 (p52)	rate_local12 =0.005-0.103 (p45-79)	51.85	0.72	27.83	0.08	3.06E-05
**104**	den_population >15872.761 (p47) &POI_pp_mall_mkt <7.824 (p63)	rate_local12 =0.005-0.115 (p45-81)	56.86	0.70	27.77	0.16	3.06E-08
**105**	den_public_trans =6.736-95.296 (p34-92) &POI_pp_edu <14.618 (p67)	rate_local12 =0.005-0.117 (p45-82)	61.05	0.66	27.74	0.13	9.52E-06
**106**	prop_transport >0.076 (p36) &POI_pp_mall_mkt <7.469 (p61)	rate_local12 =0.005-0.104 (p45-80)	57.16	0.65	27.73	0.13	1.36E-05
**107**	gender_ratio =87.588-98.254 (p11-78) &build_area_pp <88.754 (p62) &POI_pp_edu <20.320 (p76)	rate_local12 =0.005-0.111 (p45-81)	59.32	0.66	27.70	0.07	7.17E-05
**108**	prop_over65 =0.126-0.212 (p23-89) &build_area_pp <89.825 (p62) &POI_pp_edu <14.949 (p67)	rate_local12 =0.005-0.120 (p45-82)	54.37	0.74	27.68	0.11	5.14E-05
**109**	prop_over65 =0.117-0.212 (p16-89) &den_public_trans =9.671-95.636 (p38-92) &POI_pp_sports <25.232 (p62)	rate_local12 =0.005-0.113 (p45-81)	51.16	0.77	27.63	0.09	1.29E-05

**110**	prop_over65 =0.129-0.215 (p26-91) &den_road >5.180 (p25) &POI_pp_transport <39.159 (p61)	rate_local12 =0.005-0.120 (p45-82)	54.90	0.73	27.63	0.06	6.62E-05
**111**	prop_over65 =0.129-0.215 (p26-91) &den_road >5.180 (p25) &POI_pp_mall_mkt <3.972 (p46)	rate_local12 =0.005-0.112 (p45-81)	47.56	0.84	27.63	0.09	1.95E-06
**112**	build_area_pp <57.444 (p46) &POI_pp_sports <22.558 (p57)	rate_local12 =0.005-0.114 (p45-81)	56.13	0.70	27.62	0.12	8.22E-05
**113**	prop_transport >0.068 (p35) &POI_pp_sports <26.365 (p64)	rate_local12 =0.005-0.108 (p45-81)	61.46	0.62	27.53	0.10	8.25E-05
**114**	build_area_pp <62.130 (p50) &POI_pp_edu <12.894 (p59)	rate_local12 =0.005-0.111 (p45-81)	56.65	0.68	27.51	0.13	4.81E-05
**115**	prop_preprim_edu =0.081-0.137 (p20-85) &POI_pp_edu <14.107 (p64)	rate_local12 =0.005-0.104 (p45-80)	61.92	0.60	27.46	0.09	2.85E-06
**116**	prop_transport >0.071 (p36) &POI_pp_edu <12.877 (p59)	rate_local12 =0.005-0.100 (p45-78)	56.45	0.64	27.43	0.11	5.73E-05
**117**	den_population >15872.761 (p47) &POI_pp_edu <13.254 (p60)	rate_local12 =0.005-0.116 (p45-81)	56.59	0.70	27.43	0.14	2.09E-07
**118**	prop_over65 =0.129-0.215 (p26-91) &den_road >5.180 (p25) &POI_pp_sports <23.099 (p59)	rate_local12 =0.005-0.113 (p45-81)	52.72	0.74	27.43	0.07	5.13E-05
**119**	prop_over65 =0.129-0.212 (p25-89) &POI_pp_mall_mkt <4.554 (p48)	rate_local12 =0.006-0.117 (p45-82)	53.67	0.74	27.41	0.11	5.96E-05
**120**	gender_ratio =87.683-99.716 (p11-81) &prop_preprim_edu =0.082-0.138 (p21-86) &POI_pp_mall_mkt <11.835 (p78)	rate_local12 =0.005-0.103 (p45-79)	59.22	0.62	27.39	0.06	4.47E-05
**121**	gender_ratio =87.425-98.724 (p11-78) &build_area_pp <95.076 (p64) &POI_pp_telecom_elec <25.420 (p76)	rate_local12 =0.004-0.102 (p45-79)	59.20	0.62	27.37	0.06	7.36E-05
**122**	gender_ratio =87.607-99.246 (p11-79) &den_public_trans =7.177-94.877 (p35-92) &POI_pp_telecom_elec <22.605 (p72)	rate_local12 =0.005-0.104 (p45-80)	56.10	0.66	27.35	0.07	7.59E-05
**123**	gender_ratio =87.169-100.269 (p11-83) &build_area_pp <93.213 (p63) &POI_pp_sports <37.539 (p75)	rate_local12 =0.005-0.101 (p45-78)	59.52	0.61	27.33	0.06	7.88E-05
**124**	prop_gov_insti_faci =0.026-0.139 (p38-90) &POI_pp_mall_mkt <5.609 (p53)	rate_local12 =0.005-0.107 (p45-81)	52.35	0.72	27.27	0.14	6.67E-06

**125**	prop_over65 =0.125-0.214 (p22-89) &den_population >15872.761 (p47) &POI_pp_sports <24.142 (p62)	rate_local12 =0.005-0.124 (p45-82)	51.50	0.79	27.20	0.09	7.8E-05
**126**	POI_pp_edu <6.132 (p40)	rate_local12 =0.005-0.103 (p45-79)	54.68	0.67	27.19	0.33	0
**127**	prop_over65 =0.129-0.212 (p25-89) &den_population >15872.761 (p47) &POI_pp_telecom_elec <14.160 (p57)	rate_local12 =0.005-0.125 (p45-82)	50.48	0.81	27.16	0.09	2.96E-05
**128**	prop_over65 =0.134-0.216 (p29-91) &prop_business =0.001-0.111 (p29-92) &POI_pp_telecom_elec <16.509 (p60)	rate_local12 =0.006-0.111 (p45-81)	50.93	0.75	27.15	0.09	6.33E-05
**129**	gender_ratio =87.585-98.104 (p11-78) &med_area_home <19.264 (p61)	rate_local12 =0.006-0.102 (p45-79)	58.37	0.62	27.07	0.14	1.47E-08
**130**	gender_ratio =87.251-100.572 (p11-83) &med_area_home <20.980 (p73) &den_public_trans =5.241-90.538 (p32-91)	rate_local12 =0.006-0.103 (p45-79)	54.66	0.66	27.04	0.08	2.63E-05
**131**	gender_ratio =87.835-98.216 (p11-78) &den_public_trans =4.834-84.999 (p30-91) &POI_pp_mall_mkt <11.542 (p76)	rate_local12 =0.004-0.105 (p45-80)	56.31	0.65	27.03	0.08	6.94E-05
**132**	prop_higher_edu <0.310 (p76) &prop_open_recreation =0.019-0.159 (p36-90) &POI_pp_edu <15.665 (p69)	rate_local12 =0.006-0.120 (p45-82)	52.33	0.75	26.99	0.10	4.9E-05
**133**	prop_open_recreation =0.017-0.146 (p36-89) &POI_pp_transport <31.516 (p53)	rate_local12 =0.005-0.120 (p45-82)	54.63	0.72	26.98	0.10	7.42E-05
**134**	prop_higher_edu <0.315 (p78) &prop_open_recreation =0.016-0.154 (p36-90) &POI_pp_mall_mkt <9.453 (p67)	rate_local12 =0.006-0.132 (p45-83)	54.58	0.76	26.98	0.08	4.95E-05
**135**	prop_over65 =0.117-0.212 (p16-89) &POI_pp_edu <13.746 (p61)	rate_local12 =0.005-0.125 (p45-82)	65.06	0.64	26.97	0.08	4.74E-05
**136**	prop_open_recreation =0.017-0.143 (p36-88) &POI_pp_telecom_elec <13.739 (p55)	rate_local12 =0.006-0.131 (p45-83)	56.22	0.74	26.93	0.11	3.7E-05
**137**	prop_over65 =0.121-0.212 (p20-89) &prop_preprim_edu =0.080-0.138 (p19-86) &build_area_pp <102.210 (p67)	rate_local12 =0.005-0.115 (p45-81)	55.77	0.69	26.88	0.11	6.11E-06
**138**	prop_over65 =0.117-0.212 (p16-89) &med_area_home <19.775 (p65) &build_area_pp <88.565 (p62)	rate_local12 =0.005-0.131 (p45-83)	56.88	0.72	26.85	0.10	5.8E-05

**139**	prop_over65 =0.117-0.212 (p16-89) &med_area_home <15.226 (p45)	rate_local12 =0.005-0.125 (p45-82)	54.08	0.74	26.83	0.10	5.44E-06
**140**	prop_open_recreation =0.017-0.154 (p36-90) &POI_pp_edu <12.877 (p59)	rate_local12 =0.005-0.121 (p45-82)	55.40	0.71	26.83	0.14	1.99E-05
**141**	med_income <19678.979 (p65) &prop_open_recreation =0.014-0.146 (p34-89) &POI_pp_telecom_elec <11.657 (p50)	rate_local12 =0.005-0.122 (p45-82)	49.37	0.81	26.81	0.08	1.21E-05
**142**	prop_over65 =0.117-0.212 (p16-89) &den_public_trans =9.671-95.636 (p38-92) &POI_pp_edu <13.746 (p61)	rate_local12 =0.005-0.114 (p45-81)	50.28	0.76	26.80	0.09	2.28E-05
**143**	prop_over65 =0.117-0.213 (p16-89) &gender_ratio =87.588-98.254 (p11-78) &POI_pp_sports <38.840 (p77)	rate_local12 =0.006-0.110 (p45-81)	59.26	0.63	26.75	0.08	7.47E-05
**144**	prop_preprim_edu =0.081-0.136 (p21-85) &den_road =5.778-21.939 (p28-86) &POI_pp_mall_mkt <8.083 (p64)	rate_local12 =0.006-0.098 (p45-78)	49.07	0.71	26.61	0.09	1.65E-05
**145**	prop_over65 =0.129-0.215 (p26-91) &den_population >16064.342 (p48) &POI_pp_edu <14.779 (p67)	rate_local12 =0.005-0.125 (p45-82)	51.17	0.78	26.61	0.09	3.43E-05
**146**	den_public_trans =7.631-93.088 (p36-92) &prop_gov_insti_faci =0.015-0.131 (p30-89) &POI_pp_edu <19.826 (p75)	rate_local12 =0.006-0.109 (p45-81)	53.20	0.69	26.49	0.08	8.29E-05
**147**	gender_ratio =87.008-100.319 (p11-83) &med_area_home <19.205 (p61) &prop_gov_insti_faci =0.008-0.127 (p21-88)	rate_local12 =0.005-0.096 (p45-77)	52.45	0.65	26.48	0.06	1.66E-05
**148**	prop_over65 =0.123-0.216 (p21-91) &den_public_trans =7.206-96.133 (p35-92) &POI_pp_mall_mkt <4.821 (p49)	rate_local12 =0.006-0.118 (p45-82)	46.98	0.83	26.47	0.10	7.62E-05
**149**	gender_ratio =87.587-97.703 (p11-77) &prop_preprim_edu =0.081-0.138 (p20-85) &prop_gov_insti_faci =0.016-0.142 (p31-91)	rate_local12 =0.005-0.103 (p45-79)	50.80	0.70	26.47	0.10	8.34E-06
**150**	prop_over65 =0.106-0.205 (p11-85) &gender_ratio =87.499-98.661 (p11-78) &prop_preprim_edu =0.081-0.137 (p20-85)	rate_local12 =0.005-0.106 (p45-80)	55.44	0.65	26.46	0.08	4.94E-05
**151**	build_area_pp <90.950 (p62) &prop_gov_insti_faci =0.009-0.141 (p21-91) &POI_pp_transport <59.920 (p78)	rate_local12 =0.005-0.108 (p45-81)	56.83	0.64	26.45	0.08	5.49E-05

**152**	prop_over65 =0.137-0.211 (p31-89) &prop_preprim_edu =0.081-0.138 (p20-86) &POI_pp_sports <21.150 (p56)	rate_local12 =0.005-0.104 (p45-80)	47.57	0.75	26.34	0.08	6.95E-05
**153**	build_area_pp <62.119 (p50) &POI_pp_telecom_elec <20.153 (p68)	rate_local12 =0.005-0.119 (p45-82)	60.20	0.64	26.25	0.08	7.66E-05
**154**	prop_open_recreation =0.017-0.143 (p36-88) &POI_pp_mall_mkt <8.250 (p64)	rate_local12 =0.005-0.121 (p45-82)	55.14	0.70	26.23	0.16	4.4E-07
**155**	gender_ratio =87.963-98.463 (p13-78) &den_population >15872.761 (p47) &POI_pp_transport <51.668 (p72)	rate_local12 =0.005-0.105 (p45-80)	52.72	0.67	26.22	0.06	8.13E-05
**156**	prop_open_recreation =0.016-0.143 (p36-89) &POI_pp_sports <23.662 (p59)	rate_local12 =0.006-0.124 (p45-82)	55.79	0.70	26.20	0.12	5.75E-05
**157**	gender_ratio =87.620-99.262 (p11-79) &den_public_trans =8.345-95.802 (p37-92) &build_area_pp <112.199 (p72)	rate_local12 =0.005-0.108 (p45-81)	53.53	0.67	26.15	0.07	5.8E-05
**158**	gender_ratio =87.208-100.346 (p11-83) &den_road >6.022 (p29) &build_area_pp <92.220 (p63)	rate_local12 =0.005-0.106 (p45-80)	55.57	0.64	26.10	0.08	7.85E-05
**159**	med_income <19678.979 (p65) &prop_open_recreation =0.014-0.146 (p34-89) &POI_pp_mall_mkt <9.163 (p66)	rate_local12 =0.005-0.124 (p45-82)	50.92	0.76	26.01	0.09	3.71E-05
**160**	gender_ratio =87.307-100.667 (p11-84) &prop_preprim_edu =0.081-0.137 (p20-85) &den_road >5.813 (p28)	rate_local12 =0.005-0.105 (p45-80)	55.75	0.63	25.98	0.08	5.38E-05
**161**	gender_ratio =87.963-98.463 (p13-78) &den_population >15872.761 (p47) &POI_pp_edu <19.468 (p74)	rate_local12 =0.005-0.104 (p45-80)	52.39	0.66	25.81	0.05	8.13E-05
**162**	prop_over65 =0.136-0.216 (p31-91) &prop_transport >0.055 (p33) &POI_pp_sports <17.325 (p50)	rate_local12 =0.005-0.114 (p45-81)	45.82	0.81	25.80	0.08	4.4E-05
**163**	med_area_home <16.408 (p49) &LU_entropy >0.538 (p44) &POI_pp_telecom_elec <16.099 (p59)	rate_local12 =0.005-0.090 (p45-77)	44.39	0.74	25.76	0.09	3.94E-05
**164**	gender_ratio =87.747-98.988 (p11-79) &prop_higher_edu <0.306 (p76) &den_public_trans =9.671-95.636 (p38-92)	rate_local12 =0.005-0.106 (p45-81)	49.31	0.71	25.74	0.09	3.01E-05

**165**	build_area_pp <89.794 (p62) &prop_business =0.001-0.111 (p29-92)	rate_local12 =0.006-0.105 (p45-80)	59.08	0.60	25.66	0.12	3.97E-06
**166**	prop_preprim_edu =0.081-0.138 (p20-86) &med_area_home <16.970 (p52)	rate_local12 =0.004-0.106 (p45-81)	54.51	0.64	25.66	0.10	1.35E-06
**167**	prop_over65 =0.117-0.212 (p16-89) &prop_business =0.003-0.111 (p43-92) &POI_pp_transport <45.188 (p69)	rate_local12 =0.005-0.127 (p45-83)	50.71	0.76	25.59	0.10	3.8E-05
**168**	prop_over65 =0.122-0.213 (p21-89) &prop_preprim_edu =0.081-0.137 (p20-85) &med_area_home <18.212 (p55)	rate_local12 =0.005-0.118 (p45-82)	50.71	0.73	25.58	0.09	5.76E-06
**169**	gender_ratio =87.588-98.254 (p11-78) &build_area_pp <88.754 (p62) &prop_transport >0.062 (p35)	rate_local12 =0.006-0.110 (p45-81)	52.99	0.67	25.51	0.09	5.33E-05
**170**	med_area_home <18.914 (p57) &LU_entropy >0.542 (p46) &POI_pp_sports <29.788 (p66)	rate_local12 =0.005-0.096 (p45-77)	47.11	0.70	25.45	0.09	8.18E-05
**171**	gender_ratio =87.683-99.716 (p11-81) &prop_preprim_edu =0.082-0.138 (p21-86) &prop_open_recreation =0.013-0.147 (p33-89)	rate_local12 =0.005-0.103 (p45-79)	47.20	0.73	25.45	0.11	1.82E-05
**172**	med_area_home <16.776 (p50) &LU_entropy >0.549 (p48) &POI_pp_mall_mkt <10.234 (p72)	rate_local12 =0.005-0.097 (p45-77)	44.48	0.76	25.39	0.08	4.2E-05
**173**	prop_preprim_edu =0.080-0.139 (p19-86) &den_public_trans =7.565-92.691 (p36-91) &prop_gov_insti_faci =0.019-0.133 (p32-90)	rate_local12 =0.005-0.105 (p45-80)	48.95	0.70	25.36	0.08	6.17E-05
**174**	gender_ratio =87.864-98.787 (p11-78) &prop_higher_edu <0.315 (p78) &prop_open_recreation =0.016-0.154 (p36-90)	rate_local12 =0.007-0.129 (p45-83)	49.81	0.77	25.32	0.09	4.84E-05
**175**	med_income <18862.550 (p62) &prop_open_recreation =0.018-0.143 (p36-89) &POI_pp_transport <30.172 (p52)	rate_local12 =0.006-0.124 (p45-82)	46.51	0.82	25.30	0.08	5.81E-05
**176**	prop_over65 =0.123-0.216 (p21-91) &den_public_trans =7.206-96.133 (p35-92) &build_area_pp <106.529 (p71)	rate_local12 =0.005-0.111 (p45-81)	50.04	0.71	25.30	0.11	1.82E-05

**177**	prop_over65 =0.111-0.206 (p12-85) &den_road >5.381 (p26) &build_area_pp <86.041 (p61)	rate_local12 =0.005-0.113 (p45-81)	52.45	0.68	25.18	0.09	5.41E-05
**178**	prop_business =0.002-0.094 (p41-91) &POI_pp_mall_mkt <8.302 (p64)	rate_local12 =0.005-0.115 (p45-81)	54.76	0.66	25.15	0.12	2.89E-05
**179**	prop_higher_edu <0.350 (p83) &den_public_trans =5.199-95.141 (p32-92) &prop_open_recreation =0.014-0.146 (p34-89)	rate_local12 =0.005-0.119 (p45-82)	49.74	0.73	25.12	0.12	2.09E-05
**180**	prop_preprim_edu =0.081-0.136 (p20-85) &prop_gov_insti_faci =0.026-0.139 (p38-90)	rate_local12 =0.005-0.106 (p45-81)	52.31	0.66	25.11	0.14	2E-06
**181**	med_income <18399.947 (p61) &prop_open_recreation =0.015-0.137 (p35-87) &POI_pp_edu <15.117 (p67)	rate_local12 =0.005-0.132 (p45-83)	48.30	0.80	25.07	0.11	1.94E-05
**182**	prop_over65 =0.122-0.209 (p20-88) &den_population >14284.410 (p45) &build_area_pp <81.232 (p57)	rate_local12 =0.006-0.130 (p45-83)	49.95	0.77	25.05	0.11	6.17E-05
**183**	gender_ratio =87.587-97.703 (p11-77) &prop_gov_insti_faci =0.016-0.142 (p31-91)	rate_local12 =0.005-0.103 (p45-79)	56.26	0.60	24.99	0.13	1.66E-06
**184**	prop_business <0.058 (p86) &prop_open_recreation =0.018-0.144 (p36-89) &POI_pp_telecom_elec <18.260 (p65)	rate_local12 =0.005-0.106 (p45-80)	48.87	0.69	24.95	0.07	5.66E-05
**185**	den_public_trans =7.206-96.133 (p35-92) &build_area_pp <87.159 (p62)	rate_local12 =0.006-0.112 (p45-81)	55.30	0.64	24.94	0.13	2.81E-06
**186**	med_area_home <18.994 (p58) &den_road =5.778-21.939 (p28-86) &POI_pp_edu <20.269 (p76)	rate_local12 =0.006-0.104 (p45-80)	49.83	0.67	24.91	0.07	8.27E-05
**187**	prop_over65 =0.106-0.205 (p11-85) &prop_preprim_edu =0.081-0.137 (p20-85) &den_population >14552.292 (p46)	rate_local12 =0.005-0.119 (p45-82)	50.53	0.72	24.88	0.10	1.51E-05
**188**	prop_business =0.003-0.111 (p43-92) &POI_pp_transport <45.188 (p69)	rate_local12 =0.005-0.108 (p45-81)	55.13	0.63	24.84	0.09	4.86E-05
**189**	prop_business =0.002-0.094 (p41-91) &POI_pp_edu <14.054 (p64)	rate_local12 =0.005-0.103 (p45-79)	51.46	0.65	24.79	0.13	5.4E-05
**190**	med_area_home <18.670 (p57) &build_area_pp <62.119 (p50)	rate_local12 =0.004-0.129 (p45-83)	57.16	0.67	24.78	0.09	8.19E-05

**191**	prop_preprim_edu =0.081-0.138 (p20-85) &med_area_home <16.776 (p50) &LU_entropy >0.549 (p48)	rate_local12 =0.005-0.088 (p45-76)	41.11	0.77	24.67	0.11	1.3E-06
**192**	med_area_home <15.461 (p45) &prop_open_recreation =0.014-0.146 (p34-89)	rate_local12 =0.005-0.128 (p45-83)	50.48	0.74	24.66	0.11	3.31E-05
**193**	gender_ratio =87.684-98.728 (p11-78) &prop_preprim_edu =0.081-0.136 (p21-85)	rate_local12 =0.006-0.110 (p45-81)	60.64	0.58	24.59	0.10	1.32E-05
**194**	gender_ratio =87.809-99.344 (p11-79) &med_income <18399.947 (p61) &den_public_trans =8.785-97.322 (p38-92)	rate_local12 =0.006-0.105 (p45-80)	45.92	0.73	24.58	0.10	1.06E-05
**195**	prop_preprim_edu =0.081-0.135 (p21-85) &build_area_pp <105.614 (p70)	rate_local12 =0.005-0.111 (p45-81)	61.62	0.58	24.55	0.10	1.69E-05
**196**	prop_higher_edu <0.304 (p76) &build_area_pp <99.847 (p67) &prop_open_recreation =0.015-0.145 (p35-89)	rate_local12 =0.004-0.135 (p45-84)	51.02	0.75	24.54	0.09	9.79E-06
**197**	prop_preprim_edu >0.077 (p16) &prop_open_recreation =0.014-0.146 (p34-89) &POI_pp_edu <21.521 (p79)	rate_local12 =0.005-0.127 (p45-82)	56.16	0.67	24.50	0.06	6.68E-05
**198**	gender_ratio =87.049-99.226 (p11-79) &prop_open_recreation =0.016-0.154 (p36-90)	rate_local12 =0.005-0.122 (p45-82)	54.50	0.67	24.50	0.15	7.43E-08
**199**	prop_over65 =0.118-0.213 (p17-89) &prop_higher_edu <0.298 (p76) &den_population >14359.348 (p46)	rate_local12 =0.006-0.134 (p45-84)	52.51	0.73	24.49	0.09	5.98E-05
**200**	prop_higher_edu <0.309 (p76) &den_public_trans =6.527-94.144 (p34-92)	rate_local12 =0.006-0.125 (p45-82)	58.46	0.64	24.44	0.11	1.28E-05
**201**	gender_ratio =87.489-97.990 (p11-78) &prop_higher_edu <0.320 (p79) &prop_gov_insti_faci >0.025 (p36)	rate_local12 =0.006-0.120 (p45-82)	52.61	0.68	24.30	0.08	6.31E-05
**202**	med_area_home <17.043 (p52) &prop_business =0.003-0.052 (p43-86)	rate_local12 =0.005-0.115 (p45-81)	46.47	0.75	24.25	0.18	2.65E-07
**203**	prop_over65 =0.117-0.212 (p16-89) &prop_preprim_edu =0.081-0.137 (p20-85) &den_public_trans =9.671-95.636 (p38-92)	rate_local12 =0.006-0.114 (p45-81)	47.73	0.72	24.22	0.10	1.31E-05

**204**	prop_over65 =0.134-0.212 (p29-89) &gender_ratio =87.597-100.434 (p11-83) &den_population >16895.676 (p49)	rate_local12 =0.005-0.110 (p45-81)	45.88	0.74	24.16	0.11	8.24E-05
**205**	build_area_pp <87.017 (p62) &prop_open_recreation =0.014-0.146 (p34-89) &POI_pp_edu <21.521 (p79)	rate_local12 =0.005-0.143 (p45-86)	52.91	0.74	24.15	0.05	5.49E-05
**206**	gender_ratio =87.663-98.155 (p11-78) &prop_preprim_edu >0.080 (p19) &prop_transport >0.068 (p36)	rate_local12 =0.005-0.103 (p45-79)	51.63	0.63	24.14	0.06	3.27E-05
**207**	gender_ratio =87.685-98.649 (p11-78) &build_area_pp <88.479 (p62)	rate_local12 =0.005-0.104 (p45-80)	58.45	0.57	24.10	0.10	3.36E-05
**208**	prop_over65 =0.139-0.216 (p34-91) &prop_gov_insti_faci =0.025-0.148 (p36-92) &POI_pp_sports <24.080 (p62)	rate_local12 =0.007-0.127 (p45-83)	44.54	0.81	23.87	0.11	4.75E-05
**209**	prop_over65 =0.123-0.211 (p21-89) &prop_higher_edu <0.304 (p76) &prop_open_recreation =0.014-0.146 (p34-89)	rate_local12 =0.004-0.135 (p45-84)	48.75	0.76	23.83	0.11	2.44E-05
**210**	den_road >5.911 (p28) &build_area_pp <87.159 (p62)	rate_local12 =0.005-0.114 (p45-81)	59.21	0.59	23.78	0.10	2.1E-06
**211**	prop_publicResid >0.039 (p70) &POI_pp_sports <23.417 (p59)	rate_local12 =0.005-0.110 (p45-81)	44.17	0.75	23.68	0.09	3.68E-05
**212**	gender_ratio =87.716-98.641 (p11-78) &build_area_pp <119.974 (p76) &LU_entropy >0.550 (p49)	rate_local12 =0.006-0.105 (p45-80)	47.28	0.67	23.52	0.09	3.08E-05
**213**	prop_business <0.069 (p87) &prop_open_recreation =0.022-0.138 (p41-87) &POI_pp_edu <21.487 (p79)	rate_local12 =0.005-0.114 (p45-81)	48.59	0.68	23.44	0.07	4.07E-05
**214**	gender_ratio =87.077-98.124 (p11-78) &den_population >17229.025 (p50)	rate_local12 =0.006-0.100 (p45-78)	50.85	0.61	23.43	0.12	9.71E-07
**215**	prop_publicResid >0.039 (p70) &POI_pp_edu <15.052 (p67)	rate_local12 =0.005-0.114 (p45-81)	43.95	0.76	23.40	0.09	2.65E-05
**216**	den_bldg =0.084-0.338 (p29-77) &POI_pp_telecom_elec <14.644 (p58)	rate_local12 =0.004-0.075 (p45-73)	42.90	0.62	23.39	0.13	6.01E-05
**217**	med_income <18399.947 (p61) &den_public_trans =8.785-97.322 (p38-92)	rate_local12 =0.005-0.125 (p45-82)	51.82	0.68	23.30	0.14	2.23E-06

**218**	prop_higher_edu <0.356 (p84) &prop_gov_insti_faci =0.026-0.139 (p38-90)	rate_local12 =0.004-0.133 (p45-84)	60.47	0.63	23.29	0.06	6.7E-05
**219**	prop_higher_edu <0.307 (p76) &den_population >15872.761 (p47)	rate_local12 =0.005-0.132 (p45-83)	57.34	0.65	23.27	0.09	1.01E-05
**220**	build_area_pp <85.719 (p60) &prop_transport >0.074 (p36)	rate_local12 =0.005-0.114 (p45-81)	55.73	0.61	23.27	0.11	2.76E-05
**221**	med_area_home <17.127 (p52) &den_bldg =0.084-0.337 (p29-77) &POI_pp_edu <15.194 (p67)	rate_local12 =0.005-0.073 (p45-73)	38.00	0.72	23.26	0.11	2.99E-05
**222**	prop_over65 =0.117-0.212 (p16-89) &build_area_pp <88.565 (p62) &LU_entropy >0.522 (p40)	rate_local12 =0.006-0.111 (p45-81)	44.54	0.73	23.24	0.11	3.15E-05
**223**	den_public_trans =7.631-93.088 (p36-92) &prop_gov_insti_faci =0.015-0.131 (p30-89)	rate_local12 =0.005-0.116 (p45-81)	54.44	0.62	23.08	0.10	1.59E-05
**224**	den_population >14660.093 (p46) &build_area_pp <75.037 (p54)	rate_local12 =0.005-0.128 (p45-83)	55.19	0.65	23.03	0.11	2.3E-05
**225**	gender_ratio =87.593-98.655 (p11-78) &prop_transport >0.076 (p36)	rate_local12 =0.005-0.104 (p45-80)	55.95	0.57	22.99	0.11	2.43E-05
**226**	den_bldg =0.082-0.432 (p28-83) &POI_pp_edu <17.921 (p71)	rate_local12 =0.005-0.098 (p45-78)	53.11	0.57	22.97	0.12	3.39E-05
**227**	prop_higher_edu <0.315 (p78) &prop_open_recreation =0.016-0.154 (p36-90)	rate_local12 =0.005-0.122 (p45-82)	54.56	0.64	22.94	0.12	8.09E-06
**228**	prop_preprim_edu =0.082-0.138 (p21-86) &prop_open_recreation =0.013-0.147 (p33-89)	rate_local12 =0.006-0.111 (p45-81)	51.20	0.63	22.93	0.15	1.77E-05
**229**	med_area_home <17.901 (p55) &LU_entropy >0.558 (p50)	rate_local12 =0.006-0.078 (p45-74)	42.28	0.63	22.88	0.17	1.84E-05
**230**	prop_higher_edu <0.304 (p76) &prop_business =0.003-0.081 (p43-89)	rate_local12 =0.006-0.111 (p45-81)	48.79	0.66	22.79	0.11	5.18E-06
**231**	gender_ratio =87.355-100.048 (p11-83) &den_road >6.022 (p29)	rate_local12 =0.005-0.106 (p45-80)	61.61	0.54	22.74	0.09	8.41E-06
**232**	LU_entropy >0.550 (p49) &POI_pp_telecom_elec <21.311 (p71)	rate_local12 =0.005-0.095 (p45-77)	51.65	0.57	22.71	0.11	5.7E-05
**233**	med_area_home <16.093 (p47)	rate_local12 =0.005-0.136 (p45-85)	61.30	0.62	22.56	0.23	1.16E-12
**234**	prop_over65 =0.134-0.212 (p29-89) &den_population >16895.676 (p49)	rate_local12 =0.005-0.136 (p45-86)	52.67	0.68	22.44	0.11	5.68E-06

**235**	med_income <18557.524 (p62) &prop_open_recreation =0.016-0.154 (p36-90)	rate_local12 =0.006-0.123 (p45-82)	50.91	0.66	22.40	0.14	1.12E-05
**236**	prop_over65 =0.117-0.212 (p16-89) &prop_business <0.076 (p89) &prop_open_recreation =0.022-0.142 (p40-88)	rate_local12 =0.005-0.108 (p45-81)	43.80	0.70	22.33	0.09	2.25E-05
**237**	LU_entropy >0.550 (p49) &POI_pp_sports <32.777 (p69)	rate_local12 =0.005-0.096 (p45-77)	51.80	0.57	22.31	0.10	3.38E-05
**238**	den_bldg =0.082-0.432 (p28-83) &POI_pp_mall_mkt <10.489 (p74)	rate_local12 =0.005-0.104 (p45-80)	54.18	0.57	22.29	0.11	5E-05
**239**	med_area_home <18.845 (p57) &den_road =5.778-21.939 (p28-86)	rate_local12 =0.006-0.104 (p45-80)	49.60	0.61	22.19	0.11	6.55E-05
**240**	med_area_home <18.914 (p57) &den_bldg =0.064-0.396 (p24-82)	rate_local12 =0.005-0.096 (p45-77)	47.37	0.61	22.12	0.13	3.33E-05
**241**	prop_preprim_edu =0.081-0.136 (p21-85) &den_road =5.778-21.939 (p28-86)	rate_local12 =0.006-0.098 (p45-78)	49.60	0.59	22.03	0.13	7.38E-06
**242**	build_area_pp <84.939 (p59) &LU_entropy >0.547 (p47)	rate_local12 =0.006-0.099 (p45-78)	44.72	0.64	22.03	0.17	3.83E-06
**243**	med_income <18344.977 (p61) &prop_business =0.003-0.052 (p43-86)	rate_local12 =0.005-0.114 (p45-81)	43.78	0.71	22.00	0.15	8.28E-06
**244**	build_area_pp <90.950 (p62) &prop_gov_insti_faci =0.009-0.141 (p21-91)	rate_local12 =0.006-0.122 (p45-82)	59.14	0.59	21.97	0.09	5.44E-05
**245**	prop_over65 =0.117-0.212 (p16-89) &den_public_trans =12.407-94.657 (p42-92) &LU_entropy >0.503 (p34)	rate_local12 =0.005-0.108 (p45-81)	42.30	0.72	21.95	0.10	5.25E-05
**246**	prop_over65 =0.117-0.212 (p16-89) &gender_ratio =87.732-98.813 (p11-78)	rate_local12 =0.005-0.108 (p45-81)	59.78	0.54	21.90	0.08	8.1E-05
**247**	den_bldg =0.084-0.337 (p29-77) &POI_pp_sports <23.905 (p60)	rate_local12 =0.004-0.068 (p45-73)	40.73	0.57	21.79	0.13	5.72E-05
**248**	den_public_trans =6.908-78.515 (p35-88) &LU_entropy >0.549 (p48)	rate_local12 =0.005-0.088 (p45-76)	44.37	0.60	21.62	0.14	5.05E-06
**249**	prop_gov_insti_faci =0.026-0.139 (p38-90) &LU_entropy >0.548 (p48)	rate_local12 =0.004-0.089 (p45-77)	42.38	0.63	21.62	0.16	3.71E-06
**250**	prop_over65 =0.133-0.179 (p28-68) &build_area_pp <72.374 (p54)	rate_local12 =0.004-0.130 (p45-83)	39.72	0.83	21.52	0.28	1.45E-08

**251**	prop_gov_insti_faci =0.023-0.137 (p33-90) &prop_open_recreation =0.019-0.140 (p37-88)	rate_local12 =0.005-0.098 (p45-78)	46.03	0.61	21.52	0.12	2.09E-05
**252**	med_income <18636.855 (p62) &den_population >14660.093 (p46)	rate_local12 =0.007-0.140 (p45-86)	54.78	0.65	21.51	0.09	4.47E-05
**253**	prop_over65 =0.120-0.183 (p19-72) &prop_preprim_edu =0.081-0.138 (p20-86)	rate_local12 =0.004-0.137 (p45-86)	52.54	0.66	21.47	0.14	5.05E-07
**254**	prop_publicResid >0.033 (p69)	rate_local12 =0.005-0.112 (p45-81)	46.27	0.65	21.37	0.30	8.82E-13
**255**	prop_open_recreation =0.019-0.159 (p36-90) &LU_entropy >0.547 (p47)	rate_local12 =0.005-0.101 (p45-78)	43.84	0.64	21.18	0.16	7.89E-05
**256**	gender_ratio =87.716-98.641 (p11-78) &LU_entropy >0.550 (p49)	rate_local12 =0.004-0.096 (p45-77)	48.52	0.57	21.06	0.10	6.48E-05
**257**	prop_over65 =0.129-0.215 (p26-91) &prop_business =0.003-0.041 (p42-85) &POI_pp_edu <14.779 (p67)	rate_local12 =0.005-0.125 (p45-82)	38.25	0.83	20.97	0.14	1.71E-06
**258**	den_population >16250.026 (p48) &LU_entropy >0.555 (p50)	rate_local12 =0.005-0.094 (p45-77)	39.90	0.67	20.94	0.20	3.66E-07
**259**	prop_higher_edu <0.320 (p79) &prop_transport >0.078 (p36)	rate_local12 =0.005-0.119 (p45-82)	57.54	0.57	20.80	0.09	4.83E-05
**260**	build_area_pp <95.672 (p65) &prop_utilities >0.007 (p55) &POI_pp_edu <20.556 (p76)	rate_local12 =0.006-0.115 (p45-81)	41.55	0.70	20.56	0.09	3.7E-05
**261**	prop_higher_edu <0.323 (p79) &den_road >6.778 (p31)	rate_local12 =0.005-0.112 (p45-81)	58.02	0.54	20.47	0.10	1E-05
**262**	prop_over65 =0.118-0.211 (p17-89) &prop_open_recreation =0.011-0.126 (p32-84) &LU_entropy >0.549 (p48)	rate_local12 =0.005-0.121 (p45-82)	37.03	0.82	20.38	0.15	5.11E-05
**263**	prop_publicResid >0.061 (p74) &POI_pp_telecom_elec <14.827 (p58)	rate_local12 =0.006-0.123 (p45-82)	38.98	0.77	20.33	0.07	4.94E-05
**264**	prop_business <0.067 (p87) &prop_open_recreation =0.020-0.144 (p38-89)	rate_local12 =0.005-0.109 (p45-81)	49.76	0.58	20.28	0.07	6.04E-05
**265**	med_income <20959.885 (p73) &prop_gov_insti_faci >0.025 (p36)	rate_local12 =0.005-0.124 (p45-82)	57.59	0.57	20.28	0.09	4.26E-05
**266**	prop_gov_insti_faci =0.026-0.139 (p38-90)	rate_local12 =0.004-0.133 (p45-84)	63.77	0.57	20.16	0.18	1.39E-09
**267**	prop_over65 =0.130-0.185 (p26-73) &prop_open_recreation =0.019-0.159 (p36-90)	rate_local12 =0.006-0.142 (p45-86)	43.41	0.74	20.00	0.19	3.09E-05

**268**	prop_transport >0.098 (p43) &LU_entropy >0.558 (p50)	rate_local12 =0.005-0.103 (p45-79)	41.04	0.65	19.99	0.17	9.82E-07
**269**	prop_over65 =0.129-0.212 (p25-89) &prop_business =0.001-0.043 (p37-85)	rate_local12 =0.006-0.110 (p45-81)	42.94	0.65	19.96	0.13	7.48E-05
**270**	prop_publicResid >0.061 (p74) &POI_pp_mall_mkt <7.577 (p63)	rate_local12 =0.006-0.123 (p45-82)	38.04	0.77	19.81	0.07	4.94E-05
**271**	den_road >7.233 (p34) &LU_entropy >0.548 (p48)	rate_local12 =0.006-0.100 (p45-78)	45.20	0.58	19.71	0.11	2.45E-05
**272**	prop_business =0.003-0.111 (p43-92)	rate_local12 =0.006-0.108 (p45-81)	55.62	0.53	19.67	0.19	5.52E-10
**273**	prop_over65 =0.123-0.210 (p21-89) &den_population >25589.345 (p58) &prop_business <0.052 (p86)	rate_local12 =0.005-0.108 (p45-81)	36.90	0.74	19.65	0.12	1.85E-06
**274**	den_population >15220.411 (p47)	rate_local12 =0.005-0.133 (p45-84)	64.41	0.56	19.61	0.17	1.49E-10
**275**	den_public_trans =4.501-90.772 (p29-91)	rate_local12 =0.006-0.181 (p45-90)	77.15	0.59	19.38	0.15	2.96E-09
**276**	build_area_pp <46.072 (p36)	rate_local12 =0.006-0.116 (p45-81)	47.26	0.60	19.22	0.25	1.69E-09
**277**	prop_over65 =0.129-0.215 (p26-91) &den_road >5.180 (p25) &prop_business <0.053 (p86)	rate_local12 =0.006-0.115 (p45-81)	48.50	0.59	19.02	0.07	4.59E-05
**278**	med_income <19374.821 (p63) &den_road >6.778 (p31)	rate_local12 =0.005-0.119 (p45-82)	53.52	0.56	18.89	0.11	5.61E-05
**279**	med_income <19067.492 (p63) &prop_transport >0.066 (p35)	rate_local12 =0.006-0.133 (p45-84)	55.24	0.59	18.86	0.10	5.69E-05
**280**	prop_over65 =0.129-0.192 (p26-78) &med_income <16507.205 (p51) &POI_pp_transport <80.520 (p83)	rate_local12 =0.006-0.142 (p45-86)	39.70	0.75	18.59	0.08	5.38E-05
**281**	gender_ratio =87.699-97.657 (p11-77)	rate_local12 =0.006-0.107 (p45-81)	65.31	0.47	18.34	0.13	7.02E-09
**282**	prop_preprim_edu =0.081-0.128 (p20-82)	rate_local12 =0.005-0.115 (p45-81)	63.85	0.50	17.90	0.14	1.93E-08
**283**	build_area_pp <89.111 (p62) &prop_utilities >0.007 (p55)	rate_local12 =0.006-0.115 (p45-81)	40.70	0.63	17.80	0.14	2.95E-05
**284**	prop_open_recreation =0.020-0.120 (p38-82)	rate_local12 =0.005-0.132 (p45-83)	54.10	0.57	17.78	0.19	3.09E-07
**285**	den_bldg <0.427 (p83) &den_public_trans >27.922 (p60) &POI_pp_sports <30.677 (p68)	rate_local12 =0.005-0.103 (p45-79)	32.42	0.73	17.62	0.11	5.22E-05
**286**	POI_pp_mall_mkt =1.108-4.008 (p11-46)	rate_local12 =0.005-0.103 (p45-79)	41.24	0.58	17.55	0.25	9.66E-10
**287**	den_road >7.576 (p36) &prop_business <0.058 (p86)	rate_local12 =0.005-0.101 (p45-78)	52.32	0.49	17.15	0.07	6.13E-05

**288**	prop_grassland =0.005-0.037 (p29-58) &POI_pp_edu <13.833 (p62)	rate_local12 =0.005-0.106 (p45-80)	31.55	0.74	17.02	0.22	6.64E-05
**289**	den_road =6.923-22.238 (p33-86)	rate_local12 =0.005-0.103 (p45-79)	53.40	0.48	16.59	0.15	2.51E-06
**290**	prop_over65 =0.141-0.188 (p36-76) &med_income <16341.219 (p50) &POI_pp_telecom_elec <27.683 (p78)	rate_local12 =0.006-0.142 (p45-86)	33.92	0.78	16.55	0.09	5.04E-05
**291**	prop_grassland =0.005-0.037 (p29-58) &POI_pp_telecom_elec <18.319 (p65)	rate_local12 =0.006-0.103 (p45-79)	31.60	0.70	16.52	0.18	7.27E-05
**292**	den_bldg <0.291 (p72) &prop_transport >0.106 (p44) &POI_pp_telecom_elec <17.011 (p61)	rate_local12 =0.002-0.072 (p45-73)	28.09	0.65	16.37	0.13	5.07E-05
**293**	prop_grassland =0.005-0.037 (p29-58) &POI_pp_mall_mkt <9.372 (p66)	rate_local12 =0.005-0.103 (p45-79)	31.66	0.69	16.28	0.17	7.98E-05
**294**	prop_over65 =0.136-0.177 (p31-66) &LU_entropy >0.568 (p53)	rate_local12 =0.005-0.091 (p45-77)	27.45	0.77	16.26	0.28	2E-06
**295**	LU_entropy >0.543 (p46)	rate_local12 =0.006-0.106 (p45-80)	54.87	0.47	15.52	0.13	1.6E-06
**296**	prop_transport =0.064-0.335 (p35-87)	rate_local12 =0.005-0.086 (p45-76)	50.68	0.43	15.40	0.13	3.04E-06
**297**	prop_grassland =0.005-0.037 (p29-58) &POI_pp_transport <58.495 (p77)	rate_local12 =0.005-0.103 (p45-79)	31.96	0.64	15.33	0.13	6.27E-05
**298**	prop_over65 =0.136-0.177 (p31-66)	rate_local12 =0.005-0.144 (p45-86)	45.49	0.60	14.92	0.20	1.47E-06
**299**	prop_grassland =0.005-0.037 (p29-58) &POI_pp_sports <42.413 (p79)	rate_local12 =0.005-0.108 (p45-81)	32.44	0.63	14.79	0.11	8.33E-05
**300**	den_bldg <0.427 (p83) &den_public_trans >27.922 (p60)	rate_local12 =0.005-0.103 (p45-79)	32.42	0.61	14.53	0.13	1.12E-05
**301**	prop_over65 =0.141-0.188 (p36-76) &med_income <16341.219 (p50)	rate_local12 =0.006-0.142 (p45-86)	34.41	0.69	14.41	0.14	5.91E-05
**302**	prop_open_recreation =0.023-0.101 (p43-78) &prop_transport =0.069-0.252 (p36-74)	rate_local12 =0.006-0.116 (p45-81)	26.65	0.74	13.75	0.20	3.16E-05
**303**	den_population >17626.686 (p50) &den_bldg =0.077-0.277 (p26-70)	rate_local12 =0.002-0.056 (p45-68)	24.27	0.48	13.59	0.14	2.47E-05
**304**	den_bldg =0.058-0.360 (p21-80)	rate_local12 =0.006-0.070 (p45-73)	46.71	0.38	13.40	0.11	3.74E-05
**305**	den_bldg <0.291 (p72) &prop_transport >0.106 (p44)	rate_local12 =0.002-0.072 (p45-73)	28.18	0.52	13.33	0.14	2.24E-05
**306**	med_area_home <18.868 (p57) &prop_shrubland =0.009-0.082 (p30-53) &POI_pp_mall_mkt <9.766 (p69)	rate_local12 =0.008-0.083 (p45-75)	20.57	0.77	12.77	0.11	7.24E-05

**307**	med_area_home <18.153 (p55) &prop_shrubland =0.009-0.082 (p30-53) &POI_pp_telecom_elec <18.844 (p67)	rate_local12 =0.008-0.083 (p45-75)	20.28	0.77	12.63	0.09	7.24E-05
**308**	med_income <18518.024 (p62)	rate_local12 =0.006-0.136 (p45-85)	62.63	0.48	12.12	0.09	1.23E-05
**309**	den_public_trans >28.630 (p61) &prop_shrubland =0.007-0.095 (p30-55)	rate_local12 =0.007-0.101 (p45-78)	20.20	0.79	11.83	0.30	2.46E-06
**310**	prop_shrubland =0.001-0.085 (p21-53)	rate_local12 =0.007-0.092 (p45-77)	33.46	0.48	11.76	0.17	3.94E-05
**311**	med_area_home <18.153 (p55) &prop_shrubland =0.009-0.082 (p30-53)	rate_local12 =0.008-0.083 (p45-75)	20.43	0.69	11.73	0.21	6.21E-05
**312**	prop_grassland =0.005-0.037 (p29-58)	rate_local12 =0.005-0.104 (p45-80)	32.03	0.52	11.31	0.18	4.87E-05
**313**	POI_pp_mall_mkt <1.108 (p11)	rate_local12 =0.005-0.103 (p45-79)	18.91	0.78	10.78	0.44	5.47E-07
**314**	den_population >16895.676 (p49) &prop_private_resid =0.011-0.101 (p28-66)	rate_local12 =0.002-0.046 (p45-63)	17.60	0.43	10.53	0.16	4.54E-05
**315**	prop_over65 =0.161-0.180 (p51-69) &prop_gov_insti_faci >0.041 (p48)	rate_local12 =0.005-0.103 (p45-79)	15.33	0.73	8.36	0.23	7.63E-05
**316**	prop_private_resid =0.011-0.101 (p28-66)	rate_local12 =0.002-0.046 (p45-63)	21.97	0.28	8.18	0.10	5.72E-05
**317**	prop_gov_insti_faci <0.026 (p38)	rate_local12 <0.004 (p45)	55.42	0.71	19.81	0.25	9.29E-09
**318**	den_population <15220.411 (p47)	rate_local12 <0.005 (p45)	63.23	0.63	18.05	0.18	1.27E-08
**319**	prop_open_recreation <0.020 (p38)	rate_local12 <0.005 (p45)	55.16	0.67	17.87	0.22	9.89E-07
**320**	den_public_trans <4.501 (p29)	rate_local12 <0.006 (p45)	45.70	0.74	17.71	0.29	1.31E-07
**321**	prop_private_resid <0.011 (p28)	rate_local12 <0.002 (p45)	43.51	0.74	16.71	0.28	1.14E-07
**322**	POI_pp_transport >75.207 (p82)	rate_local12 <0.005 (p45)	33.22	0.84	15.33	0.39	5.03E-08
**323**	prop_transport <0.064 (p35)	rate_local12 <0.005 (p45)	47.96	0.66	14.99	0.21	8.09E-06
**324**	POI_pp_telecom_elec >35.358 (p84)	rate_local12 <0.005 (p45)	29.82	0.84	13.74	0.39	1.54E-07
**325**	med_area_home >16.093 (p47)	rate_local12 <0.005 (p45)	65.96	0.57	13.52	0.12	4.79E-05
**326**	POI_pp_edu >29.153 (p86)	rate_local12 <0.005 (p45)	28.49	0.86	13.42	0.40	4.37E-07
**327**	prop_publicResid <0.033 (p69)	rate_local12 <0.005 (p45)	78.99	0.55	13.39	0.09	7.54E-05
**328**	den_road <6.923 (p33)	rate_local12 <0.005 (p45)	44.85	0.64	13.04	0.19	5.58E-05
**329**	POI_pp_mall_mkt >16.323 (p86)	rate_local12 <0.005 (p45)	27.34	0.86	12.96	0.41	4.37E-07
**330**	den_bldg <0.058 (p21)	rate_local12 <0.006 (p45)	32.47	0.72	12.09	0.27	2.17E-05
**331**	prop_over65 >0.180 (p69) &prop_gov_insti_faci <0.041 (p48)	rate_local12 <0.005 (p45)	26.50	0.82	11.90	0.20	5.4E-05
**332**	POI_pp_sports >68.635 (p87)	rate_local12 <0.005 (p45)	23.83	0.87	11.46	0.42	8.56E-06
**333**	prop_over65 >0.204 (p84)	rate_local12 <0.005 (p45)	25.42	0.76	10.35	0.31	6.96E-05

(b) Rules for wave-1 imported case rate; POI accessibility was used

No.	Antecedent	Consequent	Supp	Conf	Lev	Imp	*P*
**1**	prop_higher_edu >0.212 (p54) &build_area_pp >63.378 (p51)	rate_imported12 >0.100 (p68)	44.81	0.70	23.96	0.13	5.09E-05
**2**	prop_higher_edu >0.188 (p43) &med_area_home >16.738 (p50)	rate_imported12 >0.107 (p70)	47.59	0.61	23.03	0.12	7.23E-05
**3**	med_income >19583.009 (p65)	rate_imported12 >0.110 (p70)	44.69	0.62	22.24	0.31	1.22E-15
**4**	prop_higher_edu >0.171 (p39) &prop_industrial <0.010 (p71)	rate_imported12 >0.087 (p67)	59.44	0.57	22.04	0.08	1.61E-05
**5**	prop_preprim_edu <0.134 (p84) &med_area_home >16.409 (p49)	rate_imported12 >0.081 (p64)	54.36	0.60	20.85	0.08	4.54E-05
**6**	build_area_pp =68.225-212.874 (p52-90) &prop_industrial <0.014 (p76) &prop_agricultural <0.051 (p80)	rate_imported12 >0.106 (p70)	33.99	0.76	19.82	0.10	2.36E-05
**7**	med_area_home >17.118 (p52) &prop_agricultural <0.061 (p82)	rate_imported12 >0.099 (p68)	43.93	0.60	19.82	0.10	5.4E-05
**8**	med_income >16353.521 (p50) &prop_industrial <0.000 (p43)	rate_imported12 >0.100 (p68)	38.30	0.67	19.55	0.19	8.01E-07
**9**	build_area_pp =68.225-212.874 (p52-90) &prop_agricultural <0.063 (p82)	rate_imported12 >0.084 (p66)	40.16	0.69	19.16	0.16	2.68E-07
**10**	build_area_pp >63.378 (p51) &prop_industrial <0.002 (p55) &prop_grassland <0.086 (p71)	rate_imported12 >0.100 (p68)	33.34	0.75	18.77	0.12	3.02E-05
**11**	prop_preprim_edu <0.134 (p84) &build_area_pp >73.363 (p54)	rate_imported12 >0.108 (p70)	42.93	0.55	18.72	0.10	7.07E-05
**12**	med_area_home >17.900 (p55) &prop_grassland <0.073 (p69)	rate_imported12 >0.100 (p68)	36.50	0.67	18.61	0.16	4.18E-05
**13**	prop_higher_edu >0.210 (p53) &POI_pp_mall_mkt >7.907 (p64)	rate_imported12 >0.160 (p78)	30.38	0.61	18.61	0.17	1.96E-05
**14**	med_area_home >15.947 (p47) &prop_industrial <0.000 (p43)	rate_imported12 >0.100 (p68)	36.96	0.65	18.37	0.17	5.25E-05
**15**	build_area_pp >68.114 (p52) &prop_industrial <0.000 (p43)	rate_imported12 >0.116 (p71)	32.26	0.68	18.19	0.24	3.51E-06
**16**	prop_publicResid <0.005 (p56) &prop_industrial <0.009 (p70)	rate_imported12 >0.116 (p71)	44.50	0.50	18.04	0.10	5.08E-05
**17**	build_area_pp >66.770 (p52) &prop_grassland <0.086 (p71)	rate_imported12 >0.096 (p68)	40.89	0.60	17.91	0.12	7.06E-05
**18**	build_area_pp >71.028 (p53) &prop_rural_set <0.026 (p66)	rate_imported12 >0.120 (p71)	36.76	0.58	17.88	0.14	3.83E-05
**19**	med_area_home >18.079 (p55) &prop_rural_set <0.022 (p64)	rate_imported12 >0.161 (p78)	29.75	0.57	17.57	0.17	6.12E-06
**20**	prop_preprim_edu <0.097 (p52) &prop_publicResid <0.005 (p56)	rate_imported12 >0.146 (p74)	32.15	0.52	16.61	0.15	2.98E-05

**21**	prop_higher_edu >0.195 (p47) &POI_pp_edu >14.831 (p67)	rate_imported12 >0.181 (p79)	26.83	0.54	15.93	0.16	6.82E-05
**22**	prop_higher_edu >0.364 (p85)	rate_imported12 >0.124 (p71)	24.89	0.78	15.77	0.49	6.85E-11
**23**	build_area_pp =51.859-202.851 (p43-89)	rate_imported12 >0.105 (p69)	47.53	0.48	15.73	0.16	3.57E-07
**24**	prop_industrial <0.008 (p70) &POI_pp_sports >28.416 (p66)	rate_imported12 >0.123 (p71)	31.97	0.56	15.71	0.10	7.38E-05
**25**	prop_higher_edu >0.237 (p62) &POI_pp_telecom_elec >21.730 (p71)	rate_imported12 >0.209 (p81)	21.96	0.66	15.52	0.23	5.15E-05
**26**	prop_publicResid <0.034 (p69)	rate_imported12 >0.103 (p69)	62.20	0.43	15.37	0.11	1.72E-08
**27**	prop_agricultural <0.057 (p82) &POI_pp_telecom_elec >24.175 (p75)	rate_imported12 >0.184 (p80)	23.58	0.61	15.31	0.14	7.41E-05
**28**	prop_industrial <0.000 (p39) &POI_pp_transport >35.681 (p57)	rate_imported12 >0.179 (p79)	24.90	0.55	15.12	0.18	6.86E-05
**29**	prop_industrial <0.000 (p39) &POI_pp_telecom_elec >18.233 (p65)	rate_imported12 >0.195 (p80)	22.27	0.63	14.88	0.23	1.6E-05
**30**	POI_pp_sports >30.523 (p67)	rate_imported12 >0.196 (p80)	28.89	0.41	14.60	0.21	5.62E-08
**31**	POI_pp_telecom_elec >25.431 (p76)	rate_imported12 >0.213 (p81)	24.73	0.46	14.51	0.27	5.34E-10
**32**	POI_pp_transport >39.200 (p61)	rate_imported12 >0.188 (p80)	32.28	0.37	14.25	0.17	1.27E-07
**33**	gender_ratio >94.412 (p59) &build_area_pp >60.156 (p50) &prop_agricultural <0.014 (p61)	rate_imported12 >0.163 (p78)	20.18	0.78	14.19	0.28	7.68E-05
**34**	POI_pp_mall_mkt >10.941 (p75)	rate_imported12 >0.202 (p80)	25.16	0.45	14.06	0.25	1.15E-08
**35**	prop_industrial <0.000 (p43)	rate_imported12 >0.097 (p68)	44.29	0.49	13.89	0.15	1.39E-06
**36**	LU_entropy <0.561 (p51)	rate_imported12 >0.099 (p68)	48.70	0.45	12.73	0.12	5.05E-05
**37**	med_area_home >25.388 (p86)	rate_imported12 >0.169 (p78)	19.27	0.66	12.70	0.44	1.69E-12
**38**	POI_pp_edu >22.306 (p79)	rate_imported12 >0.187 (p80)	22.41	0.48	12.67	0.27	2.78E-08
**39**	ave_household_size >3.250 (p87)	rate_imported12 >0.110 (p70)	20.27	0.76	11.98	0.45	6.18E-09
**40**	prop_preprim_edu <0.078 (p17)	rate_imported12 >0.255 (p83)	17.96	0.50	11.72	0.33	1.27E-08
**41**	prop_over65 <0.140 (p35)	rate_imported12 >0.083 (p65)	38.57	0.53	11.66	0.16	5.07E-05
**42**	build_area_pp =83.395-186.699 (p58-88) &LU_entropy <0.563 (p52)	rate_imported12 >0.084 (p66)	23.07	0.72	11.48	0.16	4.34E-05
**43**	den_population =673.564-26483.860 (p13-60)	rate_imported12 >0.084 (p66)	46.61	0.48	11.11	0.11	2.65E-05
**44**	gender_ratio >95.715 (p69) &prop_rural_set <0.022 (p64)	rate_imported12 >0.261 (p83)	17.68	0.44	10.82	0.14	7.83E-05
**45**	gender_ratio >94.644 (p62) &prop_industrial <0.000 (p36)	rate_imported12 >0.203 (p80)	16.53	0.56	10.73	0.22	1.06E-05
**46**	gender_ratio >94.842 (p64)	rate_imported12 >0.207 (p81)	26.22	0.32	10.36	0.13	6.6E-05
**47**	prop_higher_edu =0.285-0.364 (p72-85)	rate_imported12 >0.124 (p71)	16.06	0.59	8.36	0.31	5.13E-05
**48**	gender_ratio >94.412 (p59) &den_population >20901.723 (p53) &build_area_pp >60.156 (p50)	rate_imported12 >0.189 (p80)	9.88	0.83	7.39	0.41	6.01E-05

**49**	build_area_pp >75.116 (p54) &prop_private_resid >0.239 (p85)	rate_imported12 >0.184 (p80)	8.11	0.85	6.08	0.49	6.97E-05
**50**	den_population <1544.010 (p22) &prop_private_resid =0.020-0.115 (p35-70)	rate_imported12 >0.188 (p80)	6.50	0.73	4.61	0.44	2.53E-05
**51**	den_public_trans <5.489 (p32) &prop_agricultural <0.007 (p56)	rate_imported12 >0.183 (p80)	5.97	0.70	4.16	0.44	3.06E-05
**52**	den_bldg <0.055 (p20) &prop_private_resid =0.032-0.183 (p43-80)	rate_imported12 >0.297 (p84)	4.05	0.76	3.23	0.53	2.03E-05
**53**	build_area_pp <68.225 (p52) &prop_agricultural >0.063 (p82)	rate_imported12 >0.084 (p66)	5.36	0.67	2.46	0.40	7.77E-05
**54**	prop_preprim_edu <0.131 (p83) &med_area_home <17.589 (p54) &POI_pp_transport <41.646 (p64)	rate_imported12 =0.003-0.094 (p36-68)	54.98	0.67	29.33	0.07	8.04E-05
**55**	gender_ratio <96.234 (p71) &prop_preprim_edu <0.130 (p83) &med_area_home <18.749 (p57)	rate_imported12 =0.004-0.112 (p36-71)	60.03	0.67	29.30	0.07	2.04E-05
**56**	prop_preprim_edu <0.131 (p83) &med_area_home <17.589 (p54) &POI_pp_edu <14.873 (p67)	rate_imported12 =0.003-0.095 (p36-68)	54.12	0.68	29.14	0.07	8.04E-05
**57**	prop_preprim_edu <0.131 (p83) &med_area_home <17.589 (p54) &POI_pp_mall_mkt <9.149 (p66)	rate_imported12 =0.005-0.096 (p36-68)	54.64	0.68	29.10	0.07	2.96E-05
**58**	prop_preprim_edu <0.131 (p83) &med_income <22172.304 (p77) &POI_pp_telecom_elec <15.807 (p59)	rate_imported12 =0.003-0.102 (p36-69)	59.49	0.64	28.98	0.07	4.44E-05
**59**	prop_preprim_edu <0.130 (p83) &med_income <20515.207 (p73) &POI_pp_transport <39.932 (p61)	rate_imported12 =0.005-0.110 (p36-70)	59.38	0.66	28.96	0.09	6.39E-05
**60**	prop_preprim_edu <0.131 (p83) &med_area_home <17.589 (p54) &POI_pp_sports <25.282 (p62)	rate_imported12 =0.005-0.096 (p36-68)	54.29	0.68	28.95	0.07	2.96E-05
**61**	prop_preprim_edu <0.132 (p83) &med_income <21672.073 (p75) &POI_pp_sports <22.607 (p57)	rate_imported12 =0.003-0.101 (p36-68)	57.69	0.64	28.61	0.08	5.21E-06
**62**	prop_preprim_edu <0.131 (p83) &POI_pp_transport <34.984 (p57)	rate_imported12 =0.005-0.156 (p36-76)	71.50	0.66	28.25	0.06	4.66E-06
**63**	gender_ratio <96.234 (p71) &prop_preprim_edu <0.130 (p83) &POI_pp_transport <30.187 (p52)	rate_imported12 =0.005-0.110 (p36-70)	55.65	0.69	28.25	0.05	7.59E-05
**64**	prop_preprim_edu <0.130 (p83) &prop_higher_edu <0.274 (p69) &POI_pp_transport <39.932 (p61)	rate_imported12 =0.004-0.101 (p36-68)	55.07	0.65	27.50	0.10	3.03E-05
**65**	gender_ratio <94.774 (p63) &med_income <20632.679 (p73) &POI_pp_telecom_elec <12.856 (p53)	rate_imported12 =0.002-0.093 (p36-68)	47.41	0.74	27.42	0.08	2.58E-05
**66**	med_area_home <17.092 (p52) &POI_pp_telecom_elec <11.488 (p50)	rate_imported12 =0.004-0.090 (p36-67)	51.47	0.66	27.41	0.14	8.79E-07

**67**	prop_preprim_edu <0.130 (p83) &med_income <19760.524 (p65) &POI_pp_mall_mkt <7.462 (p61)	rate_imported12 =0.005-0.100 (p36-68)	53.68	0.66	27.32	0.11	1.36E-05
**68**	prop_preprim_edu <0.131 (p83) &POI_pp_sports <22.607 (p57)	rate_imported12 =0.004-0.126 (p36-72)	66.48	0.61	27.09	0.06	3.04E-05
**69**	med_area_home <16.841 (p50) &POI_pp_transport <25.580 (p49)	rate_imported12 =0.002-0.082 (p36-65)	48.52	0.65	26.99	0.14	5.72E-06
**70**	med_area_home <16.854 (p50) &POI_pp_edu <13.203 (p60)	rate_imported12 =0.002-0.088 (p36-67)	51.63	0.64	26.96	0.12	4.57E-06
**71**	med_area_home <17.246 (p52) &prop_private_resid =0.010-0.221 (p27-85) &POI_pp_transport <32.004 (p53)	rate_imported12 =0.004-0.070 (p36-64)	41.99	0.72	26.94	0.14	5.42E-06
**72**	prop_preprim_edu <0.135 (p84) &med_income <20810.585 (p73) &build_area_pp <71.444 (p53)	rate_imported12 =0.003-0.112 (p36-71)	55.37	0.66	26.92	0.08	8.55E-06
**73**	gender_ratio <95.704 (p69) &prop_preprim_edu <0.130 (p83) &med_income <20318.595 (p73)	rate_imported12 =0.004-0.091 (p36-67)	54.44	0.61	26.91	0.10	1.34E-05
**74**	med_area_home <17.197 (p52) &prop_private_resid =0.010-0.221 (p27-85) &POI_pp_telecom_elec <10.658 (p49)	rate_imported12 =0.004-0.085 (p36-66)	43.24	0.79	26.90	0.13	1.14E-05
**75**	gender_ratio <94.103 (p58) &med_area_home <17.589 (p54) &POI_pp_mall_mkt <7.405 (p61)	rate_imported12 =0.004-0.098 (p36-68)	46.39	0.76	26.86	0.11	4.17E-05
**76**	prop_preprim_edu <0.130 (p82) &prop_higher_edu <0.241 (p63) &POI_pp_edu <10.562 (p52)	rate_imported12 =0.004-0.107 (p36-70)	49.44	0.73	26.79	0.12	1.08E-05
**77**	med_income <20533.112 (p73) &prop_agricultural <0.055 (p81) &POI_pp_transport <39.835 (p61)	rate_imported12 =0.004-0.083 (p36-65)	52.78	0.59	26.72	0.08	1.32E-05
**78**	prop_preprim_edu <0.130 (p83) &POI_pp_telecom_elec <18.220 (p65)	rate_imported12 =0.005-0.156 (p36-76)	75.58	0.62	26.67	0.05	6.75E-05
**79**	prop_preprim_edu <0.130 (p83) &POI_pp_mall_mkt <6.671 (p57)	rate_imported12 =0.005-0.156 (p36-76)	68.42	0.65	26.55	0.09	2.52E-07
**80**	med_income <20922.721 (p73) &prop_private_resid =0.010-0.230 (p27-85) &POI_pp_telecom_elec <16.754 (p61)	rate_imported12 =0.003-0.081 (p36-64)	46.47	0.67	26.53	0.11	7.92E-06
**81**	med_area_home <17.343 (p52) &prop_private_resid =0.009-0.218 (p26-84) &POI_pp_sports <24.226 (p62)	rate_imported12 =0.002-0.076 (p36-64)	43.21	0.71	26.45	0.12	4.13E-06
**82**	med_income <19986.043 (p65) &prop_private_resid =0.009-0.218 (p26-84) &POI_pp_transport <31.422 (p53)	rate_imported12 =0.004-0.085 (p36-66)	44.16	0.74	26.44	0.13	1.59E-06
**83**	med_area_home <16.888 (p50) &POI_pp_mall_mkt <4.472 (p48)	rate_imported12 =0.004-0.080 (p36-64)	46.75	0.66	26.43	0.16	1.37E-06
**84**	gender_ratio <96.234 (p71) &prop_preprim_edu <0.130 (p83) &prop_higher_edu <0.286 (p73)	rate_imported12 =0.003-0.096 (p36-68)	55.89	0.60	26.31	0.09	2.98E-05

**85**	med_area_home <17.493 (p52) &build_area_pp <62.960 (p50)	rate_imported12 =0.004-0.080 (p36-64)	49.97	0.60	26.28	0.11	5.15E-05
**86**	gender_ratio <93.540 (p51) &POI_pp_telecom_elec <13.409 (p54)	rate_imported12 =0.005-0.120 (p36-71)	49.54	0.75	26.28	0.19	3.1E-07
**87**	gender_ratio <93.909 (p56) &med_area_home <19.469 (p61) &POI_pp_telecom_elec <20.088 (p68)	rate_imported12 =0.004-0.142 (p36-74)	54.36	0.74	26.21	0.07	7.8E-05
**88**	med_area_home <17.092 (p52) &prop_private_resid =0.009-0.218 (p26-84) &POI_pp_mall_mkt <5.960 (p53)	rate_imported12 =0.004-0.075 (p36-64)	41.59	0.74	26.20	0.13	1.82E-05
**89**	med_area_home <16.816 (p50) &den_public_trans =4.903-80.627 (p31-89)	rate_imported12 =0.003-0.101 (p36-68)	51.61	0.66	26.18	0.12	4.57E-06
**90**	med_area_home <17.343 (p52) &prop_private_resid =0.009-0.218 (p26-84) &POI_pp_edu <12.314 (p57)	rate_imported12 =0.002-0.076 (p36-64)	42.39	0.72	26.17	0.13	2.65E-05
**91**	gender_ratio <93.712 (p53) &med_area_home <17.235 (p52) &POI_pp_transport <29.530 (p52)	rate_imported12 =0.002-0.097 (p36-68)	43.28	0.80	26.12	0.10	7.25E-07
**92**	build_area_pp <99.164 (p67) &prop_private_resid =0.006-0.245 (p23-86) &POI_pp_transport <24.809 (p49)	rate_imported12 =0.004-0.070 (p36-64)	42.50	0.67	26.04	0.08	5.56E-05
**93**	gender_ratio <96.234 (p71) &prop_preprim_edu <0.130 (p83) &POI_pp_mall_mkt <8.979 (p66)	rate_imported12 =0.003-0.082 (p36-65)	53.47	0.56	25.92	0.06	4.53E-05
**94**	med_income <19986.043 (p65) &prop_private_resid =0.009-0.218 (p26-84) &POI_pp_sports <17.590 (p50)	rate_imported12 =0.004-0.085 (p36-66)	42.83	0.75	25.85	0.12	3.1E-06
**95**	gender_ratio <93.573 (p52) &med_area_home <17.791 (p55) &POI_pp_edu <14.768 (p67)	rate_imported12 =0.003-0.097 (p36-68)	44.62	0.76	25.84	0.11	6.41E-06
**96**	gender_ratio <93.626 (p52) &POI_pp_transport <32.487 (p54)	rate_imported12 =0.004-0.107 (p36-70)	48.37	0.72	25.83	0.18	2.49E-07
**97**	gender_ratio <96.322 (p71) &prop_private_resid =0.010-0.230 (p27-85) &POI_pp_telecom_elec <13.444 (p55)	rate_imported12 =0.003-0.081 (p36-64)	45.74	0.66	25.80	0.07	3.29E-05
**98**	prop_preprim_edu <0.130 (p83) &prop_higher_edu <0.246 (p64) &prop_private_resid =0.010-0.230 (p27-85)	rate_imported12 =0.004-0.097 (p36-68)	45.90	0.73	25.77	0.11	1.25E-06
**99**	prop_preprim_edu <0.130 (p82) &POI_pp_edu <10.562 (p52)	rate_imported12 =0.004-0.120 (p36-71)	59.52	0.62	25.67	0.08	2.88E-05
**100**	prop_private_resid =0.010-0.221 (p27-85) &POI_pp_transport <18.338 (p40)	rate_imported12 =0.004-0.070 (p36-64)	41.24	0.69	25.66	0.14	1.2E-05
**101**	gender_ratio <93.712 (p53) &POI_pp_sports <19.192 (p52)	rate_imported12 =0.005-0.115 (p36-71)	47.99	0.74	25.64	0.20	2.95E-07
**102**	gender_ratio <93.712 (p53) &med_area_home <18.234 (p55)	rate_imported12 =0.005-0.115 (p36-71)	52.81	0.67	25.63	0.14	4.07E-06
**103**	prop_private_resid =0.009-0.201 (p26-83) &POI_pp_telecom_elec <6.513 (p36)	rate_imported12 =0.005-0.064 (p36-60)	38.46	0.70	25.53	0.14	1.25E-05

**104**	prop_preprim_edu <0.129 (p82) &prop_higher_edu <0.235 (p62) &prop_agricultural <0.046 (p79)	rate_imported12 =0.003-0.081 (p36-64)	47.55	0.62	25.51	0.10	5.4E-06
**105**	prop_preprim_edu <0.131 (p83) &med_area_home <17.718 (p54) &den_road =6.383-22.034 (p30-86)	rate_imported12 =0.004-0.085 (p36-66)	45.03	0.68	25.51	0.08	2.7E-05
**106**	prop_preprim_edu <0.135 (p84) &med_area_home <16.645 (p50) &LU_entropy >0.561 (p52)	rate_imported12 =0.003-0.087 (p36-67)	41.59	0.77	25.46	0.11	1.01E-06
**107**	med_income <20795.645 (p73) &prop_transport =0.069-0.334 (p36-87) &POI_pp_sports <12.803 (p42)	rate_imported12 =0.004-0.085 (p36-66)	40.83	0.79	25.44	0.10	1.54E-05
**108**	build_area_pp <65.661 (p52) &POI_pp_transport <24.748 (p49)	rate_imported12 =0.003-0.075 (p36-64)	46.35	0.60	25.40	0.12	1.66E-05
**109**	prop_higher_edu <0.246 (p64) &prop_private_resid =0.010-0.230 (p27-85) &POI_pp_telecom_elec <13.632 (p55)	rate_imported12 =0.004-0.087 (p36-67)	42.45	0.74	25.31	0.14	2.59E-06
**110**	med_area_home <17.016 (p52) &prop_transport =0.051-0.322 (p31-86) &POI_pp_transport <30.472 (p53)	rate_imported12 =0.004-0.089 (p36-67)	42.69	0.75	25.28	0.11	6.48E-05
**111**	prop_preprim_edu <0.130 (p83) &med_income <18949.418 (p62) &den_road =5.239-22.537 (p25-87)	rate_imported12 =0.003-0.109 (p36-70)	48.66	0.70	25.27	0.11	1.46E-06
**112**	build_area_pp <65.774 (p52) &POI_pp_telecom_elec <12.662 (p51)	rate_imported12 =0.003-0.077 (p36-64)	47.74	0.59	25.26	0.12	2.5E-05
**113**	prop_preprim_edu <0.130 (p83) &med_income <21622.419 (p75) &prop_private_resid =0.010-0.230 (p27-85)	rate_imported12 =0.004-0.097 (p36-68)	49.57	0.65	25.24	0.08	7.47E-06
**114**	med_income <19998.727 (p65) &prop_transport =0.069-0.334 (p36-87) &POI_pp_telecom_elec <12.856 (p53)	rate_imported12 =0.003-0.085 (p36-66)	42.13	0.74	25.23	0.13	1.67E-05
**115**	POI_pp_telecom_elec <6.513 (p36)	rate_imported12 =0.004-0.091 (p36-67)	48.88	0.64	25.23	0.33	1.78E-15
**116**	med_income <19957.502 (p65) &POI_pp_telecom_elec <12.854 (p53)	rate_imported12 =0.003-0.091 (p36-67)	51.98	0.60	25.17	0.09	2.9E-05
**117**	prop_private_resid =0.010-0.223 (p27-85) &POI_pp_sports <23.556 (p59)	rate_imported12 =0.004-0.190 (p36-80)	60.84	0.73	25.14	0.13	8.23E-07
**118**	prop_higher_edu <0.281 (p71) &prop_private_resid =0.010-0.230 (p27-85) &POI_pp_transport <37.598 (p60)	rate_imported12 =0.004-0.086 (p36-66)	44.53	0.68	25.06	0.10	3.83E-05
**119**	med_income <20342.840 (p73) &prop_agricultural <0.062 (p82) &POI_pp_sports <26.196 (p64)	rate_imported12 =0.005-0.083 (p36-65)	51.16	0.57	25.03	0.06	7.81E-05
**120**	build_area_pp <63.908 (p51) &POI_pp_sports <16.066 (p50)	rate_imported12 =0.003-0.080 (p36-64)	47.25	0.61	24.95	0.11	6.38E-05
**121**	med_area_home <17.525 (p53) &prop_transport =0.069-0.321 (p36-86) &POI_pp_telecom_elec <12.071 (p51)	rate_imported12 =0.002-0.087 (p36-67)	41.29	0.76	24.89	0.12	4.19E-05
**122**	gender_ratio <94.071 (p58) &POI_pp_mall_mkt <5.378 (p51)	rate_imported12 =0.004-0.108 (p36-70)	47.16	0.71	24.89	0.18	1.11E-06

**123**	med_income <19690.189 (p65) &prop_agricultural <0.060 (p82) &POI_pp_telecom_elec <19.914 (p68)	rate_imported12 =0.002-0.083 (p36-65)	50.90	0.57	24.84	0.06	4.41E-05
**124**	prop_preprim_edu =0.080-0.138 (p19-85) &med_area_home <17.676 (p54) &den_bldg =0.084-0.424 (p29-83)	rate_imported12 =0.003-0.079 (p36-64)	42.16	0.69	24.82	0.08	7.03E-05
**125**	med_income <19957.502 (p65) &POI_pp_transport <31.324 (p53)	rate_imported12 =0.004-0.093 (p36-68)	52.76	0.59	24.79	0.08	2.71E-05
**126**	prop_preprim_edu <0.130 (p82) &prop_higher_edu <0.228 (p61) &POI_pp_mall_mkt <6.188 (p55)	rate_imported12 =0.003-0.101 (p36-68)	46.65	0.69	24.78	0.12	2.05E-05
**127**	prop_private_resid =0.010-0.221 (p27-85) &POI_pp_edu <6.845 (p43)	rate_imported12 =0.004-0.067 (p36-62)	40.33	0.64	24.77	0.16	1.52E-06
**128**	med_income <19986.043 (p65) &prop_transport =0.065-0.333 (p35-87) &POI_pp_transport <32.480 (p54)	rate_imported12 =0.004-0.082 (p36-65)	41.95	0.71	24.77	0.12	5.04E-06
**129**	med_income <19957.502 (p65) &POI_pp_sports <23.572 (p59)	rate_imported12 =0.003-0.091 (p36-67)	55.05	0.56	24.77	0.09	4.27E-05
**130**	med_area_home <17.118 (p52) &prop_transport =0.079-0.345 (p36-90) &POI_pp_mall_mkt <4.472 (p48)	rate_imported12 =0.004-0.080 (p36-64)	38.79	0.78	24.68	0.14	3.09E-05
**131**	prop_private_resid =0.010-0.230 (p27-85) &POI_pp_mall_mkt <3.690 (p44)	rate_imported12 =0.004-0.068 (p36-62)	40.96	0.63	24.64	0.15	2.74E-06
**132**	prop_higher_edu <0.246 (p64) &prop_private_resid =0.010-0.230 (p27-85) &POI_pp_sports <18.216 (p51)	rate_imported12 =0.004-0.087 (p36-67)	41.29	0.74	24.60	0.12	2.81E-05
**133**	med_area_home <17.246 (p52) &prop_private_resid =0.010-0.221 (p27-85)	rate_imported12 =0.004-0.070 (p36-64)	44.13	0.59	24.60	0.13	2E-05
**134**	build_area_pp <65.661 (p52) &POI_pp_mall_mkt <3.751 (p44)	rate_imported12 =0.003-0.075 (p36-64)	44.19	0.61	24.46	0.11	6E-05
**135**	med_income <19761.738 (p65) &den_bldg =0.087-0.428 (p29-83) &POI_pp_telecom_elec <13.012 (p54)	rate_imported12 =0.002-0.081 (p36-64)	41.43	0.70	24.45	0.12	2.4E-05
**136**	den_public_trans =2.846-78.606 (p21-88) &build_area_pp <62.576 (p50)	rate_imported12 =0.005-0.083 (p36-66)	48.09	0.59	24.41	0.10	6.39E-05
**137**	gender_ratio <93.712 (p53) &prop_higher_edu <0.326 (p79) &POI_pp_transport <53.890 (p73)	rate_imported12 =0.001-0.097 (p36-68)	49.45	0.63	24.41	0.06	7.72E-05
**138**	med_income <20342.840 (p73) &prop_agricultural <0.062 (p82) &POI_pp_mall_mkt <7.342 (p60)	rate_imported12 =0.005-0.083 (p36-65)	49.46	0.58	24.41	0.08	6.2E-05
**139**	prop_higher_edu <0.249 (p64) &POI_pp_sports <8.775 (p32)	rate_imported12 =0.004-0.070 (p36-63)	38.34	0.71	24.37	0.11	5.15E-05
**140**	gender_ratio <93.626 (p52) &POI_pp_edu <10.116 (p50)	rate_imported12 =0.004-0.096 (p36-68)	44.18	0.71	24.36	0.20	2.32E-07
**141**	prop_gov_insti_faci >0.027 (p38) &POI_pp_transport <41.966 (p64)	rate_imported12 =0.004-0.154 (p36-75)	63.26	0.64	24.30	0.09	1.15E-05

**142**	prop_higher_edu <0.235 (p62) &prop_agricultural <0.046 (p79) &POI_pp_edu <15.157 (p67)	rate_imported12 =0.003-0.081 (p36-64)	45.45	0.62	24.24	0.11	5.46E-05
**143**	gender_ratio <95.000 (p64) &prop_gov_insti_faci >0.027 (p38) &POI_pp_mall_mkt <5.985 (p54)	rate_imported12 =0.004-0.123 (p36-71)	45.76	0.76	24.14	0.09	7.66E-05
**144**	prop_preprim_edu <0.126 (p81) &prop_higher_edu <0.237 (p62)	rate_imported12 =0.004-0.113 (p36-71)	56.25	0.60	24.14	0.16	3.72E-09
**145**	prop_preprim_edu <0.127 (p82) &med_income <18880.029 (p62)	rate_imported12 =0.003-0.111 (p36-71)	58.42	0.58	24.13	0.12	4.93E-07
**146**	prop_gov_insti_faci >0.027 (p38) &POI_pp_mall_mkt <9.043 (p66)	rate_imported12 =0.004-0.137 (p36-74)	60.60	0.62	24.12	0.12	5.29E-07
**147**	prop_higher_edu <0.246 (p64) &prop_agricultural <0.059 (p82) &POI_pp_telecom_elec <21.917 (p71)	rate_imported12 =0.004-0.082 (p36-65)	48.82	0.57	24.11	0.08	3.85E-05
**148**	prop_preprim_edu =0.080-0.138 (p19-85) &med_income <19052.086 (p63) &den_bldg =0.084-0.424 (p29-83)	rate_imported12 =0.003-0.116 (p36-71)	46.01	0.72	24.02	0.12	8.18E-05
**149**	prop_gov_insti_faci >0.027 (p38) &POI_pp_sports <27.608 (p65)	rate_imported12 =0.004-0.154 (p36-75)	62.60	0.64	23.99	0.10	3.79E-06
**150**	med_income <20560.672 (p73) &den_road >6.709 (p31) &POI_pp_sports <25.223 (p62)	rate_imported12 =0.004-0.076 (p36-64)	46.01	0.58	23.98	0.09	7.6E-05
**151**	prop_preprim_edu =0.079-0.135 (p18-84) &den_public_trans =6.497-83.636 (p34-91)	rate_imported12 =0.004-0.111 (p36-71)	53.88	0.61	23.95	0.11	1.32E-06
**152**	gender_ratio <93.624 (p52) &med_income <20108.868 (p73) &POI_pp_mall_mkt <5.988 (p54)	rate_imported12 =0.003-0.093 (p36-68)	40.84	0.75	23.95	0.08	2.75E-05
**153**	prop_gov_insti_faci >0.026 (p38) &POI_pp_edu <14.553 (p67)	rate_imported12 =0.003-0.139 (p36-74)	61.01	0.62	23.94	0.10	2.31E-06
**154**	den_population >15857.093 (p47) &prop_private_resid =0.009-0.201 (p26-83) &POI_pp_telecom_elec <12.219 (p51)	rate_imported12 =0.004-0.067 (p36-62)	36.62	0.71	23.92	0.14	5.21E-05
**155**	med_income <20846.023 (p73) &prop_private_resid =0.012-0.231 (p30-85) &POI_pp_mall_mkt <8.026 (p64)	rate_imported12 =0.005-0.078 (p36-64)	41.45	0.66	23.90	0.11	4.68E-05
**156**	build_area_pp <68.225 (p52) &POI_pp_edu <9.956 (p50)	rate_imported12 =0.004-0.083 (p36-65)	46.68	0.60	23.85	0.12	2.95E-05
**157**	med_income <18984.449 (p62) &den_public_trans =6.952-79.515 (p35-89)	rate_imported12 =0.004-0.090 (p36-67)	46.61	0.63	23.83	0.17	4.67E-08
**158**	prop_preprim_edu <0.130 (p83) &prop_higher_edu <0.246 (p64) &prop_business =0.001-0.047 (p33-86)	rate_imported12 =0.004-0.097 (p36-68)	44.68	0.68	23.83	0.07	6.34E-05
**159**	med_area_home <17.016 (p52) &prop_transport =0.051-0.322 (p31-86)	rate_imported12 =0.004-0.089 (p36-67)	46.21	0.63	23.81	0.11	7.91E-05

**160**	med_area_home <18.804 (p57) &POI_pp_sports <8.311 (p31)	rate_imported12 =0.002-0.086 (p36-66)	40.80	0.71	23.79	0.05	7.58E-05
**161**	prop_transport =0.069-0.334 (p36-87) &POI_pp_sports <12.803 (p42)	rate_imported12 =0.004-0.085 (p36-66)	42.08	0.69	23.79	0.13	6.3E-05
**162**	med_income <19892.439 (p65) &build_area_pp <63.409 (p51)	rate_imported12 =0.004-0.103 (p36-69)	51.91	0.61	23.76	0.08	8.2E-05
**163**	gender_ratio <93.932 (p56) &prop_higher_edu <0.314 (p77) &prop_gov_insti_faci >0.027 (p38)	rate_imported12 =0.003-0.094 (p36-68)	44.60	0.67	23.72	0.11	2.57E-05
**164**	prop_transport =0.070-0.336 (p36-88) &POI_pp_telecom_elec <12.219 (p51)	rate_imported12 =0.004-0.117 (p36-71)	48.55	0.68	23.68	0.12	8.13E-06
**165**	den_road =6.383-22.034 (p30-86) &POI_pp_transport <21.035 (p43)	rate_imported12 =0.004-0.081 (p36-64)	41.97	0.66	23.66	0.11	2.75E-05
**166**	prop_transport =0.075-0.346 (p36-90) &POI_pp_transport <20.897 (p43)	rate_imported12 =0.003-0.084 (p36-66)	41.60	0.68	23.64	0.13	2.27E-05
**167**	prop_higher_edu <0.253 (p65) &prop_transport =0.070-0.348 (p36-90) &POI_pp_telecom_elec <12.219 (p51)	rate_imported12 =0.004-0.094 (p36-68)	40.04	0.76	23.59	0.13	2.1E-05
**168**	med_area_home <17.155 (p52) &den_bldg =0.091-0.430 (p31-83)	rate_imported12 =0.004-0.072 (p36-64)	42.10	0.60	23.49	0.13	4.41E-05
**169**	build_area_pp <75.116 (p54) &prop_private_resid =0.006-0.239 (p22-85)	rate_imported12 =0.003-0.078 (p36-64)	45.27	0.58	23.49	0.14	1.33E-06
**170**	med_income <18973.034 (p62) &prop_agricultural <0.055 (p81) &LU_entropy >0.553 (p49)	rate_imported12 =0.004-0.081 (p36-64)	39.89	0.70	23.48	0.15	2.48E-06
**171**	med_income <19935.742 (p65) &prop_private_resid =0.010-0.230 (p27-85)	rate_imported12 =0.004-0.087 (p36-67)	47.91	0.59	23.47	0.17	8.22E-09
**172**	prop_preprim_edu <0.134 (p84) &LU_entropy >0.568 (p53) &POI_pp_telecom_elec <5.692 (p33)	rate_imported12 =0.003-0.089 (p36-67)	36.69	0.84	23.46	0.07	1.37E-05
**173**	med_area_home <16.459 (p49) &den_road =6.383-22.034 (p30-86)	rate_imported12 =0.004-0.081 (p36-64)	44.22	0.61	23.43	0.10	6.03E-05
**174**	med_area_home <15.536 (p45)	rate_imported12 =0.005-0.076 (p36-64)	50.15	0.52	23.36	0.24	1.29E-13
**175**	prop_gov_insti_faci >0.030 (p41) &POI_pp_telecom_elec <22.197 (p72)	rate_imported12 =0.003-0.136 (p36-73)	63.26	0.59	23.32	0.09	4.66E-05
**176**	med_income <18838.696 (p62) &prop_transport =0.051-0.322 (p31-86) &POI_pp_mall_mkt <5.231 (p51)	rate_imported12 =0.004-0.089 (p36-67)	39.43	0.74	23.29	0.12	4.43E-05
**177**	prop_higher_edu <0.232 (p62) &den_public_trans =6.952-79.515 (p35-89)	rate_imported12 =0.003-0.093 (p36-68)	44.77	0.65	23.28	0.18	3.04E-08

**178**	den_public_trans =6.952-79.515 (p35-89) &POI_pp_mall_mkt <4.404 (p47)	rate_imported12 =0.004-0.080 (p36-64)	42.51	0.63	23.23	0.14	1.51E-06
**179**	med_area_home <16.879 (p50) &LU_entropy >0.554 (p50)	rate_imported12 =0.003-0.078 (p36-64)	41.47	0.64	23.21	0.14	8E-05
**180**	den_population >15857.093 (p47) &prop_private_resid =0.009-0.201 (p26-83) &POI_pp_sports <21.567 (p56)	rate_imported12 =0.004-0.067 (p36-62)	36.14	0.69	23.21	0.14	4.19E-05
**181**	den_public_trans =10.237-71.472 (p38-86) &POI_pp_edu <12.318 (p57)	rate_imported12 =0.004-0.089 (p36-67)	44.80	0.63	23.19	0.15	1.83E-05
**182**	med_income <19447.658 (p63) &prop_business =0.001-0.054 (p32-86)	rate_imported12 =0.004-0.106 (p36-70)	49.52	0.62	23.07	0.14	3.5E-07
**183**	med_income <19079.165 (p63) &den_population =12488.876-93890.455 (p44-93)	rate_imported12 =0.004-0.093 (p36-68)	46.39	0.62	23.00	0.14	1.84E-06
**184**	LU_entropy >0.568 (p53) &POI_pp_telecom_elec <5.692 (p33)	rate_imported12 =0.004-0.072 (p36-64)	35.53	0.75	23.00	0.14	6.64E-05
**185**	den_population >16869.874 (p49) &prop_private_resid =0.012-0.231 (p30-85) &POI_pp_transport <17.102 (p38)	rate_imported12 =0.003-0.071 (p36-64)	33.75	0.82	23.00	0.12	1.06E-05
**186**	den_population =10945.965-84346.226 (p43-91) &POI_pp_mall_mkt <4.218 (p47)	rate_imported12 =0.004-0.068 (p36-62)	37.37	0.65	22.99	0.19	1.69E-05
**187**	den_road =6.383-22.034 (p30-86) &POI_pp_mall_mkt <3.900 (p45)	rate_imported12 =0.004-0.081 (p36-64)	41.71	0.64	22.99	0.13	1.13E-05
**188**	prop_preprim_edu <0.134 (p84) &med_area_home <16.409 (p49)	rate_imported12 =0.006-0.081 (p36-64)	47.17	0.52	22.95	0.05	7.38E-05
**189**	prop_business =0.001-0.047 (p29-86) &POI_pp_mall_mkt <9.030 (p66)	rate_imported12 =0.005-0.127 (p36-72)	57.92	0.60	22.95	0.10	1.38E-05
**190**	prop_preprim_edu =0.079-0.133 (p17-84) &LU_entropy >0.567 (p52) &POI_pp_sports <10.374 (p38)	rate_imported12 =0.005-0.084 (p36-66)	36.99	0.78	22.95	0.07	2.04E-05
**191**	prop_business =0.001-0.054 (p32-86) &POI_pp_edu <19.478 (p74)	rate_imported12 =0.003-0.141 (p36-74)	59.67	0.62	22.95	0.08	1.85E-05
**192**	prop_higher_edu <0.253 (p65) &prop_transport =0.070-0.348 (p36-90) &POI_pp_mall_mkt <5.865 (p53)	rate_imported12 =0.004-0.081 (p36-64)	38.05	0.72	22.93	0.13	9.95E-06
**193**	prop_higher_edu <0.248 (p64) &den_bldg =0.089-0.407 (p30-82) &prop_agricultural <0.053 (p81)	rate_imported12 =0.004-0.081 (p36-64)	40.77	0.65	22.83	0.09	5.66E-05
**194**	prop_higher_edu <0.255 (p65) &den_road >6.850 (p32) &POI_pp_edu <11.270 (p53)	rate_imported12 =0.004-0.077 (p36-64)	39.78	0.66	22.81	0.12	6.07E-05
**195**	prop_higher_edu <0.246 (p64) &prop_agricultural <0.055 (p81) &LU_entropy >0.553 (p49)	rate_imported12 =0.004-0.081 (p36-64)	39.01	0.69	22.81	0.14	9.56E-06

**196**	prop_transport =0.079-0.345 (p36-90) &POI_pp_mall_mkt <3.722 (p44)	rate_imported12 =0.002-0.090 (p36-67)	40.72	0.69	22.78	0.16	6.75E-06
**197**	med_area_home <19.336 (p61) &prop_business =0.001-0.054 (p32-86)	rate_imported12 =0.003-0.116 (p36-71)	51.98	0.62	22.74	0.12	2.09E-05
**198**	LU_entropy >0.567 (p52) &POI_pp_transport <13.768 (p33)	rate_imported12 =0.004-0.070 (p36-63)	35.03	0.74	22.74	0.11	4.46E-05
**199**	den_road =6.383-22.034 (p30-86) &POI_pp_sports <14.115 (p45)	rate_imported12 =0.004-0.071 (p36-64)	40.05	0.61	22.72	0.11	5.76E-05
**200**	prop_higher_edu <0.246 (p64) &den_bldg >0.098 (p33) &POI_pp_edu <12.697 (p59)	rate_imported12 =0.004-0.070 (p36-63)	38.65	0.62	22.70	0.13	7.05E-05
**201**	den_bldg =0.090-0.439 (p30-84) &POI_pp_telecom_elec <11.375 (p50)	rate_imported12 =0.004-0.070 (p36-64)	40.67	0.59	22.70	0.12	4.31E-05
**202**	gender_ratio <96.234 (p71) &prop_preprim_edu <0.130 (p83) &LU_entropy >0.565 (p52)	rate_imported12 =0.003-0.082 (p36-65)	41.72	0.63	22.65	0.10	7.92E-06
**203**	den_public_trans =10.237-71.472 (p38-86) &POI_pp_sports <16.304 (p50)	rate_imported12 =0.003-0.076 (p36-64)	40.85	0.62	22.60	0.14	4.32E-06
**204**	den_bldg =0.089-0.418 (p30-83) &POI_pp_mall_mkt <3.751 (p44)	rate_imported12 =0.004-0.072 (p36-64)	38.70	0.64	22.59	0.14	4.76E-05
**205**	den_population >17259.193 (p50) &POI_pp_transport <27.262 (p50)	rate_imported12 =0.003-0.075 (p36-64)	41.91	0.59	22.58	0.11	3.87E-05
**206**	build_area_pp <58.262 (p47) &prop_transport =0.078-0.342 (p36-89)	rate_imported12 =0.004-0.086 (p36-66)	41.65	0.65	22.58	0.14	1.42E-05
**207**	prop_preprim_edu =0.079-0.133 (p17-84) &build_area_pp <106.473 (p71) &LU_entropy >0.567 (p52)	rate_imported12 =0.003-0.099 (p36-68)	41.08	0.71	22.55	0.11	4.23E-05
**208**	den_bldg =0.086-0.380 (p29-81) &build_area_pp <62.960 (p50)	rate_imported12 =0.004-0.080 (p36-64)	40.78	0.64	22.52	0.15	1.38E-05
**209**	den_population >16592.806 (p49) &POI_pp_edu <12.477 (p58)	rate_imported12 =0.004-0.080 (p36-64)	44.74	0.58	22.51	0.13	2.88E-06
**210**	den_public_trans =10.237-71.472 (p38-86) &POI_pp_telecom_elec <11.661 (p50)	rate_imported12 =0.003-0.077 (p36-64)	41.33	0.61	22.51	0.13	4.29E-05
**211**	prop_higher_edu <0.233 (p62) &den_population =9845.500-83930.990 (p41-91)	rate_imported12 =0.003-0.106 (p36-70)	42.83	0.70	22.43	0.21	2.38E-08
**212**	med_area_home <19.931 (p65) &prop_gov_insti_faci >0.027 (p38)	rate_imported12 =0.004-0.108 (p36-70)	56.38	0.56	22.39	0.09	3.25E-05
**213**	med_income <21090.857 (p74) &POI_pp_mall_mkt <2.660 (p31)	rate_imported12 =0.004-0.095 (p36-68)	40.90	0.69	22.36	0.07	1.95E-05

**214**	med_area_home <18.228 (p55) &den_population =9467.160-80223.910 (p41-89)	rate_imported12 =0.004-0.107 (p36-70)	47.79	0.62	22.25	0.11	5.88E-05
**215**	den_population =13879.788-89721.699 (p45-93) &POI_pp_sports <16.482 (p50)	rate_imported12 =0.003-0.071 (p36-64)	38.38	0.62	22.23	0.16	6.63E-05
**216**	prop_transport =0.070-0.348 (p36-90) &POI_pp_edu <9.455 (p50)	rate_imported12 =0.003-0.088 (p36-67)	42.66	0.62	22.16	0.13	1.84E-05
**217**	med_income <20840.493 (p73) &POI_pp_edu <5.101 (p32)	rate_imported12 =0.003-0.082 (p36-65)	39.12	0.67	22.16	0.07	7.22E-05
**218**	den_population >17310.573 (p50) &LU_entropy >0.561 (p52)	rate_imported12 =0.003-0.085 (p36-66)	38.69	0.69	22.13	0.23	6.29E-08
**219**	gender_ratio <95.996 (p70) &den_population >15949.690 (p48)	rate_imported12 =0.003-0.145 (p36-74)	58.21	0.62	22.11	0.08	3.76E-05
**220**	prop_preprim_edu <0.134 (p84) &build_area_pp <73.363 (p54)	rate_imported12 =0.007-0.108 (p36-70)	56.12	0.54	22.08	0.05	7.56E-05
**221**	med_income <19664.302 (p65) &prop_gov_insti_faci >0.035 (p44)	rate_imported12 =0.003-0.085 (p36-66)	46.23	0.57	21.93	0.14	5.83E-07
**222**	gender_ratio <93.775 (p54) &build_area_pp <81.017 (p57)	rate_imported12 =0.004-0.083 (p36-66)	45.27	0.57	21.92	0.13	1.97E-05
**223**	den_road =6.383-22.034 (p30-86) &POI_pp_edu <9.176 (p50)	rate_imported12 =0.004-0.074 (p36-64)	40.37	0.59	21.90	0.13	3.64E-05
**224**	prop_preprim_edu <0.131 (p83) &prop_private_resid =0.004-0.220 (p21-84) &LU_entropy >0.557 (p50)	rate_imported12 =0.004-0.100 (p36-68)	45.10	0.63	21.88	0.07	4.64E-05
**225**	gender_ratio <96.234 (p71) &prop_preprim_edu <0.130 (p83)	rate_imported12 =0.004-0.158 (p36-76)	73.68	0.57	21.85	0.07	1.71E-06
**226**	gender_ratio <95.341 (p66) &med_income <18973.034 (p62)	rate_imported12 =0.004-0.099 (p36-68)	52.57	0.55	21.81	0.11	1.25E-05
**227**	med_income <18973.034 (p62) &prop_transport =0.061-0.335 (p35-87)	rate_imported12 =0.004-0.092 (p36-67)	46.01	0.59	21.79	0.16	9.77E-07
**228**	den_bldg =0.089-0.398 (p30-82) &POI_pp_edu <7.588 (p45)	rate_imported12 =0.004-0.070 (p36-64)	37.03	0.63	21.79	0.16	4.53E-05
**229**	prop_preprim_edu =0.083-0.131 (p23-83) &prop_agricultural <0.047 (p79) &POI_pp_mall_mkt <7.465 (p61)	rate_imported12 =0.005-0.083 (p36-65)	43.01	0.59	21.74	0.07	1.5E-05
**230**	prop_agricultural <0.047 (p79) &POI_pp_transport <39.835 (p61)	rate_imported12 =0.004-0.083 (p36-65)	53.15	0.50	21.73	0.05	6.01E-05
**231**	med_income <18949.418 (p62) &den_road =5.239-22.537 (p25-87)	rate_imported12 =0.003-0.109 (p36-70)	49.93	0.59	21.56	0.14	2.5E-07
**232**	prop_preprim_edu =0.085-0.127 (p25-82) &med_income <19433.607 (p63) &den_population =6277.989-86263.688 (p37-91)	rate_imported12 =0.005-0.105 (p36-69)	39.46	0.72	21.50	0.11	3.71E-05

**233**	gender_ratio <93.932 (p56) &prop_gov_insti_faci >0.027 (p38)	rate_imported12 =0.005-0.122 (p36-71)	52.09	0.60	21.44	0.10	1.17E-05
**234**	med_income <18814.295 (p62) &den_bldg =0.093-0.420 (p31-83)	rate_imported12 =0.004-0.084 (p36-66)	43.42	0.58	21.31	0.15	1.09E-05
**235**	prop_preprim_edu =0.072-0.129 (p11-82) &prop_gov_insti_faci >0.027 (p38) &LU_entropy >0.555 (p50)	rate_imported12 =0.003-0.127 (p36-72)	42.89	0.72	21.31	0.09	6.35E-05
**236**	POI_pp_sports <6.556 (p22)	rate_imported12 =0.004-0.108 (p36-70)	37.48	0.77	21.29	0.44	1.32E-13
**237**	den_population >16307.647 (p48) &LU_entropy >0.567 (p52) &POI_pp_edu <8.527 (p49)	rate_imported12 =0.005-0.064 (p36-60)	31.69	0.71	21.21	0.11	3.6E-05
**238**	prop_publicResid >0.034 (p69)	rate_imported12 =0.002-0.103 (p36-69)	43.49	0.63	21.17	0.30	1.54E-12
**239**	prop_higher_edu <0.258 (p65) &den_road =6.383-22.034 (p30-86)	rate_imported12 =0.003-0.097 (p36-68)	45.31	0.59	21.06	0.15	4.11E-07
**240**	prop_agricultural <0.046 (p79) &POI_pp_mall_mkt <7.099 (p60)	rate_imported12 =0.003-0.084 (p36-66)	50.81	0.51	21.06	0.07	7.55E-05
**241**	prop_higher_edu <0.214 (p55) &prop_private_resid =0.010-0.233 (p27-85)	rate_imported12 =0.002-0.106 (p36-69)	41.55	0.67	21.06	0.22	4.55E-07
**242**	gender_ratio <96.322 (p71) &prop_private_resid =0.010-0.230 (p27-85)	rate_imported12 =0.003-0.081 (p36-64)	49.53	0.50	21.03	0.09	2.44E-06
**243**	prop_agricultural <0.049 (p80) &POI_pp_edu <14.017 (p64)	rate_imported12 =0.002-0.083 (p36-65)	52.28	0.49	21.02	0.06	3.41E-05
**244**	prop_higher_edu <0.225 (p58) &prop_gov_insti_faci >0.027 (p38)	rate_imported12 =0.003-0.126 (p36-72)	47.45	0.65	21.01	0.17	2.43E-06
**245**	prop_preprim_edu =0.080-0.138 (p19-85) &den_bldg =0.084-0.424 (p29-83)	rate_imported12 =0.003-0.116 (p36-71)	50.31	0.59	20.96	0.13	6.3E-05
**246**	prop_higher_edu <0.260 (p65) &prop_business =0.003-0.064 (p43-87)	rate_imported12 =0.004-0.074 (p36-64)	38.50	0.59	20.94	0.17	1.63E-07
**247**	prop_preprim_edu =0.072-0.129 (p11-82) &prop_gov_insti_faci >0.027 (p38)	rate_imported12 =0.003-0.153 (p36-75)	61.07	0.60	20.93	0.09	2.57E-05
**248**	med_income <20533.112 (p73) &prop_agricultural <0.055 (p81)	rate_imported12 =0.004-0.083 (p36-65)	55.56	0.47	20.90	0.09	9.06E-07
**249**	prop_higher_edu <0.237 (p62) &POI_pp_telecom_elec <21.730 (p71)	rate_imported12 =0.005-0.097 (p36-68)	52.22	0.53	20.86	0.09	6.29E-05
**250**	med_income <19957.502 (p65) &den_bldg =0.096-0.353 (p32-79) &POI_pp_transport <33.445 (p56)	rate_imported12 =0.005-0.080 (p36-64)	35.84	0.68	20.85	0.10	2.6E-05
**251**	build_area_pp <51.859 (p43)	rate_imported12 =0.004-0.105 (p36-69)	50.34	0.56	20.82	0.23	9.92E-10
**252**	gender_ratio <93.712 (p53) &prop_higher_edu <0.326 (p79)	rate_imported12 =0.004-0.096 (p36-68)	50.54	0.54	20.76	0.07	7.49E-05

**253**	prop_business =0.003-0.044 (p43-85) &POI_pp_sports <27.447 (p64)	rate_imported12 =0.004-0.132 (p36-73)	47.48	0.65	20.64	0.10	4.62E-05
**254**	POI_pp_edu <3.533 (p20)	rate_imported12 =0.003-0.078 (p36-64)	32.83	0.75	20.52	0.47	8.59E-14
**255**	POI_pp_mall_mkt <1.922 (p22)	rate_imported12 =0.003-0.083 (p36-66)	33.73	0.73	20.19	0.44	8.37E-12
**256**	prop_preprim_edu =0.079-0.126 (p17-82) &prop_private_resid =0.009-0.218 (p26-84)	rate_imported12 =0.004-0.104 (p36-69)	45.83	0.59	20.16	0.13	1.39E-05
**257**	prop_preprim_edu =0.079-0.133 (p17-84) &LU_entropy >0.567 (p52)	rate_imported12 =0.003-0.138 (p36-74)	47.40	0.65	20.14	0.15	4.36E-06
**258**	build_area_pp <83.395 (p58) &LU_entropy >0.563 (p52)	rate_imported12 =0.003-0.084 (p36-66)	37.61	0.59	20.05	0.18	1.97E-05
**259**	med_income <19957.502 (p65) &den_population >15268.911 (p47) &den_bldg =0.096-0.353 (p32-79)	rate_imported12 =0.004-0.073 (p36-64)	33.90	0.66	20.01	0.11	2.6E-05
**260**	gender_ratio <93.717 (p53) &prop_open_recreation >0.027 (p46)	rate_imported12 =0.003-0.155 (p36-75)	47.66	0.68	19.83	0.14	9.56E-07
**261**	prop_preprim_edu =0.085-0.127 (p25-82) &den_population =6277.989-86263.688 (p37-91)	rate_imported12 =0.005-0.105 (p36-69)	42.92	0.61	19.78	0.14	1.02E-06
**262**	den_population >18336.564 (p50) &den_bldg =0.095-0.431 (p32-83)	rate_imported12 =0.004-0.070 (p36-64)	38.69	0.53	19.76	0.11	3.36E-05
**263**	prop_gov_insti_faci >0.027 (p38) &LU_entropy >0.555 (p50)	rate_imported12 =0.003-0.130 (p36-73)	46.75	0.63	19.73	0.15	2.87E-05
**264**	prop_higher_edu <0.238 (p63) &prop_open_recreation >0.027 (p46)	rate_imported12 =0.003-0.094 (p36-68)	42.00	0.59	19.61	0.16	6.45E-06
**265**	prop_publicResid >0.049 (p71) &POI_pp_mall_mkt <3.388 (p42)	rate_imported12 =0.002-0.058 (p36-57)	29.30	0.63	19.57	0.13	3.31E-05
**266**	prop_open_recreation >0.027 (p46) &POI_pp_transport <55.785 (p74)	rate_imported12 =0.003-0.107 (p36-70)	50.38	0.54	19.42	0.10	7.29E-05
**267**	prop_business =0.003-0.044 (p43-85) &LU_entropy >0.561 (p51)	rate_imported12 =0.002-0.138 (p36-74)	39.66	0.73	19.25	0.17	4.79E-05
**268**	prop_higher_edu <0.230 (p62) &den_bldg =0.099-0.391 (p33-82)	rate_imported12 =0.003-0.090 (p36-67)	38.82	0.61	19.20	0.16	2.73E-07
**269**	den_bldg =0.096-0.353 (p32-79) &POI_pp_sports <19.959 (p53)	rate_imported12 =0.005-0.080 (p36-64)	36.68	0.59	18.90	0.13	5.88E-05
**270**	prop_preprim_edu =0.086-0.134 (p26-84) &den_road =7.383-21.682 (p35-86)	rate_imported12 =0.004-0.090 (p36-67)	39.49	0.58	18.78	0.14	5.5E-06
**271**	prop_preprim_edu =0.078-0.131 (p17-83)	rate_imported12 =0.001-0.160 (p36-78)	75.11	0.54	18.76	0.13	6.1E-11
**272**	den_road =6.811-21.396 (p31-85) &LU_entropy >0.558 (p50)	rate_imported12 =0.004-0.079 (p36-64)	39.87	0.53	18.69	0.12	4.85E-05
**273**	prop_business =0.003-0.060 (p43-87)	rate_imported12 =0.004-0.182 (p36-79)	58.29	0.62	18.56	0.20	1.03E-07

**274**	prop_higher_edu <0.213 (p54) &prop_transport =0.079-0.345 (p36-90)	rate_imported12 =0.002-0.090 (p36-67)	36.41	0.62	18.52	0.18	6.23E-05
**275**	prop_higher_edu <0.231 (p62) &prop_grassland <0.082 (p71)	rate_imported12 =0.004-0.083 (p36-65)	42.39	0.51	18.29	0.12	5.55E-05
**276**	prop_private_resid =0.011-0.143 (p28-76) &prop_publicResid >0.063 (p74) &POI_pp_mall_mkt <3.731 (p44)	rate_imported12 =0.004-0.062 (p36-58)	24.62	0.78	17.48	0.12	7.96E-05
**277**	prop_private_resid =0.002-0.233 (p19-85)	rate_imported12 =0.005-0.182 (p36-79)	76.60	0.54	16.61	0.12	1.4E-07
**278**	den_public_trans =13.792-62.916 (p45-84)	rate_imported12 =0.004-0.140 (p36-74)	47.67	0.58	16.59	0.20	3.65E-07
**279**	gender_ratio <94.842 (p64)	rate_imported12 =0.003-0.116 (p36-71)	62.32	0.47	16.52	0.12	1.15E-06
**280**	prop_gov_insti_faci >0.030 (p41)	rate_imported12 =0.003-0.124 (p36-71)	62.21	0.49	16.38	0.13	3.9E-08
**281**	den_bldg =0.095-0.431 (p32-83)	rate_imported12 =0.003-0.160 (p36-78)	60.84	0.54	15.78	0.14	2.31E-05
**282**	den_population >26483.860 (p60)	rate_imported12 =0.004-0.084 (p36-66)	40.23	0.45	15.44	0.17	8.14E-08
**283**	den_population >17868.663 (p50) &prop_private_resid =0.020-0.115 (p35-70)	rate_imported12 =0.002-0.079 (p36-64)	26.40	0.67	15.18	0.22	2.38E-05
**284**	den_road =6.811-21.396 (p31-85)	rate_imported12 =0.004-0.079 (p36-64)	45.58	0.42	14.47	0.13	8.4E-07
**285**	LU_entropy >0.561 (p51)	rate_imported12 =0.001-0.099 (p36-68)	46.64	0.44	13.70	0.13	6.21E-05
**286**	prop_transport >0.121 (p50)	rate_imported12 =0.004-0.080 (p36-64)	43.70	0.40	12.51	0.11	4.34E-05
**287**	POI_pp_transport <4.899 (p9)	rate_imported12 =0.003-0.084 (p36-66)	18.09	0.89	12.07	0.59	1.59E-08
**288**	prop_agricultural <0.055 (p81)	rate_imported12 =0.003-0.082 (p36-65)	59.77	0.35	9.39	0.05	3.39E-05
**289**	POI_pp_transport =4.899-8.368 (p9-19)	rate_imported12 =0.003-0.084 (p36-66)	14.37	0.74	8.63	0.44	1.21E-05
**290**	prop_higher_edu >0.260 (p65) &prop_business =0.003-0.064 (p43-87)	rate_imported12 =0.074-0.221 (p64-81)	13.97	0.46	8.42	0.20	7.93E-05
**291**	med_area_home >18.804 (p57) &POI_pp_sports <8.311 (p31)	rate_imported12 =0.086-0.185 (p66-80)	2.84	0.50	2.05	0.34	4.54E-05
**292**	prop_higher_edu <0.278 (p71) &med_income <19467.587 (p63)	rate_imported12 <0.112 (p71)	119.46	0.91	26.89	0.03	3.67E-05
**293**	prop_higher_edu <0.253 (p65) &POI_pp_transport <39.932 (p61)	rate_imported12 <0.114 (p71)	90.29	0.94	22.56	0.06	2.23E-05
**294**	med_income <19583.009 (p65)	rate_imported12 <0.110 (p70)	120.22	0.85	22.51	0.16	1.22E-15
**295**	med_income <24851.593 (p79) &build_area_pp <77.706 (p56) &POI_pp_telecom_elec <18.818 (p67)	rate_imported12 <0.071 (p64)	79.53	0.86	21.92	0.04	3.57E-05
**296**	build_area_pp <59.805 (p50) &prop_agricultural <0.058 (p82) &POI_pp_telecom_elec <21.508 (p71)	rate_imported12 <0.079 (p64)	76.70	0.89	21.08	0.03	1.84E-05
**297**	build_area_pp <67.567 (p52) &prop_agricultural <0.055 (p81) &POI_pp_sports <31.736 (p69)	rate_imported12 <0.077 (p64)	76.26	0.88	21.01	0.04	2.06E-06

**298**	build_area_pp <70.858 (p53) &prop_agricultural <0.058 (p82) &POI_pp_transport <39.782 (p61)	rate_imported12 <0.079 (p64)	74.53	0.90	21.00	0.03	1.05E-05
**299**	med_income <24883.283 (p79) &build_area_pp <80.385 (p56) &prop_agricultural <0.040 (p77)	rate_imported12 <0.074 (p64)	79.49	0.86	20.66	0.06	4.57E-05
**300**	med_area_home <19.135 (p61) &build_area_pp <91.533 (p62) &POI_pp_transport <23.482 (p47)	rate_imported12 <0.084 (p66)	67.79	0.94	20.27	0.05	1.98E-06
**301**	med_income <25039.531 (p81) &den_population >15501.310 (p47) &build_area_pp <78.375 (p56)	rate_imported12 <0.079 (p64)	70.58	0.90	19.86	0.04	5.42E-05
**302**	prop_higher_edu <0.233 (p62) &prop_publicResid <0.005 (p56)	rate_imported12 <0.003 (p36)	39.78	0.71	19.48	0.25	2.68E-07
**303**	prop_higher_edu <0.255 (p65) &build_area_pp <87.354 (p62) &POI_pp_sports <14.086 (p45)	rate_imported12 <0.077 (p64)	59.91	0.93	19.31	0.05	2.38E-05
**304**	prop_over65 >0.133 (p29) &POI_pp_mall_mkt <10.180 (p72)	rate_imported12 <0.090 (p67)	93.01	0.84	19.28	0.10	5.97E-07
**305**	ave_household_size <3.155 (p84) &POI_pp_mall_mkt <6.852 (p59)	rate_imported12 <0.094 (p68)	93.10	0.85	18.75	0.05	6.87E-05
**306**	ave_household_size <3.207 (p87) &POI_pp_edu <11.974 (p55)	rate_imported12 <0.102 (p69)	92.38	0.86	18.40	0.05	4.74E-05
**307**	prop_over65 >0.130 (p26) &POI_pp_edu <14.477 (p66)	rate_imported12 <0.090 (p67)	87.67	0.84	18.25	0.08	2.45E-05
**308**	prop_preprim_edu >0.097 (p52) &prop_publicResid <0.005 (p56)	rate_imported12 <0.003 (p36)	39.13	0.67	18.20	0.18	2.35E-05
**309**	prop_over65 >0.130 (p26) &POI_pp_telecom_elec <18.764 (p66)	rate_imported12 <0.090 (p67)	89.24	0.84	18.08	0.08	8.13E-06
**310**	prop_preprim_edu >0.083 (p22) &ave_household_size <3.207 (p87)	rate_imported12 <0.102 (p69)	117.86	0.81	18.03	0.05	4.35E-05
**311**	prop_over65 >0.133 (p29) &POI_pp_transport <36.836 (p59)	rate_imported12 <0.090 (p67)	79.39	0.86	18.03	0.09	5.94E-06
**312**	prop_higher_edu <0.170 (p37)	rate_imported12 <0.039 (p50)	57.65	0.73	17.91	0.23	3.65E-07
**313**	ave_household_size <3.138 (p84) &build_area_pp <62.626 (p50)	rate_imported12 <0.114 (p71)	84.84	0.89	17.51	0.04	4.49E-05
**314**	prop_higher_edu <0.214 (p55) &prop_private_resid <0.010 (p27)	rate_imported12 <0.002 (p36)	34.06	0.74	17.48	0.10	8.1E-05
**315**	med_income <19935.742 (p65) &prop_private_resid <0.010 (p27)	rate_imported12 <0.004 (p36)	34.33	0.73	17.35	0.09	8.1E-05
**316**	med_income <20388.464 (p73) &build_area_pp >63.378 (p51) &prop_open_recreation <0.050 (p62)	rate_imported12 <0.003 (p36)	33.63	0.74	17.28	0.18	2.76E-06

**317**	ave_household_size <3.102 (p84) &POI_pp_sports <25.681 (p63)	rate_imported12 <0.113 (p71)	92.72	0.86	16.84	0.05	5.22E-05
**318**	build_area_pp <68.225 (p52) &prop_agricultural <0.063 (p82)	rate_imported12 <0.084 (p66)	82.98	0.80	16.71	0.03	1.17E-05
**319**	prop_higher_edu <0.233 (p62) &den_population <9845.500 (p41)	rate_imported12 <0.003 (p36)	36.21	0.66	16.54	0.18	4.44E-05
**320**	prop_over65 >0.142 (p36) &prop_private_resid <0.092 (p64) &prop_publicResid <0.005 (p56)	rate_imported12 <0.002 (p36)	30.62	0.78	16.48	0.21	2.11E-05
**321**	med_income <19664.302 (p65) &prop_gov_insti_faci <0.035 (p44)	rate_imported12 <0.003 (p36)	37.67	0.63	16.04	0.13	1.45E-05
**322**	prop_higher_edu <0.225 (p58) &prop_gov_insti_faci <0.027 (p38)	rate_imported12 <0.003 (p36)	35.07	0.66	16.03	0.15	2.5E-05
**323**	prop_private_resid <0.002 (p19)	rate_imported12 <0.005 (p36)	30.69	0.75	16.01	0.39	4.1E-09
**324**	prop_higher_edu <0.214 (p55) &build_area_pp >69.812 (p53) &prop_business <0.004 (p51)	rate_imported12 <0.004 (p36)	27.94	0.83	15.78	0.13	5.44E-05
**325**	prop_higher_edu <0.232 (p62) &den_public_trans <6.952 (p35)	rate_imported12 <0.003 (p36)	33.28	0.68	15.59	0.17	4.86E-05
**326**	med_income <19079.165 (p63) &den_population <12488.876 (p44)	rate_imported12 <0.004 (p36)	32.31	0.68	15.27	0.21	8.1E-05
**327**	prop_higher_edu <0.213 (p54) &prop_transport <0.079 (p36)	rate_imported12 <0.002 (p36)	31.98	0.68	15.14	0.20	4.72E-05
**328**	prop_over65 >0.140 (p35) &den_population <4756.972 (p34)	rate_imported12 <0.003 (p36)	29.97	0.72	14.96	0.18	4.73E-05
**329**	med_income <19957.502 (p65) &POI_pp_telecom_elec >12.854 (p53)	rate_imported12 <0.003 (p36)	36.45	0.61	14.93	0.17	7.26E-05
**330**	prop_higher_edu <0.212 (p54) &build_area_pp >63.378 (p51)	rate_imported12 <0.003 (p36)	32.02	0.67	14.89	0.20	2.11E-05
**331**	prop_business <0.003 (p43)	rate_imported12 <0.004 (p36)	47.88	0.52	14.64	0.16	1.82E-05
**332**	den_public_trans <3.592 (p25)	rate_imported12 <0.004 (p36)	33.98	0.63	14.57	0.27	1.77E-06
**333**	prop_higher_edu <0.230 (p62) &den_bldg <0.099 (p33)	rate_imported12 <0.003 (p36)	31.30	0.67	14.53	0.16	4.73E-05
**334**	prop_preprim_edu >0.131 (p83)	rate_imported12 <0.001 (p36)	28.77	0.73	14.52	0.37	3.28E-10
**335**	med_income <18984.449 (p62) &den_public_trans <6.952 (p35)	rate_imported12 <0.004 (p36)	28.98	0.71	14.34	0.20	7.88E-05
**336**	prop_over65 >0.144 (p36) &prop_transport <0.037 (p26)	rate_imported12 <0.002 (p36)	25.44	0.80	13.98	0.23	1.14E-05
**337**	med_income <19957.502 (p65) &POI_pp_transport >31.324 (p53)	rate_imported12 <0.004 (p36)	34.38	0.61	13.95	0.16	4E-05
**338**	med_income <18814.295 (p62) &den_bldg <0.093 (p31)	rate_imported12 <0.004 (p36)	26.44	0.75	13.80	0.23	5.83E-05
**339**	prop_over65 >0.143 (p36) &den_road <5.001 (p24)	rate_imported12 <0.003 (p36)	24.76	0.80	13.58	0.22	2.13E-05

**340**	med_income <18973.034 (p62) &prop_transport <0.061 (p35)	rate_imported12 <0.004 (p36)	26.83	0.72	13.36	0.21	7.55E-05
**341**	med_income <18949.418 (p62) &den_road <5.239 (p25)	rate_imported12 <0.003 (p36)	23.54	0.81	13.07	0.24	4.96E-05
**342**	prop_gov_insti_faci <0.030 (p41)	rate_imported12 <0.003 (p36)	43.91	0.51	12.80	0.15	7.14E-05
**343**	build_area_pp >71.028 (p53) &prop_rural_set >0.026 (p66)	rate_imported12 <0.004 (p36)	26.61	0.69	12.69	0.22	1.88E-05
**344**	prop_industrial >0.009 (p70)	rate_imported12 <0.097 (p68)	57.23	0.85	12.45	0.19	1.33E-05
**345**	ave_household_size <3.250 (p87)	rate_imported12 <0.110 (p70)	141.84	0.76	12.11	0.06	6.18E-09
**346**	prop_over65 >0.150 (p43) &den_road <14.014 (p63) &build_area_pp >81.017 (p57)	rate_imported12 <0.002 (p36)	22.99	0.76	12.09	0.21	4.05E-05
**347**	prop_over65 >0.140 (p35)	rate_imported12 <0.083 (p65)	100.87	0.72	11.69	0.08	5.07E-05
**348**	prop_publicResid <0.005 (p56) &prop_industrial >0.009 (p70)	rate_imported12 <0.007 (p36)	22.83	0.72	11.30	0.27	1.48E-05
**349**	den_bldg =0.082-0.432 (p28-83) &prop_agricultural <0.058 (p82) &POI_pp_mall_mkt <2.114 (p24)	rate_imported12 <0.079 (p64)	35.31	0.95	11.29	0.04	1.74E-05
**350**	prop_agricultural >0.055 (p81)	rate_imported12 <0.003 (p36)	25.97	0.63	11.25	0.27	2.23E-05
**351**	prop_higher_edu <0.237 (p62) &POI_pp_telecom_elec >21.730 (p71)	rate_imported12 <0.005 (p36)	22.09	0.72	11.00	0.26	7.34E-05
**352**	prop_transport <0.033 (p23)	rate_imported12 <0.004 (p36)	28.78	0.57	10.76	0.21	5.29E-05
**353**	prop_rural_set >0.069 (p80)	rate_imported12 <0.003 (p36)	26.33	0.60	10.61	0.24	4.17E-05
**354**	prop_preprim_edu >0.093 (p43) &den_bldg <0.205 (p57) &build_area_pp =68.225-212.874 (p52-90)	rate_imported12 <0.003 (p36)	20.36	0.72	10.19	0.18	2.77E-05
**355**	build_area_pp >63.378 (p51) &prop_industrial >0.009 (p70) &prop_open_recreation <0.050 (p62)	rate_imported12 <0.003 (p36)	18.96	0.77	10.12	0.19	3.2E-05
**356**	den_population <673.564 (p13)	rate_imported12 <0.004 (p36)	18.89	0.70	9.22	0.34	3.94E-06
**357**	med_income <17777.670 (p56) &med_area_home >19.469 (p61)	rate_imported12 <0.004 (p36)	15.83	0.79	8.63	0.35	5.62E-05
**358**	prop_industrial >0.009 (p70) &POI_pp_telecom_elec >18.233 (p65)	rate_imported12 <0.003 (p36)	15.02	0.80	8.28	0.35	6.7E-05
**359**	prop_industrial >0.008 (p70) &POI_pp_sports >28.416 (p66)	rate_imported12 <0.003 (p36)	14.99	0.79	8.19	0.35	8.21E-06

(c) Rules for wave-2 local case rate; POI accessibility was used

No.	Antecedent	Consequent	Supp	Conf	Lev	Imp	*P*
**1**	prop_higher_edu <0.224 (p58) &med_area_home <15.648 (p45) &POI_pp_telecom_elec <20.441 (p69)	rate_local3 >0.327 (p52)	56.26	0.78	22.07	0.06	3.013E-06
**2**	prop_higher_edu <0.224 (p58) &med_area_home <15.648 (p45) &POI_pp_transport <51.157 (p72)	rate_local3 >0.326 (p52)	56.20	0.78	22.04	0.06	1.984E-05
**3**	med_income <25645.373 (p81) &den_road >8.946 (p43)	rate_local3 >0.371 (p57)	62.10	0.66	21.68	0.14	2.24E-07
**4**	med_income <21576.614 (p75) &prop_transport >0.093 (p39)	rate_local3 >0.373 (p57)	60.96	0.66	21.64	0.14	4.513E-06
**5**	prop_higher_edu <0.224 (p58) &med_area_home <15.648 (p45) &POI_pp_mall_mkt <12.024 (p79)	rate_local3 >0.336 (p54)	54.81	0.76	21.50	0.05	2.288E-05
**6**	med_income <25727.745 (p81) &prop_shrubland <0.115 (p58)	rate_local3 >0.372 (p57)	63.91	0.64	21.37	0.12	3.952E-06
**7**	prop_preprim_edu <0.141 (p86) &prop_higher_edu <0.222 (p58) &POI_pp_telecom_elec <18.695 (p66)	rate_local3 >0.302 (p50)	60.34	0.77	21.29	0.05	5.614E-05
**8**	prop_higher_edu <0.224 (p58) &med_area_home <15.648 (p45) &POI_pp_edu <20.907 (p78)	rate_local3 >0.278 (p47)	59.04	0.83	21.18	0.06	1.283E-05
**9**	med_area_home <19.284 (p61) &prop_transport >0.092 (p39)	rate_local3 >0.373 (p57)	61.46	0.65	21.07	0.10	1.123E-05
**10**	med_area_home <19.574 (p62) &prop_shrubland <0.100 (p56)	rate_local3 >0.371 (p57)	56.70	0.68	20.89	0.13	3.136E-05
**11**	prop_preprim_edu <0.141 (p86) &prop_higher_edu <0.211 (p54) &med_area_home <19.828 (p65)	rate_local3 >0.342 (p54)	53.92	0.75	20.86	0.08	8.18E-05
**12**	prop_higher_edu <0.220 (p57) &med_area_home <15.420 (p45) &build_area_pp <140.600 (p81)	rate_local3 >0.292 (p50)	58.24	0.81	20.85	0.05	4.64E-05
**13**	prop_preprim_edu <0.147 (p89) &med_income <19861.505 (p65) &POI_pp_transport <57.236 (p76)	rate_local3 >0.248 (p42)	81.95	0.76	20.75	0.04	8.268E-05
**14**	med_income <16768.973 (p51) &den_population >15303.757 (p47)	rate_local3 >0.325 (p52)	54.81	0.76	20.73	0.13	4.975E-07
**15**	prop_preprim_edu <0.147 (p89) &med_income <19861.505 (p65) &POI_pp_edu <19.291 (p73)	rate_local3 >0.248 (p42)	80.00	0.77	20.68	0.04	3.734E-05
**16**	prop_preprim_edu <0.136 (p85) &prop_higher_edu <0.228 (p61) &POI_pp_transport <47.707 (p69)	rate_local3 >0.237 (p40)	68.15	0.84	20.57	0.07	3.119E-05
**17**	med_income <25645.373 (p81) &den_bldg >0.109 (p36)	rate_local3 >0.371 (p57)	67.02	0.62	20.42	0.12	5.626E-06
**18**	med_area_home <19.574 (p62) &den_road >8.244 (p39)	rate_local3 >0.371 (p57)	61.81	0.64	20.33	0.09	5.469E-05
**19**	med_area_home <19.574 (p62) &den_bldg >0.109 (p36)	rate_local3 >0.371 (p57)	63.25	0.63	20.33	0.08	2.446E-05
**20**	prop_higher_edu <0.226 (p60) &den_population >15303.757 (p47)	rate_local3 >0.335 (p53)	52.12	0.76	20.24	0.18	4.066E-07
**21**	med_income <23040.421 (p78) &prop_agricultural <0.028 (p72)	rate_local3 >0.335 (p53)	72.65	0.64	20.12	0.09	5.813E-05
**22**	prop_higher_edu <0.224 (p58) &med_area_home <15.648 (p45)	rate_local3 >0.336 (p54)	56.92	0.72	20.03	0.08	4.471E-05
**23**	prop_preprim_edu <0.142 (p87) &prop_higher_edu <0.223 (p58) &POI_pp_mall_mkt <10.971 (p75)	rate_local3 >0.243 (p41)	69.14	0.81	19.96	0.06	2.424E-07

**24**	med_area_home <19.516 (p61) &den_population >15303.757 (p47)	rate_local3 >0.325 (p52)	65.23	0.68	19.95	0.09	6.13E-05
**25**	prop_higher_edu <0.230 (p61) &prop_agricultural <0.024 (p71) &POI_pp_mall_mkt <11.049 (p75)	rate_local3 >0.272 (p47)	58.36	0.82	19.93	0.06	7.567E-05
**26**	prop_preprim_edu <0.141 (p86) &prop_higher_edu <0.219 (p56) &build_area_pp <79.707 (p56)	rate_local3 >0.279 (p48)	56.94	0.81	19.91	0.06	2.493E-05
**27**	prop_higher_edu <0.286 (p73) &prop_shrubland <0.107 (p57)	rate_local3 >0.365 (p56)	56.42	0.67	19.86	0.14	1.708E-05
**28**	prop_higher_edu <0.230 (p61) &prop_transport >0.101 (p43)	rate_local3 >0.373 (p57)	48.63	0.72	19.79	0.19	7.686E-07
**29**	prop_higher_edu <0.223 (p58) &med_area_home <16.151 (p47) &POI_pp_sports <33.215 (p70)	rate_local3 >0.243 (p41)	61.23	0.85	19.70	0.06	6.768E-05
**30**	prop_preprim_edu <0.141 (p86) &med_income <16682.171 (p51)	rate_local3 >0.282 (p49)	65.86	0.75	19.57	0.08	5.409E-05
**31**	prop_preprim_edu <0.141 (p86) &med_area_home <19.828 (p65)	rate_local3 >0.249 (p43)	89.09	0.73	19.56	0.05	1.799E-05
**32**	prop_preprim_edu <0.152 (p91) &med_income <18928.552 (p62) &POI_pp_mall_mkt <11.049 (p75)	rate_local3 >0.177 (p33)	85.95	0.87	19.55	0.06	3.991E-06
**33**	prop_higher_edu <0.229 (p61) &build_area_pp <110.047 (p72) &prop_shrubland <0.214 (p82)	rate_local3 >0.231 (p39)	67.64	0.83	19.48	0.07	5.576E-05
**34**	prop_preprim_edu <0.145 (p89) &med_income <18958.166 (p62) &POI_pp_telecom_elec <19.083 (p67)	rate_local3 >0.193 (p35)	79.18	0.86	19.47	0.04	4.028E-05
**35**	prop_higher_edu <0.224 (p58) &prop_agricultural <0.025 (p71) &POI_pp_transport <47.989 (p69)	rate_local3 >0.330 (p52)	50.17	0.77	19.41	0.07	5.417E-05
**36**	prop_higher_edu <0.227 (p60) &build_area_pp <64.139 (p51)	rate_local3 >0.314 (p52)	56.43	0.74	19.37	0.12	3.369E-05
**37**	prop_higher_edu <0.249 (p64) &den_road >9.024 (p43)	rate_local3 >0.349 (p55)	50.61	0.73	19.32	0.18	4.782E-06
**38**	prop_preprim_edu <0.128 (p82) &prop_higher_edu <0.223 (p58) &POI_pp_sports <33.215 (p70)	rate_local3 >0.243 (p41)	63.66	0.83	19.32	0.07	2.923E-05
**39**	prop_higher_edu <0.230 (p61) &prop_agricultural <0.024 (p71)	rate_local3 >0.335 (p53)	57.60	0.70	19.22	0.12	4.05E-05
**40**	prop_preprim_edu <0.146 (p89) &med_income <17876.257 (p56) &POI_pp_sports <35.406 (p73)	rate_local3 >0.213 (p37)	73.44	0.84	19.19	0.05	2.41E-05
**41**	prop_over65 >0.140 (p35) &prop_preprim_edu <0.130 (p83) &POI_pp_edu <21.935 (p79)	rate_local3 >0.232 (p39)	71.95	0.80	19.01	0.07	3.787E-05
**42**	gender_ratio <103.139 (p89) &med_income <18928.552 (p62) &POI_pp_mall_mkt <11.049 (p75)	rate_local3 >0.212 (p37)	83.49	0.80	18.91	0.03	6.313E-05
**43**	prop_preprim_edu <0.138 (p85) &prop_higher_edu <0.229 (p61) &POI_pp_edu <20.842 (p78)	rate_local3 >0.237 (p40)	68.67	0.81	18.80	0.05	3.264E-05
**44**	gender_ratio <103.298 (p89) &prop_higher_edu <0.220 (p57) &build_area_pp <140.600 (p81)	rate_local3 >0.245 (p42)	74.13	0.77	18.71	0.04	4.62E-05
**45**	prop_higher_edu <0.229 (p61) &den_bldg >0.109 (p36)	rate_local3 >0.348 (p55)	53.95	0.69	18.70	0.13	4.571E-05

**46**	prop_over65 >0.131 (p27) &prop_preprim_edu <0.127 (p82) &prop_higher_edu <0.342 (p81)	rate_local3 >0.231 (p39)	77.77	0.78	18.64	0.07	3.768E-05
**47**	gender_ratio <103.088 (p89) &med_income <17032.612 (p53) &POI_pp_telecom_elec <22.637 (p72)	rate_local3 >0.209 (p36)	74.44	0.84	18.59	0.03	5.347E-05
**48**	gender_ratio <104.750 (p89) &prop_higher_edu <0.246 (p64) &POI_pp_telecom_elec <19.940 (p68)	rate_local3 >0.183 (p33)	83.18	0.85	18.53	0.03	5.575E-05
**49**	prop_preprim_edu <0.130 (p82) &med_area_home <18.519 (p56) &POI_pp_telecom_elec <19.940 (p68)	rate_local3 >0.183 (p33)	79.23	0.87	18.51	0.04	2.659E-05
**50**	prop_over65 >0.141 (p36) &prop_preprim_edu <0.127 (p82) &POI_pp_telecom_elec <22.172 (p72)	rate_local3 >0.229 (p39)	69.42	0.81	18.49	0.06	1.946E-05
**51**	med_income <25547.911 (p81) &prop_grassland <0.042 (p60)	rate_local3 >0.374 (p57)	60.37	0.61	18.44	0.11	6.293E-05
**52**	gender_ratio <103.088 (p89) &med_income <17032.612 (p53) &POI_pp_transport <45.672 (p69)	rate_local3 >0.222 (p38)	69.45	0.83	18.42	0.03	4.377E-05
**53**	prop_over65 >0.140 (p35) &prop_preprim_edu <0.134 (p84) &POI_pp_transport <51.240 (p72)	rate_local3 >0.232 (p39)	69.61	0.81	18.40	0.07	3.712E-05
**54**	med_income <16608.038 (p51) &build_area_pp <69.587 (p52)	rate_local3 >0.291 (p50)	54.70	0.77	18.38	0.11	8.044E-05
**55**	prop_preprim_edu <0.152 (p91) &med_area_home <18.819 (p57) &POI_pp_mall_mkt <11.049 (p75)	rate_local3 >0.176 (p33)	84.04	0.86	18.32	0.04	4.332E-05
**56**	prop_preprim_edu <0.136 (p85) &med_area_home <18.935 (p58) &POI_pp_edu <20.842 (p78)	rate_local3 >0.193 (p35)	79.68	0.84	18.31	0.03	2.625E-05
**57**	med_income <17081.439 (p53) &prop_open_recreation >0.026 (p45)	rate_local3 >0.352 (p56)	49.42	0.71	18.23	0.11	1.034E-05
**58**	gender_ratio <104.715 (p89) &med_income <17876.257 (p56) &POI_pp_sports <35.406 (p73)	rate_local3 >0.213 (p37)	76.57	0.81	18.15	0.03	6.524E-05
**59**	med_income <25469.001 (p81) &prop_woodland <0.212 (p68)	rate_local3 >0.375 (p57)	68.42	0.58	18.00	0.07	6.138E-05
**60**	med_income <19165.125 (p63) &POI_pp_telecom_elec <21.733 (p71)	rate_local3 >0.193 (p35)	88.51	0.81	17.80	0.08	5.322E-06
**61**	med_area_home <18.519 (p56) &prop_grassland <0.072 (p69)	rate_local3 >0.343 (p54)	59.44	0.65	17.78	0.07	7.172E-05
**62**	gender_ratio <104.750 (p89) &med_area_home <18.519 (p56) &POI_pp_telecom_elec <19.940 (p68)	rate_local3 >0.183 (p33)	83.94	0.84	17.74	0.02	4.582E-05
**63**	gender_ratio <103.088 (p89) &med_income <17032.612 (p53) &POI_pp_edu <17.348 (p70)	rate_local3 >0.207 (p36)	70.23	0.84	17.66	0.04	5.708E-05
**64**	med_income <16756.602 (p51) &med_area_home <15.534 (p45) &build_area_pp <92.916 (p63)	rate_local3 >0.227 (p38)	59.47	0.85	17.60	0.05	8.176E-05
**65**	prop_preprim_edu <0.141 (p86) &prop_higher_edu <0.215 (p56)	rate_local3 >0.279 (p48)	65.93	0.72	17.41	0.08	6.447E-05
**66**	med_income <19165.125 (p63) &POI_pp_transport <47.707 (p69)	rate_local3 >0.193 (p35)	87.15	0.81	17.30	0.07	1.306E-05

**67**	prop_higher_edu <0.222 (p58) &prop_open_recreation >0.026 (p45)	rate_local3 >0.334 (p53)	49.02	0.72	17.29	0.14	1.14E-05
**68**	prop_higher_edu <0.224 (p58) &prop_gov_insti_faci >0.027 (p38)	rate_local3 >0.297 (p50)	54.45	0.74	17.12	0.12	6.168E-05
**69**	med_area_home <15.986 (p47) &build_area_pp <74.856 (p54)	rate_local3 >0.192 (p35)	69.97	0.86	16.98	0.08	4.884E-05
**70**	prop_over65 >0.141 (p36) &prop_preprim_edu <0.127 (p82) &POI_pp_mall_mkt <11.292 (p75)	rate_local3 >0.229 (p39)	65.31	0.81	16.93	0.07	3.072E-05
**71**	med_income <22654.708 (p78) &prop_gov_insti_faci >0.026 (p38)	rate_local3 >0.199 (p36)	82.41	0.81	16.92	0.09	6.82E-05
**72**	med_area_home <13.826 (p27)	rate_local3 >0.340 (p54)	43.20	0.76	16.91	0.30	1.257E-08
**73**	med_area_home <19.925 (p65) &prop_gov_insti_faci >0.027 (p38)	rate_local3 >0.188 (p34)	83.83	0.82	16.89	0.07	5.142E-05
**74**	gender_ratio <102.576 (p88) &med_area_home <20.274 (p67) &POI_pp_edu <17.820 (p71)	rate_local3 >0.172 (p31)	89.18	0.84	16.82	0.03	4.33E-05
**75**	med_income <15406.084 (p39)	rate_local3 >0.335 (p53)	57.08	0.66	16.75	0.19	1.492E-06
**76**	prop_higher_edu <0.228 (p61) &POI_pp_transport <47.707 (p69)	rate_local3 >0.193 (p35)	80.16	0.82	16.56	0.09	1.747E-05
**77**	gender_ratio <100.809 (p84) &prop_higher_edu <0.249 (p64) &POI_pp_sports <28.870 (p66)	rate_local3 >0.174 (p32)	77.27	0.86	16.55	0.04	5.409E-05
**78**	prop_preprim_edu <0.153 (p91) &med_area_home <16.836 (p50) &POI_pp_sports <29.915 (p67)	rate_local3 >0.165 (p29)	75.35	0.88	16.53	0.03	7.482E-05
**79**	med_area_home <15.159 (p43) &POI_pp_transport <51.240 (p72)	rate_local3 >0.236 (p40)	62.92	0.80	16.49	0.06	6.56E-05
**80**	ave_household_size <2.962 (p63) &prop_shrubland <0.134 (p64)	rate_local3 >0.452 (p64)	50.06	0.53	16.42	0.09	8.294E-05
**81**	prop_over65 >0.141 (p36) &prop_preprim_edu <0.127 (p82) &POI_pp_sports <31.916 (p69)	rate_local3 >0.229 (p39)	61.99	0.81	16.31	0.07	3.072E-05
**82**	med_area_home <15.038 (p43) &POI_pp_telecom_elec <20.031 (p68)	rate_local3 >0.214 (p37)	65.46	0.82	16.28	0.06	1.97E-05
**83**	med_area_home <15.648 (p45) &POI_pp_edu <20.907 (p78)	rate_local3 >0.193 (p35)	72.17	0.84	16.25	0.05	5.236E-05
**84**	prop_higher_edu <0.222 (p58) &POI_pp_sports <12.643 (p42)	rate_local3 >0.277 (p47)	49.14	0.79	16.23	0.13	7.736E-05
**85**	prop_higher_edu <0.224 (p58) &prop_agricultural <0.023 (p70) &POI_pp_edu <21.158 (p78)	rate_local3 >0.239 (p41)	54.93	0.83	16.17	0.05	6.419E-05
**86**	gender_ratio <104.715 (p89) &med_income <17876.257 (p56)	rate_local3 >0.223 (p38)	85.92	0.74	16.07	0.03	6.418E-05
**87**	prop_preprim_edu <0.140 (p86) &POI_pp_transport <21.248 (p43)	rate_local3 >0.182 (p33)	74.09	0.85	16.06	0.03	3.366E-05
**88**	prop_over65 >0.141 (p36) &POI_pp_telecom_elec <22.172 (p72)	rate_local3 >0.256 (p44)	73.69	0.72	16.04	0.08	3.485E-05
**89**	gender_ratio <103.391 (p89) &med_area_home <17.921 (p55) &POI_pp_mall_mkt <13.167 (p81)	rate_local3 >0.150 (p27)	85.93	0.87	15.93	0.02	7.275E-05
**90**	build_area_pp <61.517 (p50) &POI_pp_mall_mkt <13.124 (p81)	rate_local3 >0.177 (p33)	81.49	0.84	15.88	0.02	8.324E-06
**91**	med_income <19861.505 (p65) &POI_pp_edu <19.291 (p73)	rate_local3 >0.180 (p33)	94.43	0.80	15.87	0.06	2.841E-05
**92**	ave_household_size <2.900 (p49) &den_bldg >0.126 (p40)	rate_local3 >0.408 (p59)	48.67	0.58	15.72	0.09	3.968E-05

**93**	prop_over65 >0.120 (p19) &gender_ratio <102.576 (p88) &POI_pp_edu <17.820 (p71)	rate_local3 >0.172 (p31)	93.84	0.82	15.71	0.03	8.049E-05
**94**	ave_household_size <2.900 (p49) &den_road >10.099 (p47)	rate_local3 >0.408 (p59)	44.42	0.61	15.70	0.12	4.951E-05
**95**	med_area_home <15.626 (p45) &POI_pp_mall_mkt <13.167 (p81)	rate_local3 >0.188 (p34)	73.17	0.84	15.70	0.05	7.213E-05
**96**	prop_household_3gen <0.081 (p92) &med_area_home <16.469 (p49)	rate_local3 >0.179 (p33)	81.22	0.83	15.69	0.04	3.328E-06
**97**	prop_higher_edu <0.229 (p61) &prop_grassland <0.043 (p60)	rate_local3 >0.311 (p51)	48.88	0.72	15.68	0.13	5.346E-05
**98**	gender_ratio <103.076 (p89) &prop_preprim_edu <0.153 (p91) &POI_pp_sports <15.701 (p50)	rate_local3 >0.142 (p26)	83.74	0.88	15.62	0.01	4.391E-05
**99**	prop_higher_edu <0.228 (p61) &POI_pp_mall_mkt <9.663 (p69)	rate_local3 >0.233 (p39)	75.07	0.74	15.46	0.06	4.663E-05
**100**	prop_preprim_edu <0.126 (p82) &POI_pp_edu <7.095 (p44)	rate_local3 >0.190 (p35)	68.96	0.84	15.42	0.05	3.375E-05
**101**	gender_ratio <103.088 (p89) &POI_pp_transport <23.063 (p45)	rate_local3 >0.137 (p26)	84.11	0.88	15.23	0.02	1.123E-05
**102**	prop_preprim_edu <0.147 (p90) &POI_pp_telecom_elec <6.955 (p37)	rate_local3 >0.193 (p35)	64.76	0.85	15.17	0.02	3.01E-05
**103**	med_area_home <15.986 (p47) &POI_pp_sports <33.639 (p70)	rate_local3 >0.176 (p33)	72.35	0.85	15.15	0.06	1.022E-05
**104**	med_income <18723.236 (p62) &prop_rural_set <0.000 (p44) &prop_industrial <0.008 (p69)	rate_local3 >0.428 (p63)	29.28	0.77	15.05	0.15	6.342E-05
**105**	prop_over65 >0.140 (p35) &POI_pp_transport <51.240 (p72)	rate_local3 >0.232 (p39)	76.83	0.74	15.00	0.08	6.242E-05
**106**	prop_preprim_edu <0.140 (p86) &POI_pp_sports <18.293 (p51)	rate_local3 >0.132 (p25)	88.59	0.88	14.99	0.03	7.173E-05
**107**	prop_higher_edu <0.371 (p86) &POI_pp_telecom_elec <6.955 (p37)	rate_local3 >0.193 (p35)	64.47	0.84	14.97	0.02	3.01E-05
**108**	prop_over65 >0.147 (p39) &prop_preprim_edu =0.092-0.132 (p39-84)	rate_local3 >0.245 (p42)	52.23	0.80	14.89	0.11	1.027E-05
**109**	prop_preprim_edu <0.142 (p87) &med_area_home <13.845 (p27) &POI_pp_transport <55.590 (p74)	rate_local3 >0.231 (p39)	45.49	0.88	14.88	0.02	3.838E-05
**110**	gender_ratio <102.821 (p88) &POI_pp_edu <18.657 (p73)	rate_local3 >0.127 (p25)	119.39	0.84	14.88	0.03	4.211E-05
**111**	med_income <18958.166 (p62) &POI_pp_sports <35.685 (p73)	rate_local3 >0.193 (p35)	81.29	0.79	14.86	0.06	6.207E-05
**112**	prop_preprim_edu =0.097-0.150 (p49-90) &den_population >15303.757 (p47)	rate_local3 >0.343 (p54)	37.23	0.76	14.82	0.15	2.737E-06
**113**	gender_ratio <103.088 (p89) &POI_pp_sports <16.105 (p50)	rate_local3 >0.138 (p26)	86.02	0.87	14.76	0.02	1.346E-05
**114**	med_area_home <18.502 (p56) &prop_woodland <0.075 (p39)	rate_local3 >0.361 (p56)	38.79	0.70	14.65	0.13	6.515E-05
**115**	prop_preprim_edu <0.143 (p89) &POI_pp_mall_mkt <7.036 (p60)	rate_local3 >0.142 (p26)	95.53	0.85	14.62	0.04	3.793E-05
**116**	gender_ratio <102.650 (p88) &med_area_home <18.935 (p58)	rate_local3 >0.163 (p29)	94.86	0.82	14.49	0.03	3.809E-05
**117**	POI_pp_transport <16.809 (p38)	rate_local3 >0.191 (p35)	65.98	0.84	14.48	0.18	1.673E-07
**118**	build_area_pp <61.380 (p50)	rate_local3 >0.222 (p38)	75.64	0.75	14.46	0.14	6.155E-06
**119**	build_area_pp <77.922 (p56) &prop_grassland <0.070 (p67)	rate_local3 >0.159 (p29)	74.86	0.86	14.37	0.06	1.771E-05

**120**	med_income <18357.304 (p61) &POI_pp_mall_mkt <11.049 (p75)	rate_local3 >0.177 (p33)	85.03	0.81	14.24	0.05	5.201E-05
**121**	prop_higher_edu <0.237 (p62)	rate_local3 >0.335 (p53)	75.42	0.57	14.15	0.11	7.001E-06
**122**	gender_ratio <98.034 (p78) &POI_pp_mall_mkt <4.803 (p49)	rate_local3 >0.129 (p25)	78.44	0.89	14.05	0.05	6.672E-05
**123**	den_population >21684.808 (p53)	rate_local3 >0.267 (p47)	68.18	0.69	14.02	0.14	3.312E-05
**124**	prop_higher_edu <0.244 (p63) &POI_pp_edu <21.332 (p79)	rate_local3 >0.203 (p36)	84.69	0.76	14.01	0.05	8.162E-05
**125**	ave_household_size <2.659 (p29) &prop_transport >0.098 (p43)	rate_local3 >0.475 (p66)	29.62	0.63	13.98	0.15	3.579E-05
**126**	prop_transport >0.141 (p55)	rate_local3 >0.382 (p57)	54.56	0.56	13.89	0.14	4.175E-05
**127**	POI_pp_telecom_elec <6.955 (p37)	rate_local3 >0.193 (p35)	65.22	0.82	13.81	0.17	2.008E-06
**128**	ave_household_size <2.733 (p37) &den_population >20736.122 (p52)	rate_local3 >0.361 (p56)	32.65	0.76	13.71	0.18	7.586E-05
**129**	gender_ratio <103.088 (p89) &POI_pp_telecom_elec <24.978 (p76)	rate_local3 >0.130 (p25)	124.27	0.82	13.65	0.02	5.276E-05
**130**	prop_higher_edu <0.222 (p58) &prop_open_recreation >0.026 (p45) &POI_pp_telecom_elec <32.319 (p80)	rate_local3 >0.185 (p33)	55.34	0.88	13.63	0.05	5.976E-05
**131**	prop_higher_edu <0.222 (p58) &prop_open_recreation >0.026 (p45) &POI_pp_sports <37.567 (p75)	rate_local3 >0.185 (p33)	53.89	0.88	13.51	0.05	5.238E-05
**132**	ave_household_size <2.636 (p29) &prop_rural_set <0.001 (p46)	rate_local3 >0.493 (p68)	25.71	0.65	13.15	0.17	5.449E-05
**133**	prop_grassland <0.070 (p67) &POI_pp_telecom_elec <11.261 (p50)	rate_local3 >0.156 (p28)	64.11	0.87	12.57	0.05	7.501E-05
**134**	prop_publicResid >0.063 (p74)	rate_local3 >0.295 (p50)	40.41	0.74	12.49	0.23	6.569E-05
**135**	prop_shrubland <0.009 (p30)	rate_local3 >0.570 (p76)	28.29	0.43	12.25	0.19	1.275E-05
**136**	prop_woodland <0.012 (p22)	rate_local3 >0.409 (p59)	30.91	0.65	12.19	0.26	1.005E-05
**137**	prop_preprim_edu =0.097-0.143 (p51-89)	rate_local3 >0.313 (p52)	50.96	0.64	11.51	0.14	7.439E-06
**138**	ave_household_size <2.627 (p29) &prop_gov_insti_faci >0.026 (p38)	rate_local3 >0.453 (p64)	24.61	0.65	11.34	0.15	2.563E-05
**139**	prop_grassland <0.000 (p16)	rate_local3 >0.500 (p68)	21.99	0.64	11.34	0.33	1.079E-05
**140**	den_road >9.024 (p43)	rate_local3 >0.407 (p59)	59.85	0.49	11.28	0.09	5.277E-05
**141**	prop_preprim_edu <0.126 (p82) &POI_pp_sports <22.459 (p57) &POI_pp_telecom_elec <4.154 (p22)	rate_local3 >0.192 (p35)	39.39	0.89	10.70	0.02	3.007E-05
**142**	prop_preprim_edu <0.126 (p82) &POI_pp_telecom_elec <4.154 (p22) &POI_pp_mall_mkt <10.155 (p71)	rate_local3 >0.192 (p35)	39.39	0.89	10.70	0.02	2.927E-05
**143**	prop_preprim_edu <0.126 (p82) &POI_pp_edu <23.861 (p81) &POI_pp_telecom_elec <4.154 (p22)	rate_local3 >0.192 (p35)	39.39	0.89	10.70	0.02	2.474E-05
**144**	prop_preprim_edu <0.126 (p82) &POI_pp_telecom_elec <4.154 (p22) &POI_pp_transport <80.155 (p83)	rate_local3 >0.192 (p35)	39.39	0.89	10.70	0.02	1.321E-05
**145**	prop_over65 >0.141 (p36)	rate_local3 >0.274 (p47)	85.51	0.61	10.65	0.08	8.153E-05

**146**	den_bldg >0.136 (p43)	rate_local3 >0.411 (p59)	59.27	0.47	10.35	0.08	8.318E-05
**147**	prop_preprim_edu <0.142 (p86) &ave_household_size <2.615 (p29)	rate_local3 >0.453 (p64)	25.52	0.59	10.28	0.07	6.777E-05
**148**	prop_higher_edu <0.212 (p54) &prop_private_resid >0.104 (p66)	rate_local3 >0.372 (p57)	17.56	0.94	9.55	0.39	3.173E-05
**149**	ave_household_size <2.580 (p17)	rate_local3 >0.621 (p79)	18.74	0.43	9.54	0.22	2.656E-05
**150**	prop_business >0.039 (p84)	rate_local3 >0.518 (p71)	19.10	0.54	8.80	0.25	2.866E-05
**151**	POI_pp_mall_mkt <2.023 (p22)	rate_local3 =0.193-0.721 (p35-85)	39.01	0.80	15.66	0.32	5.067E-08
**152**	POI_pp_edu <4.431 (p27)	rate_local3 =0.197-0.692 (p36-83)	41.95	0.72	14.71	0.25	1.587E-08
**153**	POI_pp_sports <5.899 (p20)	rate_local3 =0.184-0.660 (p33-80)	34.14	0.80	14.59	0.34	7.999E-08
**154**	prop_higher_edu >0.228 (p61) &POI_pp_transport <47.707 (p69)	rate_local3 =0.043-0.193 (p16-35)	21.31	0.43	12.16	0.14	2.852E-05
**155**	med_income >19165.125 (p63) &POI_pp_telecom_elec <21.733 (p71)	rate_local3 =0.043-0.193 (p16-35)	19.20	0.45	11.25	0.14	6.091E-06
**156**	med_income >19165.125 (p63) &POI_pp_transport <47.707 (p69)	rate_local3 =0.043-0.193 (p16-35)	17.83	0.45	10.53	0.15	1.161E-05
**157**	med_income >18958.166 (p62) &POI_pp_sports <35.685 (p73)	rate_local3 =0.043-0.193 (p16-35)	17.69	0.39	9.24	0.09	1.777E-05
**158**	med_area_home >15.986 (p47) &POI_pp_sports <33.639 (p70)	rate_local3 =0.030-0.176 (p16-33)	20.05	0.30	9.16	0.10	5.818E-05
**159**	POI_pp_telecom_elec =6.955-20.167 (p37-68)	rate_local3 =0.043-0.193 (p16-35)	20.75	0.32	8.72	0.13	5.217E-06
**160**	POI_pp_transport =16.809-55.037 (p38-74)	rate_local3 =0.041-0.191 (p16-35)	21.80	0.29	7.99	0.11	3.919E-07
**161**	prop_higher_edu >0.371 (p86) &POI_pp_telecom_elec =6.955-20.167 (p37-68)	rate_local3 =0.043-0.193 (p16-35)	7.89	0.78	6.01	0.37	6.132E-05
**162**	prop_publicResid <0.028 (p66) &prop_transport <0.226 (p69)	rate_local3 <0.354 (p56)	74.65	0.75	19.20	0.10	2.527E-05
**163**	prop_publicResid <0.024 (p66) &prop_woodland >0.072 (p38)	rate_local3 <0.329 (p52)	62.51	0.75	18.13	0.13	6.149E-05
**164**	prop_publicResid <0.027 (p66) &prop_business <0.041 (p85)	rate_local3 <0.345 (p55)	79.04	0.71	18.13	0.07	7.714E-05
**165**	prop_higher_edu >0.225 (p58) &prop_business <0.041 (p85)	rate_local3 <0.350 (p55)	54.07	0.81	17.14	0.09	7.998E-05
**166**	prop_higher_edu >0.226 (p60) &ave_household_size >2.484 (p11)	rate_local3 <0.328 (p52)	55.85	0.77	17.10	0.07	7.254E-05
**167**	prop_higher_edu >0.191 (p45) &prop_woodland >0.066 (p36)	rate_local3 <0.382 (p57)	57.52	0.82	16.56	0.13	4.994E-06
**168**	den_public_trans <38.548 (p72) &prop_publicResid <0.031 (p67)	rate_local3 <0.395 (p58)	78.64	0.75	16.45	0.08	5.161E-05
**169**	POI_pp_telecom_elec >20.167 (p68)	rate_local3 <0.043 (p16)	26.96	0.39	15.56	0.22	2.922E-10
**170**	med_area_home >19.838 (p65)	rate_local3 <0.340 (p54)	56.62	0.75	15.50	0.20	5.078E-08
**171**	prop_preprim_edu <0.126 (p82) &prop_higher_edu >0.215 (p55) &prop_woodland >0.102 (p50)	rate_local3 <0.395 (p58)	42.76	0.90	14.52	0.03	7.042E-05
**172**	prop_gov_insti_faci <0.023 (p33)	rate_local3 <0.010 (p15)	26.01	0.36	14.42	0.20	5.041E-09
**173**	build_area_pp >61.380 (p50)	rate_local3 <0.222 (p38)	58.89	0.52	14.37	0.13	6.155E-06
**174**	den_population <21684.808 (p53)	rate_local3 <0.267 (p47)	66.24	0.58	14.34	0.13	3.312E-05
**175**	den_public_trans <4.083 (p28)	rate_local3 <0.016 (p15)	23.50	0.40	14.24	0.24	9.331E-10
**176**	POI_pp_transport >55.037 (p74)	rate_local3 <0.041 (p16)	23.91	0.40	14.13	0.24	2.208E-10
**177**	med_income >24009.220 (p79)	rate_local3 <0.335 (p53)	38.57	0.84	13.99	0.31	3.4E-07
**178**	prop_higher_edu >0.237 (p62)	rate_local3 <0.335 (p53)	58.31	0.71	13.93	0.17	7.001E-06

**179**	prop_transport <0.141 (p55)	rate_local3 <0.382 (p57)	81.67	0.70	13.79	0.12	4.175E-05
**180**	POI_pp_sports >45.148 (p80)	rate_local3 <0.007 (p15)	19.72	0.47	13.04	0.31	3.503E-10
**181**	POI_pp_edu >22.794 (p81)	rate_local3 <0.015 (p15)	20.34	0.41	12.49	0.25	1.476E-10
**182**	prop_publicResid <0.063 (p74)	rate_local3 <0.295 (p50)	90.45	0.57	12.43	0.08	6.569E-05
**183**	den_road <9.024 (p43)	rate_local3 <0.407 (p59)	66.36	0.73	11.35	0.12	5.277E-05
**184**	prop_grassland >0.056 (p63) &POI_pp_edu >13.709 (p61)	rate_local3 <0.003 (p15)	16.47	0.51	11.30	0.20	3.968E-05
**185**	POI_pp_mall_mkt >13.520 (p82)	rate_local3 <0.028 (p16)	18.26	0.42	11.30	0.26	1.201E-08
**186**	prop_over65 <0.141 (p36)	rate_local3 <0.274 (p47)	44.95	0.61	10.53	0.14	8.153E-05
**187**	prop_woodland >0.322 (p82)	rate_local3 <0.409 (p59)	34.03	0.87	10.45	0.27	3.918E-05
**188**	den_bldg <0.136 (p43)	rate_local3 <0.411 (p59)	64.66	0.72	10.38	0.12	8.318E-05
**189**	prop_private_resid <0.021 (p36)	rate_local3 <0.041 (p16)	22.79	0.30	10.35	0.14	4.461E-05
**190**	ave_household_size >2.925 (p63)	rate_local3 <0.621 (p79)	78.93	0.91	9.95	0.11	8.125E-05
**191**	prop_shrubland >0.281 (p89)	rate_local3 <0.166 (p30)	16.40	0.71	9.31	0.40	1.539E-06
**192**	prop_business <0.039 (p84)	rate_local3 <0.518 (p71)	135.32	0.76	8.74	0.05	2.866E-05
**193**	prop_open_recreation <0.002 (p14)	rate_local3 <0.016 (p15)	13.07	0.44	8.31	0.28	9.219E-06
**194**	prop_preprim_edu >0.143 (p89)	rate_local3 <0.027 (p16)	12.07	0.45	7.72	0.29	8.83E-07
**195**	prop_private_resid >0.093 (p65) &prop_transport =0.062-0.249 (p35-73)	rate_local3 <0.293 (p50)	23.94	0.70	7.00	0.20	6.679E-05
**196**	prop_household_3gen >0.083 (p93)	rate_local3 <0.011 (p15)	9.00	0.56	6.44	0.40	4.402E-06

(d) Rules for wave-3 local case rate; POI accessibility was used

No.	Antecedent	Consequent	Supp	Conf	Lev	Imp	*P*
**1**	med_income <28651.117 (p84) &prop_grassland <0.020 (p46) &LU_entropy <0.553 (p49)	rate_local4 >0.837 (p79)	30.96	0.73	21.91	0.17	1.556E-06
**2**	med_income <23276.405 (p78) &den_road >7.322 (p35) &LU_entropy <0.551 (p49)	rate_local4 >0.804 (p78)	31.43	0.75	21.83	0.20	7.632E-07
**3**	med_income <23241.294 (p78) &prop_transport >0.066 (p35) &LU_entropy <0.553 (p49)	rate_local4 >0.815 (p78)	30.79	0.74	21.48	0.20	1.333E-06
**4**	med_income <23276.405 (p78) &den_bldg >0.121 (p39) &LU_entropy <0.551 (p49)	rate_local4 >0.804 (p78)	30.77	0.75	21.30	0.20	1.333E-06
**5**	med_income <23810.093 (p79) &prop_shrubland <0.014 (p34) &LU_entropy <0.551 (p49)	rate_local4 >0.804 (p78)	29.68	0.80	21.09	0.17	2.784E-06
**6**	med_income <23241.294 (p78) &prop_rural_set <0.002 (p49) &LU_entropy <0.553 (p49)	rate_local4 >0.815 (p78)	30.26	0.72	20.80	0.19	4.613E-06
**7**	med_area_home <19.307 (p61) &prop_grassland <0.023 (p48) &LU_entropy <0.551 (p49)	rate_local4 >0.804 (p78)	29.07	0.79	20.64	0.22	1.023E-05
**8**	med_income <23017.503 (p78) &prop_agricultural <0.009 (p58) &LU_entropy <0.551 (p49)	rate_local4 >0.827 (p79)	30.47	0.67	20.56	0.19	1.391E-06
**9**	ave_household_size <2.976 (p63) &prop_grassland <0.011 (p36) &LU_entropy <0.556 (p50)	rate_local4 >0.821 (p79)	29.60	0.73	20.54	0.14	3.422E-05
**10**	med_income <23372.096 (p78) &prop_woodland <0.065 (p36) &LU_entropy <0.551 (p49)	rate_local4 >0.814 (p78)	29.35	0.74	20.40	0.14	9.908E-06
**11**	den_road >10.434 (p49) &LU_entropy <0.551 (p49)	rate_local4 >0.788 (p77)	34.18	0.59	20.35	0.21	1.835E-07
**12**	den_bldg >0.158 (p47) &LU_entropy <0.551 (p49)	rate_local4 >0.788 (p77)	34.55	0.58	20.27	0.20	4.824E-07
**13**	den_population >19314.432 (p51) &prop_agricultural <0.012 (p60) &prop_shrubland <0.106 (p57)	rate_local4 >0.524 (p55)	55.05	0.73	20.27	0.05	7.448E-05
**14**	ave_household_size <2.976 (p63) &prop_agricultural <0.013 (p61) &LU_entropy <0.552 (p49)	rate_local4 >0.784 (p76)	32.73	0.63	20.21	0.13	4.611E-05
**15**	med_income <29532.689 (p84) &prop_grassland <0.007 (p31)	rate_local4 >0.852 (p80)	30.41	0.62	20.17	0.13	4.521E-05
**16**	prop_shrubland <0.018 (p36) &LU_entropy <0.543 (p46)	rate_local4 >0.784 (p76)	32.37	0.64	20.15	0.18	3.677E-07
**17**	prop_rural_set <0.026 (p66) &prop_shrubland <0.112 (p58)	rate_local4 >0.564 (p60)	58.38	0.64	20.10	0.10	6.15E-05
**18**	prop_rural_set <0.002 (p50) &LU_entropy <0.551 (p49)	rate_local4 >0.788 (p77)	35.56	0.54	19.91	0.17	5.059E-07
**19**	den_population >18056.735 (p50) &prop_shrubland <0.117 (p59)	rate_local4 >0.510 (p53)	62.02	0.70	19.88	0.08	3.985E-05
**20**	med_area_home <19.307 (p61) &den_bldg >0.158 (p47) &LU_entropy <0.551 (p49)	rate_local4 >0.804 (p78)	28.45	0.76	19.77	0.19	3.194E-05
**21**	den_population >18562.555 (p50) &prop_agricultural <0.010 (p59)	rate_local4 >0.481 (p49)	65.43	0.72	19.74	0.07	2.749E-05

**22**	med_income <29357.618 (p84) &den_road >11.996 (p56) &prop_agricultural <0.021 (p69)	rate_local4 >0.427 (p42)	59.56	0.85	19.69	0.04	5.566E-05
**23**	med_area_home <18.664 (p56) &den_road >7.251 (p34) &LU_entropy <0.551 (p49)	rate_local4 >0.792 (p77)	28.64	0.76	19.68	0.18	2.645E-05
**24**	prop_transport >0.106 (p44) &prop_agricultural <0.013 (p61)	rate_local4 >0.417 (p42)	77.13	0.77	19.60	0.06	1.139E-05
**25**	med_area_home <18.225 (p55) &prop_shrubland <0.141 (p66) &LU_entropy <0.551 (p49)	rate_local4 >0.796 (p77)	27.68	0.79	19.46	0.19	2.478E-06
**26**	prop_woodland <0.094 (p45) &LU_entropy <0.541 (p45)	rate_local4 >0.786 (p77)	31.90	0.62	19.41	0.22	1.983E-07
**27**	den_road >16.383 (p71)	rate_local4 >0.722 (p72)	36.83	0.60	19.40	0.31	4.628E-12
**28**	med_area_home <18.225 (p55) &prop_transport >0.099 (p43) &LU_entropy <0.551 (p49)	rate_local4 >0.801 (p78)	27.59	0.78	19.32	0.18	3.759E-05
**29**	med_income <23241.294 (p78) &den_public_trans >19.793 (p54) &LU_entropy <0.553 (p49)	rate_local4 >0.797 (p77)	28.24	0.74	19.30	0.15	5.69E-05
**30**	den_population >24987.561 (p56) &den_bldg >0.200 (p57)	rate_local4 >0.522 (p54)	50.05	0.75	19.30	0.10	3.24E-05
**31**	med_income <26594.841 (p83) &prop_transport >0.104 (p44) &prop_agricultural <0.013 (p61)	rate_local4 >0.386 (p39)	67.24	0.86	19.18	0.07	5.276E-05
**32**	den_population >18939.324 (p50) &LU_entropy <0.551 (p49)	rate_local4 >0.762 (p74)	31.52	0.64	18.95	0.25	2.438E-08
**33**	prop_agricultural <0.003 (p48) &LU_entropy <0.551 (p49)	rate_local4 >0.804 (p78)	33.41	0.53	18.91	0.16	1.653E-05
**34**	den_road >11.996 (p56) &prop_agricultural <0.021 (p69)	rate_local4 >0.457 (p45)	66.68	0.74	18.80	0.03	7.77E-05
**35**	med_income <27261.773 (p84) &prop_agricultural <0.014 (p61) &prop_grassland <0.067 (p66)	rate_local4 >0.421 (p42)	65.96	0.80	18.78	0.05	6.086E-05
**36**	med_area_home <18.225 (p55) &prop_woodland <0.088 (p43) &LU_entropy <0.551 (p49)	rate_local4 >0.796 (p77)	26.44	0.81	18.76	0.21	1.67E-05
**37**	prop_agricultural <0.012 (p60) &prop_shrubland <0.099 (p56)	rate_local4 >0.481 (p49)	66.92	0.70	18.72	0.09	2.668E-05
**38**	ave_household_size <2.969 (p63) &prop_rural_set <0.001 (p46) &LU_entropy <0.551 (p49)	rate_local4 >0.867 (p80)	27.47	0.63	18.69	0.13	6.515E-05
**39**	den_population >24987.561 (p56) &den_road >12.063 (p56)	rate_local4 >0.466 (p46)	54.94	0.79	18.68	0.09	3.783E-05
**40**	den_bldg >0.226 (p62)	rate_local4 >0.603 (p64)	50.39	0.60	18.56	0.22	3.089E-07
**41**	med_area_home <18.664 (p56) &prop_agricultural <0.010 (p59) &LU_entropy <0.551 (p49)	rate_local4 >0.797 (p77)	28.38	0.68	18.53	0.10	6.38E-05
**42**	den_public_trans >18.777 (p53) &LU_entropy <0.553 (p49)	rate_local4 >0.800 (p78)	30.72	0.59	18.52	0.22	5.005E-07
**43**	prop_grassland <0.027 (p51) &LU_entropy <0.551 (p49)	rate_local4 >0.905 (p81)	29.95	0.49	18.48	0.17	2.07E-06
**44**	prop_transport >0.096 (p41) &prop_woodland <0.128 (p53)	rate_local4 >0.767 (p74)	40.29	0.47	18.43	0.10	7.07E-05
**45**	prop_rural_set <0.011 (p59) &prop_transport >0.088 (p39)	rate_local4 >0.528 (p56)	60.81	0.65	18.05	0.08	7.362E-05
**46**	med_income <23878.185 (p79) &den_bldg >0.293 (p72)	rate_local4 >0.774 (p75)	29.22	0.65	17.99	0.13	5.123E-05
**47**	prop_shrubland <0.000 (p0)	rate_local4 >0.785 (p76)	27.11	0.71	17.92	0.47	4.929E-14

**48**	den_bldg >0.149 (p44) &prop_agricultural <0.024 (p71)	rate_local4 >0.476 (p49)	72.91	0.68	17.90	0.04	7.33E-05
**49**	den_bldg >0.117 (p37) &prop_woodland <0.124 (p53)	rate_local4 >0.788 (p77)	39.51	0.44	17.83	0.08	8.008E-05
**50**	prop_rural_set <0.010 (p57) &prop_woodland <0.096 (p46)	rate_local4 >0.737 (p74)	36.86	0.52	17.81	0.11	7.81E-05
**51**	med_area_home <16.801 (p50) &LU_entropy <0.551 (p49)	rate_local4 >0.813 (p78)	27.15	0.65	17.66	0.28	7.617E-08
**52**	ave_household_size <2.950 (p63) &med_area_home <21.925 (p78) &LU_entropy <0.555 (p50)	rate_local4 >0.770 (p75)	29.61	0.62	17.64	0.11	5.782E-05
**53**	prop_private_resid >0.057 (p55) &prop_woodland <0.108 (p50)	rate_local4 >0.752 (p74)	34.29	0.54	17.54	0.15	3.589E-05
**54**	med_income <23017.503 (p78) &prop_agricultural <0.022 (p70) &prop_shrubland <0.118 (p59)	rate_local4 >0.336 (p35)	70.16	0.89	17.52	0.08	1.854E-05
**55**	prop_agricultural <0.016 (p63) &prop_woodland <0.093 (p45)	rate_local4 >0.716 (p71)	38.72	0.52	17.52	0.09	7.897E-05
**56**	prop_agricultural <0.001 (p40)	rate_local4 >0.766 (p74)	39.90	0.46	17.43	0.20	1.127E-08
**57**	prop_grassland <0.004 (p23)	rate_local4 >0.848 (p80)	28.16	0.56	17.42	0.34	1.388E-13
**58**	prop_rural_set <0.011 (p59) &prop_grassland <0.026 (p51)	rate_local4 >0.528 (p56)	56.17	0.66	17.37	0.07	5.015E-05
**59**	med_income <27378.576 (p84) &prop_agricultural <0.011 (p60)	rate_local4 >0.383 (p38)	77.74	0.79	17.35	0.07	7.293E-05
**60**	med_income <23017.503 (p78) &prop_private_resid >0.045 (p49) &LU_entropy <0.551 (p49)	rate_local4 >0.794 (p77)	25.50	0.73	17.28	0.20	4.009E-05
**61**	ave_household_size <2.908 (p63) &LU_entropy <0.551 (p49)	rate_local4 >0.811 (p78)	30.42	0.53	17.27	0.16	3.321E-05
**62**	ave_household_size <2.739 (p37) &prop_grassland <0.007 (p30)	rate_local4 >0.722 (p72)	26.84	0.79	17.08	0.22	6.76E-05
**63**	ave_household_size <2.771 (p37) &prop_agricultural <0.001 (p43)	rate_local4 >0.694 (p70)	31.12	0.67	17.07	0.18	7.577E-05
**64**	med_income <23017.503 (p78) &prop_open_recreation >0.026 (p45) &LU_entropy <0.551 (p49)	rate_local4 >0.827 (p79)	24.94	0.69	17.05	0.17	2.642E-05
**65**	den_population >26219.620 (p60)	rate_local4 >0.466 (p46)	63.71	0.71	16.81	0.19	1.213E-06
**66**	ave_household_size <2.771 (p37) &prop_rural_set <0.005 (p53)	rate_local4 >0.694 (p70)	32.72	0.62	16.77	0.18	6.159E-05
**67**	med_income <24196.197 (p79) &prop_rural_set <0.034 (p70) &prop_shrubland <0.081 (p53)	rate_local4 >0.350 (p36)	58.38	0.91	16.76	0.09	6.451E-05
**68**	med_income <24580.681 (p79) &prop_private_resid >0.103 (p66)	rate_local4 >0.794 (p77)	27.32	0.60	16.50	0.17	6.413E-05
**69**	prop_private_resid >0.070 (p58) &LU_entropy <0.543 (p46)	rate_local4 >0.789 (p77)	27.76	0.58	16.37	0.20	6.484E-07
**70**	prop_agricultural <0.013 (p61) &POI_pp_telecom_elec <20.130 (p68)	rate_local4 >0.309 (p30)	82.26	0.86	16.16	0.07	7.365E-05
**71**	ave_household_size <2.660 (p29) &prop_woodland <0.064 (p36)	rate_local4 >0.751 (p74)	26.29	0.67	16.00	0.19	6.676E-05
**72**	ave_household_size <2.688 (p29) &prop_shrubland <0.048 (p45)	rate_local4 >0.753 (p74)	27.23	0.63	15.93	0.19	7.356E-05
**73**	prop_agricultural <0.016 (p63) &POI_pp_edu <22.044 (p79)	rate_local4 >0.297 (p29)	89.86	0.86	15.93	0.06	7.813E-06
**74**	prop_agricultural <0.016 (p63) &POI_pp_transport <46.920 (p69)	rate_local4 >0.297 (p29)	82.99	0.87	15.90	0.07	2.413E-05
**75**	med_area_home <17.445 (p52) &prop_agricultural <0.016 (p63) &POI_pp_edu <22.044 (p79)	rate_local4 >0.297 (p29)	69.95	0.91	15.88	0.04	8.253E-07
**76**	prop_rural_set <0.001 (p46)	rate_local4 >0.630 (p64)	50.56	0.52	15.79	0.16	4.681E-05

77	med_income <26849.258 (p83) &prop_rural_set <0.030 (p69) &prop_transport >0.094 (p40)	rate_local4 >0.297 (p29)	69.42	0.91	15.78	0.06	2.417E-05
78	med_income <25084.765 (p81) &den_road >12.237 (p56) &prop_industrial <0.000 (p36)	rate_local4 >0.963 (p84)	20.91	0.67	15.71	0.22	4.003E-05
79	prop_open_recreation >0.030 (p48) &LU_entropy <0.551 (p49)	rate_local4 >0.804 (p78)	26.92	0.55	15.70	0.18	7.193E-06
80	prop_agricultural <0.016 (p63) &POI_pp_mall_mkt <11.956 (p79)	rate_local4 >0.297 (p29)	87.04	0.86	15.70	0.06	1.027E-05
81	med_income <26890.250 (p83) &prop_business >0.020 (p77) &LU_entropy <0.557 (p50)	rate_local4 >0.794 (p77)	22.76	0.75	15.61	0.14	4.218E-05
82	den_bldg >0.113 (p37) &prop_shrubland <0.133 (p64)	rate_local4 >0.425 (p42)	83.37	0.70	15.56	0.05	6.068E-05
83	med_income <23549.657 (p78) &den_road >12.275 (p56)	rate_local4 >0.332 (p33)	61.54	0.90	15.55	0.08	5.389E-05
84	med_area_home <18.774 (p57) &prop_rural_set <0.004 (p52) &LU_entropy <0.526 (p40)	rate_local4 >0.702 (p71)	25.35	0.77	15.55	0.12	6.675E-05
85	med_income <24082.337 (p79) &prop_publicResid <0.019 (p64) &prop_transport >0.102 (p43)	rate_local4 >0.809 (p78)	24.24	0.63	15.46	0.18	6.286E-05
86	med_income <24190.580 (p79) &den_bldg >0.159 (p47) &prop_industrial <0.001 (p50)	rate_local4 >0.868 (p80)	22.66	0.62	15.38	0.20	5.334E-05
87	med_area_home <17.682 (p54) &prop_agricultural <0.025 (p71)	rate_local4 >0.398 (p40)	72.64	0.76	15.38	0.08	3.933E-05
88	med_income <27900.398 (p84) &prop_rural_set <0.001 (p48)	rate_local4 >0.334 (p34)	66.11	0.87	15.33	0.08	6.776E-06
89	med_area_home <17.445 (p52) &prop_agricultural <0.016 (p63) &POI_pp_transport <46.920 (p69)	rate_local4 >0.297 (p29)	66.19	0.92	15.28	0.04	4.646E-06
90	den_population >25206.184 (p57) &prop_transport >0.225 (p69)	rate_local4 >0.459 (p45)	43.12	0.82	15.27	0.07	5.948E-05
91	prop_transport >0.104 (p44) &LU_entropy <0.535 (p43)	rate_local4 >1.002 (p86)	24.18	0.47	15.27	0.18	5.883E-06
92	med_income <23865.262 (p79) &prop_transport >0.225 (p69)	rate_local4 >0.431 (p43)	45.30	0.85	15.25	0.08	5.562E-05
93	ave_household_size <2.924 (p63) &prop_private_resid >0.085 (p63)	rate_local4 >0.788 (p77)	26.46	0.56	15.23	0.16	4.897E-05
94	ave_household_size <2.784 (p37) &den_bldg >0.117 (p37)	rate_local4 >0.703 (p71)	34.91	0.52	15.20	0.11	8.025E-05
95	med_income <31762.966 (p88) &den_bldg >0.160 (p47) &prop_shrubland <0.132 (p64)	rate_local4 >0.299 (p29)	76.13	0.88	15.03	0.03	5.7E-05
96	med_income <23954.158 (p79) &den_public_trans >19.357 (p54)	rate_local4 >0.330 (p33)	71.48	0.85	15.01	0.05	3.628E-05
97	med_area_home <16.985 (p52) &prop_agricultural <0.016 (p63) &POI_pp_sports <35.908 (p73)	rate_local4 >0.297 (p29)	66.41	0.91	14.99	0.03	1.638E-05
98	med_income <23935.978 (p79) &den_road >9.841 (p46) &prop_rural_set <0.005 (p54)	rate_local4 >0.308 (p30)	58.82	0.93	14.97	0.06	8.219E-05
99	med_income <28540.924 (p84) &prop_agricultural <0.012 (p60) &POI_pp_edu <21.253 (p78)	rate_local4 >0.297 (p29)	72.28	0.89	14.96	0.03	7.126E-05
100	med_area_home <17.445 (p52) &prop_agricultural <0.016 (p63) &POI_pp_telecom_elec <18.648 (p66)	rate_local4 >0.297 (p29)	65.22	0.91	14.85	0.04	3.325E-06

**101**	build_area_pp <62.470 (p50) &prop_agricultural <0.013 (p61)	rate_local4 >0.309 (p30)	65.43	0.90	14.84	0.10	4.488E-06
**102**	prop_industrial <0.000 (p41) &prop_woodland <0.079 (p41)	rate_local4 >0.978 (p85)	21.03	0.53	14.74	0.23	1.442E-05
**103**	ave_household_size <2.787 (p37) &prop_industrial <0.000 (p36)	rate_local4 >0.868 (p80)	22.23	0.60	14.73	0.23	1.409E-05
**104**	med_income <23641.798 (p78) &den_public_trans >18.687 (p53) &prop_publicResid <0.021 (p65)	rate_local4 >0.855 (p80)	21.86	0.64	14.70	0.18	5.161E-05
**105**	prop_gov_insti_faci >0.025 (p36) &LU_entropy <0.546 (p47)	rate_local4 >0.765 (p74)	29.27	0.51	14.66	0.11	5.005E-05
**106**	med_income <24082.337 (p79) &den_bldg >0.143 (p43) &prop_publicResid <0.019 (p64)	rate_local4 >0.847 (p80)	23.71	0.55	14.62	0.16	3.651E-05
**107**	prop_transport >0.241 (p71)	rate_local4 >0.897 (p81)	25.98	0.43	14.55	0.24	3.647E-08
**108**	prop_business >0.020 (p77) &LU_entropy <0.547 (p47)	rate_local4 >0.805 (p78)	22.74	0.62	14.36	0.21	3.029E-05
**109**	med_income <26378.359 (p83) &med_area_home <17.924 (p55) &prop_rural_set <0.012 (p59)	rate_local4 >0.299 (p29)	67.89	0.89	14.33	0.02	4.846E-05
**110**	LU_entropy <0.548 (p47)	rate_local4 >0.863 (p80)	34.55	0.34	14.21	0.14	1.67E-07
**111**	med_income <20626.543 (p73) &den_road >10.786 (p50) &prop_publicResid <0.017 (p63)	rate_local4 >0.871 (p80)	20.25	0.66	14.15	0.24	5.195E-05
**112**	ave_household_size <2.670 (p29) &den_population >17876.725 (p50)	rate_local4 >0.648 (p66)	27.29	0.70	13.98	0.18	6.353E-05
**113**	prop_private_resid >0.087 (p64)	rate_local4 >0.731 (p73)	36.23	0.45	13.84	0.17	1E-06
**114**	med_income <21943.402 (p75) &prop_publicResid <0.003 (p55) &prop_agricultural <0.009 (p59)	rate_local4 >0.846 (p80)	19.86	0.67	13.60	0.24	1.993E-05
**115**	build_area_pp <60.573 (p50) &prop_rural_set <0.035 (p70)	rate_local4 >0.307 (p30)	67.74	0.87	13.38	0.06	2.228E-05
**116**	med_income <20556.841 (p73) &prop_publicResid <0.012 (p61) &prop_shrubland <0.036 (p41)	rate_local4 >0.964 (p84)	18.25	0.63	13.37	0.20	7.668E-05
**117**	den_public_trans >26.097 (p59) &prop_publicResid <0.010 (p60)	rate_local4 >0.917 (p81)	20.79	0.50	13.35	0.19	5.171E-05
**118**	med_area_home <15.838 (p46) &POI_pp_edu =4.426-24.205 (p27-81)	rate_local4 >0.579 (p62)	30.04	0.72	13.20	0.22	1.864E-05
**119**	med_income <24082.337 (p79) &prop_rural_set <0.001 (p46) &POI_pp_sports >19.996 (p53)	rate_local4 >0.827 (p79)	19.48	0.68	13.18	0.22	4.616E-05
**120**	med_income <22034.807 (p77) &prop_publicResid <0.000 (p44) &prop_rural_set <0.001 (p48)	rate_local4 >0.883 (p81)	17.76	0.75	13.17	0.27	5.077E-05
**121**	prop_industrial <0.000 (p41) &prop_transport >0.211 (p66)	rate_local4 >1.076 (p87)	17.70	0.51	13.12	0.22	7.716E-05
**122**	med_area_home <17.924 (p55) &prop_rural_set <0.012 (p59)	rate_local4 >0.299 (p29)	68.29	0.87	13.12	0.09	5.205E-05
**123**	med_area_home <18.806 (p57) &prop_publicResid <0.023 (p66) &prop_grassland <0.027 (p51)	rate_local4 >0.889 (p81)	18.93	0.63	13.11	0.24	3.253E-05
**124**	med_income <24082.337 (p79) &den_road >10.205 (p48) &POI_pp_mall_mkt >6.459 (p57)	rate_local4 >0.895 (p81)	18.62	0.63	13.02	0.22	4.562E-05
**125**	med_area_home <17.143 (p52) &prop_grassland <0.079 (p70)	rate_local4 >0.253 (p24)	78.04	0.89	12.96	0.07	3.482E-05
**126**	ave_household_size <2.713 (p37)	rate_local4 >0.865 (p80)	27.87	0.38	12.90	0.17	2.343E-07

**127**	med_income <24082.337 (p79) &prop_transport >0.108 (p46) &POI_pp_sports >19.996 (p53)	rate_local4 >0.802 (p78)	19.76	0.66	12.80	0.18	2.342E-05
**128**	med_income <24082.337 (p79) &den_road >10.205 (p48) &POI_pp_sports >19.996 (p53)	rate_local4 >0.827 (p79)	19.00	0.67	12.74	0.21	1.948E-05
**129**	prop_industrial <0.000 (p36)	rate_local4 >1.033 (p86)	23.82	0.31	12.74	0.17	2.01E-07
**130**	med_income <23276.021 (p78) &prop_rural_set <0.001 (p47) &POI_pp_mall_mkt >7.209 (p60)	rate_local4 >0.867 (p80)	17.95	0.68	12.65	0.24	1.314E-05
**131**	med_income <24271.605 (p79) &den_bldg >0.160 (p47) &POI_pp_mall_mkt >7.122 (p60)	rate_local4 >0.856 (p80)	18.49	0.65	12.62	0.21	5.202E-06
**132**	med_income <23241.294 (p78) &prop_rural_set <0.002 (p49) &POI_pp_transport >32.416 (p54)	rate_local4 >0.815 (p78)	18.37	0.70	12.49	0.24	6.878E-05
**133**	med_income <14989.703 (p27) &prop_woodland <0.048 (p31)	rate_local4 >0.844 (p80)	17.70	0.70	12.39	0.25	4.493E-05
**134**	med_income <14793.785 (p26) &LU_entropy <0.523 (p40)	rate_local4 >0.824 (p79)	16.83	0.83	12.33	0.46	1.042E-07
**135**	med_income <20556.841 (p73) &prop_publicResid <0.012 (p61) &prop_grassland <0.021 (p46)	rate_local4 >0.964 (p84)	16.86	0.61	12.21	0.21	3.042E-05
**136**	prop_preprim_edu <0.143 (p88) &med_area_home <17.670 (p54)	rate_local4 >0.289 (p29)	86.10	0.83	12.14	0.05	4.795E-05
**137**	den_population >23908.264 (p55) &prop_publicResid <0.003 (p55)	rate_local4 >0.798 (p77)	19.59	0.62	12.05	0.24	3.907E-05
**138**	med_income <24082.337 (p79) &prop_transport >0.108 (p46) &POI_pp_transport >32.812 (p55)	rate_local4 >0.802 (p78)	18.40	0.67	12.03	0.19	4.034E-05
**139**	med_income <24082.337 (p79) &den_road >10.205 (p48) &POI_pp_transport >33.359 (p56)	rate_local4 >0.816 (p78)	17.62	0.69	11.89	0.23	3.485E-05
**140**	med_income <24618.752 (p79) &prop_shrubland <0.006 (p29)	rate_local4 >0.349 (p36)	41.49	0.91	11.88	0.10	3.884E-05
**141**	med_income <20556.841 (p73) &prop_private_resid >0.053 (p52) &prop_publicResid <0.012 (p61)	rate_local4 >0.964 (p84)	16.79	0.57	11.83	0.23	7.817E-05
**142**	med_income <24082.337 (p79) &prop_transport >0.108 (p46) &POI_pp_mall_mkt >7.865 (p63)	rate_local4 >0.802 (p78)	17.48	0.71	11.81	0.22	1.27E-05
**143**	med_income <24082.337 (p79) &den_road >11.631 (p53) &POI_pp_telecom_elec >12.518 (p51)	rate_local4 >0.849 (p80)	17.23	0.66	11.75	0.21	6.413E-05
**144**	med_income <23276.021 (p78) &prop_agricultural <0.008 (p57) &POI_pp_mall_mkt >7.209 (p60)	rate_local4 >0.867 (p80)	17.53	0.61	11.70	0.22	3.66E-05
**145**	prop_business >0.058 (p86)	rate_local4 >0.784 (p76)	18.49	0.65	11.61	0.41	1.455E-08
**146**	prop_agricultural <0.017 (p64) &prop_grassland <0.071 (p68) &POI_pp_transport <21.102 (p43)	rate_local4 >0.289 (p29)	46.01	0.94	11.25	0.05	5.489E-05
**147**	med_area_home <16.759 (p50) &prop_private_resid >0.175 (p80)	rate_local4 >0.834 (p79)	15.16	0.75	10.75	0.29	6.168E-05
**148**	med_income <23943.235 (p79) &den_bldg >0.160 (p47) &POI_pp_sports >23.218 (p59)	rate_local4 >0.867 (p80)	16.03	0.61	10.71	0.20	2.676E-05

**149**	med_area_home <16.705 (p50) &POI_pp_mall_mkt =3.036-11.675 (p38-77)	rate_local4 >0.685 (p70)	19.54	0.68	10.63	0.27	3.488E-06
**150**	med_income <24424.417 (p79) &prop_agricultural <0.005 (p51) &POI_pp_sports >23.218 (p59)	rate_local4 >0.830 (p79)	16.00	0.62	10.33	0.20	7.629E-05
**151**	med_area_home <18.066 (p55) &prop_publicResid <0.000 (p44)	rate_local4 >0.906 (p81)	15.35	0.55	10.17	0.29	2.137E-05
**152**	build_area_pp <58.100 (p47)	rate_local4 >0.222 (p21)	83.55	0.87	10.09	0.11	1.522E-05
**153**	prop_woodland <0.016 (p25)	rate_local4 >1.282 (p90)	15.27	0.29	10.03	0.19	3.756E-08
**154**	med_area_home <15.641 (p45)	rate_local4 >0.299 (p29)	74.53	0.81	9.64	0.10	7.299E-05
**155**	med_income <23865.262 (p79) &prop_business >0.038 (p84)	rate_local4 >0.431 (p43)	28.14	0.84	9.20	0.11	6.398E-05
**156**	med_income <23641.798 (p78) &prop_shrubland <0.048 (p44) &POI_pp_transport >42.763 (p64)	rate_local4 >0.984 (p85)	11.09	0.62	8.25	0.22	7.123E-05
**157**	med_area_home <18.225 (p55) &POI_pp_sports >22.655 (p58)	rate_local4 >0.870 (p80)	14.21	0.45	7.90	0.18	4.278E-05
**158**	den_public_trans >95.120 (p92)	rate_local4 >0.780 (p76)	12.45	0.67	7.89	0.42	1.191E-05
**159**	den_public_trans =46.130-95.120 (p78-92)	rate_local4 >0.780 (p76)	15.91	0.45	7.37	0.21	6.368E-05
**160**	med_income <12559.030 (p7)	rate_local4 >0.831 (p79)	8.91	0.64	5.86	0.42	2.821E-05
**161**	prop_household_3gen <0.018 (p15) &med_area_home =13.055-16.289 (p18-49)	rate_local4 >0.807 (p78)	5.48	0.90	4.08	0.55	8.043E-05
**162**	gender_ratio >87.552 (p11) &den_public_trans <62.626 (p84) &POI_pp_transport <63.461 (p78)	rate_local4 =0.074-0.862 (p13-80)	106.96	0.87	25.33	0.04	2.889E-06
**163**	gender_ratio >87.552 (p11) &prop_business <0.055 (p86) &POI_pp_transport <61.104 (p78)	rate_local4 =0.116-1.001 (p14-86)	114.51	0.89	23.71	0.03	2.871E-05
**164**	den_road <17.297 (p74) &POI_pp_transport <61.104 (p78)	rate_local4 =0.119-0.854 (p14-80)	104.43	0.84	23.63	0.09	2.29E-06
**165**	gender_ratio >87.664 (p11) &den_road <22.221 (p86) &POI_pp_telecom_elec <30.272 (p80)	rate_local4 =0.075-0.782 (p13-76)	102.72	0.81	23.47	0.03	6.635E-05
**166**	gender_ratio >87.552 (p11) &ave_household_size >2.554 (p17) &POI_pp_transport <63.461 (p78)	rate_local4 =0.029-1.030 (p13-86)	109.42	0.91	23.23	0.04	8.243E-05
**167**	den_road <17.068 (p72) &POI_pp_telecom_elec <28.789 (p79)	rate_local4 =0.117-0.793 (p14-77)	99.71	0.81	23.17	0.09	4.255E-08
**168**	prop_preprim_edu <0.149 (p90) &ave_household_size >2.627 (p29) &POI_pp_transport <64.846 (p78)	rate_local4 =0.152-0.892 (p15-81)	99.66	0.85	23.05	0.03	1.642E-05
**169**	gender_ratio >87.552 (p11) &LU_entropy >0.507 (p36) &POI_pp_transport <60.769 (p78)	rate_local4 =0.127-0.859 (p14-80)	91.26	0.87	22.93	0.05	2.403E-06
**170**	den_public_trans <48.505 (p79) &POI_pp_transport <60.966 (p78)	rate_local4 =0.075-0.771 (p13-75)	101.81	0.79	22.78	0.08	1.151E-05
**171**	den_bldg <0.442 (p84) &POI_pp_transport <61.104 (p78)	rate_local4 =0.119-0.854 (p14-80)	114.32	0.81	22.58	0.06	2.049E-06
**172**	den_public_trans <62.360 (p84) &POI_pp_sports <27.660 (p65)	rate_local4 =0.119-0.893 (p14-81)	101.26	0.86	22.57	0.07	1.239E-08
**173**	den_public_trans <59.674 (p84) &POI_pp_telecom_elec <29.787 (p80)	rate_local4 =0.146-0.860 (p15-80)	112.76	0.81	22.56	0.07	2.46E-08

**174**	den_road <17.892 (p75) &POI_pp_sports <27.660 (p65)	rate_local4 =0.119-0.893 (p14-81)	94.09	0.88	22.43	0.08	4.124E-08
**175**	gender_ratio >87.321 (p11) &den_bldg <0.327 (p76) &POI_pp_telecom_elec <30.098 (p80)	rate_local4 =0.119-0.926 (p14-82)	99.58	0.88	22.43	0.03	3.516E-05
**176**	gender_ratio >87.664 (p11) &den_road <22.221 (p86) &POI_pp_sports <35.319 (p73)	rate_local4 =0.132-0.855 (p14-80)	99.17	0.84	22.35	0.02	3.001E-05
**177**	prop_business <0.055 (p86) &POI_pp_transport <61.104 (p78)	rate_local4 =0.116-1.001 (p14-86)	125.70	0.86	22.29	0.04	8.775E-06
**178**	prop_preprim_edu <0.152 (p91) &den_road <23.627 (p88) &POI_pp_transport <36.856 (p59)	rate_local4 =0.156-0.824 (p15-79)	87.61	0.84	22.11	0.03	6.631E-05
**179**	LU_entropy >0.529 (p41) &POI_pp_telecom_elec <29.095 (p79)	rate_local4 =0.140-0.863 (p14-80)	93.04	0.84	22.04	0.06	3.469E-06
**180**	prop_business <0.055 (p86) &POI_pp_telecom_elec <29.275 (p79)	rate_local4 =0.116-1.001 (p14-86)	126.69	0.85	21.99	0.05	8.133E-07
**181**	gender_ratio >87.719 (p11) &LU_entropy >0.509 (p36) &POI_pp_telecom_elec <28.491 (p79)	rate_local4 =0.130-1.016 (p14-86)	95.60	0.92	21.88	0.04	3.473E-08
**182**	LU_entropy >0.522 (p40) &POI_pp_sports <28.731 (p66)	rate_local4 =0.119-0.888 (p14-81)	89.34	0.88	21.87	0.09	1.684E-06
**183**	gender_ratio >87.552 (p11) &LU_entropy >0.509 (p36) &POI_pp_sports <39.565 (p78)	rate_local4 =0.136-0.875 (p14-80)	89.58	0.87	21.78	0.03	3.063E-06
**184**	den_road <17.665 (p75) &POI_pp_edu <15.683 (p69)	rate_local4 =0.119-0.888 (p14-81)	96.47	0.86	21.72	0.08	2.669E-05
**185**	gender_ratio >87.664 (p11) &den_road <23.313 (p87) &POI_pp_edu <20.490 (p76)	rate_local4 =0.132-0.855 (p14-80)	103.30	0.82	21.67	0.03	7.89E-05
**186**	prop_preprim_edu <0.152 (p91) &den_road <23.627 (p88) &POI_pp_edu <18.296 (p71)	rate_local4 =0.127-1.168 (p14-90)	113.10	0.91	21.61	0.03	8.729E-06
**187**	prop_preprim_edu <0.149 (p90) &LU_entropy >0.543 (p46)	rate_local4 =0.152-0.892 (p15-81)	89.08	0.87	21.59	0.06	1.641E-07
**188**	den_public_trans <60.562 (p84) &POI_pp_edu <14.808 (p67)	rate_local4 =0.148-0.855 (p15-80)	98.55	0.83	21.58	0.06	1.276E-06
**189**	ave_household_size >2.713 (p37) &POI_pp_transport <65.844 (p78)	rate_local4 =0.119-0.865 (p14-80)	95.57	0.85	21.58	0.09	3.322E-06
**190**	prop_preprim_edu <0.134 (p84) &prop_business <0.078 (p89) &POI_pp_transport <73.036 (p82)	rate_local4 =0.185-1.255 (p16-90)	118.43	0.88	21.56	0.04	4.998E-06
**191**	gender_ratio >87.484 (p11) &prop_preprim_edu <0.149 (p90) &den_public_trans <68.485 (p86)	rate_local4 =0.152-0.892 (p15-81)	113.66	0.81	21.50	0.04	6.846E-06
**192**	prop_preprim_edu <0.155 (p92) &den_public_trans <78.224 (p88) &POI_pp_transport <73.036 (p82)	rate_local4 =0.176-1.249 (p16-90)	126.32	0.88	21.43	0.03	4.512E-05
**193**	gender_ratio >87.552 (p11) &den_public_trans <59.812 (p84) &POI_pp_edu <24.132 (p81)	rate_local4 =0.061-0.885 (p13-81)	107.41	0.84	21.36	0.04	2.81E-05
**194**	build_area_pp <58.100 (p47)	rate_local4 =0.119-0.888 (p14-81)	91.57	0.87	21.36	0.08	1.104E-05
**195**	gender_ratio >87.313 (p11) &POI_pp_transport <60.966 (p78)	rate_local4 =0.042-1.091 (p13-87)	125.64	0.88	21.31	0.04	5.07E-06
**196**	prop_business <0.048 (p86) &POI_pp_sports <34.167 (p71)	rate_local4 =0.122-1.008 (p14-86)	115.53	0.86	21.29	0.05	5.447E-05
**197**	ave_household_size >2.577 (p17) &POI_pp_edu <15.808 (p69)	rate_local4 =0.141-0.946 (p14-83)	101.59	0.86	21.26	0.06	8.574E-06
**198**	den_public_trans <62.360 (p84) &POI_pp_mall_mkt <8.569 (p65)	rate_local4 =0.119-0.893 (p14-81)	102.35	0.84	21.24	0.05	4.219E-07

**199**	gender_ratio >87.552 (p11) &LU_entropy >0.507 (p36) &POI_pp_edu <24.229 (p81)	rate_local4 =0.064-0.851 (p13-80)	92.87	0.85	21.20	0.04	2.794E-06
**200**	prop_preprim_edu <0.149 (p90) &den_public_trans <75.883 (p88) &POI_pp_mall_mkt <13.767 (p82)	rate_local4 =0.152-0.892 (p15-81)	105.98	0.82	21.16	0.04	8.19E-05
**201**	LU_entropy >0.522 (p40) &POI_pp_transport <45.562 (p69)	rate_local4 =0.119-0.888 (p14-81)	90.82	0.87	21.11	0.08	1.949E-06
**202**	prop_preprim_edu <0.149 (p90) &den_road <16.141 (p71) &POI_pp_mall_mkt <13.767 (p82)	rate_local4 =0.152-0.892 (p15-81)	89.90	0.86	21.07	0.05	7.633E-05
**203**	den_road <18.362 (p76) &POI_pp_mall_mkt <9.434 (p66)	rate_local4 =0.133-0.898 (p14-81)	99.36	0.85	21.05	0.06	6.746E-06
**204**	ave_household_size >2.627 (p29) &POI_pp_telecom_elec <31.680 (p80)	rate_local4 =0.152-0.892 (p15-81)	105.85	0.82	20.97	0.08	9.036E-06
**205**	prop_preprim_edu <0.149 (p90) &prop_business <0.057 (p86) &POI_pp_mall_mkt <13.767 (p82)	rate_local4 =0.152-0.892 (p15-81)	107.11	0.82	20.96	0.04	3.917E-05
**206**	prop_preprim_edu <0.149 (p90) &ave_household_size >2.627 (p29) &POI_pp_mall_mkt <12.788 (p81)	rate_local4 =0.152-0.892 (p15-81)	96.03	0.84	20.88	0.03	4.652E-05
**207**	LU_entropy >0.522 (p40) &POI_pp_mall_mkt <9.905 (p70)	rate_local4 =0.171-0.959 (p16-83)	92.31	0.86	20.87	0.08	2.491E-05
**208**	den_bldg <0.312 (p75) &POI_pp_telecom_elec <31.454 (p80)	rate_local4 =0.141-0.802 (p14-78)	98.86	0.78	20.84	0.08	5.531E-05
**209**	prop_preprim_edu <0.133 (p84) &ave_household_size >2.577 (p17) &POI_pp_edu <15.808 (p69)	rate_local4 =0.077-1.261 (p13-90)	101.16	0.96	20.83	0.03	7.277E-05
**210**	prop_business <0.043 (p85) &POI_pp_edu <16.667 (p69)	rate_local4 =0.130-0.987 (p14-85)	110.72	0.86	20.81	0.05	1.533E-06
**211**	gender_ratio >87.664 (p11) &ave_household_size >2.622 (p29) &POI_pp_edu <19.430 (p74)	rate_local4 =0.072-0.894 (p13-81)	93.60	0.87	20.80	0.03	8.129E-06
**212**	gender_ratio >87.552 (p11) &ave_household_size >2.478 (p11) &POI_pp_telecom_elec <29.632 (p80)	rate_local4 =0.124-0.871 (p14-80)	105.83	0.82	20.77	0.03	5.806E-06
**213**	gender_ratio >87.552 (p11) &den_bldg <0.323 (p76) &POI_pp_edu <21.469 (p79)	rate_local4 =0.074-0.862 (p13-80)	94.34	0.85	20.72	0.03	5.601E-05
**214**	prop_preprim_edu <0.138 (p85) &prop_business <0.055 (p86) &POI_pp_edu <16.401 (p69)	rate_local4 =0.130-1.129 (p14-88)	101.12	0.91	20.60	0.04	2.738E-05
**215**	gender_ratio >87.533 (p11) &prop_business <0.058 (p86) &POI_pp_edu <19.430 (p74)	rate_local4 =0.124-0.871 (p14-80)	103.71	0.82	20.43	0.02	1.845E-05
**216**	den_bldg <0.311 (p75) &POI_pp_sports <33.191 (p70)	rate_local4 =0.083-0.884 (p13-81)	93.81	0.86	20.39	0.07	1.873E-05
**217**	ave_household_size >2.659 (p29) &POI_pp_sports <37.538 (p75)	rate_local4 =0.125-0.871 (p14-80)	95.49	0.84	20.38	0.08	7.254E-06
**218**	prop_preprim_edu <0.133 (p84) &POI_pp_edu <15.808 (p69)	rate_local4 =0.077-1.261 (p13-90)	117.26	0.93	20.35	0.03	3.944E-05
**219**	prop_preprim_edu <0.144 (p89) &POI_pp_mall_mkt <8.043 (p64)	rate_local4 =0.178-1.268 (p16-90)	108.78	0.89	20.22	0.03	7.586E-05
**220**	gender_ratio >87.664 (p11) &prop_business <0.058 (p86) &POI_pp_telecom_elec <38.005 (p86)	rate_local4 =0.132-0.855 (p14-80)	111.05	0.79	20.19	0.03	1.941E-05

**221**	gender_ratio >87.552 (p11) &den_public_trans <59.812 (p84) &POI_pp_mall_mkt <11.259 (p75)	rate_local4 =0.093-1.007 (p13-86)	106.54	0.87	20.01	0.03	5.631E-05
**222**	den_bldg <0.331 (p77) &POI_pp_edu <18.032 (p71)	rate_local4 =0.075-0.893 (p13-81)	97.26	0.85	20.00	0.07	2.433E-06
**223**	prop_preprim_edu <0.138 (p85) &prop_business <0.055 (p86) &POI_pp_telecom_elec <40.063 (p87)	rate_local4 =0.130-1.129 (p14-88)	119.06	0.87	19.73	0.04	4.165E-05
**224**	prop_preprim_edu <0.139 (p86) &den_bldg <0.327 (p76) &POI_pp_mall_mkt <13.993 (p82)	rate_local4 =0.130-0.835 (p14-79)	88.22	0.82	19.65	0.04	8.07E-05
**225**	gender_ratio >87.552 (p11) &ave_household_size >2.554 (p17) &POI_pp_sports <51.798 (p85)	rate_local4 =0.121-1.214 (p14-90)	117.63	0.90	19.61	0.03	1.906E-05
**226**	prop_preprim_edu <0.136 (p85) &den_bldg <0.302 (p74) &POI_pp_transport <73.313 (p82)	rate_local4 =0.040-1.093 (p13-87)	96.83	0.92	19.58	0.04	3.686E-05
**227**	gender_ratio >87.329 (p11) &prop_preprim_edu <0.138 (p86) &ave_household_size >2.491 (p11)	rate_local4 =0.139-1.229 (p14-90)	122.96	0.89	19.55	0.04	1.363E-07
**228**	den_bldg <0.331 (p77) &POI_pp_telecom_elec <16.177 (p59) &POI_pp_mall_mkt <10.957 (p75)	rate_local4 =0.053-1.094 (p13-87)	89.21	0.94	19.54	0.01	2.266E-06
**229**	gender_ratio >87.484 (p11) &prop_preprim_edu <0.149 (p90) &prop_business <0.043 (p85)	rate_local4 =0.152-0.892 (p15-81)	112.11	0.79	19.47	0.03	3.258E-05
**230**	gender_ratio >87.533 (p11) &prop_business <0.058 (p86) &POI_pp_mall_mkt <17.630 (p86)	rate_local4 =0.124-0.871 (p14-80)	114.86	0.79	19.45	0.03	3.286E-05
**231**	prop_woodland >0.100 (p48) &POI_pp_transport <61.104 (p78)	rate_local4 =0.119-0.854 (p14-80)	78.39	0.87	19.34	0.11	4.325E-05
**232**	prop_preprim_edu <0.138 (p86) &den_road <17.068 (p72)	rate_local4 =0.145-0.867 (p15-80)	101.63	0.80	19.20	0.06	2.522E-06
**233**	gender_ratio >87.552 (p11) &ave_household_size >2.602 (p29) &POI_pp_mall_mkt <10.696 (p74)	rate_local4 =0.126-0.963 (p14-84)	93.13	0.86	19.08	0.02	1.489E-05
**234**	gender_ratio >87.528 (p11) &prop_preprim_edu <0.146 (p89) &LU_entropy >0.508 (p36)	rate_local4 =0.069-1.113 (p13-88)	98.58	0.90	18.87	0.04	1.893E-07
**235**	prop_woodland >0.072 (p38) &POI_pp_sports <33.191 (p70)	rate_local4 =0.119-0.888 (p14-81)	80.89	0.87	18.82	0.09	2.517E-05
**236**	ave_household_size >2.627 (p29) &POI_pp_mall_mkt <12.788 (p81)	rate_local4 =0.152-0.892 (p15-81)	100.45	0.81	18.70	0.07	5.27E-05
**237**	den_bldg <0.331 (p77) &POI_pp_telecom_elec <16.177 (p59) &POI_pp_transport <39.095 (p61)	rate_local4 =0.040-1.093 (p13-87)	84.80	0.94	18.66	0.01	2.567E-05
**238**	gender_ratio >87.552 (p11) &POI_pp_sports <38.723 (p76)	rate_local4 =0.084-1.253 (p13-90)	126.70	0.90	18.66	0.02	5.313E-05
**239**	prop_preprim_edu <0.152 (p91) &POI_pp_transport <29.486 (p52)	rate_local4 =0.125-1.126 (p14-88)	96.15	0.91	18.65	0.02	9.1E-06
**240**	gender_ratio >87.552 (p11) &POI_pp_edu <14.961 (p67)	rate_local4 =0.084-1.253 (p13-90)	115.85	0.91	18.64	0.02	8.192E-05
**241**	POI_pp_sports <33.191 (p70)	rate_local4 =0.062-1.249 (p13-90)	133.04	0.89	18.50	0.12	3.4E-12
**242**	ave_household_size >2.683 (p29) &POI_pp_telecom_elec <17.911 (p64) &POI_pp_mall_mkt <11.035 (p75)	rate_local4 =0.075-1.088 (p13-87)	85.52	0.93	18.40	0.01	7.588E-05

243	prop_preprim_edu <0.144 (p89) &ave_household_size >2.509 (p17) &prop_household_3gen <0.081 (p92)	rate_local4 =0.078-1.234 (p13-90)	130.76	0.89	18.32	0.02	4.511E-05
244	prop_preprim_edu <0.140 (p86) &den_public_trans <64.180 (p85)	rate_local4 =0.152-0.892 (p15-81)	118.61	0.77	18.20	0.05	5.411E-06
245	prop_business <0.060 (p87) &POI_pp_mall_mkt <14.399 (p83)	rate_local4 =0.145-0.943 (p15-83)	121.83	0.80	18.18	0.04	6.386E-05
246	prop_woodland >0.065 (p36) &POI_pp_telecom_elec <16.538 (p60)	rate_local4 =0.088-0.877 (p13-80)	72.87	0.88	17.95	0.10	2.292E-05
247	POI_pp_transport <68.907 (p80)	rate_local4 =0.048-1.284 (p13-90)	149.12	0.87	17.83	0.10	2.154E-14
248	prop_preprim_edu <0.138 (p85) &prop_business <0.050 (p86)	rate_local4 =0.152-0.892 (p15-81)	119.03	0.77	17.71	0.06	2.441E-05
249	gender_ratio >87.924 (p11) &LU_entropy >0.522 (p40)	rate_local4 =0.119-0.888 (p14-81)	92.35	0.82	17.69	0.03	2.472E-05
250	prop_preprim_edu <0.149 (p90) &ave_household_size >2.627 (p29)	rate_local4 =0.152-0.892 (p15-81)	111.36	0.78	17.55	0.04	3.074E-05
251	prop_private_resid =0.013-0.123 (p30-70) &POI_pp_transport <66.085 (p78)	rate_local4 =0.124-0.575 (p14-62)	51.61	0.70	17.55	0.09	3.873E-05
252	LU_entropy >0.548 (p47)	rate_local4 =0.140-0.863 (p14-80)	90.44	0.80	17.54	0.15	3.552E-08
253	prop_woodland >0.065 (p36) &POI_pp_edu <19.430 (p74)	rate_local4 =0.050-0.879 (p13-81)	85.14	0.84	17.50	0.08	6.634E-05
254	POI_pp_mall_mkt <6.232 (p55)	rate_local4 =0.071-1.079 (p13-87)	103.59	0.88	17.43	0.15	3.721E-08
255	prop_private_resid =0.013-0.123 (p30-70) &POI_pp_telecom_elec <31.454 (p80)	rate_local4 =0.124-0.575 (p14-62)	51.03	0.69	17.28	0.09	8.372E-06
256	gender_ratio >87.552 (p11) &POI_pp_mall_mkt <10.886 (p75)	rate_local4 =0.080-0.857 (p13-80)	108.46	0.78	17.19	0.03	7.21E-06
257	prop_preprim_edu <0.136 (p85) &prop_household_3gen <0.081 (p92) &prop_business <0.044 (p85)	rate_local4 =0.078-1.234 (p13-90)	124.09	0.88	16.97	0.02	6.113E-05
258	gender_ratio >87.664 (p11) &ave_household_size >2.732 (p37)	rate_local4 =0.132-0.855 (p14-80)	95.82	0.79	16.87	0.04	1.574E-05
259	POI_pp_telecom_elec <30.272 (p80)	rate_local4 =0.039-1.211 (p13-90)	145.90	0.85	16.73	0.10	8.204E-12
260	ave_household_size >2.654 (p29) &prop_household_3gen <0.060 (p86)	rate_local4 =0.062-0.869 (p13-80)	100.40	0.78	16.68	0.05	5.797E-05
261	prop_grassland =0.004-0.085 (p23-71) &POI_pp_telecom_elec <26.772 (p77)	rate_local4 =0.188-0.848 (p16-80)	66.62	0.82	16.40	0.09	1.936E-05
262	prop_private_resid =0.008-0.104 (p26-66) &POI_pp_edu <16.203 (p69)	rate_local4 =0.062-0.820 (p13-78)	58.99	0.88	16.34	0.11	7.753E-05
263	prop_household_3gen <0.081 (p92) &den_public_trans <56.620 (p82)	rate_local4 =0.156-0.860 (p15-80)	116.83	0.73	15.92	0.03	3.957E-05
264	prop_household_3gen <0.052 (p81) &den_bldg <0.435 (p83)	rate_local4 =0.113-0.833 (p13-79)	105.04	0.76	15.92	0.06	4.932E-05
265	prop_preprim_edu <0.149 (p90) &den_bldg <0.328 (p76)	rate_local4 =0.152-0.892 (p15-81)	107.85	0.77	15.60	0.05	7.199E-05
266	prop_grassland =0.007-0.073 (p30-69) &POI_pp_mall_mkt <9.685 (p69)	rate_local4 =0.218-1.005 (p21-86)	52.57	0.91	15.51	0.15	1.497E-05
267	den_road <16.676 (p72) &prop_transport >0.067 (p35)	rate_local4 =0.178-1.014 (p16-86)	70.86	0.87	15.30	0.12	6.16E-06
268	prop_business <0.060 (p87) &prop_transport >0.067 (p35)	rate_local4 =0.184-1.158 (p16-89)	94.98	0.84	15.23	0.09	3.549E-05
269	prop_grassland =0.007-0.073 (p30-69) &POI_pp_transport <42.169 (p64)	rate_local4 =0.218-1.005 (p21-86)	50.86	0.91	15.09	0.15	5.184E-05

**270**	gender_ratio >87.664 (p11) &den_road <23.313 (p87)	rate_local4 =0.132-0.855 (p14-80)	119.41	0.74	14.92	0.04	3.003E-05
**271**	POI_pp_edu <5.310 (p35)	rate_local4 =0.197-0.642 (p16-65)	48.35	0.68	14.66	0.21	2.402E-05
**272**	prop_grassland =0.007-0.073 (p30-69) &POI_pp_sports <43.022 (p80)	rate_local4 =0.218-1.005 (p21-86)	55.78	0.87	14.58	0.11	7.826E-05
**273**	gender_ratio >87.552 (p11) &prop_business <0.055 (p86)	rate_local4 =0.124-0.871 (p14-80)	121.86	0.75	14.36	0.03	6.16E-06
**274**	prop_transport >0.104 (p44) &LU_entropy >0.535 (p43)	rate_local4 =0.170-1.002 (p16-86)	59.50	0.88	14.12	0.08	2.237E-05
**275**	ave_household_size >2.713 (p37)	rate_local4 =0.119-0.865 (p14-80)	106.31	0.76	14.06	0.10	4.393E-07
**276**	prop_household_3gen <0.081 (p92) &den_road <15.967 (p70)	rate_local4 =0.142-0.793 (p14-77)	93.08	0.72	13.83	0.04	7.901E-05
**277**	gender_ratio >87.277 (p11) &prop_preprim_edu <0.128 (p82)	rate_local4 =0.083-1.256 (p13-90)	131.00	0.85	13.68	0.04	1.048E-05
**278**	prop_grassland <0.027 (p51) &LU_entropy >0.551 (p49)	rate_local4 =0.305-0.905 (p29-81)	37.33	0.79	13.04	0.17	4.997E-05
**279**	prop_transport =0.035-0.151 (p25-57)	rate_local4 =0.186-0.534 (p16-56)	39.08	0.57	12.25	0.18	2.687E-06
**280**	prop_preprim_edu <0.136 (p85) &prop_household_3gen <0.081 (p92)	rate_local4 =0.078-1.234 (p13-90)	140.09	0.83	12.04	0.03	4.293E-05
**281**	prop_grassland =0.004-0.085 (p23-71)	rate_local4 =0.188-0.848 (p16-80)	72.91	0.73	11.18	0.11	7.233E-05
**282**	prop_private_resid =0.022-0.087 (p36-64)	rate_local4 =0.117-0.731 (p14-73)	43.28	0.77	10.72	0.19	7.179E-06
**283**	prop_publicResid >0.042 (p70)	rate_local4 =0.168-1.073 (p16-87)	56.16	0.86	10.24	0.16	3.66E-05
**284**	prop_preprim_edu <0.134 (p84)	rate_local4 =0.081-1.273 (p13-90)	147.41	0.82	9.14	0.05	8.833E-06
**285**	prop_woodland <0.016 (p25)	rate_local4 =0.744-1.282 (p74-90)	18.16	0.34	9.13	0.17	3.327E-05
**286**	prop_gov_insti_faci >0.033 (p43)	rate_local4 =0.027-1.271 (p13-90)	103.73	0.84	8.92	0.07	6.359E-05
**287**	prop_household_3gen <0.081 (p92)	rate_local4 =0.041-1.292 (p13-90)	158.15	0.81	7.35	0.04	1.62E-06
**288**	den_road <20.368 (p81) &den_public_trans <47.014 (p78)	rate_local4 <0.727 (p73)	131.03	0.86	21.34	0.04	5.871E-08
**289**	den_road <16.383 (p71)	rate_local4 <0.722 (p72)	128.58	0.84	19.36	0.13	4.628E-12
**290**	den_population <37522.136 (p69) &prop_business <0.036 (p83)	rate_local4 <0.727 (p73)	113.33	0.87	18.94	0.04	1.498E-06
**291**	den_population <36962.203 (p68) &den_public_trans <32.715 (p66)	rate_local4 <0.716 (p71)	105.49	0.87	18.91	0.04	3.106E-05
**292**	den_bldg <0.226 (p62)	rate_local4 <0.603 (p64)	99.30	0.76	18.44	0.14	3.089E-07
**293**	den_population <8644.075 (p40) &POI_pp_sports >29.162 (p66)	rate_local4 <0.070 (p13)	23.55	0.53	17.73	0.20	5.63E-06
**294**	den_population <26219.620 (p60)	rate_local4 <0.466 (p46)	75.80	0.61	16.78	0.14	1.213E-06
**295**	den_population <8644.075 (p40) &POI_pp_transport >54.345 (p73)	rate_local4 <0.053 (p13)	21.67	0.56	16.63	0.20	7.284E-06
**296**	den_population <11819.200 (p43) &POI_pp_telecom_elec >17.798 (p64)	rate_local4 <0.080 (p13)	22.86	0.43	15.95	0.13	4.768E-05
**297**	prop_shrubland >0.064 (p49)	rate_local4 <0.785 (p76)	101.16	0.90	15.67	0.14	2.934E-07
**298**	den_population <8644.075 (p40) &POI_pp_edu >16.779 (p69)	rate_local4 <0.035 (p13)	20.53	0.54	15.55	0.21	4.103E-05
**299**	prop_private_resid <0.069 (p57) &prop_publicResid <0.039 (p70)	rate_local4 <0.191 (p16)	29.04	0.36	15.42	0.11	1.535E-05
**300**	den_public_trans <46.130 (p78)	rate_local4 <0.780 (p76)	136.41	0.85	15.26	0.10	4.312E-10
**301**	den_population <8644.075 (p40) &POI_pp_mall_mkt >9.300 (p66)	rate_local4 <0.058 (p13)	19.87	0.55	15.16	0.22	4.103E-05
**302**	prop_private_resid <0.025 (p39) &POI_pp_sports >25.381 (p62)	rate_local4 <0.072 (p13)	20.28	0.50	15.01	0.21	1.012E-05
**303**	prop_woodland =0.016-0.309 (p25-81)	rate_local4 <0.744 (p74)	100.70	0.86	14.25	0.12	1.391E-06
**304**	POI_pp_sports >33.191 (p70)	rate_local4 <0.062 (p13)	22.58	0.35	14.17	0.22	8.477E-12

**305**	POI_pp_telecom_elec >30.272 (p80)	rate_local4 <0.039 (p13)	19.74	0.45	14.04	0.32	3.555E-13
**306**	POI_pp_transport >68.907 (p80)	rate_local4 <0.048 (p13)	19.71	0.45	14.01	0.32	7.438E-15
**307**	den_bldg <0.160 (p47) &POI_pp_mall_mkt >7.205 (p60)	rate_local4 <0.075 (p13)	19.21	0.48	14.00	0.23	2.826E-06
**308**	prop_agricultural >0.017 (p64)	rate_local4 <0.430 (p43)	46.73	0.61	13.80	0.18	5.866E-06
**309**	prop_private_resid <0.025 (p39) &POI_pp_mall_mkt >7.305 (p60)	rate_local4 <0.148 (p15)	18.82	0.51	13.44	0.21	7.541E-06
**310**	prop_business <0.058 (p86)	rate_local4 <0.784 (p76)	152.27	0.82	11.69	0.06	1.455E-08
**311**	prop_private_resid <0.022 (p36)	rate_local4 <0.117 (p14)	22.60	0.29	11.64	0.15	2.503E-08
**312**	prop_transport <0.035 (p25)	rate_local4 <0.186 (p16)	20.05	0.39	11.58	0.22	8.801E-07
**313**	prop_rural_set >0.032 (p69)	rate_local4 <0.348 (p36)	35.01	0.51	10.90	0.16	6.869E-05
**314**	prop_household_3gen >0.052 (p81) &den_population <11819.200 (p43)	rate_local4 <0.080 (p13)	14.30	0.54	10.86	0.21	2.837E-05
**315**	POI_pp_edu >25.063 (p83)	rate_local4 <0.061 (p13)	15.92	0.40	10.72	0.27	2.209E-09
**316**	prop_rural_set >0.032 (p69) &POI_pp_sports >19.775 (p53)	rate_local4 <0.062 (p13)	14.85	0.45	10.52	0.20	6.233E-05
**317**	prop_gov_insti_faci <0.033 (p43)	rate_local4 <0.027 (p13)	21.99	0.24	10.15	0.11	9.447E-06
**318**	prop_open_recreation <0.006 (p25)	rate_local4 <0.065 (p13)	16.70	0.32	9.76	0.18	2.281E-06
**319**	med_area_home >15.641 (p45)	rate_local4 <0.299 (p29)	45.56	0.37	9.68	0.08	7.299E-05
**320**	build_area_pp >58.100 (p47)	rate_local4 <0.222 (p21)	36.65	0.31	9.66	0.08	1.522E-05
**321**	prop_grassland >0.085 (p71)	rate_local4 <0.188 (p16)	20.40	0.32	9.55	0.15	6.504E-06
**322**	prop_rural_set >0.024 (p65) &POI_pp_mall_mkt >10.138 (p71)	rate_local4 <0.067 (p13)	11.97	0.64	9.51	0.33	3.838E-05
**323**	POI_pp_mall_mkt >19.264 (p88)	rate_local4 <0.071 (p13)	12.77	0.48	9.31	0.35	2.595E-09
**324**	prop_publicResid <0.042 (p70)	rate_local4 <0.168 (p16)	32.56	0.22	8.66	0.06	3.282E-05
**325**	prop_preprim_edu >0.156 (p92)	rate_local4 <0.081 (p13)	11.00	0.60	8.62	0.47	2.726E-10
**326**	prop_household_3gen >0.081 (p92)	rate_local4 <0.041 (p13)	10.67	0.59	8.30	0.46	4.761E-11
**327**	prop_over65 >0.229 (p93)	rate_local4 <0.299 (p29)	11.05	0.78	6.81	0.48	2.318E-05
**328**	gender_ratio >109.623 (p94)	rate_local4 <0.334 (p34)	10.16	0.79	5.84	0.45	5.987E-05
**329**	gender_ratio <87.552 (p11) &prop_grassland >0.070 (p67)	rate_local4 <0.078 (p13)	6.00	0.98	5.20	0.69	1.336E-05
**330**	gender_ratio <86.999 (p11) &den_population <11819.200 (p43)	rate_local4 <0.089 (p13)	6.25	0.77	5.19	0.47	3.162E-05
**331**	gender_ratio <87.087 (p11) &den_public_trans <6.868 (p34)	rate_local4 <0.032 (p13)	5.56	0.94	4.79	0.65	5.737E-05
**332**	gender_ratio <87.552 (p11) &den_bldg <0.112 (p36)	rate_local4 <0.078 (p13)	5.47	0.98	4.74	0.69	4.75E-05

(e) Rules for wave-3 local case rate, excluding cases in the Dancing/Singing Cluster; POI accessibility was used

No.	Antecedent	Consequent	Supp	Conf	Lev	Imp	*P*
**1**	med_income <23893.698 (p79) &prop_rural_set <0.020 (p64) &prop_shrubland <0.106 (p57)	rate_local4nondance >0.421 (p58)	50.22	0.79	22.55	0.14	7E-07
**2**	med_income <26997.433 (p83) &prop_agricultural <0.018 (p64) &prop_shrubland <0.106 (p57)	rate_local4nondance >0.417 (p58)	56.31	0.72	22.15	0.09	1.9E-05
**3**	med_income <27021.574 (p84) &prop_rural_set <0.014 (p59) &prop_transport >0.089 (p39)	rate_local4nondance >0.352 (p48)	59.83	0.83	21.69	0.10	5.3E-05
**4**	med_income <27548.320 (p84) &den_road >7.805 (p37) &prop_agricultural <0.024 (p71)	rate_local4nondance >0.339 (p44)	70.57	0.78	21.45	0.07	3.1E-05
**5**	med_income <26534.581 (p83) &prop_rural_set <0.020 (p64) &prop_grassland <0.048 (p61)	rate_local4nondance >0.421 (p58)	51.93	0.73	21.24	0.12	5.6E-05
**6**	med_income <27021.574 (p84) &den_road >7.987 (p38) &prop_rural_set <0.032 (p69)	rate_local4nondance >0.352 (p48)	62.75	0.80	21.21	0.10	3.1E-05
**7**	prop_higher_edu <0.354 (p84) &prop_rural_set <0.018 (p63) &prop_shrubland <0.114 (p58)	rate_local4nondance >0.385 (p52)	53.12	0.80	21.19	0.12	2.4E-05
**8**	med_income <23378.274 (p78) &prop_transport >0.130 (p52) &prop_agricultural <0.012 (p60)	rate_local4nondance >0.338 (p44)	58.69	0.85	21.01	0.07	8.3E-05
**9**	med_income <28521.509 (p84) &den_bldg >0.118 (p38) &prop_shrubland <0.131 (p64)	rate_local4nondance >0.342 (p45)	71.66	0.76	20.96	0.06	1.6E-05
**10**	med_income <28309.145 (p84) &den_public_trans >17.077 (p50) &prop_shrubland <0.107 (p57)	rate_local4nondance >0.408 (p56)	55.63	0.72	20.96	0.08	7.9E-05
**11**	med_income <28532.667 (p84) &prop_transport >0.140 (p54)	rate_local4nondance >0.412 (p57)	57.31	0.70	20.80	0.08	6.8E-05
**12**	med_income <23455.266 (p78) &den_bldg >0.180 (p51)	rate_local4nondance >0.411 (p57)	54.28	0.72	20.67	0.12	7.1E-07
**13**	med_income <24026.190 (p79) &prop_rural_set <0.002 (p49)	rate_local4nondance >0.397 (p54)	54.12	0.75	20.39	0.14	1.3E-05
**14**	med_area_home <17.602 (p54) &prop_rural_set <0.012 (p59)	rate_local4nondance >0.396 (p53)	56.33	0.73	20.34	0.14	1.2E-05
**15**	med_income <24039.820 (p79) &den_road >11.532 (p53)	rate_local4nondance >0.421 (p58)	52.05	0.71	20.14	0.12	3E-06
**16**	med_income <23493.899 (p78) &prop_agricultural <0.000 (p40)	rate_local4nondance >0.411 (p57)	46.02	0.78	19.88	0.16	3.5E-07
**17**	den_population >18758.717 (p50) &prop_agricultural <0.016 (p64)	rate_local4nondance >0.348 (p47)	69.20	0.75	19.73	0.05	4.8E-05
**18**	med_area_home <18.912 (p57) &prop_rural_set <0.018 (p63) &prop_shrubland <0.104 (p56)	rate_local4nondance >0.348 (p46)	52.19	0.86	19.66	0.11	8.2E-05
**19**	med_income <26688.145 (p83) &den_road >7.671 (p36) &prop_shrubland <0.145 (p67)	rate_local4nondance >0.342 (p45)	70.54	0.75	19.65	0.04	3.2E-05
**20**	med_income <27897.286 (p84) &prop_grassland <0.035 (p58)	rate_local4nondance >0.400 (p55)	64.71	0.66	19.46	0.08	5.9E-05
**21**	med_area_home <17.602 (p54) &prop_agricultural <0.014 (p61)	rate_local4nondance >0.363 (p49)	63.10	0.74	19.33	0.10	4.4E-05

**22**	med_income <23893.698 (p79) &prop_transport >0.056 (p33) &LU_entropy <0.546 (p47)	rate_local4nondance >0.621 (p74)	30.15	0.71	19.29	0.17	5.1E-05
**23**	prop_higher_edu <0.342 (p81) &den_road >11.532 (p53) &prop_rural_set <0.010 (p59)	rate_local4nondance >0.421 (p58)	44.45	0.76	19.29	0.09	6.4E-05
**24**	med_income <23361.519 (p78) &den_road >6.327 (p30) &LU_entropy <0.538 (p44)	rate_local4nondance >0.596 (p74)	29.78	0.75	19.05	0.21	1.7E-06
**25**	med_area_home <18.088 (p55) &den_bldg >0.187 (p53)	rate_local4nondance >0.421 (p58)	47.62	0.72	19.04	0.12	5.7E-06
**26**	prop_rural_set <0.035 (p70) &prop_shrubland <0.104 (p56)	rate_local4nondance >0.343 (p46)	68.19	0.75	18.94	0.08	3.1E-05
**27**	prop_transport >0.116 (p50) &LU_entropy <0.550 (p49)	rate_local4nondance >0.674 (p77)	31.51	0.58	18.91	0.23	6.6E-07
**28**	med_income <28202.741 (p84) &den_public_trans >23.601 (p57)	rate_local4nondance >0.357 (p48)	60.54	0.76	18.85	0.06	4.8E-05
**29**	med_area_home <18.912 (p57) &prop_shrubland <0.104 (p56)	rate_local4nondance >0.417 (p58)	54.20	0.67	18.67	0.10	7E-05
**30**	den_road >9.857 (p46) &LU_entropy <0.551 (p49)	rate_local4nondance >0.687 (p78)	31.83	0.54	18.64	0.20	6.9E-06
**31**	prop_household_3gen <0.081 (p92) &med_area_home <17.219 (p52) &prop_rural_set <0.041 (p71)	rate_local4nondance >0.263 (p35)	72.22	0.87	18.63	0.03	3.5E-05
**32**	den_population >18148.394 (p50) &prop_rural_set <0.041 (p71)	rate_local4nondance >0.263 (p35)	78.68	0.84	18.60	0.04	4.6E-05
**33**	prop_agricultural <0.028 (p73) &POI_pp_telecom_elec <20.290 (p69)	rate_local4nondance >0.234 (p31)	95.14	0.84	18.54	0.06	5E-05
**34**	med_income <23361.519 (p78) &prop_agricultural <0.009 (p58) &LU_entropy <0.547 (p47)	rate_local4nondance >0.623 (p74)	29.97	0.67	18.54	0.19	5.8E-06
**35**	med_income <27081.831 (p84) &med_area_home <18.198 (p55) &prop_rural_set <0.012 (p59)	rate_local4nondance >0.259 (p35)	68.92	0.89	18.50	0.02	4.1E-05
**36**	med_income <28202.741 (p84) &prop_shrubland <0.071 (p50)	rate_local4nondance >0.406 (p56)	57.96	0.66	18.45	0.08	3.4E-05
**37**	med_income <23361.519 (p78) &den_bldg >0.144 (p43) &LU_entropy <0.538 (p44)	rate_local4nondance >0.596 (p74)	28.51	0.76	18.40	0.19	1.1E-05
**38**	den_bldg >0.158 (p47) &LU_entropy <0.550 (p49)	rate_local4nondance >0.684 (p78)	31.81	0.54	18.40	0.18	3.1E-05
**39**	med_area_home <17.981 (p55) &prop_grassland <0.022 (p46) &LU_entropy <0.550 (p49)	rate_local4nondance >0.711 (p79)	25.42	0.77	18.33	0.20	4.1E-05
**40**	ave_household_size <2.963 (p63) &prop_rural_set <0.004 (p51) &LU_entropy <0.555 (p50)	rate_local4nondance >0.647 (p76)	30.06	0.62	18.30	0.14	8.2E-05
**41**	med_income <23361.519 (p78) &prop_grassland <0.040 (p59) &LU_entropy <0.538 (p44)	rate_local4nondance >0.596 (p74)	29.38	0.71	18.30	0.18	9.6E-06
**42**	prop_higher_edu <0.346 (p82) &prop_rural_set <0.000 (p43)	rate_local4nondance >0.389 (p53)	48.36	0.76	18.27	0.14	2.4E-05
**43**	med_area_home <17.728 (p54) &den_road >11.019 (p51)	rate_local4nondance >0.426 (p59)	48.39	0.69	18.27	0.11	7E-05
**44**	den_population >19719.011 (p51) &LU_entropy <0.550 (p49)	rate_local4nondance >0.691 (p78)	28.99	0.60	18.20	0.24	3.2E-07

**45**	den_population >26714.031 (p60) &prop_agricultural <0.015 (p62) &prop_shrubland <0.123 (p61)	rate_local4nondance >0.297 (p37)	59.67	0.84	18.20	0.03	1.1E-05
**46**	prop_grassland <0.025 (p50) &LU_entropy <0.550 (p49)	rate_local4nondance >0.652 (p76)	32.64	0.54	18.17	0.18	4.8E-06
**47**	den_population >26714.031 (p60) &prop_shrubland <0.123 (p61)	rate_local4nondance >0.284 (p37)	66.15	0.85	18.13	0.03	3.7E-05
**48**	med_income <23361.519 (p78) &prop_shrubland <0.115 (p58) &LU_entropy <0.538 (p44)	rate_local4nondance >0.596 (p74)	28.93	0.72	18.05	0.17	2.5E-05
**49**	prop_agricultural <0.035 (p75) &POI_pp_transport <50.430 (p71)	rate_local4nondance >0.234 (p31)	96.23	0.83	18.02	0.07	3.1E-06
**50**	den_population >31290.715 (p63) &den_bldg >0.179 (p51)	rate_local4nondance >0.447 (p61)	44.12	0.68	18.01	0.06	5.3E-05
**51**	med_area_home <17.981 (p55) &prop_shrubland <0.122 (p60) &LU_entropy <0.550 (p49)	rate_local4nondance >0.716 (p79)	25.15	0.75	17.98	0.18	4.9E-05
**52**	med_area_home <17.981 (p55) &den_bldg >0.140 (p43) &LU_entropy <0.550 (p49)	rate_local4nondance >0.623 (p74)	26.70	0.78	17.95	0.17	5.4E-05
**53**	med_income <21465.840 (p75) &prop_rural_set <0.004 (p51) &LU_entropy <0.550 (p49)	rate_local4nondance >0.615 (p74)	28.12	0.72	17.94	0.21	1.1E-05
**54**	prop_higher_edu <0.342 (p81) &den_road >11.532 (p53)	rate_local4nondance >0.421 (p58)	49.83	0.68	17.91	0.09	2.4E-05
**55**	prop_rural_set <0.055 (p75) &prop_transport >0.062 (p35)	rate_local4nondance >0.292 (p37)	89.28	0.76	17.81	0.06	6.2E-05
**56**	prop_shrubland <0.021 (p37) &LU_entropy <0.545 (p46)	rate_local4nondance >0.642 (p76)	30.47	0.59	17.81	0.17	3.4E-07
**57**	prop_preprim_edu <0.156 (p92) &med_area_home <17.524 (p53) &prop_agricultural <0.015 (p62)	rate_local4nondance >0.221 (p30)	75.59	0.90	17.76	0.02	3.1E-06
**58**	den_population >26428.002 (p60)	rate_local4nondance >0.376 (p51)	62.01	0.69	17.69	0.20	3.2E-07
**59**	prop_rural_set <0.037 (p70) &POI_pp_telecom_elec <20.290 (p69)	rate_local4nondance >0.258 (p35)	86.12	0.82	17.66	0.07	2.1E-05
**60**	prop_woodland <0.072 (p38) &LU_entropy <0.543 (p46)	rate_local4nondance >0.656 (p76)	29.67	0.59	17.62	0.18	2.4E-06
**61**	prop_higher_edu <0.346 (p82) &prop_agricultural <0.002 (p46)	rate_local4nondance >0.418 (p58)	48.65	0.68	17.58	0.11	1.8E-05
**62**	med_area_home <17.610 (p54) &den_road >11.808 (p55) &LU_entropy <0.541 (p45)	rate_local4nondance >0.738 (p79)	23.76	0.77	17.51	0.19	4E-05
**63**	prop_higher_edu <0.338 (p81) &med_area_home <18.198 (p55) &prop_rural_set <0.012 (p59)	rate_local4nondance >0.253 (p34)	66.46	0.89	17.49	0.02	3.5E-05
**64**	build_area_pp <73.548 (p54) &prop_rural_set <0.044 (p73) &POI_pp_transport <50.430 (p71)	rate_local4nondance >0.234 (p31)	67.87	0.91	17.48	0.05	4.8E-05
**65**	prop_higher_edu <0.342 (p81) &den_road >9.857 (p46) &LU_entropy <0.551 (p49)	rate_local4nondance >0.760 (p80)	24.71	0.66	17.46	0.16	4E-05
**66**	prop_preprim_edu <0.123 (p80) &POI_pp_transport <45.027 (p69)	rate_local4nondance >0.172 (p26)	103.99	0.88	17.34	0.05	7.9E-06

**67**	prop_rural_set <0.044 (p73) &POI_pp_transport <50.430 (p71)	rate_local4nondance >0.234 (p31)	90.88	0.84	17.32	0.08	2.8E-05
**68**	med_area_home <18.503 (p56) &prop_transport >0.090 (p39) &LU_entropy <0.537 (p44)	rate_local4nondance >0.721 (p79)	24.18	0.74	17.27	0.17	7.7E-05
**69**	prop_preprim_edu <0.153 (p91) &med_area_home <17.090 (p52)	rate_local4nondance >0.314 (p40)	76.10	0.75	17.20	0.05	6.4E-05
**70**	prop_transport >0.143 (p55)	rate_local4nondance >0.419 (p58)	59.22	0.61	17.17	0.18	9.2E-08
**71**	med_area_home <18.503 (p56) &den_population >15207.279 (p47) &LU_entropy <0.555 (p50)	rate_local4nondance >0.781 (p82)	23.93	0.64	17.06	0.14	2.1E-05
**72**	build_area_pp <89.773 (p62) &prop_rural_set <0.078 (p82) &prop_transport >0.062 (p35)	rate_local4nondance >0.202 (p27)	75.22	0.92	17.05	0.06	1.1E-05
**73**	med_area_home <18.727 (p57) &prop_woodland <0.064 (p36) &LU_entropy <0.543 (p46)	rate_local4nondance >0.656 (p76)	24.79	0.76	17.04	0.17	4.7E-05
**74**	prop_rural_set <0.003 (p50) &LU_entropy <0.550 (p49)	rate_local4nondance >0.710 (p79)	31.06	0.48	17.03	0.14	4.1E-05
**75**	build_area_pp <89.773 (p62) &prop_rural_set <0.067 (p80) &prop_agricultural <0.013 (p61)	rate_local4nondance >0.207 (p29)	72.28	0.92	16.90	0.02	1.4E-05
**76**	build_area_pp <57.599 (p46) &prop_agricultural <0.009 (p59)	rate_local4nondance >0.284 (p37)	57.58	0.87	16.89	0.12	1.6E-05
**77**	prop_transport >0.148 (p55) &prop_agricultural <0.028 (p73)	rate_local4nondance >0.296 (p37)	69.77	0.78	16.66	0.02	5.6E-05
**78**	den_road >6.756 (p31) &build_area_pp <87.950 (p62) &prop_rural_set <0.052 (p75)	rate_local4nondance >0.189 (p27)	69.90	0.94	16.65	0.07	2.1E-07
**79**	med_area_home <16.629 (p50) &LU_entropy <0.550 (p49)	rate_local4nondance >0.607 (p74)	27.40	0.67	16.65	0.29	1.7E-07
**80**	med_area_home <17.728 (p54) &den_road >7.987 (p38) &prop_rural_set <0.049 (p74)	rate_local4nondance >0.235 (p31)	65.45	0.91	16.64	0.06	2.3E-05
**81**	prop_preprim_edu <0.123 (p80) &POI_pp_sports <37.106 (p74)	rate_local4nondance >0.172 (p26)	113.01	0.86	16.53	0.04	2.6E-05
**82**	build_area_pp <84.798 (p59) &prop_rural_set <0.039 (p71) &POI_pp_telecom_elec <29.624 (p80)	rate_local4nondance >0.198 (p27)	75.65	0.91	16.46	0.04	7.3E-05
**83**	prop_over65 <0.212 (p89) &med_area_home <17.531 (p53)	rate_local4nondance >0.294 (p37)	74.90	0.77	16.46	0.06	3E-05
**84**	prop_preprim_edu <0.144 (p89) &med_area_home <17.727 (p54) &POI_pp_edu <24.537 (p82)	rate_local4nondance >0.172 (p26)	84.61	0.91	16.46	0.03	5E-05
**85**	prop_preprim_edu <0.144 (p89) &med_area_home <17.727 (p54) &POI_pp_mall_mkt <10.119 (p71)	rate_local4nondance >0.172 (p26)	80.32	0.92	16.41	0.04	7.7E-05
**86**	prop_grassland <0.001 (p17)	rate_local4nondance >0.693 (p78)	24.54	0.67	16.40	0.45	1.7E-13
**87**	med_area_home <17.524 (p53) &prop_agricultural <0.015 (p62) &POI_pp_edu <19.208 (p73)	rate_local4nondance >0.221 (p30)	65.80	0.92	16.38	0.04	1.4E-05
**88**	den_bldg >0.116 (p37) &prop_shrubland <0.134 (p64)	rate_local4nondance >0.297 (p37)	87.76	0.74	16.38	0.04	4.8E-05
**89**	ave_household_size <2.831 (p49) &prop_agricultural <0.001 (p43)	rate_local4nondance >0.626 (p74)	29.32	0.57	16.36	0.18	3.2E-05

**90**	prop_preprim_edu <0.144 (p89) &prop_household_3gen <0.062 (p86) &med_area_home <17.727 (p54)	rate_local4nondance >0.172 (p26)	86.57	0.90	16.27	0.02	5E-05
**91**	build_area_pp <56.323 (p46) &prop_rural_set <0.010 (p58)	rate_local4nondance >0.311 (p40)	52.41	0.85	16.25	0.13	7.3E-05
**92**	build_area_pp <86.576 (p61) &prop_rural_set <0.044 (p73) &prop_shrubland <0.151 (p69)	rate_local4nondance >0.234 (p31)	60.89	0.92	16.19	0.09	6.2E-05
**93**	prop_over65 <0.213 (p89) &med_area_home <17.069 (p52) &prop_rural_set <0.031 (p69)	rate_local4nondance >0.193 (p27)	68.66	0.94	16.19	0.04	2.6E-05
**94**	prop_household_3gen <0.081 (p92) &med_area_home <16.834 (p50)	rate_local4nondance >0.263 (p35)	80.97	0.81	16.17	0.04	2.1E-05
**95**	med_area_home <17.408 (p52) &prop_grassland <0.004 (p26)	rate_local4nondance >0.792 (p82)	22.13	0.67	16.15	0.20	4.3E-05
**96**	med_area_home <15.828 (p46) &prop_agricultural <0.009 (p59) &POI_pp_mall_mkt <11.754 (p77)	rate_local4nondance >0.261 (p35)	55.86	0.91	16.09	0.03	7.1E-05
**97**	ave_household_size <2.814 (p49) &prop_rural_set <0.004 (p51)	rate_local4nondance >0.568 (p72)	32.20	0.57	16.02	0.17	6.2E-05
**98**	med_area_home <15.809 (p46) &POI_pp_edu <22.066 (p79)	rate_local4nondance >0.228 (p31)	76.23	0.86	16.01	0.04	2.9E-05
**99**	prop_private_resid >0.061 (p55) &prop_woodland <0.101 (p49)	rate_local4nondance >0.700 (p78)	29.37	0.48	15.99	0.14	8E-05
**100**	prop_agricultural <0.034 (p75) &POI_pp_edu <21.430 (p79)	rate_local4nondance >0.191 (p27)	103.74	0.85	15.94	0.06	3.9E-05
**101**	den_public_trans >16.704 (p50) &LU_entropy <0.550 (p49)	rate_local4nondance >0.768 (p81)	26.39	0.48	15.92	0.18	1.2E-07
**102**	prop_preprim_edu <0.123 (p80) &POI_pp_telecom_elec <30.383 (p80)	rate_local4nondance >0.172 (p26)	120.64	0.84	15.88	0.04	7E-05
**103**	prop_agricultural <0.018 (p64) &POI_pp_sports <29.408 (p66)	rate_local4nondance >0.200 (p27)	83.29	0.88	15.87	0.07	7.5E-05
**104**	med_income <20991.546 (p73) &prop_private_resid >0.102 (p66)	rate_local4nondance >0.709 (p79)	24.81	0.60	15.86	0.20	2.6E-05
**105**	med_area_home <15.434 (p45) &POI_pp_mall_mkt <12.467 (p79)	rate_local4nondance >0.182 (p26)	77.87	0.91	15.73	0.04	1.1E-05
**106**	med_income <23361.519 (p78) &prop_private_resid >0.055 (p53) &LU_entropy <0.547 (p47)	rate_local4nondance >0.670 (p77)	23.32	0.71	15.73	0.21	2.6E-05
**107**	med_income <25216.681 (p81) &prop_woodland <0.014 (p23)	rate_local4nondance >0.451 (p61)	31.42	0.79	15.72	0.13	3.8E-05
**108**	med_area_home <17.564 (p53) &den_public_trans >16.704 (p50) &LU_entropy <0.550 (p49)	rate_local4nondance >0.768 (p81)	22.23	0.66	15.71	0.11	6.6E-05
**109**	prop_household_3gen <0.081 (p92) &prop_rural_set <0.041 (p71)	rate_local4nondance >0.271 (p36)	104.87	0.75	15.63	0.03	6E-05
**110**	ave_household_size <2.908 (p63) &LU_entropy <0.550 (p49)	rate_local4nondance >0.721 (p79)	27.78	0.48	15.63	0.15	6.2E-05
**111**	prop_agricultural <0.013 (p61) &POI_pp_mall_mkt <10.307 (p72)	rate_local4nondance >0.184 (p26)	80.85	0.90	15.58	0.07	3.1E-05
**112**	den_public_trans >43.717 (p76)	rate_local4nondance >0.469 (p64)	35.25	0.67	15.45	0.29	2.6E-08
**113**	ave_household_size <2.835 (p49) &prop_transport >0.116 (p50)	rate_local4nondance >0.527 (p69)	36.27	0.56	15.43	0.11	8E-05

**114**	prop_preprim_edu <0.144 (p89) &build_area_pp <89.360 (p62) &prop_transport >0.084 (p38)	rate_local4nondance >0.184 (p26)	79.05	0.90	15.39	0.02	2.4E-06
**115**	prop_over65 <0.212 (p89) &POI_pp_telecom_elec <12.318 (p51)	rate_local4nondance >0.189 (p27)	89.36	0.87	15.37	0.02	5E-05
**116**	prop_agricultural <0.009 (p58) &LU_entropy <0.547 (p47)	rate_local4nondance >0.765 (p81)	28.53	0.41	15.34	0.11	4.3E-05
**117**	prop_preprim_edu <0.143 (p88) &den_road >6.756 (p31) &build_area_pp <87.144 (p62)	rate_local4nondance >0.189 (p27)	80.98	0.89	15.33	0.02	1.6E-05
**118**	med_area_home <15.434 (p45) &POI_pp_sports <79.051 (p90)	rate_local4nondance >0.182 (p26)	80.47	0.90	15.27	0.03	1E-05
**119**	med_area_home <15.547 (p45) &build_area_pp <90.055 (p62)	rate_local4nondance >0.183 (p26)	75.30	0.91	15.23	0.05	5.8E-05
**120**	build_area_pp <93.207 (p63) &prop_rural_set <0.044 (p73) &prop_grassland <0.072 (p68)	rate_local4nondance >0.200 (p27)	67.56	0.91	15.16	0.07	2.2E-05
**121**	prop_rural_set <0.008 (p56)	rate_local4nondance >0.316 (p40)	84.47	0.70	15.11	0.13	1E-05
**122**	ave_household_size <2.908 (p63) &prop_private_resid >0.086 (p63)	rate_local4nondance >0.686 (p78)	25.02	0.57	15.10	0.18	2.9E-05
**123**	med_area_home <17.544 (p53) &prop_woodland <0.064 (p36)	rate_local4nondance >0.484 (p65)	32.34	0.67	15.08	0.15	5.5E-05
**124**	med_area_home <17.981 (p55) &prop_agricultural <0.009 (p59) &LU_entropy <0.528 (p40)	rate_local4nondance >0.503 (p66)	26.45	0.80	15.07	0.10	7.8E-05
**125**	prop_preprim_edu <0.144 (p89) &build_area_pp <89.360 (p62) &prop_shrubland <0.122 (p60)	rate_local4nondance >0.184 (p26)	72.52	0.91	15.02	0.03	1.6E-05
**126**	prop_agricultural <0.000 (p40)	rate_local4nondance >0.684 (p78)	34.23	0.40	15.00	0.18	5.7E-07
**127**	ave_household_size <2.977 (p63) &prop_private_resid >0.067 (p56) &LU_entropy <0.546 (p47)	rate_local4nondance >0.668 (p77)	22.46	0.70	14.96	0.19	5.7E-05
**128**	med_area_home <16.784 (p50) &prop_private_resid >0.105 (p66)	rate_local4nondance >0.710 (p79)	22.43	0.64	14.92	0.24	1.7E-05
**129**	den_road >7.464 (p36) &build_area_pp <90.055 (p62)	rate_local4nondance >0.183 (p26)	82.29	0.88	14.83	0.07	2.2E-06
**130**	prop_preprim_edu <0.152 (p91) &med_area_home <20.005 (p67) &build_area_pp <90.055 (p62)	rate_local4nondance >0.183 (p26)	87.01	0.87	14.83	0.04	7.7E-05
**131**	prop_preprim_edu <0.154 (p91) &build_area_pp <68.364 (p52)	rate_local4nondance >0.171 (p26)	92.29	0.87	14.79	0.02	1.5E-05
**132**	prop_grassland <0.097 (p73) &POI_pp_mall_mkt <10.119 (p71)	rate_local4nondance >0.172 (p26)	92.45	0.87	14.74	0.07	2.6E-05
**133**	med_area_home <15.828 (p46) &prop_rural_set <0.033 (p70) &POI_pp_mall_mkt <11.754 (p77)	rate_local4nondance >0.195 (p27)	61.25	0.94	14.71	0.03	5.9E-05
**134**	prop_woodland <0.064 (p36)	rate_local4nondance >0.699 (p78)	31.86	0.41	14.59	0.19	2.1E-07
**135**	med_area_home <15.828 (p46) &prop_agricultural <0.014 (p61) &POI_pp_transport <41.883 (p64)	rate_local4nondance >0.172 (p26)	59.66	0.97	14.57	0.04	5.5E-05
**136**	den_bldg >0.353 (p79)	rate_local4nondance >0.705 (p79)	24.98	0.52	14.55	0.30	2.2E-09
**137**	build_area_pp <89.360 (p62) &prop_transport >0.084 (p38)	rate_local4nondance >0.184 (p26)	80.37	0.88	14.51	0.05	4.3E-05

**138**	prop_preprim_edu <0.141 (p86) &med_income <20294.359 (p73)	rate_local4nondance >0.453 (p62)	62.86	0.51	14.48	0.06	7.1E-05
**139**	med_area_home <15.828 (p46) &LU_entropy <0.632 (p70) &POI_pp_mall_mkt <11.754 (p77)	rate_local4nondance >0.301 (p37)	44.53	0.88	14.38	0.04	7.2E-05
**140**	prop_open_recreation >0.030 (p48) &LU_entropy <0.550 (p49)	rate_local4nondance >0.715 (p79)	24.66	0.51	14.33	0.18	3.6E-05
**141**	prop_private_resid >0.067 (p56) &LU_entropy <0.544 (p46)	rate_local4nondance >0.692 (p78)	25.06	0.52	14.26	0.17	4.7E-05
**142**	build_area_pp <90.055 (p62) &prop_shrubland <0.118 (p60)	rate_local4nondance >0.183 (p26)	74.06	0.89	13.94	0.08	7.7E-05
**143**	prop_shrubland <0.138 (p65) &POI_pp_transport <58.177 (p76)	rate_local4nondance >0.233 (p31)	82.40	0.81	13.86	0.06	3.9E-05
**144**	med_area_home <16.784 (p50)	rate_local4nondance >0.402 (p55)	62.71	0.59	13.79	0.13	7.1E-05
**145**	prop_preprim_edu <0.154 (p91) &med_income <22780.889 (p78) &prop_open_recreation >0.029 (p47)	rate_local4nondance >0.172 (p26)	74.31	0.90	13.78	0.04	5.6E-05
**146**	prop_industrial <0.000 (p39) &prop_woodland <0.082 (p41)	rate_local4nondance >0.883 (p85)	19.57	0.50	13.70	0.22	2.2E-05
**147**	prop_preprim_edu <0.143 (p88) &den_bldg >0.176 (p50) &build_area_pp <87.144 (p62)	rate_local4nondance >0.189 (p27)	59.91	0.93	13.66	0.02	5.9E-05
**148**	prop_rural_set <0.053 (p75) &POI_pp_sports <14.628 (p46)	rate_local4nondance >0.263 (p35)	60.44	0.83	13.47	0.07	8.2E-05
**149**	prop_preprim_edu <0.140 (p86) &prop_shrubland <0.157 (p71)	rate_local4nondance >0.201 (p27)	108.19	0.81	13.34	0.04	3.7E-05
**150**	med_area_home <17.981 (p55) &prop_gov_insti_faci >0.025 (p36) &LU_entropy <0.528 (p40)	rate_local4nondance >0.501 (p66)	21.89	0.87	13.32	0.17	2.1E-05
**151**	prop_private_resid >0.102 (p66)	rate_local4nondance >0.709 (p79)	28.91	0.40	13.28	0.18	2.2E-07
**152**	den_bldg >0.180 (p52) &build_area_pp <89.773 (p62) &prop_rural_set <0.078 (p82)	rate_local4nondance >0.202 (p27)	55.05	0.93	13.22	0.03	3.6E-05
**153**	prop_preprim_edu <0.154 (p91) &build_area_pp <104.259 (p69) &prop_open_recreation >0.029 (p47)	rate_local4nondance >0.172 (p26)	74.73	0.89	13.17	0.03	3.8E-06
**154**	ave_household_size <2.670 (p29) &den_population >26514.578 (p60)	rate_local4nondance >0.528 (p69)	24.23	0.70	13.14	0.21	7.1E-05
**155**	ave_household_size <2.776 (p37) &prop_industrial <0.000 (p36)	rate_local4nondance >0.883 (p85)	18.56	0.49	12.96	0.20	7.5E-05
**156**	build_area_pp <60.952 (p50)	rate_local4nondance >0.177 (p26)	86.02	0.86	12.89	0.13	1.8E-06
**157**	den_bldg >0.195 (p54) &build_area_pp <90.055 (p62)	rate_local4nondance >0.183 (p26)	57.77	0.93	12.71	0.07	2.5E-05
**158**	prop_shrubland <0.000 (p18)	rate_local4nondance >0.966 (p88)	17.77	0.46	12.58	0.32	6.6E-13
**159**	den_population >53156.370 (p79) &build_area_pp <104.293 (p69) &prop_rural_set <0.046 (p73)	rate_local4nondance >0.238 (p31)	42.43	0.95	12.30	0.01	5.4E-05
**160**	den_public_trans >23.523 (p57) &build_area_pp <89.360 (p62)	rate_local4nondance >0.184 (p26)	60.66	0.91	12.29	0.06	7.9E-05
**161**	den_public_trans >25.295 (p58) &build_area_pp <85.683 (p60) &prop_shrubland <0.186 (p79)	rate_local4nondance >0.184 (p26)	55.09	0.93	12.09	0.02	6.4E-05
**162**	den_population >53156.370 (p79) &build_area_pp <104.293 (p69)	rate_local4nondance >0.238 (p31)	43.64	0.93	12.07	0.00	7.1E-06

**163**	med_income <14611.112 (p25) &LU_entropy <0.543 (p46)	rate_local4nondance >0.604 (p74)	18.09	0.79	12.01	0.37	8.7E-06
**164**	LU_entropy <0.549 (p48)	rate_local4nondance >0.673 (p77)	35.65	0.35	11.85	0.12	2.9E-05
**165**	LU_entropy <0.632 (p70) &POI_pp_telecom_elec <8.323 (p41) &POI_pp_mall_mkt <11.754 (p77)	rate_local4nondance >0.208 (p29)	46.52	0.94	11.72	0.02	7.8E-05
**166**	build_area_pp <99.648 (p67) &prop_open_recreation >0.027 (p46)	rate_local4nondance >0.172 (p26)	74.96	0.86	11.34	0.06	3.7E-05
**167**	ave_household_size <2.593 (p17)	rate_local4nondance >0.540 (p71)	26.06	0.54	11.30	0.23	1.6E-06
**168**	den_road >18.893 (p78) &prop_private_resid >0.066 (p56)	rate_local4nondance >0.298 (p37)	34.91	0.89	11.27	0.08	5E-05
**169**	prop_industrial <0.000 (p36)	rate_local4nondance >0.883 (p85)	22.79	0.29	11.10	0.14	4.5E-06
**170**	prop_publicResid >0.060 (p74)	rate_local4nondance >0.259 (p35)	46.79	0.84	10.49	0.19	5.3E-05
**171**	den_road =16.248-26.910 (p71-92)	rate_local4nondance >0.711 (p79)	19.42	0.43	9.62	0.21	3.7E-06
**172**	med_income <14182.663 (p21)	rate_local4nondance >0.455 (p62)	26.82	0.60	9.40	0.21	7E-05
**173**	build_area_pp <37.211 (p27) &prop_grassland <0.020 (p46) &LU_entropy <0.523 (p40)	rate_local4nondance >0.737 (p79)	11.65	0.97	9.22	0.24	6.3E-05
**174**	build_area_pp <35.213 (p22) &prop_shrubland <0.172 (p76) &LU_entropy <0.550 (p49)	rate_local4nondance >0.780 (p82)	11.24	0.86	8.82	0.22	3.7E-05
**175**	prop_business >0.085 (p90)	rate_local4nondance >0.663 (p77)	14.01	0.63	8.75	0.39	1.6E-07
**176**	build_area_pp <37.211 (p27) &LU_entropy <0.523 (p40)	rate_local4nondance >0.737 (p79)	11.69	0.73	8.44	0.39	6.2E-05
**177**	prop_preprim_edu <0.154 (p91)	rate_local4nondance >0.181 (p26)	149.43	0.77	8.23	0.04	5.3E-07
**178**	den_road >26.910 (p92)	rate_local4nondance >0.711 (p79)	11.57	0.67	7.87	0.46	4.1E-06
**179**	build_area_pp <39.920 (p30) &POI_pp_transport >34.785 (p57)	rate_local4nondance >1.045 (p89)	4.14	0.56	3.34	0.39	3.5E-05
**180**	build_area_pp <39.359 (p29) &POI_pp_sports >28.053 (p65)	rate_local4nondance >1.036 (p89)	3.45	0.62	2.84	0.45	1E-05
**181**	prop_preprim_edu <0.146 (p89) &ave_household_size >2.549 (p17) &POI_pp_mall_mkt <8.343 (p65)	rate_local4nondance =0.014-0.963 (p18-88)	98.12	0.91	23.43	0.03	1.1E-05
**182**	ave_household_size >2.549 (p17) &POI_pp_telecom_elec <17.433 (p62) &POI_pp_mall_mkt <8.343 (p65)	rate_local4nondance =0.014-0.963 (p18-88)	96.71	0.91	23.43	0.02	1.2E-05
**183**	ave_household_size >2.549 (p17) &POI_pp_telecom_elec <17.433 (p62)	rate_local4nondance =0.014-0.963 (p18-88)	100.00	0.89	22.52	0.05	8.5E-07
**184**	prop_preprim_edu <0.136 (p85) &POI_pp_mall_mkt <8.343 (p65)	rate_local4nondance =0.014-0.963 (p18-88)	103.84	0.88	22.28	0.04	2.4E-05
**185**	den_road <17.868 (p75) &POI_pp_telecom_elec <17.433 (p62) &POI_pp_mall_mkt <8.343 (p65)	rate_local4nondance =0.014-0.963 (p18-88)	88.33	0.92	22.19	0.02	7.4E-05
**186**	ave_household_size >2.657 (p29) &POI_pp_sports <28.806 (p66)	rate_local4nondance =0.022-0.951 (p18-88)	92.04	0.90	22.14	0.07	2.4E-05
**187**	den_road <23.151 (p87) &POI_pp_telecom_elec <13.898 (p56)	rate_local4nondance =0.048-0.883 (p18-85)	91.10	0.88	22.11	0.05	7.8E-05

**188**	LU_entropy >0.549 (p48) &POI_pp_telecom_elec <18.430 (p65)	rate_local4nondance =0.016-0.871 (p18-85)	80.71	0.91	21.73	0.11	6.8E-07
**189**	LU_entropy >0.539 (p44) &POI_pp_sports <31.203 (p68)	rate_local4nondance =0.017-0.650 (p18-76)	76.60	0.80	21.65	0.10	1.1E-05
**190**	ave_household_size >2.527 (p17) &POI_pp_telecom_elec <20.788 (p69) &POI_pp_transport <33.527 (p56)	rate_local4nondance =0.060-1.051 (p18-89)	94.64	0.91	21.52	0.01	4.5E-05
**191**	ave_household_size >2.663 (p29) &POI_pp_mall_mkt <7.867 (p63)	rate_local4nondance =0.014-0.899 (p18-86)	87.95	0.89	21.27	0.06	4.2E-05
**192**	ave_household_size >2.527 (p17) &POI_pp_transport <33.527 (p56)	rate_local4nondance =0.060-1.051 (p18-89)	95.34	0.91	21.03	0.03	1.5E-05
**193**	ave_household_size >2.616 (p29) &POI_pp_edu <17.631 (p70)	rate_local4nondance =0.014-0.963 (p18-88)	100.36	0.87	20.93	0.06	1.9E-05
**194**	prop_preprim_edu <0.154 (p91) &POI_pp_edu <15.368 (p68)	rate_local4nondance =0.006-0.926 (p18-86)	108.44	0.84	20.87	0.03	4.6E-05
**195**	POI_pp_sports <28.160 (p66)	rate_local4nondance =0.015-0.998 (p18-88)	117.21	0.85	20.85	0.15	1E-11
**196**	LU_entropy >0.545 (p47) &POI_pp_transport <44.490 (p67)	rate_local4nondance =0.019-0.778 (p18-82)	79.21	0.85	20.79	0.09	3E-05
**197**	LU_entropy >0.526 (p40) &POI_pp_mall_mkt <8.791 (p66)	rate_local4nondance =0.016-0.659 (p18-77)	79.54	0.78	20.62	0.08	5.9E-05
**198**	POI_pp_transport <44.490 (p67)	rate_local4nondance =0.019-0.992 (p18-88)	117.66	0.84	20.31	0.15	1.8E-11
**199**	prop_private_resid <0.192 (p81) &POI_pp_telecom_elec <15.780 (p59)	rate_local4nondance =0.044-0.724 (p18-79)	81.10	0.81	19.99	0.06	1.8E-05
**200**	prop_private_resid <0.163 (p78) &POI_pp_sports <28.912 (p66)	rate_local4nondance =0.057-0.775 (p18-82)	85.96	0.81	19.90	0.06	2.7E-05
**201**	prop_preprim_edu <0.153 (p91) &LU_entropy >0.545 (p47)	rate_local4nondance =0.003-0.874 (p18-85)	87.60	0.86	19.77	0.06	9E-08
**202**	prop_private_resid <0.162 (p78) &POI_pp_telecom_elec <20.788 (p69) &POI_pp_transport <33.527 (p56)	rate_local4nondance =0.060-1.051 (p18-89)	83.56	0.92	19.70	0.01	2E-05
**203**	LU_entropy >0.538 (p44) &POI_pp_edu <15.368 (p68)	rate_local4nondance =0.006-0.926 (p18-86)	83.14	0.89	19.65	0.08	4E-05
**204**	POI_pp_telecom_elec <19.858 (p68)	rate_local4nondance =0.015-0.998 (p18-88)	121.45	0.83	19.63	0.13	2.7E-10
**205**	prop_private_resid <0.162 (p78) &POI_pp_transport <33.527 (p56)	rate_local4nondance =0.060-1.051 (p18-89)	84.37	0.91	19.21	0.04	7.4E-05
**206**	POI_pp_mall_mkt <8.922 (p66)	rate_local4nondance =0.015-0.998 (p18-88)	118.47	0.83	19.14	0.13	4E-11
**207**	prop_private_resid <0.192 (p81) &POI_pp_edu <12.154 (p55)	rate_local4nondance =0.044-0.724 (p18-79)	79.80	0.80	18.89	0.05	7.6E-05
**208**	den_bldg <0.254 (p65) &POI_pp_sports <28.912 (p66)	rate_local4nondance =0.057-0.775 (p18-82)	75.97	0.82	18.79	0.07	4.1E-05
**209**	POI_pp_edu <17.631 (p70)	rate_local4nondance =0.015-0.998 (p18-88)	123.19	0.82	18.52	0.12	4E-11
**210**	prop_over65 <0.214 (p90) &LU_entropy >0.551 (p49)	rate_local4nondance =0.015-0.933 (p18-86)	82.41	0.87	17.94	0.06	3.1E-05
**211**	den_road <13.077 (p60) &POI_pp_sports <28.912 (p66)	rate_local4nondance =0.057-0.775 (p18-82)	67.80	0.83	17.05	0.08	1.6E-05
**212**	den_road <13.077 (p60) &POI_pp_transport <43.655 (p65)	rate_local4nondance =0.057-0.775 (p18-82)	67.29	0.83	16.78	0.08	4.6E-05
**213**	LU_entropy >0.549 (p48)	rate_local4nondance =0.058-0.673 (p18-77)	81.07	0.72	15.78	0.14	4.6E-07
**214**	prop_preprim_edu <0.146 (p89) &ave_household_size >2.549 (p17)	rate_local4nondance =0.014-0.963 (p18-88)	120.96	0.78	14.31	0.05	5.9E-05
**215**	den_public_trans >16.704 (p50) &LU_entropy >0.550 (p49)	rate_local4nondance =0.289-0.768 (p37-81)	35.37	0.61	10.80	0.11	2.7E-05

**216**	prop_shrubland =0.000-0.071 (p18-50)	rate_local4nondance =0.169-0.966 (p26-88)	50.94	0.74	9.41	0.14	7.6E-05
**217**	prop_household_3gen <0.062 (p86)	rate_local4nondance =0.015-0.998 (p18-88)	137.28	0.75	8.91	0.05	3.4E-05
**218**	den_public_trans >20.063 (p54) &prop_shrubland =0.041-0.165 (p42-74)	rate_local4nondance =0.170-0.814 (p26-83)	20.01	0.81	6.17	0.18	4.2E-05
**219**	den_road <15.914 (p70) &prop_publicResid <0.049 (p71)	rate_local4nondance <0.287 (p37)	59.77	0.57	20.58	0.10	5.5E-05
**220**	den_bldg <0.262 (p67) &prop_publicResid <0.045 (p71)	rate_local4nondance <0.289 (p37)	57.88	0.58	20.38	0.10	7.1E-05
**221**	prop_publicResid <0.059 (p73) &prop_transport <0.184 (p64)	rate_local4nondance <0.308 (p38)	62.49	0.60	19.39	0.08	7.9E-05
**222**	prop_publicResid <0.048 (p71) &prop_business <0.011 (p70)	rate_local4nondance <0.317 (p40)	62.51	0.59	19.35	0.11	4.8E-05
**223**	prop_transport <0.145 (p55) &POI_pp_sports >25.649 (p63)	rate_local4nondance <0.013 (p18)	28.20	0.54	19.01	0.16	1.1E-05
**224**	den_population <9985.274 (p41) &POI_pp_sports >25.649 (p63)	rate_local4nondance <0.013 (p18)	27.65	0.56	18.95	0.18	9.7E-06
**225**	den_population <7419.977 (p38)	rate_local4nondance <0.168 (p26)	39.48	0.48	18.88	0.23	7.5E-11
**226**	prop_private_resid <0.123 (p70) &prop_publicResid <0.045 (p71)	rate_local4nondance <0.289 (p37)	56.36	0.57	18.87	0.11	3.7E-05
**227**	den_bldg <0.243 (p64) &POI_pp_transport >33.527 (p56)	rate_local4nondance <0.014 (p18)	29.14	0.49	18.54	0.14	2.7E-05
**228**	den_population <10950.266 (p43) &POI_pp_transport >40.350 (p62)	rate_local4nondance <0.006 (p18)	26.94	0.54	18.09	0.16	5.9E-05
**229**	den_road <13.077 (p60) &POI_pp_sports >28.912 (p66)	rate_local4nondance <0.057 (p18)	26.42	0.57	17.95	0.17	1.5E-05
**230**	prop_publicResid <0.048 (p71) &prop_grassland >0.033 (p57)	rate_local4nondance <0.263 (p35)	46.43	0.57	17.91	0.08	1.9E-05
**231**	prop_transport <0.132 (p52) &POI_pp_transport >29.897 (p52)	rate_local4nondance <0.106 (p20)	30.09	0.49	17.87	0.15	6.7E-05
**232**	den_bldg <0.254 (p65) &POI_pp_sports >28.912 (p66)	rate_local4nondance <0.057 (p18)	26.92	0.54	17.83	0.14	7E-05
**233**	den_road <13.077 (p60) &POI_pp_transport >43.655 (p65)	rate_local4nondance <0.057 (p18)	26.22	0.57	17.79	0.17	4.6E-05
**234**	prop_transport <0.140 (p54) &POI_pp_telecom_elec >12.979 (p54)	rate_local4nondance <0.073 (p19)	29.76	0.46	17.52	0.13	4.9E-05
**235**	POI_pp_sports >28.160 (p66)	rate_local4nondance <0.015 (p18)	31.09	0.41	17.52	0.23	1E-11
**236**	den_road <16.248 (p71)	rate_local4nondance <0.711 (p79)	136.45	0.90	17.39	0.11	5.1E-12
**237**	den_road <17.994 (p75) &prop_business <0.057 (p86)	rate_local4nondance <0.697 (p78)	139.38	0.89	17.38	0.01	1.7E-06
**238**	den_population <47091.966 (p74) &prop_business <0.079 (p89)	rate_local4nondance <0.474 (p64)	108.32	0.75	17.29	0.03	6.3E-05
**239**	den_road <13.077 (p60) &POI_pp_edu >11.980 (p55)	rate_local4nondance <0.091 (p19)	27.82	0.51	17.26	0.17	8.6E-06
**240**	med_area_home >14.781 (p37) &prop_business <0.056 (p86)	rate_local4nondance <0.469 (p64)	90.52	0.78	17.17	0.06	5.6E-05
**241**	den_bldg <0.154 (p46) &POI_pp_telecom_elec >12.150 (p51)	rate_local4nondance <0.101 (p20)	28.73	0.51	17.15	0.17	2.5E-05
**242**	prop_transport <0.143 (p55)	rate_local4nondance <0.419 (p58)	83.18	0.71	17.13	0.15	9.2E-08
**243**	prop_transport <0.134 (p52) &POI_pp_edu >11.888 (p55)	rate_local4nondance <0.090 (p19)	27.48	0.50	16.93	0.16	3.3E-05
**244**	prop_shrubland >0.058 (p48) &POI_pp_sports >25.506 (p63)	rate_local4nondance <0.010 (p18)	25.08	0.55	16.92	0.16	6.1E-05
**245**	den_bldg <0.193 (p53)	rate_local4nondance <0.317 (p40)	64.47	0.56	16.92	0.15	6.1E-06
**246**	prop_transport <0.145 (p55) &POI_pp_mall_mkt >7.372 (p60)	rate_local4nondance <0.006 (p18)	25.34	0.53	16.84	0.18	1.2E-05

**247**	POI_pp_transport >44.490 (p67)	rate_local4nondance <0.019 (p18)	29.99	0.41	16.83	0.23	3.9E-11
**248**	den_road <13.153 (p60) &POI_pp_mall_mkt >7.878 (p64)	rate_local4nondance <0.009 (p18)	24.54	0.56	16.77	0.21	5.3E-06
**249**	den_population <9173.983 (p40) &POI_pp_edu >15.204 (p67)	rate_local4nondance <0.026 (p18)	24.11	0.58	16.68	0.20	1.6E-05
**250**	den_public_trans <24.072 (p58)	rate_local4nondance <0.469 (p64)	93.72	0.76	16.63	0.14	7.8E-08
**251**	den_bldg <0.165 (p48) &POI_pp_edu >11.833 (p55)	rate_local4nondance <0.106 (p20)	25.79	0.55	16.48	0.21	8.9E-06
**252**	den_population <10000.913 (p42) &POI_pp_mall_mkt >8.364 (p65)	rate_local4nondance <0.003 (p18)	23.44	0.58	16.25	0.21	6.4E-05
**253**	den_bldg <0.154 (p46) &POI_pp_mall_mkt >7.030 (p60)	rate_local4nondance <0.015 (p18)	22.89	0.57	15.72	0.23	7.3E-06
**254**	prop_private_resid <0.117 (p70) &POI_pp_mall_mkt >5.409 (p51)	rate_local4nondance <0.194 (p27)	34.68	0.49	15.30	0.12	6.9E-05
**255**	prop_shrubland >0.047 (p43) &POI_pp_mall_mkt >6.422 (p56)	rate_local4nondance <0.084 (p19)	24.87	0.49	15.20	0.16	2.3E-05
**256**	den_road <20.469 (p81) &den_public_trans <24.072 (p58)	rate_local4nondance <0.708 (p79)	106.89	0.91	14.96	0.03	5.4E-05
**257**	POI_pp_edu >17.631 (p70)	rate_local4nondance <0.015 (p18)	26.36	0.41	14.94	0.23	1E-09
**258**	prop_agricultural >0.027 (p72)	rate_local4nondance <0.296 (p37)	39.10	0.63	14.82	0.24	1.9E-07
**259**	prop_shrubland >0.058 (p48) &POI_pp_edu >13.918 (p63)	rate_local4nondance <0.098 (p19)	23.43	0.53	14.65	0.16	3.2E-05
**260**	POI_pp_mall_mkt >8.922 (p66)	rate_local4nondance <0.015 (p18)	27.13	0.38	14.31	0.20	7.4E-08
**261**	prop_woodland >0.064 (p36)	rate_local4nondance <0.699 (p78)	120.23	0.88	14.31	0.11	2.1E-07
**262**	den_public_trans <14.796 (p47) &POI_pp_mall_mkt >7.062 (p60)	rate_local4nondance <0.000 (p0)	22.43	0.47	13.98	0.14	7.9E-05
**263**	prop_publicResid <0.052 (p72) &prop_shrubland =0.108-0.217 (p57-82)	rate_local4nondance <0.263 (p35)	27.66	0.70	13.96	0.16	2.3E-05
**264**	med_area_home >16.784 (p50)	rate_local4nondance <0.402 (p55)	71.80	0.67	13.70	0.13	7.1E-05
**265**	prop_grassland >0.038 (p58)	rate_local4nondance <0.364 (p49)	60.21	0.63	13.65	0.14	2.1E-05
**266**	prop_private_resid <0.102 (p66)	rate_local4nondance <0.709 (p79)	124.65	0.88	13.36	0.09	2.2E-07
**267**	build_area_pp >60.952 (p50)	rate_local4nondance <0.177 (p26)	43.74	0.38	12.74	0.11	1.8E-06
**268**	prop_gov_insti_faci <0.023 (p33)	rate_local4nondance <0.016 (p18)	25.53	0.35	12.54	0.17	2.5E-06
**269**	prop_open_recreation <0.008 (p27)	rate_local4nondance <0.277 (p37)	33.47	0.58	12.41	0.21	6E-05
**270**	prop_rural_set >0.040 (p71)	rate_local4nondance <0.316 (p40)	38.48	0.62	12.32	0.20	2E-05
**271**	prop_shrubland >0.071 (p50)	rate_local4nondance <0.169 (p26)	39.12	0.37	11.44	0.11	6.6E-05
**272**	ave_household_size >2.593 (p17)	rate_local4nondance <0.540 (p71)	126.28	0.76	11.40	0.07	1.6E-06
**273**	den_bldg <0.113 (p37) &prop_woodland <0.153 (p58)	rate_local4nondance <0.399 (p55)	25.15	0.94	10.66	0.24	2.3E-05
**274**	prop_publicResid <0.060 (p74)	rate_local4nondance <0.259 (p35)	66.26	0.42	10.61	0.07	5.3E-05
**275**	POI_pp_telecom_elec >50.291 (p90)	rate_local4nondance <0.015 (p18)	13.60	0.60	9.55	0.42	2.5E-08
**276**	prop_household_3gen >0.062 (p86)	rate_local4nondance <0.015 (p18)	14.93	0.49	9.51	0.31	2.6E-07
**277**	prop_preprim_edu >0.154 (p91)	rate_local4nondance <0.003 (p18)	12.86	0.65	9.36	0.47	1.4E-09
**278**	POI_pp_telecom_elec =19.858-50.291 (p68-90)	rate_local4nondance <0.015 (p18)	17.52	0.38	9.28	0.20	4.8E-05

**279**	prop_business <0.085 (p90)	rate_local4nondance <0.663 (p77)	155.00	0.81	8.64	0.05	1.6E-07
**280**	med_income <26002.239 (p82) &den_bldg <0.118 (p38) &prop_woodland <0.150 (p57)	rate_local4nondance <0.339 (p44)	18.43	0.80	7.99	0.04	7.6E-05
**281**	prop_over65 >0.232 (p94)	rate_local4nondance <0.177 (p26)	10.00	0.77	6.64	0.51	1.3E-05
**282**	gender_ratio >109.069 (p94)	rate_local4nondance <0.087 (p19)	9.00	0.68	6.47	0.49	2.2E-06

(f) Rules for wave-1 local case rate; POI density was used

No.	Antecedent	Consequent	Supp	Conf	Lev	Imp	*P*
**1**	build_area_pp >66.740 (p52)	rate_local12 >0.113 (p81)	32.36	0.30	11.69	0.11	2.006E-05
**2**	POI_den_sports =235.262-1634.870 (p58-91)	rate_local12 >0.039 (p60)	39.15	0.56	11.23	0.16	2.365E-05
**3**	prop_preprim_edu <0.080 (p19)	rate_local12 >0.114 (p81)	17.89	0.44	10.13	0.25	7.552E-06
**4**	LU_entropy <0.543 (p46)	rate_local12 >0.103 (p79)	30.07	0.31	9.55	0.10	7.803E-05
**5**	prop_private_resid >0.162 (p78)	rate_local12 >0.062 (p71)	24.06	0.50	9.24	0.19	6.131E-05
**6**	prop_household_3gen <0.023 (p19)	rate_local12 >0.102 (p78)	18.22	0.40	8.54	0.19	2.745E-05
**7**	med_area_home >15.950 (p47)	rate_local12 >0.132 (p83)	27.40	0.23	8.53	0.07	5.739E-05
**8**	POI_den_telecom_elec >375.328 (p72)	rate_local12 >0.103 (p79)	19.82	0.34	7.34	0.12	3.887E-05
**9**	med_income >19063.503 (p63)	rate_local12 >0.133 (p84)	19.74	0.25	7.27	0.09	6.98E-05
**10**	prop_transport >0.336 (p88)	rate_local12 >0.091 (p77)	12.50	0.49	6.58	0.26	1.018E-05
**11**	prop_higher_edu >0.382 (p87)	rate_local12 >0.136 (p86)	10.35	0.39	6.23	0.24	3.376E-05
**12**	gender_ratio >106.162 (p92)	rate_local12 >0.108 (p81)	9.79	0.52	6.00	0.32	4.133E-05
**13**	prop_private_resid =0.017-0.058 (p33-55) &prop_business <0.000 (p26)	rate_local12 >0.170 (p89)	3.83	0.68	3.17	0.53	1.705E-08
**14**	gender_ratio =87.315-99.297 (p11-79) &med_area_home <18.056 (p55)	rate_local12 =0.005-0.102 (p45-78)	57.32	0.63	27.28	0.12	2.065E-07
**15**	prop_preprim_edu =0.081-0.132 (p20-83) &med_area_home <17.190 (p52) &POI_den_telecom_elec <632.455 (p82)	rate_local12 =0.005-0.099 (p45-78)	49.00	0.74	27.18	0.11	5.256E-05
**16**	prop_preprim_edu =0.080-0.134 (p19-84) &med_area_home <17.190 (p52) &POI_den_edu <626.668 (p81)	rate_local12 =0.005-0.096 (p45-77)	48.86	0.72	27.15	0.11	6.1E-06
**17**	prop_preprim_edu =0.081-0.132 (p20-83) &med_area_home <17.190 (p52) &POI_den_mall_mkt <344.195 (p80)	rate_local12 =0.006-0.092 (p45-77)	47.56	0.72	26.97	0.11	1.286E-05
**18**	gender_ratio =85.221-98.201 (p10-78) &med_area_home <21.719 (p78) &prop_gov_insti_faci =0.017-0.148 (p31-92)	rate_local12 =0.005-0.105 (p45-80)	55.03	0.66	26.65	0.07	4.552E-05
**19**	prop_preprim_edu =0.081-0.132 (p20-83) &med_area_home <17.190 (p52) &POI_den_sports <854.408 (p78)	rate_local12 =0.005-0.091 (p45-77)	46.56	0.73	26.65	0.11	1.966E-05
**20**	den_road >5.958 (p28) &build_area_pp <91.523 (p62) &POI_den_mall_mkt <420.619 (p84)	rate_local12 =0.006-0.104 (p45-80)	53.21	0.67	26.57	0.11	1.911E-05
**21**	den_road >5.901 (p28) &build_area_pp <89.092 (p62) &POI_den_sports <1142.618 (p83)	rate_local12 =0.005-0.102 (p45-79)	52.17	0.67	26.37	0.11	3.762E-05
**22**	den_road >5.901 (p28) &build_area_pp <89.092 (p62) &POI_den_edu <593.042 (p80)	rate_local12 =0.005-0.099 (p45-78)	50.21	0.68	26.13	0.13	1.04E-05
**23**	build_area_pp <90.579 (p62) &prop_transport >0.073 (p36) &POI_den_mall_mkt <425.102 (p84)	rate_local12 =0.005-0.105 (p45-80)	50.61	0.70	26.06	0.12	1.69E-05
**24**	prop_over65 =0.120-0.208 (p19-86) &med_area_home <19.142 (p61) &build_area_pp <97.100 (p65)	rate_local12 =0.004-0.118 (p45-82)	53.31	0.70	25.97	0.09	7.57E-05

**25**	den_road >5.957 (p28) &build_area_pp <92.118 (p63) &POI_den_transport <1507.864 (p80)	rate_local12 =0.006-0.100 (p45-78)	50.42	0.68	25.95	0.13	7.5E-06
**26**	gender_ratio =86.676-98.772 (p11-78) &den_road >6.094 (p29) &build_area_pp <97.142 (p65)	rate_local12 =0.006-0.106 (p45-80)	55.24	0.64	25.94	0.08	4.19E-05
**27**	gender_ratio <96.984 (p75) &med_area_home <19.270 (p61) &POI_den_telecom_elec <596.386 (p80)	rate_local12 =0.005-0.104 (p45-79)	54.94	0.63	25.80	0.07	1.107E-05
**28**	med_area_home <17.472 (p52) &prop_business <0.064 (p87)	rate_local12 =0.004-0.115 (p45-81)	60.46	0.62	25.73	0.07	3.7E-05
**29**	den_road >5.750 (p27) &build_area_pp <88.011 (p62) &POI_den_telecom_elec <592.792 (p80)	rate_local12 =0.005-0.102 (p45-78)	50.45	0.68	25.69	0.12	2.301E-06
**30**	build_area_pp <92.122 (p63) &prop_transport >0.075 (p36) &POI_den_edu <635.332 (p81)	rate_local12 =0.005-0.097 (p45-77)	47.80	0.70	25.62	0.14	1.355E-06
**31**	med_area_home <18.611 (p56) &prop_gov_insti_faci =0.013-0.147 (p29-91) &POI_den_edu <1024.661 (p91)	rate_local12 =0.005-0.149 (p45-87)	61.61	0.70	25.61	0.05	6.69E-05
**32**	med_area_home <15.485 (p45) &POI_den_edu <628.086 (p81)	rate_local12 =0.005-0.096 (p45-77)	49.00	0.67	25.57	0.12	3.239E-06
**33**	den_population >14298.147 (p45) &build_area_pp <61.172 (p50) &POI_den_mall_mkt <420.564 (p84)	rate_local12 =0.005-0.113 (p45-81)	47.19	0.77	25.50	0.13	7.112E-06
**34**	med_area_home <17.787 (p55) &build_area_pp <61.308 (p50) &POI_den_mall_mkt <420.564 (p84)	rate_local12 =0.004-0.105 (p45-80)	47.40	0.73	25.47	0.11	4.477E-05
**35**	med_area_home <15.549 (p45) &POI_den_mall_mkt <301.051 (p78)	rate_local12 =0.005-0.101 (p45-78)	48.58	0.69	25.45	0.13	1.529E-05
**36**	gender_ratio =87.133-95.838 (p11-69) &prop_gov_insti_faci =0.028-0.140 (p39-91) &POI_den_edu <709.299 (p82)	rate_local12 =0.005-0.102 (p45-78)	46.79	0.73	25.40	0.07	5.552E-05
**37**	prop_preprim_edu =0.080-0.134 (p19-84) &med_area_home <17.190 (p52)	rate_local12 =0.005-0.096 (p45-77)	53.10	0.62	25.39	0.11	9.426E-06
**38**	prop_preprim_edu =0.081-0.137 (p20-85) &build_area_pp <89.843 (p62) &POI_den_transport <1475.696 (p79)	rate_local12 =0.005-0.095 (p45-77)	50.91	0.64	25.39	0.09	2.008E-05
**39**	med_area_home <15.520 (p45) &POI_den_sports <785.405 (p78)	rate_local12 =0.005-0.102 (p45-79)	48.41	0.70	25.39	0.13	7.961E-06
**40**	gender_ratio =86.919-97.765 (p11-77) &prop_preprim_edu =0.079-0.131 (p18-83) &prop_gov_insti_faci =0.027-0.148 (p38-92)	rate_local12 =0.006-0.100 (p45-78)	47.13	0.71	25.38	0.09	7.554E-05
**41**	gender_ratio <97.028 (p75) &med_area_home <19.771 (p65) &POI_den_mall_mkt <377.755 (p81)	rate_local12 =0.005-0.096 (p45-77)	54.91	0.60	25.37	0.07	6.913E-05
**42**	med_area_home <22.436 (p80) &den_population >14968.054 (p46) &POI_den_mall_mkt <344.016 (p80)	rate_local12 =0.005-0.106 (p45-80)	48.93	0.70	25.36	0.06	6.944E-05
**43**	den_population >14968.054 (p46) &build_area_pp <63.670 (p51) &POI_den_telecom_elec <776.385 (p86)	rate_local12 =0.005-0.115 (p45-81)	48.35	0.75	25.28	0.11	4.685E-05
**44**	build_area_pp <90.225 (p62) &prop_transport >0.077 (p36) &POI_den_transport <1565.639 (p81)	rate_local12 =0.005-0.096 (p45-77)	46.74	0.70	25.26	0.14	2.331E-06

**45**	gender_ratio =86.526-97.713 (p10-77) &prop_preprim_edu =0.083-0.132 (p23-83) &build_area_pp <120.964 (p76)	rate_local12 =0.005-0.108 (p45-81)	53.67	0.65	25.21	0.07	6.911E-05
**46**	med_area_home <20.365 (p67) &den_population >14308.702 (p45) &prop_business <0.064 (p87)	rate_local12 =0.004-0.115 (p45-81)	54.31	0.67	25.20	0.08	2.889E-05
**47**	build_area_pp <61.021 (p50) &POI_den_transport =27.865-2265.299 (p12-90)	rate_local12 =0.005-0.106 (p45-81)	54.09	0.64	25.17	0.09	4.499E-06
**48**	med_area_home <15.485 (p45) &POI_den_telecom_elec <451.899 (p77)	rate_local12 =0.005-0.096 (p45-77)	47.28	0.69	25.17	0.13	6.868E-06
**49**	gender_ratio =86.694-98.115 (p11-78) &build_area_pp <83.408 (p58) &prop_transport >0.078 (p36)	rate_local12 =0.005-0.107 (p45-81)	49.85	0.69	25.14	0.09	3.381E-05
**50**	den_population >14968.054 (p46) &build_area_pp <60.895 (p50) &POI_den_sports <1154.560 (p84)	rate_local12 =0.005-0.117 (p45-82)	46.60	0.78	25.12	0.13	8.232E-06
**51**	build_area_pp <93.046 (p63) &prop_transport >0.075 (p36) &POI_den_sports <900.782 (p79)	rate_local12 =0.005-0.099 (p45-78)	47.05	0.70	25.11	0.14	1.386E-06
**52**	prop_over65 =0.114-0.194 (p15-80) &gender_ratio =86.802-97.562 (p11-76) &med_area_home <19.908 (p65)	rate_local12 =0.006-0.094 (p45-77)	47.78	0.67	25.11	0.10	8.208E-05
**53**	med_area_home <22.436 (p80) &den_population >14968.054 (p46) &POI_den_telecom_elec <580.164 (p80)	rate_local12 =0.005-0.106 (p45-80)	48.74	0.70	25.07	0.05	2.41E-05
**54**	med_area_home <15.717 (p46) &POI_den_transport <1281.420 (p77)	rate_local12 =0.005-0.095 (p45-77)	47.41	0.68	25.07	0.13	2.463E-06
**55**	gender_ratio =87.078-97.412 (p11-76) &prop_gov_insti_faci =0.025-0.149 (p36-92)	rate_local12 =0.005-0.104 (p45-79)	53.81	0.63	25.01	0.13	1.903E-07
**56**	prop_preprim_edu =0.081-0.138 (p21-86) &prop_gov_insti_faci =0.025-0.143 (p36-91)	rate_local12 =0.005-0.100 (p45-78)	52.08	0.63	24.94	0.13	3.104E-07
**57**	build_area_pp <58.503 (p48) &POI_den_edu <1049.012 (p91)	rate_local12 =0.005-0.109 (p45-81)	56.46	0.62	24.93	0.06	3.904E-05
**58**	build_area_pp <58.503 (p48) &prop_gov_insti_faci =0.025-0.150 (p36-92) &POI_den_mall_mkt <444.965 (p86)	rate_local12 =0.005-0.099 (p45-78)	43.62	0.76	24.89	0.12	5.007E-05
**59**	build_area_pp <94.661 (p64) &prop_transport >0.055 (p33) &POI_den_telecom_elec <499.056 (p78)	rate_local12 =0.005-0.092 (p45-77)	47.69	0.66	24.85	0.14	4.578E-06
**60**	den_population >14308.702 (p45) &build_area_pp <58.435 (p48) &POI_den_edu <716.583 (p83)	rate_local12 =0.005-0.114 (p45-81)	46.09	0.77	24.85	0.13	1.044E-05
**61**	build_area_pp <61.172 (p50) &prop_gov_insti_faci =0.025-0.138 (p36-90) &POI_den_transport <1795.006 (p84)	rate_local12 =0.005-0.111 (p45-81)	44.79	0.78	24.82	0.11	2.153E-05
**62**	prop_preprim_edu =0.080-0.128 (p19-82) &build_area_pp <69.699 (p52) &POI_den_edu <596.553 (p80)	rate_local12 =0.005-0.101 (p45-78)	47.57	0.69	24.77	0.10	4.259E-05
**63**	prop_preprim_edu =0.079-0.130 (p18-83) &den_road >5.901 (p28) &POI_den_telecom_elec <591.098 (p80)	rate_local12 =0.005-0.093 (p45-77)	50.31	0.62	24.71	0.10	2.342E-06

**64**	prop_over65 =0.124-0.201 (p21-83) &med_area_home <17.338 (p52) &prop_business <0.054 (p86)	rate_local12 =0.005-0.113 (p45-81)	46.41	0.75	24.66	0.10	2.519E-05
**65**	den_bldg >0.080 (p27) &build_area_pp <92.118 (p63) &POI_den_edu <685.349 (p82)	rate_local12 =0.006-0.103 (p45-79)	50.30	0.66	24.62	0.11	6.437E-06
**66**	prop_preprim_edu =0.078-0.138 (p17-85) &build_area_pp <61.966 (p50) &POI_den_telecom_elec <825.848 (p88)	rate_local12 =0.004-0.095 (p45-77)	48.12	0.65	24.57	0.08	4.993E-05
**67**	med_area_home <19.500 (p61) &prop_business <0.063 (p87) &prop_transport >0.076 (p36)	rate_local12 =0.006-0.109 (p45-81)	52.60	0.65	24.54	0.10	7.55E-05
**68**	prop_preprim_edu =0.086-0.138 (p27-86) &med_area_home <19.252 (p61) &POI_den_transport <1263.521 (p77)	rate_local12 =0.005-0.090 (p45-77)	45.33	0.68	24.50	0.12	1.013E-05
**69**	prop_preprim_edu =0.080-0.126 (p19-81) &den_road >5.313 (p26) &POI_den_transport <1481.564 (p79)	rate_local12 =0.005-0.097 (p45-77)	50.37	0.63	24.46	0.10	6.204E-06
**70**	prop_preprim_edu =0.082-0.133 (p21-84) &den_population >14692.010 (p46) &POI_den_mall_mkt <365.673 (p81)	rate_local12 =0.004-0.104 (p45-80)	45.49	0.73	24.46	0.10	2.975E-05
**71**	med_area_home <18.692 (p57) &build_area_pp <68.071 (p52)	rate_local12 =0.006-0.126 (p45-82)	57.32	0.65	24.42	0.10	4.1E-05
**72**	gender_ratio =87.051-98.202 (p11-78) &prop_preprim_edu =0.080-0.135 (p19-84)	rate_local12 =0.005-0.103 (p45-79)	59.16	0.57	24.42	0.11	3.323E-05
**73**	den_population >16031.141 (p48) &build_area_pp <61.172 (p50) &POI_den_transport <1927.571 (p85)	rate_local12 =0.005-0.106 (p45-80)	45.45	0.73	24.36	0.11	1.522E-05
**74**	den_bldg >0.083 (p29) &build_area_pp <90.579 (p62) &POI_den_mall_mkt <346.234 (p81)	rate_local12 =0.005-0.104 (p45-80)	48.82	0.67	24.34	0.12	6.422E-05
**75**	med_area_home <20.056 (p67) &den_road >5.901 (p28) &POI_den_telecom_elec <591.098 (p80)	rate_local12 =0.005-0.095 (p45-77)	50.16	0.62	24.33	0.11	1.226E-05
**76**	prop_preprim_edu =0.082-0.133 (p21-84) &den_population >14549.083 (p46) &POI_den_transport <1640.618 (p81)	rate_local12 =0.005-0.099 (p45-78)	45.09	0.71	24.32	0.10	3.251E-06
**77**	med_area_home <20.882 (p70) &den_population >15116.978 (p46) &POI_den_transport <1378.863 (p78)	rate_local12 =0.006-0.106 (p45-81)	46.26	0.72	24.32	0.07	4.879E-05
**78**	med_income <20258.781 (p73) &den_population >14878.080 (p46) &prop_business <0.059 (p87)	rate_local12 =0.005-0.116 (p45-81)	50.90	0.69	24.31	0.08	1.445E-05
**79**	prop_preprim_edu =0.081-0.132 (p20-83) &prop_transport >0.066 (p35) &POI_den_telecom_elec <664.424 (p82)	rate_local12 =0.005-0.091 (p45-77)	48.43	0.63	24.30	0.10	3.328E-05
**80**	prop_gov_insti_faci =0.025-0.143 (p36-91) &POI_den_sports <1542.023 (p90)	rate_local12 =0.004-0.127 (p45-83)	62.74	0.62	24.29	0.06	2.641E-05
**81**	den_bldg >0.079 (p27) &build_area_pp <95.175 (p64) &POI_den_sports <991.027 (p81)	rate_local12 =0.006-0.102 (p45-78)	49.68	0.65	24.29	0.12	1.85E-05

**82**	prop_preprim_edu =0.081-0.132 (p20-83) &med_area_home <15.638 (p45) &LU_entropy >0.546 (p47)	rate_local12 =0.005-0.090 (p45-77)	40.46	0.77	24.28	0.10	3.971E-05
**83**	build_area_pp <53.930 (p44) &prop_gov_insti_faci =0.024-0.143 (p35-91) &POI_den_sports <1267.682 (p85)	rate_local12 =0.005-0.108 (p45-81)	43.39	0.78	24.26	0.11	2.902E-05
**84**	prop_preprim_edu =0.081-0.129 (p20-82) &build_area_pp <67.864 (p52) &POI_den_mall_mkt <386.588 (p81)	rate_local12 =0.006-0.102 (p45-79)	46.89	0.69	24.22	0.10	6.7E-05
**85**	med_area_home <20.485 (p68) &den_road >5.901 (p28) &POI_den_mall_mkt <327.353 (p79)	rate_local12 =0.005-0.100 (p45-78)	50.93	0.63	24.20	0.12	1.125E-05
**86**	prop_over65 =0.116-0.183 (p16-72) &prop_preprim_edu =0.080-0.133 (p19-84) &build_area_pp <107.621 (p71)	rate_local12 =0.005-0.121 (p45-82)	48.16	0.74	24.18	0.10	4.717E-06
**87**	gender_ratio =87.078-97.412 (p11-76) &den_population >14870.710 (p46)	rate_local12 =0.006-0.104 (p45-80)	53.34	0.61	24.14	0.12	2.306E-06
**88**	prop_gov_insti_faci =0.025-0.143 (p36-91) &POI_den_edu <993.101 (p91)	rate_local12 =0.004-0.131 (p45-83)	63.47	0.62	24.13	0.06	5.862E-05
**89**	prop_preprim_edu =0.082-0.133 (p21-84) &den_population >14549.083 (p46) &POI_den_edu <592.070 (p80)	rate_local12 =0.005-0.105 (p45-80)	44.73	0.73	24.11	0.10	7.059E-05
**90**	prop_over65 =0.115-0.177 (p15-67) &med_area_home <17.474 (p52)	rate_local12 =0.006-0.157 (p45-88)	49.15	0.82	24.11	0.21	7.398E-08
**91**	med_area_home <18.171 (p55) &prop_gov_insti_faci =0.025-0.138 (p36-90) &POI_den_mall_mkt <312.138 (p78)	rate_local12 =0.005-0.088 (p45-76)	42.96	0.70	24.11	0.11	3.603E-05
**92**	med_area_home <20.097 (p67) &den_road >6.094 (p29) &prop_business <0.062 (p87)	rate_local12 =0.005-0.118 (p45-82)	56.79	0.63	24.08	0.08	7.078E-05
**93**	prop_preprim_edu =0.081-0.132 (p20-84) &den_road >5.613 (p27) &POI_den_edu <693.439 (p82)	rate_local12 =0.005-0.098 (p45-77)	50.97	0.62	24.08	0.09	8.257E-05
**94**	prop_over65 =0.123-0.196 (p21-80) &prop_preprim_edu =0.079-0.136 (p18-85) &med_area_home <19.662 (p64)	rate_local12 =0.005-0.099 (p45-78)	46.54	0.68	24.03	0.09	4.361E-05
**95**	gender_ratio =86.780-98.004 (p11-78) &build_area_pp <93.535 (p63)	rate_local12 =0.005-0.107 (p45-81)	59.84	0.57	24.01	0.10	6.367E-05
**96**	prop_preprim_edu =0.081-0.130 (p20-83) &prop_transport >0.055 (p33) &POI_den_edu <623.970 (p81)	rate_local12 =0.005-0.098 (p45-77)	49.08	0.64	23.99	0.10	4.875E-05
**97**	med_area_home <17.992 (p55) &prop_gov_insti_faci =0.025-0.138 (p36-90) &POI_den_sports <809.146 (p78)	rate_local12 =0.005-0.105 (p45-80)	44.27	0.74	23.98	0.12	5.021E-05
**98**	den_population >15691.896 (p47) &POI_den_edu <635.332 (p81)	rate_local12 =0.006-0.102 (p45-78)	50.07	0.64	23.97	0.14	8.013E-08
**99**	med_area_home <17.655 (p54) &den_bldg =0.071-0.351 (p25-79) &POI_den_edu <609.144 (p81)	rate_local12 =0.004-0.096 (p45-77)	43.25	0.72	23.97	0.08	6.009E-05
**100**	gender_ratio =87.188-97.999 (p11-78) &prop_open_recreation =0.019-0.142 (p37-88)	rate_local12 =0.005-0.106 (p45-81)	48.64	0.67	23.93	0.17	9.105E-09

101	med_area_home <20.056 (p67) &den_road >5.901 (p28) &POI_den_edu <612.902 (p81)	rate_local12 =0.005-0.102 (p45-79)	51.03	0.63	23.92	0.11	7.922E-06
102	den_bldg >0.080 (p27) &build_area_pp <92.118 (p63) &POI_den_transport <1280.897 (p77)	rate_local12 =0.005-0.100 (p45-78)	46.99	0.67	23.91	0.13	2.919E-05
103	med_area_home <19.500 (p61) &prop_transport >0.076 (p36) &POI_den_mall_mkt <316.409 (p79)	rate_local12 =0.005-0.095 (p45-77)	45.51	0.67	23.90	0.12	6.316E-05
104	gender_ratio <95.235 (p65) &prop_gov_insti_faci =0.025-0.142 (p36-91) &POI_den_sports <995.228 (p81)	rate_local12 =0.005-0.095 (p45-77)	45.71	0.67	23.89	0.10	1.874E-05
105	prop_preprim_edu =0.080-0.126 (p19-81) &den_road >5.313 (p26) &POI_den_sports <783.545 (p78)	rate_local12 =0.005-0.097 (p45-77)	48.60	0.64	23.88	0.11	7.836E-05
106	med_area_home <20.908 (p70) &prop_gov_insti_faci =0.018-0.135 (p32-90) &POI_den_transport <1208.412 (p77)	rate_local12 =0.005-0.090 (p45-77)	46.08	0.64	23.82	0.09	7.874E-05
107	med_area_home <18.611 (p56) &prop_gov_insti_faci =0.018-0.133 (p32-90)	rate_local12 =0.005-0.112 (p45-81)	55.01	0.62	23.80	0.10	1.066E-05
108	prop_preprim_edu =0.081-0.138 (p21-86) &build_area_pp <100.058 (p67)	rate_local12 =0.006-0.129 (p45-83)	63.74	0.61	23.76	0.10	2.801E-06
109	den_population >16619.203 (p49) &POI_den_mall_mkt <284.016 (p78)	rate_local12 =0.005-0.104 (p45-80)	46.81	0.68	23.72	0.18	1.293E-07
110	den_population >15589.742 (p47) &POI_den_telecom_elec <459.162 (p77)	rate_local12 =0.006-0.103 (p45-79)	47.31	0.67	23.71	0.17	1.29E-08
111	den_population >15024.709 (p46) &POI_den_sports <785.405 (p78)	rate_local12 =0.005-0.104 (p45-80)	48.29	0.66	23.65	0.16	2.927E-07
112	den_public_trans >17.266 (p51) &build_area_pp <92.372 (p63) &POI_den_telecom_elec <667.276 (p82)	rate_local12 =0.005-0.102 (p45-79)	42.95	0.74	23.65	0.14	3.522E-05
113	build_area_pp <62.260 (p50) &POI_den_telecom_elec <757.100 (p85)	rate_local12 =0.006-0.100 (p45-78)	52.59	0.60	23.65	0.07	7.772E-05
114	den_public_trans >17.104 (p50) &build_area_pp <85.758 (p60) &POI_den_edu <633.888 (p81)	rate_local12 =0.005-0.104 (p45-80)	41.89	0.77	23.60	0.16	2.926E-05
115	med_area_home <19.500 (p61) &prop_transport >0.076 (p36) &POI_den_transport <1281.473 (p77)	rate_local12 =0.003-0.091 (p45-77)	43.89	0.68	23.57	0.12	3.547E-05
116	prop_preprim_edu =0.081-0.128 (p20-82) &prop_transport >0.063 (p35) &POI_den_mall_mkt <316.461 (p79)	rate_local12 =0.006-0.099 (p45-78)	46.74	0.66	23.52	0.12	6.808E-05
117	den_road =7.045-24.181 (p34-88) &build_area_pp <82.481 (p58)	rate_local12 =0.006-0.106 (p45-80)	49.54	0.65	23.50	0.16	2.158E-06
118	den_public_trans >17.779 (p52) &build_area_pp <85.318 (p60) &POI_den_mall_mkt <387.886 (p81)	rate_local12 =0.005-0.105 (p45-80)	41.90	0.77	23.50	0.15	3.347E-06
119	gender_ratio <95.675 (p69) &med_area_home <19.849 (p65) &POI_den_sports <991.027 (p81)	rate_local12 =0.005-0.090 (p45-77)	49.18	0.59	23.50	0.08	7.146E-05
120	med_area_home <19.500 (p61) &prop_transport >0.076 (p36) &POI_den_sports <834.276 (p78)	rate_local12 =0.006-0.095 (p45-77)	44.78	0.67	23.45	0.11	8.057E-05

**121**	gender_ratio =85.828-99.276 (p10-79) &prop_gov_insti_faci =0.022-0.148 (p33-92) &prop_open_recreation =0.020-0.135 (p39-87)	rate_local12 =0.005-0.097 (p45-77)	42.56	0.72	23.40	0.08	5.909E-05
**122**	prop_higher_edu <0.362 (p85) &prop_transport >0.055 (p32) &POI_den_edu <1016.048 (p91)	rate_local12 =0.006-0.106 (p45-81)	60.21	0.56	23.38	0.06	7.872E-05
**123**	build_area_pp <60.752 (p50) &prop_open_recreation =0.017-0.133 (p36-86)	rate_local12 =0.004-0.132 (p45-83)	46.88	0.77	23.38	0.18	5.232E-07
**124**	gender_ratio =85.267-98.835 (p10-78) &prop_preprim_edu =0.082-0.133 (p21-84) &prop_open_recreation >0.027 (p46)	rate_local12 =0.005-0.102 (p45-79)	45.13	0.69	23.37	0.09	3.559E-05
**125**	prop_over65 =0.124-0.191 (p21-77) &build_area_pp <97.025 (p65) &POI_den_transport =27.865-2265.299 (p12-90)	rate_local12 =0.005-0.102 (p45-78)	45.13	0.69	23.34	0.09	6.606E-06
**126**	prop_preprim_edu =0.080-0.129 (p19-82) &den_road >7.114 (p34) &POI_den_mall_mkt <327.350 (p79)	rate_local12 =0.005-0.096 (p45-77)	45.56	0.66	23.34	0.12	2.246E-05
**127**	den_population >16816.044 (p49) &POI_den_transport <1281.420 (p77)	rate_local12 =0.005-0.097 (p45-77)	45.40	0.67	23.31	0.18	7.138E-08
**128**	prop_higher_edu <0.383 (p87) &den_road >7.114 (p34) &POI_den_transport <1528.257 (p80)	rate_local12 =0.005-0.099 (p45-78)	51.03	0.60	23.31	0.07	2.437E-05
**129**	gender_ratio =87.078-97.412 (p11-76) &prop_preprim_edu >0.081 (p20) &prop_transport >0.066 (p35)	rate_local12 =0.005-0.104 (p45-79)	49.92	0.63	23.31	0.06	1.211E-05
**130**	den_public_trans >17.779 (p52) &build_area_pp <85.318 (p60) &POI_den_sports <1029.490 (p82)	rate_local12 =0.004-0.103 (p45-79)	41.22	0.77	23.27	0.16	1.556E-06
**131**	med_area_home <20.485 (p68) &den_bldg >0.078 (p27) &POI_den_mall_mkt <298.672 (p78)	rate_local12 =0.005-0.100 (p45-78)	49.21	0.62	23.25	0.11	5.827E-05
**132**	med_area_home <16.550 (p50) &LU_entropy >0.549 (p48)	rate_local12 =0.005-0.088 (p45-76)	43.39	0.66	23.23	0.16	1.516E-06
**133**	build_area_pp <79.448 (p56) &prop_gov_insti_faci =0.019-0.134 (p32-90)	rate_local12 =0.005-0.109 (p45-81)	51.75	0.63	23.22	0.12	2.014E-05
**134**	prop_over65 =0.124-0.191 (p21-77) &med_area_home <17.474 (p52) &POI_den_transport <1675.179 (p82)	rate_local12 =0.004-0.094 (p45-77)	41.30	0.73	23.19	0.10	6.843E-05
**135**	gender_ratio <95.675 (p69) &build_area_pp <95.175 (p64) &POI_den_sports <991.027 (p81)	rate_local12 =0.006-0.102 (p45-78)	50.56	0.61	23.18	0.09	6.929E-05
**136**	den_population >14308.702 (p45) &build_area_pp <79.259 (p56)	rate_local12 =0.006-0.121 (p45-82)	55.20	0.63	23.12	0.11	7.498E-05
**137**	gender_ratio =86.919-97.765 (p11-77) &prop_household_3gen =0.017-0.065 (p15-87) &prop_gov_insti_faci >0.034 (p43)	rate_local12 =0.005-0.124 (p45-82)	49.69	0.69	23.09	0.09	5.379E-05
**138**	build_area_pp <60.752 (p50) &prop_open_recreation >0.028 (p47) &POI_den_mall_mkt <422.918 (p84)	rate_local12 =0.005-0.099 (p45-78)	39.80	0.78	23.08	0.14	1.441E-05
**139**	med_area_home <19.500 (p61) &prop_open_recreation >0.026 (p45) &POI_den_mall_mkt <332.133 (p79)	rate_local12 =0.005-0.089 (p45-76)	43.21	0.66	23.07	0.13	3.699E-05

**140**	prop_preprim_edu =0.084-0.144 (p24-89) &den_bldg >0.084 (p29) &POI_den_mall_mkt <380.696 (p81)	rate_local12 =0.005-0.091 (p45-77)	48.27	0.60	23.07	0.10	1.495E-05
**141**	build_area_pp <60.752 (p50) &prop_open_recreation >0.028 (p47) &POI_den_transport <1980.133 (p86)	rate_local12 =0.005-0.099 (p45-78)	40.68	0.76	23.01	0.11	1.178E-05
**142**	gender_ratio <95.784 (p69) &build_area_pp <98.252 (p66) &POI_den_mall_mkt <375.689 (p81)	rate_local12 =0.005-0.099 (p45-78)	50.39	0.60	23.00	0.09	7.146E-05
**143**	build_area_pp <90.579 (p62) &prop_transport >0.073 (p36)	rate_local12 =0.005-0.109 (p45-81)	55.92	0.59	22.97	0.11	4.923E-05
**144**	prop_over65 =0.120-0.197 (p19-80) &med_area_home <19.558 (p62) &POI_den_telecom_elec <596.572 (p80)	rate_local12 =0.005-0.101 (p45-78)	45.27	0.67	22.93	0.08	3.361E-06
**145**	gender_ratio =86.694-98.115 (p11-78) &prop_transport >0.078 (p36)	rate_local12 =0.005-0.104 (p45-80)	55.47	0.57	22.89	0.11	1.247E-05
**146**	prop_over65 =0.124-0.192 (p21-78) &med_area_home <19.126 (p61) &POI_den_mall_mkt <373.855 (p81)	rate_local12 =0.005-0.099 (p45-78)	42.88	0.70	22.86	0.10	3.968E-05
**147**	gender_ratio =87.315-99.297 (p11-79) &den_road >5.711 (p27)	rate_local12 =0.005-0.102 (p45-78)	60.92	0.53	22.85	0.08	5.979E-06
**148**	gender_ratio =87.595-102.374 (p11-88) &prop_higher_edu <0.314 (p77) &den_road >7.378 (p35)	rate_local12 =0.005-0.102 (p45-78)	49.56	0.62	22.84	0.09	4.579E-05
**149**	build_area_pp <60.752 (p50) &prop_open_recreation >0.027 (p45) &POI_den_edu <608.393 (p81)	rate_local12 =0.005-0.099 (p45-78)	39.13	0.79	22.83	0.15	3.78E-06
**150**	den_population >14878.080 (p46) &prop_business <0.059 (p87)	rate_local12 =0.005-0.116 (p45-81)	56.62	0.60	22.82	0.08	2.504E-05
**151**	build_area_pp <60.752 (p50) &prop_open_recreation >0.028 (p47) &POI_den_sports <1120.275 (p83)	rate_local12 =0.005-0.101 (p45-78)	39.25	0.79	22.79	0.14	1.912E-05
**152**	prop_higher_edu <0.309 (p76) &prop_gov_insti_faci =0.023-0.131 (p33-89) &prop_open_recreation =0.013-0.127 (p33-84)	rate_local12 =0.005-0.114 (p45-81)	42.68	0.76	22.77	0.13	9.449E-07
**153**	prop_over65 =0.120-0.187 (p20-75) &den_road >5.249 (p25) &build_area_pp <84.736 (p59)	rate_local12 =0.005-0.121 (p45-82)	44.58	0.75	22.76	0.08	8.197E-06
**154**	gender_ratio <95.784 (p69) &build_area_pp <98.252 (p66) &POI_den_telecom_elec <597.752 (p80)	rate_local12 =0.006-0.097 (p45-77)	49.16	0.60	22.70	0.09	5.582E-05
**155**	build_area_pp <64.348 (p51) &prop_gov_insti_faci =0.025-0.143 (p36-91) &POI_den_telecom_elec <462.307 (p77)	rate_local12 =0.004-0.127 (p45-83)	42.66	0.81	22.65	0.12	6.622E-05
**156**	build_area_pp <94.048 (p63) &LU_entropy >0.548 (p47)	rate_local12 =0.005-0.097 (p45-77)	46.45	0.63	22.62	0.17	2.138E-07
**157**	prop_higher_edu <0.324 (p79) &den_road >6.934 (p33) &POI_den_mall_mkt <390.782 (p82)	rate_local12 =0.005-0.095 (p45-77)	48.79	0.60	22.56	0.09	5.86E-05
**158**	prop_gov_insti_faci =0.025-0.138 (p36-90) &POI_den_transport <1237.047 (p77)	rate_local12 =0.005-0.103 (p45-79)	49.90	0.61	22.56	0.11	5.851E-05
**159**	prop_preprim_edu =0.081-0.125 (p20-81) &prop_open_recreation >0.029 (p47) &POI_den_mall_mkt <308.017 (p78)	rate_local12 =0.005-0.097 (p45-77)	41.83	0.70	22.53	0.13	2.411E-05

160	prop_over65 =0.117-0.182 (p17-71) &prop_preprim_edu >0.077 (p16) &den_population >15589.742 (p47)	rate_local12 =0.004-0.135 (p45-84)	43.99	0.80	22.52	0.10	7.031E-07
161	med_area_home <15.950 (p47)	rate_local12 =0.005-0.132 (p45-83)	59.87	0.62	22.42	0.23	3.443E-13
162	med_area_home <19.988 (p65) &prop_open_recreation >0.027 (p46) &POI_den_transport <1199.840 (p77)	rate_local12 =0.004-0.092 (p45-77)	41.65	0.68	22.42	0.13	3.791E-05
163	med_area_home <18.718 (p57) &den_bldg =0.071-0.351 (p25-79) &POI_den_telecom_elec <184.109 (p60)	rate_local12 =0.005-0.095 (p45-77)	37.89	0.78	22.35	0.16	7.28E-06
164	prop_gov_insti_faci =0.023-0.146 (p33-91) &prop_open_recreation >0.026 (p45) &POI_den_mall_mkt <399.371 (p83)	rate_local12 =0.005-0.093 (p45-77)	43.94	0.64	22.34	0.09	3.523E-05
165	prop_gov_insti_faci =0.022-0.148 (p33-92) &prop_open_recreation =0.020-0.135 (p39-87) &POI_den_sports <896.109 (p79)	rate_local12 =0.005-0.091 (p45-77)	39.59	0.72	22.33	0.12	1.005E-05
166	prop_gov_insti_faci =0.018-0.135 (p32-90) &prop_open_recreation =0.020-0.135 (p39-87) &POI_den_telecom_elec <551.719 (p79)	rate_local12 =0.006-0.091 (p45-77)	39.45	0.71	22.21	0.11	1.931E-05
167	prop_higher_edu <0.324 (p79) &den_road >6.934 (p33) &POI_den_sports <1215.425 (p84)	rate_local12 =0.005-0.088 (p45-76)	48.13	0.57	22.18	0.09	7.291E-05
168	med_income <20838.946 (p73) &prop_business <0.057 (p86) &prop_open_recreation =0.013-0.128 (p33-85)	rate_local12 =0.005-0.110 (p45-81)	44.32	0.70	22.17	0.09	4.086E-05
169	prop_preprim_edu =0.080-0.133 (p19-84) &prop_open_recreation =0.020-0.143 (p38-88)	rate_local12 =0.005-0.121 (p45-82)	50.59	0.65	22.14	0.12	5.217E-05
170	prop_preprim_edu =0.082-0.133 (p21-84) &den_population >14549.083 (p46)	rate_local12 =0.005-0.113 (p45-81)	51.67	0.62	22.09	0.10	7.629E-05
171	med_area_home <20.858 (p70) &den_bldg >0.100 (p33) &prop_business <0.064 (p87)	rate_local12 =0.006-0.109 (p45-81)	52.84	0.59	22.06	0.08	3.871E-05
172	med_area_home <17.675 (p54) &den_bldg =0.071-0.351 (p25-79) &POI_den_transport <518.406 (p64)	rate_local12 =0.006-0.092 (p45-77)	37.04	0.77	21.98	0.13	2.038E-05
173	med_area_home <17.675 (p54) &den_bldg =0.071-0.351 (p25-79)	rate_local12 =0.005-0.091 (p45-77)	43.19	0.64	21.98	0.15	1.823E-05
174	prop_over65 =0.132-0.182 (p28-71) &build_area_pp <66.434 (p52)	rate_local12 =0.005-0.136 (p45-86)	41.80	0.83	21.97	0.25	1.848E-08
175	prop_gov_insti_faci =0.023-0.146 (p33-91) &prop_open_recreation >0.026 (p45) &POI_den_edu <590.665 (p80)	rate_local12 =0.005-0.095 (p45-77)	42.72	0.66	21.95	0.10	4.216E-05
176	prop_higher_edu <0.308 (p76) &prop_open_recreation =0.020-0.136 (p39-87)	rate_local12 =0.006-0.126 (p45-82)	50.32	0.66	21.94	0.11	1.04E-05
177	build_area_pp <76.435 (p55) &prop_open_recreation >0.028 (p47) &POI_den_telecom_elec <579.555 (p80)	rate_local12 =0.005-0.099 (p45-78)	39.46	0.74	21.92	0.14	7.307E-05
178	prop_preprim_edu >0.076 (p16) &den_public_trans >19.183 (p54) &prop_business <0.051 (p86)	rate_local12 =0.005-0.114 (p45-81)	45.35	0.69	21.91	0.08	7.036E-06

179	prop_preprim_edu =0.082-0.133 (p21-84) &den_road =6.118-24.410 (p29-89)	rate_local12 =0.004-0.101 (p45-78)	50.56	0.58	21.91	0.12	9.934E-06
180	prop_over65 =0.118-0.180 (p17-71) &prop_preprim_edu =0.080-0.128 (p19-82)	rate_local12 =0.006-0.139 (p45-86)	51.40	0.69	21.89	0.16	4.205E-05
181	prop_higher_edu <0.345 (p82) &den_road =7.251-22.038 (p34-86)	rate_local12 =0.005-0.105 (p45-80)	50.58	0.60	21.86	0.10	4.416E-05
182	gender_ratio <94.290 (p58) &prop_preprim_edu =0.079-0.130 (p18-83) &POI_den_edu <713.517 (p82)	rate_local12 =0.005-0.094 (p45-77)	46.69	0.60	21.85	0.08	5.562E-05
183	prop_preprim_edu =0.080-0.125 (p19-81) &prop_open_recreation >0.029 (p47) &POI_den_sports <785.405 (p78)	rate_local12 =0.005-0.104 (p45-80)	41.42	0.71	21.84	0.13	4.325E-06
184	prop_transport >0.088 (p38) &POI_den_edu <628.086 (p81)	rate_local12 =0.005-0.096 (p45-77)	50.19	0.57	21.78	0.13	6.691E-07
185	den_public_trans >20.695 (p55) &prop_business <0.053 (p86) &POI_den_mall_mkt <539.883 (p90)	rate_local12 =0.005-0.113 (p45-81)	44.88	0.69	21.75	0.06	8.032E-05
186	med_income <19466.460 (p63) &prop_gov_insti_faci =0.021-0.133 (p33-90) &prop_open_recreation =0.013-0.128 (p33-85)	rate_local12 =0.006-0.105 (p45-80)	41.06	0.72	21.70	0.11	8.326E-05
187	gender_ratio <95.675 (p69) &prop_open_recreation >0.027 (p46) &POI_den_sports <991.027 (p81)	rate_local12 =0.004-0.098 (p45-77)	43.43	0.65	21.68	0.10	3.856E-05
188	prop_preprim_edu =0.081-0.123 (p20-80) &prop_open_recreation >0.026 (p45) &POI_den_transport <1390.516 (p79)	rate_local12 =0.005-0.100 (p45-78)	41.25	0.69	21.66	0.12	2.435E-05
189	prop_over65 =0.115-0.177 (p15-67) &prop_preprim_edu =0.081-0.136 (p20-85) &prop_gov_insti_faci =0.011-0.122 (p24-87)	rate_local12 =0.006-0.138 (p45-86)	42.70	0.79	21.46	0.11	5.292E-05
190	prop_gov_insti_faci =0.025-0.136 (p36-90) &LU_entropy >0.553 (p49)	rate_local12 =0.005-0.090 (p45-77)	42.01	0.63	21.43	0.16	1.055E-05
191	den_public_trans >17.558 (p52) &build_area_pp <86.476 (p61)	rate_local12 =0.005-0.120 (p45-82)	48.86	0.65	21.41	0.14	1.121E-05
192	den_population >14700.561 (p46) &LU_entropy >0.552 (p49)	rate_local12 =0.005-0.097 (p45-77)	41.95	0.66	21.38	0.19	6.688E-07
193	prop_over65 =0.124-0.192 (p21-78) &med_area_home <19.126 (p61) &POI_den_edu <672.277 (p81)	rate_local12 =0.005-0.099 (p45-78)	41.34	0.68	21.38	0.07	3.968E-05
194	gender_ratio =86.303-98.194 (p10-78) &den_public_trans >16.696 (p50)	rate_local12 =0.005-0.102 (p45-79)	49.20	0.59	21.35	0.13	2.899E-06
195	gender_ratio =86.007-98.493 (p10-78) &med_income <18326.955 (p61)	rate_local12 =0.005-0.114 (p45-81)	52.86	0.59	21.13	0.12	2.14E-05
196	prop_over65 =0.118-0.179 (p17-68) &gender_ratio =86.802-97.562 (p11-76) &build_area_pp <61.402 (p50)	rate_local12 =0.005-0.090 (p45-77)	36.25	0.74	21.13	0.07	5.086E-05
197	med_income <19446.921 (p63) &prop_open_recreation =0.013-0.128 (p33-85)	rate_local12 =0.005-0.114 (p45-81)	47.23	0.64	21.05	0.14	2.624E-06
198	den_public_trans >18.486 (p53) &POI_den_mall_mkt <330.394 (p79)	rate_local12 =0.005-0.099 (p45-78)	43.51	0.63	20.89	0.17	1.948E-07
199	prop_household_3gen =0.017-0.065 (p15-87) &den_road >5.711 (p27) &POI_den_sports <954.460 (p81)	rate_local12 =0.006-0.115 (p45-81)	52.16	0.59	20.83	0.07	4.562E-05
200	build_area_pp <98.252 (p66) &prop_grassland <0.042 (p60) &POI_den_mall_mkt <373.003 (p81)	rate_local12 =0.005-0.098 (p45-78)	40.91	0.66	20.83	0.15	2.241E-05

**201**	prop_over65 =0.120-0.183 (p19-72) &prop_preprim_edu >0.080 (p19) &den_road >5.249 (p25)	rate_local12 =0.005-0.134 (p45-84)	46.48	0.70	20.82	0.11	6.66E-07
**202**	build_area_pp <98.252 (p66) &prop_grassland <0.042 (p60) &POI_den_edu <675.056 (p82)	rate_local12 =0.005-0.098 (p45-78)	40.92	0.66	20.78	0.15	2.241E-05
**203**	prop_higher_edu <0.319 (p79) &prop_transport >0.079 (p36)	rate_local12 =0.005-0.122 (p45-82)	57.62	0.58	20.78	0.09	4.833E-05
**204**	gender_ratio <95.051 (p64) &prop_preprim_edu =0.079-0.130 (p18-83) &POI_den_telecom_elec <657.018 (p82)	rate_local12 =0.004-0.090 (p45-77)	46.14	0.56	20.76	0.07	7.37E-05
**205**	med_area_home <20.056 (p67) &den_road >5.901 (p28)	rate_local12 =0.006-0.114 (p45-81)	60.70	0.54	20.74	0.06	4.799E-05
**206**	prop_business <0.059 (p87) &prop_open_recreation =0.018-0.141 (p36-88)	rate_local12 =0.005-0.116 (p45-81)	52.18	0.59	20.70	0.08	5.102E-05
**207**	den_public_trans >19.385 (p54) &POI_den_edu <645.069 (p81)	rate_local12 =0.005-0.099 (p45-78)	43.91	0.62	20.69	0.16	1.551E-07
**208**	prop_business <0.063 (p87) &prop_transport >0.076 (p36)	rate_local12 =0.006-0.109 (p45-81)	58.13	0.54	20.64	0.08	3.5E-05
**209**	den_public_trans >19.022 (p53) &POI_den_transport <1450.759 (p79)	rate_local12 =0.005-0.096 (p45-77)	42.28	0.63	20.59	0.17	1.324E-08
**210**	med_income <19466.460 (p63) &prop_open_recreation =0.013-0.128 (p33-85) &POI_den_transport <1281.420 (p77)	rate_local12 =0.005-0.103 (p45-79)	38.69	0.71	20.51	0.11	2.809E-05
**211**	prop_transport >0.091 (p39) &LU_entropy >0.559 (p50)	rate_local12 =0.005-0.102 (p45-79)	42.57	0.64	20.47	0.16	1.503E-05
**212**	build_area_pp <99.427 (p67) &prop_grassland <0.042 (p60) &POI_den_sports <972.367 (p81)	rate_local12 =0.005-0.098 (p45-78)	40.04	0.67	20.41	0.15	5.946E-05
**213**	prop_over65 =0.118-0.179 (p17-68) &gender_ratio =86.802-97.562 (p11-76) &prop_open_recreation >0.029 (p48)	rate_local12 =0.005-0.090 (p45-77)	34.64	0.75	20.36	0.15	3.468E-05
**214**	prop_gov_insti_faci =0.025-0.143 (p36-91) &POI_den_telecom_elec <175.141 (p59)	rate_local12 =0.005-0.101 (p45-78)	38.29	0.71	20.34	0.21	3.289E-05
**215**	den_road >7.114 (p34) &POI_den_mall_mkt <327.350 (p79)	rate_local12 =0.005-0.096 (p45-77)	51.69	0.53	20.31	0.12	6.109E-06
**216**	prop_over65 =0.122-0.177 (p20-66) &med_area_home <17.425 (p52) &LU_entropy >0.553 (p49)	rate_local12 =0.006-0.081 (p45-75)	30.66	0.87	20.30	0.21	5.077E-06
**217**	build_area_pp <66.740 (p52)	rate_local12 =0.005-0.113 (p45-81)	58.45	0.54	20.29	0.19	4.019E-09
**218**	den_road >7.114 (p34) &POI_den_transport <1528.257 (p80)	rate_local12 =0.005-0.090 (p45-77)	51.27	0.51	20.28	0.11	9.674E-07
**219**	prop_gov_insti_faci =0.025-0.143 (p36-91)	rate_local12 =0.004-0.131 (p45-83)	64.44	0.56	20.20	0.18	8.888E-09
**220**	gender_ratio <95.235 (p65) &prop_open_recreation >0.033 (p50) &POI_den_mall_mkt <349.960 (p81)	rate_local12 =0.005-0.087 (p45-76)	38.27	0.65	20.20	0.10	7.101E-05
**221**	prop_publicResid >0.050 (p71)	rate_local12 =0.005-0.112 (p45-81)	41.65	0.68	20.17	0.33	1.466E-11
**222**	prop_open_recreation =0.020-0.135 (p39-87) &LU_entropy >0.551 (p49)	rate_local12 =0.005-0.103 (p45-79)	40.35	0.67	20.03	0.16	1.26E-06
**223**	prop_preprim_edu >0.077 (p16) &prop_transport >0.091 (p39)	rate_local12 =0.005-0.114 (p45-81)	56.53	0.55	19.88	0.07	5.036E-05
**224**	den_road >7.757 (p37) &POI_den_edu <628.086 (p81)	rate_local12 =0.005-0.096 (p45-77)	50.49	0.53	19.87	0.11	1.454E-06
**225**	den_road >6.004 (p29) &LU_entropy >0.548 (p47)	rate_local12 =0.005-0.097 (p45-77)	47.81	0.55	19.82	0.09	1.002E-05

**226**	prop_open_recreation >0.027 (p46) &POI_den_telecom_elec <539.258 (p78)	rate_local12 =0.005-0.090 (p45-77)	46.21	0.54	19.75	0.11	1.374E-05
**227**	prop_over65 =0.118-0.179 (p17-68) &build_area_pp <95.055 (p64) &LU_entropy >0.536 (p43)	rate_local12 =0.005-0.091 (p45-77)	33.09	0.78	19.74	0.18	2.336E-05
**228**	prop_over65 =0.120-0.197 (p19-80) &prop_open_recreation >0.025 (p45)	rate_local12 =0.006-0.156 (p45-88)	53.20	0.66	19.66	0.12	2.306E-05
**229**	den_population >15589.742 (p47)	rate_local12 =0.005-0.131 (p45-83)	63.59	0.55	19.66	0.17	2.319E-10
**230**	prop_higher_edu <0.296 (p75) &prop_gov_insti_faci >0.029 (p40)	rate_local12 =0.006-0.120 (p45-82)	52.81	0.58	19.56	0.10	8.002E-05
**231**	prop_open_recreation >0.029 (p47) &POI_den_mall_mkt <280.812 (p78)	rate_local12 =0.005-0.090 (p45-77)	44.28	0.56	19.55	0.12	1.015E-05
**232**	med_income <18450.148 (p61) &den_road >5.958 (p28)	rate_local12 =0.005-0.117 (p45-82)	54.35	0.56	19.53	0.10	7.866E-05
**233**	prop_open_recreation >0.029 (p47) &POI_den_sports <785.405 (p78)	rate_local12 =0.006-0.092 (p45-77)	44.31	0.56	19.37	0.12	1.015E-05
**234**	den_bldg =0.071-0.351 (p25-79) &build_area_pp <91.239 (p62)	rate_local12 =0.005-0.095 (p45-77)	43.38	0.57	19.03	0.12	6.882E-05
**235**	prop_transport >0.103 (p43) &POI_den_transport <543.613 (p65)	rate_local12 =0.005-0.091 (p45-77)	34.42	0.70	18.97	0.27	1.406E-07
**236**	gender_ratio <94.290 (p58) &prop_open_recreation >0.035 (p52) &POI_den_transport <1626.628 (p81)	rate_local12 =0.005-0.095 (p45-77)	36.78	0.66	18.97	0.12	2.563E-05
**237**	prop_preprim_edu >0.078 (p17) &den_public_trans >17.390 (p52)	rate_local12 =0.005-0.118 (p45-82)	50.96	0.57	18.88	0.08	7.494E-06
**238**	prop_transport >0.104 (p44) &POI_den_sports <351.464 (p64)	rate_local12 =0.005-0.092 (p45-77)	33.62	0.71	18.82	0.28	4.268E-09
**239**	den_road >6.555 (p30) &prop_business <0.065 (p87)	rate_local12 =0.004-0.116 (p45-81)	61.71	0.51	18.62	0.06	6.877E-05
**240**	den_road >5.303 (p26) &prop_open_recreation =0.013-0.127 (p33-84) &POI_den_telecom_elec <170.060 (p59)	rate_local12 =0.006-0.099 (p45-78)	33.18	0.74	18.56	0.14	2.866E-05
**241**	prop_transport >0.106 (p44) &POI_den_mall_mkt <115.474 (p64)	rate_local12 =0.004-0.088 (p45-76)	32.86	0.70	18.50	0.28	1.568E-09
**242**	prop_preprim_edu =0.080-0.125 (p19-81)	rate_local12 =0.005-0.114 (p45-81)	65.28	0.49	18.35	0.14	1.929E-08
**243**	den_public_trans >19.855 (p54) &LU_entropy >0.556 (p50)	rate_local12 =0.005-0.104 (p45-80)	35.23	0.70	18.30	0.21	4.693E-06
**244**	prop_household_3gen =0.017-0.065 (p15-87) &den_road >5.711 (p27)	rate_local12 =0.006-0.124 (p45-82)	60.91	0.53	18.22	0.08	6.733E-05
**245**	prop_over65 =0.128-0.180 (p24-70) &med_area_home <17.547 (p53) &den_bldg =0.071-0.351 (p25-79)	rate_local12 =0.006-0.082 (p45-75)	27.47	0.87	18.19	0.23	4.268E-05
**246**	prop_over65 =0.142-0.212 (p36-89) &den_road >5.958 (p28) &POI_den_mall_mkt <439.465 (p85)	rate_local12 =0.005-0.107 (p45-81)	40.41	0.62	18.13	0.11	5.96E-05
**247**	den_public_trans >24.563 (p58) &prop_business <0.065 (p87)	rate_local12 =0.004-0.116 (p45-81)	41.88	0.63	18.09	0.12	3.298E-06
**248**	prop_over65 =0.142-0.212 (p36-89) &den_road >5.958 (p28) &POI_den_transport <2239.160 (p90)	rate_local12 =0.005-0.122 (p45-82)	43.52	0.63	18.06	0.09	5.765E-05
**249**	prop_open_recreation =0.013-0.127 (p33-84) &prop_transport >0.073 (p36) &POI_den_telecom_elec <239.361 (p65)	rate_local12 =0.004-0.104 (p45-80)	32.12	0.76	17.95	0.14	5.223E-05
**250**	prop_transport >0.110 (p47) &POI_den_telecom_elec <175.141 (p59)	rate_local12 =0.004-0.091 (p45-77)	29.71	0.79	17.94	0.36	6.562E-10
**251**	gender_ratio =87.053-98.128 (p11-78)	rate_local12 =0.005-0.108 (p45-81)	66.61	0.47	17.92	0.13	1.976E-08
**252**	med_income <21881.055 (p75) &POI_den_transport =149.484-1813.275 (p40-84)	rate_local12 =0.005-0.108 (p45-81)	41.11	0.61	17.91	0.12	8.321E-05

**253**	prop_open_recreation =0.022-0.138 (p39-87)	rate_local12 =0.005-0.128 (p45-83)	55.07	0.56	17.90	0.18	3.556E-09
**254**	den_bldg >0.114 (p37) &POI_den_telecom_elec <523.327 (p78)	rate_local12 =0.005-0.093 (p45-77)	46.40	0.52	17.90	0.12	2.383E-05
**255**	den_road >7.783 (p37) &POI_den_telecom_elec <175.141 (p59)	rate_local12 =0.005-0.093 (p45-77)	34.19	0.66	17.87	0.25	2.222E-07
**256**	build_area_pp <95.886 (p65) &prop_shrubland <0.068 (p50) &POI_den_mall_mkt <349.458 (p81)	rate_local12 =0.005-0.094 (p45-77)	32.35	0.71	17.82	0.19	4.285E-05
**257**	build_area_pp <95.886 (p65) &prop_shrubland <0.068 (p50) &POI_den_transport <1578.798 (p81)	rate_local12 =0.005-0.098 (p45-78)	32.76	0.71	17.73	0.19	4.285E-05
**258**	gender_ratio <95.235 (p65) &POI_den_sports <995.228 (p81)	rate_local12 =0.005-0.095 (p45-77)	53.33	0.48	17.65	0.07	3.397E-06
**259**	gender_ratio <94.290 (p58) &POI_den_transport <1626.628 (p81)	rate_local12 =0.005-0.095 (p45-77)	50.33	0.49	17.63	0.08	1.295E-05
**260**	gender_ratio <94.290 (p58) &POI_den_mall_mkt <345.003 (p81)	rate_local12 =0.005-0.095 (p45-77)	49.72	0.50	17.59	0.08	7.435E-06
**261**	den_bldg >0.124 (p39) &POI_den_mall_mkt <301.168 (p78)	rate_local12 =0.005-0.090 (p45-77)	43.82	0.52	17.48	0.13	3.233E-05
**262**	prop_higher_edu <0.308 (p76) &den_bldg >0.117 (p38)	rate_local12 =0.005-0.118 (p45-82)	52.61	0.54	17.47	0.10	3.215E-05
**263**	den_bldg >0.123 (p39) &POI_den_edu <645.069 (p81)	rate_local12 =0.005-0.095 (p45-77)	46.89	0.51	17.47	0.11	3.757E-05
**264**	den_bldg >0.124 (p39) &POI_den_transport <1281.420 (p77)	rate_local12 =0.005-0.087 (p45-76)	42.96	0.51	17.47	0.13	3.807E-05
**265**	gender_ratio <94.372 (p59) &POI_den_telecom_elec <632.455 (p82)	rate_local12 =0.005-0.099 (p45-78)	51.19	0.50	17.41	0.08	7.422E-05
**266**	prop_over65 =0.127-0.178 (p23-67) &LU_entropy >0.557 (p50)	rate_local12 =0.005-0.095 (p45-77)	31.94	0.70	17.33	0.23	6.65E-06
**267**	den_road >8.230 (p39) &POI_den_sports <342.772 (p64)	rate_local12 =0.005-0.088 (p45-76)	34.13	0.62	17.25	0.22	5.588E-07
**268**	med_area_home <19.900 (p65) &prop_grassland <0.046 (p61) &POI_den_sports <362.105 (p64)	rate_local12 =0.004-0.096 (p45-77)	29.97	0.76	17.23	0.17	3.615E-05
**269**	den_road =7.251-22.038 (p34-86)	rate_local12 =0.005-0.103 (p45-79)	53.18	0.49	17.19	0.16	8.734E-07
**270**	den_bldg =0.094-0.328 (p31-76) &prop_open_recreation =0.017-0.131 (p36-86)	rate_local12 =0.005-0.097 (p45-77)	34.28	0.64	17.08	0.17	4.294E-05
**271**	den_public_trans >19.385 (p54) &POI_den_sports <336.087 (p63)	rate_local12 =0.005-0.099 (p45-78)	29.68	0.76	16.91	0.30	6.272E-07
**272**	den_bldg >0.100 (p33) &prop_business <0.064 (p87)	rate_local12 =0.005-0.110 (p45-81)	57.23	0.49	16.56	0.06	8.087E-05
**273**	prop_transport =0.088-0.336 (p39-88)	rate_local12 =0.005-0.091 (p45-77)	48.74	0.47	16.38	0.16	9.057E-08
**274**	prop_preprim_edu >0.076 (p16) &den_public_trans >19.183 (p54) &POI_den_sports <315.206 (p62)	rate_local12 =0.005-0.106 (p45-81)	28.42	0.80	16.36	0.04	1.251E-05
**275**	den_public_trans >20.275 (p55) &POI_den_telecom_elec <175.141 (p59)	rate_local12 =0.005-0.094 (p45-77)	26.48	0.83	16.27	0.38	1.05E-08
**276**	prop_grassland <0.042 (p60) &POI_den_edu <675.056 (p82)	rate_local12 =0.005-0.098 (p45-78)	45.33	0.51	16.14	0.11	3.567E-05
**277**	prop_grassland <0.040 (p59) &POI_den_telecom_elec <175.141 (p59)	rate_local12 =0.005-0.091 (p45-77)	29.86	0.66	15.73	0.28	2.02E-07
**278**	LU_entropy >0.543 (p46)	rate_local12 =0.005-0.103 (p45-79)	54.68	0.47	15.70	0.13	1.235E-06
**279**	den_bldg >0.124 (p39) &POI_den_sports <342.772 (p64)	rate_local12 =0.006-0.085 (p45-76)	32.23	0.59	15.70	0.21	6.974E-06
**280**	den_bldg =0.080-0.296 (p27-73) &prop_rural_set <0.063 (p78) &POI_den_telecom_elec <270.226 (p67)	rate_local12 =0.005-0.106 (p45-80)	30.48	0.69	15.60	0.15	1.579E-05
**281**	den_public_trans >35.072 (p68) &prop_business <0.076 (p89) &POI_den_sports <1375.127 (p87)	rate_local12 =0.003-0.110 (p45-81)	29.16	0.73	15.20	0.10	5.496E-05

**282**	prop_shrubland <0.089 (p55) &POI_den_mall_mkt <355.591 (p81)	rate_local12 =0.004-0.084 (p45-75)	37.91	0.50	15.14	0.13	2.641E-05
**283**	prop_over65 =0.134-0.178 (p29-67)	rate_local12 =0.004-0.150 (p45-87)	48.30	0.60	15.10	0.19	5.755E-06
**284**	prop_grassland <0.042 (p60) &POI_den_mall_mkt <150.646 (p67)	rate_local12 =0.004-0.095 (p45-77)	33.94	0.58	15.02	0.18	1.638E-05
**285**	prop_over65 =0.131-0.195 (p28-80) &den_public_trans >26.846 (p59) &prop_business <0.065 (p87)	rate_local12 =0.005-0.117 (p45-82)	28.72	0.75	14.94	0.14	3.741E-05
**286**	prop_shrubland <0.089 (p55) &POI_den_transport <1458.002 (p79)	rate_local12 =0.006-0.093 (p45-77)	38.04	0.52	14.90	0.14	3.555E-05
**287**	den_public_trans >16.696 (p50)	rate_local12 =0.005-0.126 (p45-82)	57.19	0.51	14.80	0.13	7.127E-07
**288**	den_public_trans >35.072 (p68) &prop_gov_insti_faci =0.013-0.147 (p29-91) &POI_den_sports <1375.127 (p87)	rate_local12 =0.004-0.135 (p45-85)	31.07	0.74	14.69	0.07	4.98E-05
**289**	den_bldg =0.094-0.328 (p31-76)	rate_local12 =0.005-0.071 (p45-73)	40.63	0.43	14.67	0.16	5.131E-06
**290**	prop_grassland <0.042 (p60) &POI_den_transport <434.482 (p59)	rate_local12 =0.005-0.082 (p45-75)	28.00	0.62	14.59	0.26	4.821E-06
**291**	den_public_trans >35.072 (p68) &prop_gov_insti_faci =0.013-0.147 (p29-91) &POI_den_telecom_elec <946.930 (p91)	rate_local12 =0.004-0.135 (p45-85)	31.40	0.73	14.51	0.08	2.548E-05
**292**	den_bldg <0.281 (p70) &prop_transport >0.103 (p44)	rate_local12 =0.005-0.072 (p45-73)	29.92	0.53	14.46	0.15	6.393E-05
**293**	prop_grassland <0.042 (p60) &POI_den_sports <279.052 (p60)	rate_local12 =0.004-0.093 (p45-77)	28.67	0.62	14.10	0.24	1.583E-05
**294**	POI_den_mall_mkt =31.091-261.421 (p42-77)	rate_local12 =0.005-0.103 (p45-79)	37.55	0.52	13.46	0.19	1.277E-06
**295**	prop_over65 =0.132-0.195 (p28-80) &den_public_trans >33.002 (p67) &POI_den_sports <764.402 (p78)	rate_local12 =0.005-0.123 (p45-82)	21.65	0.86	12.27	0.13	3.534E-05
**296**	prop_household_3gen =0.023-0.062 (p19-86)	rate_local12 =0.004-0.102 (p45-78)	58.15	0.42	12.13	0.09	1.588E-05
**297**	den_road <14.354 (p64) &prop_transport >0.111 (p48)	rate_local12 =0.006-0.097 (p45-77)	24.28	0.63	11.85	0.19	5.325E-05
**298**	prop_private_resid >0.044 (p48) &POI_den_sports <283.563 (p60)	rate_local12 =0.005-0.086 (p45-76)	25.71	0.56	11.85	0.19	1.275E-05
**299**	med_income <19063.503 (p63)	rate_local12 =0.007-0.133 (p45-84)	63.97	0.47	11.72	0.09	4.749E-05
**300**	prop_shrubland <0.068 (p50) &POI_den_telecom_elec <173.241 (p59)	rate_local12 =0.005-0.083 (p45-75)	21.66	0.63	11.45	0.28	1.08E-05
**301**	prop_higher_edu <0.245 (p64)	rate_local12 =0.007-0.036 (p45-59)	26.42	0.20	8.61	0.06	7.147E-05
**302**	prop_private_resid =0.043-0.162 (p48-78)	rate_local12 =0.004-0.062 (p45-71)	23.72	0.37	8.56	0.13	7.922E-05
**303**	prop_shrubland <0.020 (p36) &POI_den_sports <204.087 (p55)	rate_local12 =0.003-0.078 (p45-74)	8.61	0.76	5.35	0.45	6.349E-05
**304**	prop_rural_set >0.051 (p75) &prop_gov_insti_faci >0.086 (p73)	rate_local12 =0.006-0.033 (p45-58)	3.52	0.73	2.91	0.53	7.621E-05
**305**	prop_private_resid <0.098 (p66) &prop_publicResid <0.017 (p63)	rate_local12 <0.004 (p45)	57.79	0.71	20.80	0.15	1.543E-05
**306**	prop_gov_insti_faci <0.025 (p36)	rate_local12 <0.004 (p45)	54.86	0.71	19.79	0.26	2.083E-08
**307**	prop_publicResid <0.001 (p48)	rate_local12 <0.005 (p45)	65.00	0.63	18.28	0.18	4.83E-07
**308**	prop_open_recreation <0.017 (p36)	rate_local12 <0.005 (p45)	52.91	0.69	18.17	0.24	1.02E-07
**309**	den_population <15589.742 (p47)	rate_local12 <0.005 (p45)	63.62	0.63	18.06	0.18	1.272E-08
**310**	prop_preprim_edu >0.098 (p54) &prop_publicResid <0.011 (p61)	rate_local12 <0.005 (p45)	42.75	0.72	15.82	0.16	4.813E-05
**311**	prop_private_resid <0.009 (p26)	rate_local12 <0.004 (p45)	41.31	0.73	15.77	0.28	2.089E-07
**312**	den_public_trans <16.696 (p50)	rate_local12 <0.005 (p45)	61.80	0.60	15.23	0.15	5.857E-06
**313**	prop_transport <0.088 (p39)	rate_local12 <0.005 (p45)	53.39	0.63	14.86	0.17	5.076E-05

**314**	prop_preprim_edu >0.104 (p62) &prop_private_resid <0.017 (p33)	rate_local12 <0.004 (p45)	33.52	0.77	13.73	0.11	5.207E-05
**315**	den_road <7.251 (p34)	rate_local12 <0.005 (p45)	46.67	0.64	13.43	0.18	5.593E-05
**316**	med_area_home >15.950 (p47)	rate_local12 <0.005 (p45)	66.61	0.57	13.35	0.11	4.79E-05
**317**	prop_business <0.000 (p23)	rate_local12 <0.002 (p45)	35.94	0.71	12.97	0.26	1.506E-05
**318**	prop_utilities <0.001 (p20)	rate_local12 <0.004 (p45)	31.85	0.76	12.79	0.30	7.404E-06
**319**	den_bldg <0.055 (p20)	rate_local12 <0.005 (p45)	31.06	0.73	11.86	0.28	3.534E-05
**320**	prop_over65 >0.198 (p82)	rate_local12 <0.004 (p45)	29.43	0.74	11.38	0.29	5.639E-05
**321**	prop_grassland >0.135 (p81)	rate_local12 <0.003 (p45)	28.59	0.72	10.48	0.26	6.798E-05
**322**	prop_household_3gen >0.062 (p86)	rate_local12 <0.004 (p45)	23.16	0.77	9.49	0.31	7.69E-05

(g) Rules for wave-1 imported case rate; POI density was used

No.	Antecedent	Consequent	Supp	Conf	Lev	Imp	*P*
**1**	prop_higher_edu >0.209 (p53) &prop_industrial <0.010 (p71)	rate_imported12 >0.087 (p67)	54.47	0.66	25.02	0.07	4.865E-05
**2**	prop_higher_edu >0.213 (p54) &build_area_pp >62.720 (p50)	rate_imported12 >0.122 (p71)	42.50	0.67	24.21	0.14	7.081E-05
**3**	prop_higher_edu >0.193 (p47) &med_area_home >17.271 (p52)	rate_imported12 >0.111 (p71)	45.05	0.62	22.89	0.13	1.799E-05
**4**	prop_preprim_edu <0.117 (p78) &build_area_pp >60.189 (p50) &prop_industrial <0.011 (p73)	rate_imported12 >0.094 (p68)	45.31	0.68	22.75	0.10	6.715E-05
**5**	prop_preprim_edu <0.135 (p84) &med_area_home >16.429 (p49)	rate_imported12 >0.081 (p65)	54.56	0.60	21.10	0.08	6.929E-05
**6**	med_income >16651.172 (p51) &build_area_pp >69.910 (p53) &prop_agricultural <0.068 (p83)	rate_imported12 >0.084 (p66)	40.24	0.75	20.65	0.11	7.63E-05
**7**	prop_preprim_edu <0.099 (p55) &prop_publicResid <0.014 (p62)	rate_imported12 >0.101 (p68)	44.59	0.60	20.57	0.15	1.505E-05
**8**	build_area_pp >60.696 (p50) &prop_industrial <0.006 (p64)	rate_imported12 >0.098 (p68)	44.52	0.61	20.17	0.15	3.171E-06
**9**	med_area_home >17.271 (p52) &prop_rural_set <0.050 (p75)	rate_imported12 >0.111 (p71)	40.51	0.60	19.92	0.12	6.752E-05
**10**	med_income >17090.451 (p53) &prop_industrial <0.000 (p44)	rate_imported12 >0.100 (p68)	37.37	0.70	19.91	0.18	1.157E-05
**11**	build_area_pp >63.743 (p51) &prop_rural_set <0.010 (p58) &prop_industrial <0.017 (p79)	rate_imported12 >0.117 (p71)	34.19	0.70	19.73	0.10	2.362E-05
**12**	prop_preprim_edu <0.135 (p84) &build_area_pp >67.205 (p52)	rate_imported12 >0.122 (p71)	43.72	0.52	19.69	0.09	4.473E-05
**13**	build_area_pp >69.910 (p53) &prop_agricultural <0.068 (p83)	rate_imported12 >0.084 (p66)	46.58	0.63	19.66	0.13	5.865E-07
**14**	prop_publicResid <0.010 (p60) &prop_industrial <0.010 (p71)	rate_imported12 >0.100 (p68)	50.71	0.53	19.56	0.10	2.048E-05
**15**	med_area_home >16.755 (p50)	rate_imported12 >0.115 (p71)	50.99	0.47	18.58	0.17	9.407E-08
**16**	build_area_pp >68.387 (p52) &prop_rural_set <0.024 (p65)	rate_imported12 >0.094 (p68)	39.59	0.62	18.07	0.15	7.401E-06
**17**	prop_publicResid <0.035 (p69)	rate_imported12 >0.111 (p71)	60.36	0.41	15.60	0.11	9.516E-08
**18**	build_area_pp >64.307 (p51)	rate_imported12 >0.119 (p71)	47.49	0.43	15.47	0.14	1.109E-05
**19**	den_public_trans =3.582-84.723 (p25-91) &build_area_pp >71.228 (p53)	rate_imported12 >0.084 (p66)	34.30	0.65	15.23	0.16	6.249E-05
**20**	prop_higher_edu =0.263-0.402 (p66-90)	rate_imported12 >0.101 (p68)	30.69	0.61	14.21	0.28	1.212E-06
**21**	build_area_pp >75.510 (p55) &prop_gov_insti_faci >0.025 (p36)	rate_imported12 >0.068 (p62)	34.91	0.68	13.94	0.15	6.787E-06
**22**	prop_industrial <0.000 (p44)	rate_imported12 >0.094 (p68)	45.19	0.49	13.87	0.15	3.763E-06
**23**	prop_preprim_edu <0.084 (p25)	rate_imported12 >0.114 (p71)	29.21	0.55	13.16	0.25	1.013E-05
**24**	LU_entropy <0.561 (p51)	rate_imported12 >0.096 (p68)	49.34	0.45	12.72	0.12	5.049E-05
**25**	med_income =19043.072-37169.137 (p63-92)	rate_imported12 >0.109 (p70)	31.37	0.51	12.44	0.20	6.84E-07
**26**	ave_household_size >3.251 (p87)	rate_imported12 >0.109 (p70)	20.28	0.76	11.96	0.45	6.175E-09
**27**	prop_over65 <0.133 (p28)	rate_imported12 >0.080 (p64)	34.12	0.56	11.49	0.19	5.436E-05
**28**	med_income >37169.137 (p92)	rate_imported12 >0.109 (p70)	16.02	0.92	10.66	0.61	4.383E-08
**29**	den_population =845.965-18750.995 (p14-50)	rate_imported12 >0.082 (p65)	38.62	0.51	10.58	0.14	6.303E-05
**30**	prop_higher_edu >0.402 (p90)	rate_imported12 >0.101 (p68)	16.66	0.83	10.13	0.51	3.384E-08

**31**	build_area_pp >69.988 (p53) &POI_den_telecom_elec >420.031 (p76)	rate_imported12 >0.228 (p81)	14.05	0.54	9.17	0.23 3.174E-05
**32**	den_population <7302.660 (p37) &prop_private_resid =0.022-0.228 (p36-85)	rate_imported12 >0.090 (p67)	16.83	0.73	8.81	0.32 3.955E-05
**33**	den_population =8304.569-47145.016 (p39-74) &POI_den_telecom_elec >206.748 (p64)	rate_imported12 >0.094 (p68)	20.60	0.56	8.06	0.16 2.352E-05
**34**	gender_ratio >103.674 (p89)	rate_imported12 >0.205 (p80)	11.96	0.49	7.21	0.30 7.005E-05
**35**	build_area_pp >67.949 (p52) &prop_private_resid >0.220 (p85)	rate_imported12 >0.180 (p79)	10.17	0.74	7.19	0.38 2.466E-05
**36**	den_bldg <0.058 (p21) &prop_private_resid =0.026-0.163 (p39-78)	rate_imported12 >0.192 (p80)	7.05	0.96	5.52	0.64 3.457E-06
**37**	build_area_pp <69.910 (p53) &prop_agricultural >0.068 (p83)	rate_imported12 >0.084 (p66)	4.65	0.59	1.78	0.35 3.577E-05
**38**	gender_ratio <96.153 (p71) &prop_preprim_edu <0.124 (p80) &med_area_home <17.388 (p52)	rate_imported12 =0.003-0.123 (p36-71)	58.18	0.71	28.90	0.07 7.727E-05
**39**	prop_preprim_edu <0.130 (p82) &med_area_home <17.522 (p53) &POI_den_transport <1435.842 (p79)	rate_imported12 =0.002-0.101 (p36-68)	50.40	0.73	27.80	0.11 2.704E-05
**40**	prop_preprim_edu <0.130 (p82) &med_area_home <17.522 (p53) &POI_den_mall_mkt <125.736 (p65)	rate_imported12 =0.003-0.093 (p36-68)	44.03	0.81	26.97	0.15 3.205E-07
**41**	prop_preprim_edu <0.130 (p82) &med_area_home <17.522 (p53) &POI_den_sports <425.600 (p66)	rate_imported12 =0.002-0.101 (p36-68)	45.43	0.80	26.97	0.14 4.385E-07
**42**	prop_preprim_edu <0.131 (p83) &med_area_home <17.376 (p52) &POI_den_telecom_elec <210.823 (p64)	rate_imported12 =0.003-0.096 (p36-68)	43.85	0.82	26.88	0.14 1.749E-07
**43**	gender_ratio <95.332 (p66) &prop_preprim_edu <0.132 (p83) &med_income <20537.185 (p73)	rate_imported12 =0.003-0.091 (p36-67)	53.85	0.61	26.74	0.10 1.251E-05
**44**	prop_preprim_edu <0.132 (p83) &med_income <20537.185 (p73) &build_area_pp <70.879 (p53)	rate_imported12 =0.001-0.110 (p36-71)	54.38	0.66	26.71	0.08 8.546E-06
**45**	prop_preprim_edu <0.131 (p83) &med_income <20826.029 (p73) &den_public_trans =8.583-88.534 (p38-91)	rate_imported12 =0.003-0.094 (p36-68)	49.86	0.67	26.47	0.06 5.183E-05
**46**	med_area_home <18.538 (p56) &den_public_trans =6.785-83.086 (p34-90)	rate_imported12 =0.002-0.118 (p36-71)	55.54	0.67	26.41	0.14 2.195E-05
**47**	med_area_home <17.126 (p52) &build_area_pp <62.684 (p50)	rate_imported12 =0.004-0.085 (p36-66)	50.77	0.62	26.35	0.11 1.904E-05
**48**	gender_ratio <94.297 (p58) &med_area_home <18.565 (p56)	rate_imported12 =0.003-0.122 (p36-71)	56.36	0.66	26.19	0.13 3.363E-05
**49**	gender_ratio <96.137 (p71) &prop_preprim_edu <0.133 (p84) &prop_higher_edu <0.296 (p75)	rate_imported12 =0.003-0.090 (p36-67)	56.14	0.58	26.19	0.09 2.004E-05
**50**	prop_preprim_edu <0.134 (p84) &prop_higher_edu <0.236 (p62) &prop_private_resid =0.010-0.219 (p27-84)	rate_imported12 =0.003-0.090 (p36-67)	44.92	0.73	26.16	0.10 6.142E-07
**51**	prop_preprim_edu <0.133 (p84) &prop_higher_edu <0.242 (p63) &prop_agricultural <0.049 (p80)	rate_imported12 =0.003-0.079 (p36-64)	48.83	0.61	26.16	0.10 1.749E-06

**52**	prop_preprim_edu <0.134 (p84) &med_income <18486.007 (p61) &prop_private_resid =0.010-0.219 (p27-84)	rate_imported12 =0.003-0.088 (p36-67)	45.68	0.70	25.96	0.09	4.844E-06
**53**	prop_preprim_edu <0.124 (p80) &prop_higher_edu <0.249 (p64) &prop_transport >0.057 (p33)	rate_imported12 =0.003-0.086 (p36-66)	47.95	0.65	25.86	0.10	6.649E-05
**54**	prop_preprim_edu <0.135 (p84) &med_income <20537.185 (p73) &prop_agricultural <0.049 (p80)	rate_imported12 =0.003-0.082 (p36-65)	55.02	0.54	25.73	0.07	1.924E-05
**55**	prop_preprim_edu <0.132 (p83) &prop_higher_edu <0.269 (p68) &med_area_home <20.396 (p68)	rate_imported12 =0.005-0.083 (p36-65)	52.34	0.56	25.11	0.07	6.099E-05
**56**	prop_preprim_edu <0.127 (p82) &med_income <19791.098 (p65) &POI_den_transport =40.112-1204.130 (p17-77)	rate_imported12 =0.003-0.095 (p36-68)	46.03	0.69	24.95	0.13	1.399E-05
**57**	prop_preprim_edu <0.133 (p84) &prop_higher_edu <0.244 (p63) &build_area_pp <67.205 (p52)	rate_imported12 =0.002-0.087 (p36-67)	46.14	0.65	24.86	0.08	6.917E-05
**58**	med_income <18922.890 (p62) &den_public_trans =6.320-77.509 (p33-88)	rate_imported12 =0.004-0.096 (p36-68)	48.85	0.64	24.80	0.17	7.034E-08
**59**	prop_preprim_edu <0.127 (p82) &med_area_home <16.639 (p50) &LU_entropy >0.566 (p52)	rate_imported12 =0.002-0.083 (p36-65)	39.75	0.77	24.79	0.12	6.309E-06
**60**	med_area_home <17.685 (p54) &den_public_trans =5.972-93.660 (p33-92) &POI_den_sports <406.305 (p65)	rate_imported12 =0.003-0.090 (p36-67)	41.04	0.77	24.67	0.13	6.354E-06
**61**	prop_preprim_edu <0.129 (p82) &prop_higher_edu <0.240 (p63) &POI_den_transport =40.112-1204.130 (p17-77)	rate_imported12 =0.004-0.087 (p36-67)	44.23	0.68	24.60	0.14	6.737E-05
**62**	med_area_home <16.096 (p47) &prop_private_resid =0.010-0.219 (p27-84)	rate_imported12 =0.003-0.068 (p36-62)	42.13	0.60	24.36	0.12	3.107E-05
**63**	med_income <19454.231 (p63) &prop_agricultural <0.051 (p80) &LU_entropy >0.546 (p47)	rate_imported12 =0.003-0.083 (p36-65)	41.87	0.70	24.35	0.15	1.903E-06
**64**	med_area_home <15.786 (p46) &POI_den_mall_mkt <106.828 (p63)	rate_imported12 =0.004-0.083 (p36-65)	40.78	0.72	24.29	0.19	7.457E-06
**65**	prop_preprim_edu <0.126 (p81) &prop_higher_edu <0.241 (p63)	rate_imported12 =0.001-0.115 (p36-71)	57.26	0.60	24.21	0.16	4.375E-08
**66**	prop_preprim_edu <0.128 (p82) &med_income <19189.057 (p63)	rate_imported12 =0.004-0.107 (p36-70)	59.05	0.57	24.21	0.12	6.278E-07
**67**	med_area_home <18.572 (p56) &build_area_pp <60.400 (p50) &POI_den_mall_mkt <96.359 (p61)	rate_imported12 =0.003-0.084 (p36-66)	38.31	0.80	24.18	0.18	2.488E-05
**68**	med_area_home <18.617 (p56) &build_area_pp <62.684 (p50) &POI_den_sports <322.303 (p63)	rate_imported12 =0.004-0.085 (p36-66)	38.94	0.78	24.17	0.17	7.825E-05
**69**	med_income <19851.218 (p65) &den_public_trans =7.873-93.077 (p37-92) &POI_den_transport <580.508 (p65)	rate_imported12 =0.004-0.089 (p36-67)	40.01	0.77	24.15	0.12	5.517E-05
**70**	prop_higher_edu <0.264 (p66) &prop_agricultural <0.054 (p81) &POI_den_transport =31.335-969.972 (p14-73)	rate_imported12 =0.003-0.081 (p36-64)	43.19	0.65	24.08	0.15	1.288E-05

**71**	med_area_home <18.116 (p55) &den_public_trans =6.106-93.761 (p33-92) &POI_den_mall_mkt <98.355 (p62)	rate_imported12 =0.003-0.089 (p36-67)	38.81	0.80	24.03	0.17	8.82E-07
**72**	med_area_home <16.075 (p47) &POI_den_telecom_elec <193.066 (p62)	rate_imported12 =0.003-0.087 (p36-66)	41.08	0.72	24.02	0.19	1.003E-05
**73**	med_area_home <18.888 (p57) &build_area_pp <66.954 (p52) &POI_den_telecom_elec <197.000 (p62)	rate_imported12 =0.003-0.090 (p36-67)	40.34	0.76	24.00	0.15	1.564E-05
**74**	med_area_home <18.647 (p56) &build_area_pp <59.831 (p50) &POI_den_transport <472.110 (p62)	rate_imported12 =0.004-0.087 (p36-67)	38.40	0.80	23.99	0.18	3.754E-06
**75**	prop_preprim_edu <0.136 (p85) &prop_higher_edu <0.253 (p65) &POI_den_edu =10.689-412.222 (p15-77)	rate_imported12 =0.003-0.090 (p36-67)	45.60	0.65	23.98	0.13	4.353E-05
**76**	med_area_home <18.394 (p55) &prop_agricultural <0.046 (p79) &POI_den_mall_mkt <104.329 (p62)	rate_imported12 =0.004-0.083 (p36-65)	40.66	0.71	23.96	0.12	1.915E-05
**77**	med_income <19851.218 (p65) &den_public_trans =7.873-93.077 (p37-92) &POI_den_mall_mkt <105.844 (p63)	rate_imported12 =0.004-0.089 (p36-67)	38.86	0.79	23.96	0.12	1.303E-05
**78**	med_area_home <15.681 (p46) &POI_den_sports <348.045 (p64)	rate_imported12 =0.003-0.087 (p36-66)	40.88	0.72	23.92	0.18	4.81E-06
**79**	prop_preprim_edu <0.128 (p82) &prop_higher_edu <0.224 (p58) &den_road >6.792 (p31)	rate_imported12 =0.002-0.097 (p36-68)	44.55	0.69	23.86	0.11	8.142E-05
**80**	med_income <19717.770 (p65) &build_area_pp <64.307 (p51)	rate_imported12 =0.004-0.108 (p36-70)	52.38	0.62	23.86	0.09	8.203E-05
**81**	med_area_home <18.887 (p57) &prop_agricultural <0.049 (p80) &POI_den_telecom_elec <183.375 (p60)	rate_imported12 =0.003-0.079 (p36-64)	39.83	0.71	23.84	0.13	1.632E-05
**82**	med_income <19505.735 (p65) &den_public_trans =6.106-93.761 (p33-92) &POI_den_telecom_elec <212.143 (p64)	rate_imported12 =0.004-0.086 (p36-66)	39.70	0.75	23.76	0.14	7.837E-05
**83**	gender_ratio <95.542 (p67) &den_public_trans =7.873-93.077 (p37-92)	rate_imported12 =0.003-0.133 (p36-73)	57.95	0.63	23.75	0.09	6.066E-05
**84**	med_area_home <17.463 (p52) &POI_den_transport =38.999-486.006 (p17-63)	rate_imported12 =0.003-0.085 (p36-66)	40.04	0.73	23.75	0.23	4.609E-07
**85**	med_income <19735.441 (p65) &prop_private_resid =0.010-0.219 (p27-84)	rate_imported12 =0.002-0.086 (p36-66)	47.35	0.60	23.75	0.17	5.37E-09
**86**	build_area_pp <63.849 (p51) &POI_den_transport =40.112-1204.130 (p17-77)	rate_imported12 =0.002-0.078 (p36-64)	43.44	0.62	23.74	0.14	6.031E-05
**87**	med_area_home <16.121 (p47) &POI_den_edu =10.689-412.222 (p15-77)	rate_imported12 =0.004-0.088 (p36-67)	43.98	0.66	23.70	0.13	5.747E-05
**88**	prop_preprim_edu <0.130 (p83) &med_income <19284.575 (p63) &LU_entropy >0.566 (p52)	rate_imported12 =0.003-0.090 (p36-67)	42.18	0.70	23.61	0.14	2.537E-05
**89**	prop_preprim_edu <0.135 (p84) &med_area_home <16.429 (p49)	rate_imported12 =0.003-0.081 (p36-65)	48.25	0.53	23.57	0.06	7.384E-05
**90**	prop_higher_edu <0.275 (p70) &prop_agricultural <0.048 (p79) &POI_den_edu =10.689-412.222 (p15-77)	rate_imported12 =0.004-0.082 (p36-65)	43.62	0.63	23.42	0.14	7.003E-05

**91**	prop_higher_edu <0.300 (p76) &den_public_trans =7.873-93.077 (p37-92) &POI_den_sports <395.798 (p65)	rate_imported12 =0.004-0.089 (p36-67)	40.25	0.73	23.42	0.09	7.69E-05
**92**	gender_ratio <96.478 (p73) &den_public_trans =6.990-86.032 (p35-91) &POI_den_sports <419.316 (p66)	rate_imported12 =0.003-0.091 (p36-67)	42.18	0.69	23.35	0.08	1.972E-05
**93**	prop_higher_edu <0.239 (p63) &den_population =4861.224-83419.474 (p35-91)	rate_imported12 =0.002-0.093 (p36-68)	44.58	0.65	23.29	0.20	1.344E-08
**94**	prop_higher_edu <0.241 (p63) &prop_agricultural <0.049 (p80) &LU_entropy >0.538 (p44)	rate_imported12 =0.003-0.083 (p36-66)	40.62	0.69	23.28	0.16	3.89E-05
**95**	med_area_home <19.358 (p61) &prop_agricultural <0.045 (p79) &POI_den_transport <488.377 (p63)	rate_imported12 =0.003-0.080 (p36-64)	40.00	0.69	23.27	0.14	9.172E-06
**96**	med_area_home <18.714 (p57) &prop_transport >0.061 (p35) &POI_den_telecom_elec <192.550 (p62)	rate_imported12 =0.002-0.082 (p36-65)	36.97	0.78	23.24	0.17	2.874E-06
**97**	prop_preprim_edu <0.128 (p82) &med_income <20156.073 (p73) &POI_den_edu =10.689-412.222 (p15-77)	rate_imported12 =0.004-0.082 (p36-65)	44.04	0.62	23.21	0.12	6.497E-05
**98**	prop_preprim_edu <0.129 (p82) &build_area_pp <96.254 (p65) &LU_entropy >0.560 (p51)	rate_imported12 =0.002-0.128 (p36-72)	45.24	0.75	23.19	0.09	1.636E-05
**99**	gender_ratio <96.269 (p71) &med_area_home <18.007 (p55) &POI_den_mall_mkt <97.869 (p62)	rate_imported12 =0.002-0.086 (p36-66)	39.84	0.72	23.16	0.08	7.577E-05
**100**	prop_preprim_edu <0.135 (p84) &build_area_pp <67.205 (p52)	rate_imported12 =0.002-0.122 (p36-71)	57.76	0.59	23.07	0.05	6.934E-05
**101**	build_area_pp <59.052 (p48) &LU_entropy >0.559 (p50)	rate_imported12 =0.002-0.077 (p36-64)	38.16	0.70	23.03	0.21	3.026E-05
**102**	med_income <21629.447 (p75) &prop_agricultural <0.048 (p79) &POI_den_edu =10.689-412.222 (p15-77)	rate_imported12 =0.004-0.082 (p36-65)	44.26	0.61	23.03	0.13	4.515E-05
**103**	prop_preprim_edu <0.127 (p82) &med_income <20297.090 (p73) &POI_den_telecom_elec =11.547-222.397 (p10-65)	rate_imported12 =0.004-0.085 (p36-66)	41.70	0.67	22.99	0.16	1.424E-05
**104**	med_area_home <22.842 (p81) &prop_agricultural <0.048 (p79) &POI_den_edu =10.689-412.222 (p15-77)	rate_imported12 =0.004-0.082 (p36-65)	46.34	0.58	22.89	0.10	2.287E-05
**105**	med_income <19402.935 (p63) &prop_agricultural <0.060 (p82) &POI_den_telecom_elec =11.547-254.047 (p10-66)	rate_imported12 =0.003-0.084 (p36-66)	39.89	0.69	22.87	0.17	7.091E-07
**106**	prop_preprim_edu >0.075 (p14) &den_public_trans =4.801-82.617 (p30-90)	rate_imported12 =0.003-0.207 (p36-81)	71.24	0.65	22.87	0.07	8.003E-06
**107**	prop_higher_edu <0.226 (p60) &den_public_trans =6.320-77.509 (p33-88)	rate_imported12 =0.004-0.096 (p36-68)	44.75	0.65	22.86	0.17	1.691E-06
**108**	med_area_home <18.394 (p55) &prop_transport >0.065 (p35) &POI_den_mall_mkt <104.329 (p62)	rate_imported12 =0.004-0.083 (p36-65)	36.97	0.76	22.83	0.17	4.956E-06
**109**	med_income <19695.014 (p65) &prop_agricultural <0.068 (p83) &POI_den_transport =38.999-486.006 (p17-63)	rate_imported12 =0.003-0.083 (p36-65)	38.04	0.73	22.79	0.16	4.363E-07

**110**	gender_ratio <96.478 (p73) &den_public_trans =6.990-86.032 (p35-91) &POI_den_transport <452.694 (p61)	rate_imported12 =0.003-0.091 (p36-67)	38.61	0.75	22.75	0.10	1.753E-06
**111**	build_area_pp <58.474 (p48) &prop_transport >0.070 (p36) &POI_den_telecom_elec <433.771 (p77)	rate_imported12 =0.004-0.083 (p36-66)	38.27	0.72	22.73	0.13	8.185E-05
**112**	med_area_home <18.394 (p55) &prop_transport >0.065 (p35) &POI_den_transport <488.377 (p63)	rate_imported12 =0.003-0.082 (p36-65)	36.36	0.77	22.72	0.18	1.033E-06
**113**	gender_ratio <96.478 (p73) &den_public_trans =6.990-86.032 (p35-91) &POI_den_mall_mkt <148.238 (p67)	rate_imported12 =0.003-0.091 (p36-67)	41.68	0.67	22.60	0.08	4.163E-05
**114**	gender_ratio <95.776 (p69) &med_area_home <19.466 (p61) &POI_den_transport <425.836 (p59)	rate_imported12 =0.002-0.093 (p36-68)	39.90	0.72	22.58	0.13	2.072E-05
**115**	prop_higher_edu <0.263 (p66) &prop_agricultural <0.050 (p80) &POI_den_telecom_elec =11.547-254.047 (p10-66)	rate_imported12 =0.004-0.076 (p36-64)	39.11	0.65	22.50	0.14	3.187E-06
**116**	gender_ratio <95.831 (p69) &prop_preprim_edu <0.129 (p82)	rate_imported12 =0.002-0.196 (p36-80)	77.00	0.61	22.45	0.07	8.695E-06
**117**	gender_ratio <95.202 (p65) &med_area_home <19.225 (p61) &POI_den_telecom_elec <174.743 (p59)	rate_imported12 =0.004-0.087 (p36-67)	38.32	0.73	22.43	0.13	1.565E-05
**118**	med_area_home <16.202 (p48) &LU_entropy >0.566 (p52)	rate_imported12 =0.003-0.090 (p36-67)	41.14	0.68	22.43	0.14	6.549E-05
**119**	den_population =14189.352-82431.823 (p45-90) &LU_entropy >0.550 (p49)	rate_imported12 =0.004-0.086 (p36-66)	40.07	0.68	22.43	0.20	3.733E-07
**120**	prop_preprim_edu <0.127 (p82) &med_income <22078.420 (p77) &den_bldg =0.088-0.389 (p30-82)	rate_imported12 =0.004-0.135 (p36-73)	47.61	0.70	22.40	0.10	3.665E-05
**121**	gender_ratio <96.488 (p74) &prop_transport >0.062 (p35) &POI_den_transport <522.196 (p64)	rate_imported12 =0.003-0.088 (p36-67)	39.96	0.69	22.40	0.10	5.412E-05
**122**	med_income <20092.498 (p73) &prop_transport >0.062 (p35) &POI_den_transport <612.990 (p66)	rate_imported12 =0.003-0.086 (p36-66)	40.08	0.68	22.39	0.12	3.309E-05
**123**	prop_preprim_edu <0.129 (p82) &POI_den_telecom_elec =13.135-361.714 (p13-71)	rate_imported12 =0.004-0.189 (p36-80)	66.25	0.65	22.38	0.10	1.214E-07
**124**	med_income <19621.899 (p65) &prop_business =0.003-0.087 (p44-90) &POI_den_sports <339.929 (p64)	rate_imported12 =0.004-0.090 (p36-67)	35.54	0.83	22.38	0.17	2.17E-05
**125**	den_public_trans =3.582-84.723 (p25-91) &build_area_pp <71.228 (p53)	rate_imported12 =0.004-0.084 (p36-66)	46.06	0.54	22.35	0.10	3.119E-05
**126**	med_income <19621.899 (p65) &prop_gov_insti_faci >0.032 (p43)	rate_imported12 =0.004-0.085 (p36-66)	47.46	0.56	22.27	0.14	3.292E-07
**127**	prop_preprim_edu <0.129 (p82) &prop_gov_insti_faci >0.029 (p40) &POI_den_mall_mkt <141.320 (p66)	rate_imported12 =0.002-0.196 (p36-80)	48.53	0.79	22.27	0.08	2.163E-05
**128**	med_area_home <16.755 (p50)	rate_imported12 =0.002-0.115 (p36-71)	58.83	0.55	22.26	0.21	2.302E-09
**129**	prop_higher_edu <0.233 (p62) &prop_gov_insti_faci >0.035 (p45)	rate_imported12 =0.003-0.095 (p36-68)	43.40	0.65	22.19	0.20	6.686E-08

**130**	prop_preprim_edu <0.130 (p83) &den_bldg >0.081 (p27) &POI_den_telecom_elec <347.789 (p70)	rate_imported12 =0.002-0.215 (p36-81)	57.30	0.72	22.19	0.09	2.869E-05
**131**	prop_preprim_edu <0.134 (p84) &med_income <20130.817 (p73) &POI_den_sports =45.780-437.190 (p24-67)	rate_imported12 =0.002-0.094 (p36-68)	39.30	0.72	22.18	0.14	3.247E-06
**132**	med_income <20323.607 (p73) &prop_transport >0.056 (p33) &POI_den_mall_mkt <116.205 (p64)	rate_imported12 =0.003-0.086 (p36-66)	39.91	0.67	22.18	0.11	2.704E-05
**133**	med_area_home <19.970 (p65) &prop_gov_insti_faci >0.027 (p38) &POI_den_transport <1024.689 (p75)	rate_imported12 =0.003-0.094 (p36-68)	42.77	0.65	22.18	0.11	2.422E-05
**134**	den_public_trans =7.873-93.077 (p37-92) &LU_entropy >0.561 (p51)	rate_imported12 =0.003-0.086 (p36-66)	43.16	0.62	22.16	0.16	2.008E-05
**135**	gender_ratio <96.478 (p73) &prop_transport >0.069 (p36) &POI_den_mall_mkt <117.957 (p64)	rate_imported12 =0.002-0.096 (p36-68)	40.02	0.70	22.07	0.09	8.812E-06
**136**	prop_preprim_edu <0.129 (p82) &prop_transport >0.055 (p33) &POI_den_mall_mkt <141.320 (p66)	rate_imported12 =0.002-0.196 (p36-80)	51.35	0.75	22.07	0.09	1.737E-05
**137**	med_income <19851.218 (p65) &den_population =3043.984-80440.162 (p29-89)	rate_imported12 =0.004-0.089 (p36-67)	48.11	0.56	22.06	0.15	4.796E-07
**138**	gender_ratio <93.751 (p53) &build_area_pp <75.416 (p54)	rate_imported12 =0.004-0.081 (p36-64)	44.33	0.57	22.05	0.12	3.686E-05
**139**	build_area_pp <58.064 (p47) &prop_transport >0.067 (p35) &POI_den_mall_mkt <105.844 (p63)	rate_imported12 =0.003-0.073 (p36-64)	33.53	0.78	22.00	0.18	3.149E-06
**140**	prop_preprim_edu <0.133 (p84) &prop_transport >0.064 (p35) &POI_den_sports <458.042 (p68)	rate_imported12 =0.003-0.198 (p36-80)	51.04	0.76	21.99	0.08	2.397E-06
**141**	prop_preprim_edu <0.127 (p82) &POI_den_transport =40.112-1204.130 (p17-77)	rate_imported12 =0.003-0.232 (p36-82)	71.45	0.66	21.98	0.08	1.494E-05
**142**	build_area_pp <56.179 (p46) &POI_den_edu =10.689-412.222 (p15-77)	rate_imported12 =0.003-0.085 (p36-66)	40.74	0.64	21.95	0.13	7.162E-05
**143**	gender_ratio <96.697 (p74) &prop_agricultural <0.046 (p79) &POI_den_sports <382.888 (p65)	rate_imported12 =0.004-0.098 (p36-68)	46.07	0.61	21.93	0.11	6.806E-05
**144**	prop_higher_edu <0.242 (p63) &prop_agricultural <0.049 (p80)	rate_imported12 =0.003-0.087 (p36-67)	51.40	0.53	21.92	0.12	7.098E-06
**145**	gender_ratio <95.529 (p67) &den_public_trans =7.873-93.077 (p37-92) &POI_den_edu <207.187 (p63)	rate_imported12 =0.004-0.089 (p36-67)	36.48	0.76	21.87	0.11	3.914E-05
**146**	prop_preprim_edu <0.131 (p83) &POI_den_edu =10.689-412.222 (p15-77)	rate_imported12 =0.003-0.240 (p36-82)	72.74	0.66	21.86	0.09	8.288E-07
**147**	prop_higher_edu <0.296 (p75) &prop_transport >0.056 (p33) &POI_den_telecom_elec <246.746 (p65)	rate_imported12 =0.003-0.092 (p36-67)	42.26	0.64	21.85	0.08	7.524E-05
**148**	prop_preprim_edu <0.130 (p83) &den_road >5.415 (p26) &POI_den_mall_mkt <142.262 (p67)	rate_imported12 =0.002-0.128 (p36-72)	49.00	0.65	21.82	0.09	3.286E-05
**149**	med_income <18745.215 (p62) &prop_transport >0.062 (p35)	rate_imported12 =0.003-0.081 (p36-64)	49.31	0.52	21.74	0.11	1.627E-05

**150**	gender_ratio <96.439 (p72) &prop_preprim_edu <0.127 (p82) &LU_entropy >0.562 (p52)	rate_imported12 =0.003-0.075 (p36-64)	39.99	0.60	21.72	0.10	3.028E-05
**151**	gender_ratio <96.056 (p71) &prop_preprim_edu <0.135 (p84) &POI_den_transport <547.167 (p65)	rate_imported12 =0.003-0.105 (p36-69)	46.60	0.62	21.69	0.10	7.642E-05
**152**	med_income <20126.227 (p73) &prop_business =0.003-0.053 (p44-86) &POI_den_telecom_elec <185.721 (p60)	rate_imported12 =0.002-0.093 (p36-67)	34.23	0.85	21.69	0.17	6.68E-06
**153**	gender_ratio <94.869 (p64) &med_income <18909.328 (p62)	rate_imported12 =0.001-0.103 (p36-69)	51.43	0.57	21.69	0.11	4.167E-05
**154**	prop_agricultural <0.047 (p79) &LU_entropy >0.559 (p50)	rate_imported12 =0.002-0.077 (p36-64)	42.24	0.57	21.68	0.16	2.224E-08
**155**	build_area_pp <67.949 (p52) &prop_private_resid =0.013-0.220 (p31-85)	rate_imported12 =0.003-0.094 (p36-68)	40.53	0.67	21.67	0.18	7.887E-06
**156**	med_income <19505.735 (p65) &prop_agricultural <0.051 (p80)	rate_imported12 =0.003-0.084 (p36-66)	53.72	0.49	21.66	0.09	9.089E-06
**157**	build_area_pp <66.552 (p52) &prop_transport >0.060 (p35) &POI_den_transport <428.546 (p59)	rate_imported12 =0.003-0.078 (p36-64)	33.56	0.78	21.43	0.19	3.02E-05
**158**	med_area_home <19.409 (p61) &den_road >6.596 (p30) &POI_den_mall_mkt <105.844 (p63)	rate_imported12 =0.003-0.082 (p36-65)	36.45	0.70	21.37	0.16	4.185E-05
**159**	gender_ratio <95.237 (p65) &prop_gov_insti_faci >0.028 (p38)	rate_imported12 =0.003-0.114 (p36-71)	55.10	0.56	21.32	0.10	7.37E-05
**160**	prop_preprim_edu <0.129 (p82) &prop_higher_edu <0.256 (p65) &POI_den_sports =49.068-381.951 (p25-65)	rate_imported12 =0.001-0.106 (p36-69)	37.36	0.77	21.28	0.17	1.163E-05
**161**	prop_higher_edu <0.296 (p75) &prop_transport >0.063 (p35) &POI_den_mall_mkt <127.884 (p65)	rate_imported12 =0.003-0.092 (p36-67)	40.03	0.66	21.27	0.08	6.289E-05
**162**	med_income <18908.149 (p62) &den_road >5.527 (p27)	rate_imported12 =0.003-0.087 (p36-67)	51.95	0.51	21.25	0.09	2.02E-05
**163**	prop_transport >0.062 (p35) &prop_agricultural <0.049 (p80) &POI_den_transport <439.881 (p59)	rate_imported12 =0.003-0.081 (p36-64)	35.17	0.73	21.24	0.13	2.54E-05
**164**	med_area_home <19.806 (p65) &den_road >6.452 (p30) &POI_den_transport <514.562 (p63)	rate_imported12 =0.003-0.085 (p36-66)	37.24	0.69	21.19	0.15	2.527E-05
**165**	med_area_home <17.765 (p55) &build_area_pp <59.277 (p49) &POI_den_edu <188.430 (p62)	rate_imported12 =0.001-0.101 (p36-68)	36.05	0.78	21.09	0.15	9.647E-06
**166**	med_area_home <18.817 (p57) &prop_business =0.003-0.074 (p43-88)	rate_imported12 =0.003-0.088 (p36-67)	42.34	0.60	21.08	0.14	7.339E-05
**167**	prop_publicResid >0.035 (p69)	rate_imported12 =0.002-0.111 (p36-71)	44.17	0.65	21.07	0.31	4.385E-11
**168**	prop_gov_insti_faci >0.031 (p41) &POI_den_telecom_elec <218.105 (p64)	rate_imported12 =0.003-0.143 (p36-74)	45.15	0.72	21.06	0.21	6.918E-09
**169**	gender_ratio <96.409 (p72) &prop_agricultural <0.049 (p80) &POI_den_telecom_elec <174.011 (p59)	rate_imported12 =0.003-0.080 (p36-64)	39.44	0.61	21.03	0.13	3.177E-06
**170**	den_public_trans =9.794-95.867 (p38-92) &POI_den_edu <249.193 (p65)	rate_imported12 =0.003-0.091 (p36-67)	40.04	0.65	21.01	0.17	2.961E-05

**171**	med_area_home <16.479 (p49) &prop_business =0.003-0.078 (p47-89) &POI_den_transport <476.079 (p62)	rate_imported12 =0.004-0.080 (p36-64)	30.69	0.90	21.01	0.21	9.694E-06
**172**	med_area_home <16.930 (p51) &prop_business =0.003-0.079 (p47-89) &POI_den_telecom_elec <178.907 (p60)	rate_imported12 =0.004-0.082 (p36-65)	30.77	0.92	21.00	0.21	1.661E-06
**173**	med_area_home <16.253 (p49) &prop_business =0.003-0.074 (p43-88) &POI_den_mall_mkt <84.896 (p59)	rate_imported12 =0.004-0.094 (p36-68)	32.05	0.91	20.98	0.19	6.436E-06
**174**	med_income <19621.899 (p65) &den_road >6.596 (p30) &POI_den_sports <382.146 (p65)	rate_imported12 =0.003-0.087 (p36-67)	37.53	0.68	20.95	0.14	8.315E-05
**175**	med_area_home <19.083 (p60) &prop_gov_insti_faci >0.025 (p36) &POI_den_telecom_elec <171.541 (p59)	rate_imported12 =0.004-0.090 (p36-67)	35.38	0.75	20.95	0.14	3.172E-05
**176**	med_area_home <20.145 (p67) &den_road >6.596 (p30) &POI_den_telecom_elec <249.524 (p66)	rate_imported12 =0.003-0.085 (p36-66)	38.56	0.65	20.95	0.12	4.804E-05
**177**	gender_ratio <96.048 (p71) &prop_agricultural <0.047 (p79) &POI_den_edu <188.430 (p62)	rate_imported12 =0.004-0.086 (p36-66)	40.75	0.61	20.93	0.12	3.187E-05
**178**	gender_ratio <95.669 (p69) &prop_private_resid =0.010-0.219 (p27-84)	rate_imported12 =0.003-0.088 (p36-67)	48.45	0.53	20.91	0.10	2.03E-05
**179**	gender_ratio <96.126 (p71) &prop_gov_insti_faci >0.026 (p38) &POI_den_sports <378.094 (p65)	rate_imported12 =0.003-0.087 (p36-67)	38.17	0.67	20.90	0.10	1.1E-05
**180**	med_income <21515.682 (p75) &prop_business =0.002-0.040 (p37-84) &POI_den_transport <549.891 (p65)	rate_imported12 =0.003-0.076 (p36-64)	36.50	0.65	20.85	0.11	6.704E-05
**181**	prop_preprim_edu <0.132 (p84) &den_road >6.792 (p31) &POI_den_sports <374.361 (p64)	rate_imported12 =0.003-0.105 (p36-69)	41.15	0.67	20.83	0.09	9.297E-06
**182**	med_income <19407.964 (p63) &den_road >6.596 (p30) &POI_den_mall_mkt <105.844 (p63)	rate_imported12 =0.003-0.086 (p36-66)	36.32	0.70	20.82	0.14	2.875E-05
**183**	prop_gov_insti_faci >0.034 (p44) &POI_den_sports <400.252 (p65)	rate_imported12 =0.004-0.137 (p36-74)	43.47	0.72	20.82	0.21	1.215E-06
**184**	den_bldg >0.081 (p27) &build_area_pp <83.201 (p58) &POI_den_telecom_elec <181.077 (p60)	rate_imported12 =0.003-0.083 (p36-65)	34.83	0.72	20.78	0.19	3.218E-05
**185**	prop_preprim_edu <0.130 (p83) &den_road >5.415 (p26) &POI_den_transport <528.371 (p64)	rate_imported12 =0.002-0.128 (p36-72)	45.72	0.67	20.76	0.09	1.247E-05
**186**	gender_ratio <96.199 (p71) &den_public_trans =12.043-85.211 (p42-91) &LU_entropy >0.566 (p52)	rate_imported12 =0.004-0.090 (p36-67)	36.99	0.70	20.75	0.07	5.669E-05
**187**	prop_higher_edu <0.254 (p65) &prop_agricultural <0.050 (p80) &POI_den_sports =40.055-361.877 (p22-64)	rate_imported12 =0.003-0.088 (p36-67)	35.56	0.73	20.71	0.14	2.254E-05
**188**	prop_higher_edu <0.212 (p54) &prop_private_resid =0.010-0.219 (p27-84)	rate_imported12 =0.003-0.088 (p36-67)	38.87	0.65	20.67	0.22	2.24E-07
**189**	build_area_pp <68.937 (p52) &prop_gov_insti_faci >0.025 (p36) &POI_den_sports <442.247 (p67)	rate_imported12 =0.003-0.069 (p36-62)	33.15	0.67	20.63	0.15	4.935E-05

**190**	med_income <19505.735 (p65) &prop_agricultural <0.051 (p80) &POI_den_sports =42.524-353.919 (p23-64)	rate_imported12 =0.003-0.084 (p36-66)	34.19	0.74	20.59	0.16	5.386E-06
**191**	gender_ratio <95.443 (p66) &prop_agricultural <0.047 (p79) &POI_den_mall_mkt <106.828 (p63)	rate_imported12 =0.001-0.093 (p36-68)	40.42	0.63	20.53	0.12	6.752E-05
**192**	den_bldg =0.095-0.347 (p32-78) &build_area_pp <60.696 (p50)	rate_imported12 =0.004-0.079 (p36-64)	36.23	0.65	20.51	0.16	6.571E-06
**193**	med_income <19402.085 (p63) &POI_den_transport =40.112-1204.130 (p17-77)	rate_imported12 =0.003-0.095 (p36-68)	46.84	0.56	20.47	0.14	2.209E-05
**194**	med_area_home <15.859 (p46) &prop_business =0.003-0.079 (p47-89) &POI_den_edu <181.634 (p62)	rate_imported12 =0.003-0.087 (p36-67)	30.43	0.92	20.47	0.22	7.045E-07
**195**	gender_ratio <96.199 (p71) &prop_transport >0.063 (p35)	rate_imported12 =0.002-0.087 (p36-67)	54.50	0.48	20.43	0.08	4.537E-05
**196**	gender_ratio <95.227 (p65) &den_population =5790.352-78246.851 (p37-89)	rate_imported12 =0.001-0.109 (p36-70)	49.59	0.57	20.43	0.09	2.924E-05
**197**	med_income <18839.224 (p62) &prop_business =0.004-0.051 (p48-86) &POI_den_mall_mkt <125.093 (p65)	rate_imported12 =0.003-0.091 (p36-67)	31.98	0.85	20.40	0.16	1.54E-05
**198**	prop_business =0.003-0.081 (p47-89) &POI_den_transport =38.999-486.006 (p17-63)	rate_imported12 =0.004-0.142 (p36-74)	37.62	0.83	20.39	0.26	4.407E-08
**199**	med_area_home <19.409 (p61) &den_road >6.596 (p30) &POI_den_sports <316.434 (p63)	rate_imported12 =0.003-0.082 (p36-65)	34.87	0.70	20.38	0.16	3.398E-05
**200**	med_area_home <19.466 (p61) &den_bldg >0.098 (p33) &POI_den_transport <425.836 (p59)	rate_imported12 =0.003-0.091 (p36-67)	33.41	0.79	20.37	0.20	3.18E-07
**201**	prop_preprim_edu <0.135 (p84) &build_area_pp <67.205 (p52) &POI_den_telecom_elec <177.637 (p60)	rate_imported12 =0.002-0.090 (p36-67)	37.18	0.68	20.36	0.09	6.96E-05
**202**	build_area_pp <70.628 (p53) &prop_gov_insti_faci >0.025 (p36) &POI_den_telecom_elec <171.541 (p59)	rate_imported12 =0.004-0.084 (p36-66)	32.62	0.79	20.33	0.19	5.068E-06
**203**	gender_ratio <96.153 (p71) &prop_business =0.003-0.068 (p47-87) &POI_den_telecom_elec <177.345 (p60)	rate_imported12 =0.003-0.091 (p36-67)	32.05	0.84	20.31	0.11	3.139E-05
**204**	prop_higher_edu <0.224 (p58) &den_road >6.792 (p31)	rate_imported12 =0.003-0.084 (p36-66)	43.52	0.55	20.30	0.16	1.002E-06
**205**	gender_ratio <95.505 (p67) &prop_gov_insti_faci >0.025 (p36) &POI_den_telecom_elec <171.541 (p59)	rate_imported12 =0.004-0.096 (p36-68)	34.56	0.76	20.27	0.14	8.242E-07
**206**	med_area_home <16.479 (p49) &prop_business =0.003-0.078 (p47-89) &POI_den_sports <305.784 (p62)	rate_imported12 =0.003-0.084 (p36-66)	29.86	0.92	20.25	0.22	2.511E-05
**207**	prop_higher_edu <0.233 (p62) &den_bldg >0.100 (p33)	rate_imported12 =0.003-0.084 (p36-66)	44.77	0.54	20.20	0.14	2.839E-07
**208**	prop_gov_insti_faci >0.034 (p44) &POI_den_mall_mkt <106.828 (p63)	rate_imported12 =0.003-0.124 (p36-71)	39.85	0.73	20.18	0.23	6.265E-06
**209**	med_area_home <21.600 (p75) &prop_transport >0.070 (p36)	rate_imported12 =0.004-0.083 (p36-66)	53.52	0.47	20.15	0.08	2.943E-05
**210**	med_area_home <21.183 (p74) &prop_private_resid =0.013-0.220 (p31-85) &POI_den_sports =51.511-381.357 (p26-65)	rate_imported12 =0.002-0.080 (p36-64)	33.55	0.71	20.14	0.11	2.933E-05

**211**	prop_business =0.004-0.078 (p50-89) &POI_den_mall_mkt <104.329 (p62)	rate_imported12 =0.001-0.126 (p36-72)	35.84	0.82	20.11	0.27	4.734E-08
**212**	prop_business =0.003-0.071 (p46-88) &POI_den_sports <367.628 (p64)	rate_imported12 =0.002-0.128 (p36-72)	39.61	0.74	20.11	0.19	2.628E-06
**213**	prop_higher_edu <0.261 (p66) &prop_business =0.003-0.054 (p47-86) &POI_den_telecom_elec <217.233 (p64)	rate_imported12 =0.003-0.094 (p36-68)	32.75	0.81	20.08	0.13	5.058E-05
**214**	den_population =13525.712-86590.585 (p45-91) &POI_den_edu <188.430 (p62)	rate_imported12 =0.003-0.080 (p36-64)	32.51	0.74	19.91	0.28	9.322E-07
**215**	den_bldg >0.081 (p27) &build_area_pp <61.518 (p50) &POI_den_mall_mkt <86.482 (p59)	rate_imported12 =0.003-0.074 (p36-64)	31.08	0.75	19.87	0.16	1.092E-05
**216**	prop_higher_edu <0.240 (p63) &POI_den_transport =40.112-1204.130 (p17-77)	rate_imported12 =0.003-0.085 (p36-66)	44.94	0.53	19.85	0.13	9.678E-06
**217**	build_area_pp <64.307 (p51)	rate_imported12 =0.002-0.119 (p36-71)	56.57	0.54	19.83	0.19	1.392E-08
**218**	prop_higher_edu <0.261 (p66) &prop_business =0.003-0.054 (p47-86)	rate_imported12 =0.003-0.094 (p36-68)	37.45	0.66	19.82	0.18	5.691E-07
**219**	prop_preprim_edu >0.080 (p19) &den_population =5790.352-78246.851 (p37-89)	rate_imported12 =0.001-0.119 (p36-71)	49.94	0.58	19.81	0.09	6.704E-05
**220**	gender_ratio <96.056 (p71) &den_bldg >0.103 (p34) &POI_den_transport <547.167 (p65)	rate_imported12 =0.003-0.089 (p36-67)	36.56	0.67	19.81	0.10	2.45E-05
**221**	build_area_pp <68.937 (p52) &prop_gov_insti_faci >0.025 (p36) &POI_den_transport <418.284 (p58)	rate_imported12 =0.003-0.085 (p36-66)	31.58	0.78	19.77	0.19	1.72E-05
**222**	med_income <21376.057 (p75) &prop_private_resid =0.022-0.228 (p36-85) &POI_den_sports =39.533-357.737 (p22-64)	rate_imported12 =0.003-0.083 (p36-65)	31.45	0.79	19.72	0.19	8.888E-06
**223**	prop_higher_edu <0.214 (p55) &prop_transport >0.092 (p39)	rate_imported12 =0.002-0.085 (p36-66)	39.09	0.60	19.54	0.19	2.569E-06
**224**	prop_agricultural <0.054 (p81) &POI_den_transport =31.335-969.972 (p14-73)	rate_imported12 =0.003-0.081 (p36-64)	48.00	0.49	19.51	0.09	2.67E-05
**225**	den_road >5.514 (p27) &build_area_pp <78.671 (p56) &POI_den_transport <396.222 (p58)	rate_imported12 =0.004-0.087 (p36-67)	33.89	0.71	19.51	0.16	8.177E-05
**226**	prop_agricultural <0.050 (p80) &POI_den_telecom_elec =11.547-254.047 (p10-66)	rate_imported12 =0.003-0.075 (p36-64)	42.10	0.51	19.51	0.13	6.384E-07
**227**	med_income <18909.328 (p62) &den_bldg =0.087-0.362 (p29-80)	rate_imported12 =0.003-0.078 (p36-64)	39.33	0.56	19.43	0.15	1.504E-06
**228**	prop_gov_insti_faci >0.028 (p39) &LU_entropy >0.552 (p49)	rate_imported12 =0.002-0.098 (p36-68)	43.06	0.58	19.35	0.14	9.098E-06
**229**	prop_gov_insti_faci >0.028 (p39) &POI_den_transport <493.197 (p63)	rate_imported12 =0.002-0.092 (p36-67)	38.51	0.62	19.29	0.19	1.441E-07
**230**	gender_ratio <96.478 (p73) &POI_den_transport =50.731-764.399 (p19-69)	rate_imported12 =0.003-0.091 (p36-67)	45.19	0.54	19.26	0.10	1.058E-05
**231**	med_area_home <18.890 (p57) &den_bldg >0.112 (p36) &POI_den_edu <188.430 (p62)	rate_imported12 =0.003-0.090 (p36-67)	31.96	0.77	19.12	0.18	4.063E-06

**232**	prop_business =0.003-0.068 (p47-87) &prop_gov_insti_faci >0.033 (p43) &POI_den_telecom_elec <177.345 (p60)	rate_imported12 =0.003-0.122 (p36-71)	31.54	0.90	19.03	0.11	9.176E-07
**233**	prop_gov_insti_faci >0.027 (p38) &prop_transport >0.060 (p35) &POI_den_transport <428.546 (p59)	rate_imported12 =0.003-0.078 (p36-64)	30.86	0.73	18.95	0.15	3.02E-05
**234**	prop_preprim_edu <0.133 (p84) &prop_higher_edu <0.246 (p64) &POI_den_mall_mkt =23.559-142.109 (p36-67)	rate_imported12 =0.003-0.091 (p36-67)	31.50	0.77	18.94	0.11	1.067E-05
**235**	den_bldg >0.112 (p36) &prop_gov_insti_faci >0.026 (p38) &POI_den_telecom_elec <183.001 (p60)	rate_imported12 =0.002-0.116 (p36-71)	33.15	0.81	18.90	0.15	3.377E-05
**236**	prop_business =0.004-0.078 (p50-89) &POI_den_telecom_elec <174.335 (p59)	rate_imported12 =0.003-0.115 (p36-71)	32.17	0.83	18.84	0.31	2.013E-09
**237**	med_income <19309.602 (p63) &prop_business =0.003-0.054 (p48-86)	rate_imported12 =0.002-0.102 (p36-69)	36.81	0.67	18.83	0.17	3.529E-06
**238**	prop_agricultural <0.050 (p80) &POI_den_sports =40.055-361.877 (p22-64)	rate_imported12 =0.003-0.088 (p36-67)	39.09	0.59	18.83	0.12	7.629E-05
**239**	prop_transport >0.091 (p39) &LU_entropy >0.551 (p49)	rate_imported12 =0.003-0.087 (p36-67)	39.41	0.57	18.72	0.15	1.998E-05
**240**	prop_preprim_edu <0.133 (p84) &POI_den_sports =40.055-361.877 (p22-64)	rate_imported12 =0.002-0.104 (p36-69)	43.01	0.58	18.68	0.09	1.744E-05
**241**	den_road >5.679 (p27) &prop_business =0.003-0.081 (p47-89) &POI_den_transport <383.279 (p57)	rate_imported12 =0.003-0.122 (p36-71)	31.17	0.88	18.62	0.08	1.959E-05
**242**	prop_transport >0.103 (p43) &POI_den_telecom_elec <177.715 (p60)	rate_imported12 =0.003-0.080 (p36-64)	30.39	0.74	18.62	0.34	4.031E-08
**243**	prop_higher_edu <0.254 (p65) &prop_private_resid =0.022-0.228 (p36-85) &POI_den_sports =40.868-344.556 (p22-64)	rate_imported12 =0.004-0.072 (p36-64)	28.07	0.78	18.56	0.20	1.991E-05
**244**	prop_private_resid =0.004-0.226 (p21-85) &prop_business =0.003-0.054 (p48-86)	rate_imported12 =0.003-0.254 (p36-83)	45.22	0.79	18.51	0.13	6.233E-06
**245**	med_income <20156.073 (p73) &POI_den_edu =10.689-412.222 (p15-77)	rate_imported12 =0.003-0.086 (p36-66)	45.95	0.50	18.51	0.11	5.135E-05
**246**	med_area_home <22.030 (p78) &den_road >6.792 (p31)	rate_imported12 =0.004-0.079 (p36-64)	50.80	0.45	18.42	0.07	3.478E-05
**247**	den_public_trans =13.940-70.916 (p45-86)	rate_imported12 =0.003-0.146 (p36-74)	52.30	0.59	18.28	0.21	4.451E-08
**248**	med_income <19381.772 (p63) &prop_private_resid =0.022-0.228 (p36-85) &POI_den_transport =58.916-397.463 (p22-58)	rate_imported12 =0.003-0.080 (p36-64)	27.10	0.88	18.28	0.25	3.732E-05
**249**	prop_preprim_edu >0.083 (p22) &prop_transport >0.092 (p39)	rate_imported12 =0.002-0.090 (p36-67)	46.77	0.50	18.27	0.09	4.43E-05
**250**	gender_ratio <95.227 (p65) &den_population =5790.352-78246.851 (p37-89) &POI_den_transport <380.617 (p57)	rate_imported12 =0.004-0.095 (p36-68)	30.24	0.79	18.25	0.08	3.139E-05
**251**	prop_higher_edu <0.275 (p70) &POI_den_edu =10.689-412.222 (p15-77)	rate_imported12 =0.004-0.082 (p36-65)	45.35	0.49	18.24	0.10	6.591E-05
**252**	den_bldg >0.113 (p37) &POI_den_sports <403.902 (p65)	rate_imported12 =0.003-0.128 (p36-72)	41.82	0.64	18.21	0.20	6.083E-06
**253**	build_area_pp <75.510 (p55) &prop_gov_insti_faci >0.025 (p36)	rate_imported12 =0.002-0.068 (p36-62)	38.13	0.44	18.18	0.08	3.424E-05

**254**	den_road >6.274 (p30) &LU_entropy >0.566 (p52)	rate_imported12 =0.004-0.085 (p36-66)	41.06	0.53	18.14	0.10	1.783E-05
**255**	prop_business =0.004-0.084 (p49-90) &POI_den_edu =7.872-186.946 (p12-62)	rate_imported12 =0.002-0.111 (p36-71)	32.31	0.77	18.13	0.26	1.147E-07
**256**	prop_business =0.003-0.070 (p44-88)	rate_imported12 =0.002-0.207 (p36-81)	59.26	0.64	18.10	0.19	3.968E-08
**257**	den_bldg >0.112 (p36) &build_area_pp <60.487 (p50) &POI_den_edu <188.430 (p62)	rate_imported12 =0.003-0.090 (p36-67)	29.02	0.81	18.06	0.20	1.663E-05
**258**	prop_transport >0.094 (p40) &POI_den_edu <188.430 (p62)	rate_imported12 =0.005-0.083 (p36-65)	32.44	0.66	18.01	0.25	4.368E-06
**259**	den_public_trans =11.541-64.163 (p40-85) &prop_gov_insti_faci >0.028 (p39) &POI_den_transport <421.770 (p58)	rate_imported12 =0.004-0.086 (p36-66)	28.36	0.80	17.81	0.08	5.223E-05
**260**	den_road >6.792 (p31) &POI_den_transport <668.661 (p66)	rate_imported12 =0.003-0.099 (p36-68)	41.89	0.56	17.68	0.16	1.87E-05
**261**	den_road >8.553 (p41) &POI_den_sports <416.114 (p66)	rate_imported12 =0.004-0.109 (p36-70)	36.66	0.64	17.30	0.22	5.737E-06
**262**	prop_transport >0.128 (p52) &POI_den_mall_mkt <106.881 (p63)	rate_imported12 =0.004-0.087 (p36-67)	27.89	0.79	17.29	0.37	7.363E-09
**263**	gender_ratio <93.726 (p53)	rate_imported12 =0.003-0.105 (p36-69)	55.16	0.48	17.25	0.15	3.542E-07
**264**	prop_preprim_edu >0.090 (p35) &prop_gov_insti_faci >0.030 (p40)	rate_imported12 =0.003-0.090 (p36-67)	40.61	0.53	17.24	0.11	3.875E-05
**265**	den_bldg >0.124 (p39) &POI_den_mall_mkt <162.791 (p69)	rate_imported12 =0.002-0.087 (p36-67)	36.44	0.57	17.11	0.18	8.978E-06
**266**	prop_transport >0.119 (p50) &POI_den_transport <436.867 (p59)	rate_imported12 =0.003-0.078 (p36-64)	26.61	0.78	16.99	0.38	3.043E-08
**267**	den_bldg >0.124 (p39) &POI_den_telecom_elec <177.715 (p60)	rate_imported12 =0.003-0.087 (p36-67)	31.30	0.65	16.92	0.27	4.593E-08
**268**	gender_ratio <93.642 (p53) &den_bldg >0.124 (p39) &POI_den_telecom_elec <177.715 (p60)	rate_imported12 =0.003-0.084 (p36-66)	25.93	0.84	16.82	0.19	5.855E-06
**269**	prop_private_resid =0.004-0.226 (p21-85)	rate_imported12 =0.003-0.254 (p36-83)	80.21	0.59	16.74	0.12	1.506E-07
**270**	den_road >7.146 (p34) &POI_den_edu <209.057 (p63)	rate_imported12 =0.002-0.088 (p36-67)	36.21	0.56	16.60	0.17	3.762E-05
**271**	prop_preprim_edu >0.083 (p23) &den_road >8.068 (p38)	rate_imported12 =0.004-0.084 (p36-66)	44.71	0.47	16.59	0.09	3.893E-05
**272**	den_bldg >0.112 (p36) &POI_den_transport <421.770 (p58)	rate_imported12 =0.003-0.088 (p36-67)	32.22	0.63	16.58	0.24	4.606E-07
**273**	den_population =13178.148-61556.392 (p44-81) &POI_den_mall_mkt <99.356 (p62)	rate_imported12 =0.003-0.078 (p36-64)	26.81	0.73	16.55	0.29	4.86E-06
**274**	gender_ratio <96.439 (p72) &POI_den_edu =29.847-182.989 (p27-62)	rate_imported12 =0.003-0.075 (p36-64)	32.18	0.56	16.54	0.12	3.834E-06
**275**	prop_gov_insti_faci >0.031 (p41)	rate_imported12 =0.003-0.124 (p36-71)	61.92	0.49	16.39	0.13	3.895E-08
**276**	den_population >28322.843 (p61) &prop_private_resid =0.022-0.228 (p36-85)	rate_imported12 =0.004-0.090 (p36-67)	30.08	0.64	16.35	0.17	2.969E-05
**277**	prop_private_resid =0.022-0.228 (p36-85) &POI_den_sports =39.533-357.737 (p22-64)	rate_imported12 =0.005-0.062 (p36-59)	28.64	0.53	16.35	0.16	2.555E-05
**278**	prop_transport >0.131 (p52) &POI_den_sports <320.746 (p63)	rate_imported12 =0.003-0.091 (p36-67)	26.52	0.80	16.33	0.38	7.363E-09
**279**	den_road >9.124 (p43) &POI_den_mall_mkt <105.844 (p63)	rate_imported12 =0.002-0.105 (p36-69)	31.22	0.69	16.14	0.27	3.047E-07
**280**	prop_preprim_edu <0.126 (p81) &med_income <15470.453 (p39) &POI_den_mall_mkt <95.198 (p60)	rate_imported12 =0.004-0.098 (p36-68)	26.29	0.83	16.12	0.24	1.369E-05

**281**	prop_private_resid =0.022-0.228 (p36-85) &POI_den_transport =58.916-397.463 (p22-58)	rate_imported12 =0.004-0.069 (p36-62)	27.66	0.60	15.93	0.18	1.259E-05
**282**	prop_private_resid =0.022-0.228 (p36-85) &POI_den_telecom_elec =35.039-212.421 (p26-64)	rate_imported12 =0.004-0.068 (p36-62)	27.93	0.58	15.81	0.16	6.417E-05
**283**	prop_preprim_edu <0.134 (p84) &den_population >28322.843 (p61) &POI_den_transport <421.427 (p58)	rate_imported12 =0.004-0.079 (p36-64)	22.22	0.90	15.22	0.03	9.634E-07
**284**	den_population =18750.995-80373.241 (p50-89)	rate_imported12 =0.004-0.082 (p36-65)	37.27	0.45	15.07	0.18	8.436E-09
**285**	den_road >9.364 (p44) &POI_den_telecom_elec <177.715 (p60)	rate_imported12 =0.003-0.080 (p36-64)	26.15	0.67	15.06	0.31	2.537E-08
**286**	den_population =10614.285-60830.356 (p42-81) &POI_den_transport <374.683 (p57)	rate_imported12 =0.003-0.068 (p36-62)	23.56	0.69	15.03	0.30	7.268E-06
**287**	POI_den_sports =51.286-360.682 (p26-64)	rate_imported12 =0.004-0.111 (p36-71)	42.19	0.52	14.68	0.18	8.623E-07
**288**	POI_den_edu =10.689-412.222 (p15-77)	rate_imported12 =0.003-0.240 (p36-82)	74.78	0.57	14.67	0.11	3.956E-06
**289**	POI_den_transport =103.949-504.827 (p30-63)	rate_imported12 =0.003-0.095 (p36-68)	35.96	0.52	14.24	0.21	6.721E-06
**290**	prop_transport >0.087 (p38)	rate_imported12 =0.003-0.078 (p36-64)	50.15	0.39	14.13	0.11	1.68E-06
**291**	den_bldg >0.146 (p43) &LU_entropy >0.570 (p53)	rate_imported12 =0.002-0.128 (p36-72)	33.29	0.63	14.11	0.14	5.819E-05
**292**	den_population =13475.738-56328.069 (p45-80) &POI_den_sports <280.418 (p60)	rate_imported12 =0.003-0.067 (p36-62)	22.28	0.67	14.08	0.27	2.725E-05
**293**	build_area_pp <69.988 (p53) &prop_business =0.005-0.066 (p53-87) &POI_den_mall_mkt <123.207 (p65)	rate_imported12 =0.003-0.068 (p36-62)	20.55	0.79	14.05	0.17	3.986E-05
**294**	den_bldg =0.096-0.297 (p32-73)	rate_imported12 =0.004-0.143 (p36-74)	44.53	0.55	13.78	0.17	2.574E-05
**295**	den_public_trans =12.345-46.981 (p42-78) &POI_den_mall_mkt <103.311 (p62)	rate_imported12 =0.003-0.086 (p36-66)	24.92	0.67	13.77	0.20	5.285E-05
**296**	LU_entropy >0.561 (p51)	rate_imported12 =0.002-0.096 (p36-68)	45.97	0.44	13.74	0.13	6.214E-05
**297**	POI_den_telecom_elec =73.296-174.511 (p41-59)	rate_imported12 =0.003-0.081 (p36-64)	25.71	0.61	13.54	0.32	5.162E-08
**298**	den_road >6.792 (p31)	rate_imported12 =0.004-0.079 (p36-64)	52.66	0.36	11.52	0.08	8.228E-07
**299**	den_population =8304.569-47145.016 (p39-74) &POI_den_telecom_elec <206.748 (p64)	rate_imported12 =0.004-0.094 (p36-68)	22.11	0.62	11.31	0.20	6.699E-07
**300**	prop_grassland <0.023 (p50) &POI_den_telecom_elec <174.335 (p59)	rate_imported12 =0.003-0.079 (p36-64)	19.72	0.65	11.03	0.30	2.625E-05
**301**	den_public_trans =14.057-39.887 (p46-73) &POI_den_telecom_elec <174.335 (p59)	rate_imported12 =0.002-0.075 (p36-64)	18.14	0.69	10.92	0.26	6.422E-06
**302**	den_public_trans =18.568-45.750 (p53-78) &POI_den_transport <410.387 (p58)	rate_imported12 =0.006-0.058 (p36-57)	14.59	0.68	10.14	0.29	9.544E-06
**303**	prop_shrubland <0.023 (p37) &POI_den_telecom_elec <213.848 (p64)	rate_imported12 =0.003-0.097 (p36-68)	16.15	0.78	9.59	0.41	8.826E-06
**304**	prop_shrubland <0.023 (p37) &POI_den_transport <520.651 (p64)	rate_imported12 =0.004-0.093 (p36-68)	15.65	0.76	9.28	0.40	2.128E-05
**305**	prop_agricultural <0.063 (p82)	rate_imported12 =0.003-0.082 (p36-65)	60.20	0.34	8.85	0.05	7.916E-05

**306**	POI_den_edu =104.122-568.992 (p51-80) &POI_den_telecom_elec <167.092 (p58)	rate_imported12 =0.004-0.090 (p36-67)	13.52	0.85	8.63	0.43	1.491E-05
**307**	den_population >47145.016 (p74) &POI_den_telecom_elec <206.748 (p64)	rate_imported12 =0.004-0.094 (p36-68)	12.86	0.81	8.10	0.35	7.595E-05
**308**	den_public_trans >39.887 (p73) &POI_den_telecom_elec <174.335 (p59)	rate_imported12 =0.002-0.075 (p36-64)	10.94	0.87	7.50	0.50	2.137E-05
**309**	prop_higher_edu >0.261 (p66) &prop_business =0.003-0.054 (p47-86)	rate_imported12 =0.094-0.211 (p68-81)	10.86	0.40	7.24	0.19	6.52E-05
**310**	den_population >56328.069 (p80) &POI_den_sports <280.418 (p60)	rate_imported12 =0.003-0.067 (p36-62)	8.77	0.96	6.51	0.55	7.007E-05
**311**	den_population >61556.392 (p81) &POI_den_mall_mkt <99.356 (p62)	rate_imported12 =0.003-0.078 (p36-64)	8.88	0.97	6.30	0.55	5.878E-06
**312**	den_public_trans >46.981 (p78) &POI_den_mall_mkt <103.311 (p62)	rate_imported12 =0.003-0.086 (p36-66)	9.56	0.86	6.25	0.49	8.109E-05
**313**	den_population >60830.356 (p81) &POI_den_transport <374.683 (p57)	rate_imported12 =0.003-0.068 (p36-62)	7.84	1.00	5.87	0.60	5.465E-05
**314**	med_income >19309.602 (p63) &prop_business =0.003-0.054 (p48-86)	rate_imported12 =0.102-0.213 (p69-81)	8.34	0.31	5.14	0.13	6.018E-05
**315**	den_public_trans >45.750 (p78) &POI_den_transport <410.387 (p58)	rate_imported12 =0.006-0.058 (p36-57)	6.41	0.80	4.74	0.52	2.175E-05
**316**	den_public_trans >37.524 (p70) &POI_den_sports <121.205 (p43)	rate_imported12 =0.006-0.029 (p36-46)	5.00	1.00	4.53	0.84	6.238E-08
**317**	POI_den_sports =360.682-802.233 (p64-78)	rate_imported12 =0.111-0.216 (p71-81)	7.04	0.24	3.69	0.13	7.558E-05
**318**	prop_grassland <0.010 (p35) &POI_den_transport <201.029 (p45)	rate_imported12 =0.002-0.033 (p36-47)	3.11	0.69	2.59	0.56	7.166E-07
**319**	med_income <19043.072 (p63)	rate_imported12 <0.109 (p70)	116.90	0.86	22.90	0.17	1.01E-14
**320**	prop_higher_edu <0.239 (p63) &prop_publicResid <0.005 (p56)	rate_imported12 <0.002 (p36)	40.34	0.70	19.56	0.25	3.634E-07
**321**	med_income <19735.441 (p65) &prop_private_resid <0.010 (p27)	rate_imported12 <0.002 (p36)	34.21	0.75	17.73	0.10	8.104E-05
**322**	prop_preprim_edu >0.080 (p19) &ave_household_size <3.213 (p87)	rate_imported12 <0.116 (p71)	124.99	0.82	17.48	0.05	4.987E-05
**323**	prop_higher_edu <0.212 (p54) &prop_private_resid <0.010 (p27)	rate_imported12 <0.003 (p36)	32.90	0.76	17.28	0.11	8.104E-05
**324**	prop_higher_edu <0.233 (p62) &prop_gov_insti_faci <0.035 (p45)	rate_imported12 <0.003 (p36)	39.85	0.63	17.18	0.14	1.972E-05
**325**	prop_higher_edu <0.239 (p63) &den_population <4861.224 (p35)	rate_imported12 <0.002 (p36)	34.62	0.71	17.08	0.18	2.684E-05
**326**	prop_preprim_edu >0.090 (p35) &prop_gov_insti_faci <0.030 (p40)	rate_imported12 <0.003 (p36)	39.12	0.63	16.86	0.12	7.46E-05
**327**	med_income <19851.218 (p65) &den_population <3043.984 (p29)	rate_imported12 <0.004 (p36)	30.14	0.80	16.51	0.21	3.585E-06
**328**	prop_preprim_edu >0.124 (p80)	rate_imported12 <0.003 (p36)	31.02	0.74	15.92	0.38	1.215E-08
**329**	prop_private_resid <0.004 (p21)	rate_imported12 <0.003 (p36)	32.00	0.71	15.85	0.35	3.29E-08
**330**	med_income <19621.899 (p65) &prop_gov_insti_faci <0.032 (p43)	rate_imported12 <0.004 (p36)	36.31	0.64	15.81	0.14	1.271E-05
**331**	prop_higher_edu <0.214 (p55) &prop_transport <0.092 (p39)	rate_imported12 <0.002 (p36)	33.95	0.66	15.49	0.19	3.837E-05
**332**	prop_higher_edu <0.226 (p60) &den_public_trans <6.320 (p33)	rate_imported12 <0.004 (p36)	31.58	0.70	15.30	0.18	2.42E-05
**333**	prop_higher_edu <0.213 (p54) &build_area_pp >62.720 (p50)	rate_imported12 <0.001 (p36)	32.36	0.67	15.08	0.20	9.105E-06
**334**	prop_business <0.003 (p44)	rate_imported12 <0.002 (p36)	48.55	0.52	14.79	0.16	1.326E-05
**335**	prop_higher_edu =0.128-0.263 (p17-66)	rate_imported12 <0.101 (p68)	87.37	0.81	14.72	0.14	2.114E-05
**336**	med_income <18922.890 (p62) &den_public_trans <6.320 (p33)	rate_imported12 <0.004 (p36)	28.11	0.73	14.32	0.22	3.382E-05
**337**	prop_higher_edu <0.224 (p58) &den_road <6.792 (p31)	rate_imported12 <0.003 (p36)	30.15	0.66	13.71	0.15	5.911E-05
**338**	den_public_trans <3.226 (p23)	rate_imported12 <0.003 (p36)	31.53	0.63	13.43	0.27	2.411E-06

**339**	build_area_pp >69.910 (p53) &prop_agricultural >0.068 (p83)	rate_imported12 <0.003 (p36)	23.66	0.82	13.32	0.14	8.83E-06
**340**	med_income <18745.215 (p62) &prop_transport <0.062 (p35)	rate_imported12 <0.003 (p36)	26.66	0.72	13.27	0.21	7.551E-05
**341**	build_area_pp >68.387 (p52) &prop_rural_set >0.024 (p65)	rate_imported12 <0.003 (p36)	27.92	0.67	13.02	0.21	4.364E-07
**342**	med_income <18909.328 (p62) &den_bldg <0.087 (p29)	rate_imported12 <0.003 (p36)	24.80	0.76	12.99	0.24	3.227E-05
**343**	prop_higher_edu <0.233 (p62) &den_bldg <0.100 (p33)	rate_imported12 <0.003 (p36)	29.77	0.64	12.91	0.15	4.727E-05
**344**	prop_gov_insti_faci <0.031 (p41)	rate_imported12 <0.003 (p36)	44.26	0.51	12.82	0.15	7.142E-05
**345**	med_income <18908.149 (p62) &den_road <5.527 (p27)	rate_imported12 <0.003 (p36)	23.52	0.74	12.11	0.20	4.679E-05
**346**	ave_household_size <3.251 (p87)	rate_imported12 <0.109 (p70)	141.61	0.76	12.11	0.06	6.175E-09
**347**	den_road <11.013 (p51) &build_area_pp >69.326 (p52)	rate_imported12 <0.003 (p36)	33.41	0.56	12.06	0.13	1.485E-05
**348**	prop_agricultural >0.063 (p82)	rate_imported12 <0.003 (p36)	25.53	0.68	11.96	0.32	4.34E-06
**349**	prop_industrial >0.011 (p73)	rate_imported12 <0.094 (p68)	50.94	0.86	11.87	0.20	2.928E-05
**350**	build_area_pp >66.697 (p52) &POI_den_mall_mkt <61.230 (p54) (not written in paper, same as asso betweeb build_area_pp and imp rate)	rate_imported12 <0.003 (p36)	33.82	0.55	11.65	0.15	5.53E-05
**351**	med_income <18250.903 (p61) &med_area_home >16.121 (p47)	rate_imported12 <0.004 (p36)	25.01	0.66	11.34	0.22	3.282E-05
**352**	den_population <845.965 (p14)	rate_imported12 <0.004 (p36)	22.46	0.70	10.97	0.34	1.138E-05
**353**	build_area_pp >69.988 (p53) &POI_den_sports <258.226 (p58) (not written in paper, same as asso betweeb build_area_pp and imp rate)	rate_imported12 <0.002 (p36)	32.91	0.54	10.89	0.14	6.918E-05
**354**	prop_publicResid <0.010 (p60) &prop_industrial >0.010 (p71)	rate_imported12 <0.001 (p36)	22.60	0.69	10.75	0.25	4.576E-05
**355**	prop_over65 >0.211 (p89)	rate_imported12 <0.003 (p36)	19.08	0.74	9.85	0.38	2.904E-07
**356**	prop_higher_edu <0.128 (p17)	rate_imported12 <0.101 (p68)	34.11	0.94	9.57	0.26	2.367E-05
**357**	prop_open_recreation <0.002 (p11)	rate_imported12 <0.001 (p36)	17.71	0.74	9.05	0.38	2.37E-05
**358**	prop_rural_set >0.069 (p80)	rate_imported12 <0.004 (p36)	24.92	0.53	7.90	0.17	4.173E-05

(h) Rules for wave-2 local case rate; POI density was used

No.	Antecedent	Consequent	Supp	Conf	Lev	Imp	*P*
**1**	med_income <24194.445 (p79) &prop_transport >0.091 (p39)	rate_local3 >0.362 (p56)	64.751	0.6623	21.946	0.1318	4.207E-06
**2**	med_income <24895.467 (p79) &den_road >8.294 (p39)	rate_local3 >0.352 (p56)	65.657	0.6666	21.657	0.1402	4.314E-06
**3**	med_income <24593.452 (p79) &prop_shrubland <0.112 (p58)	rate_local3 >0.378 (p57)	62.268	0.6421	21.384	0.1206	1.366E-05
**4**	med_area_home <20.213 (p67) &prop_shrubland <0.112 (p58)	rate_local3 >0.378 (p57)	59.254	0.6548	21.102	0.1217	5.976E-05
**5**	med_area_home <19.021 (p58) &prop_transport >0.091 (p39)	rate_local3 >0.362 (p56)	61.609	0.6619	20.86	0.1001	1.236E-05
**6**	med_income <17558.932 (p56) &den_population >15540.769 (p47)	rate_local3 >0.327 (p52)	56.807	0.7467	20.769	0.13	2.117E-06
**7**	med_income <25034.670 (p81) &den_bldg >0.115 (p37)	rate_local3 >0.370 (p57)	65.332	0.6254	20.369	0.1203	6.365E-06
**8**	prop_preprim_edu <0.128 (p82) &prop_higher_edu <0.223 (p58) &med_area_home <19.043 (p59)	rate_local3 >0.279 (p48)	57.502	0.8151	20.31	0.0944	6.116E-05
**9**	prop_higher_edu <0.304 (p76) &prop_shrubland <0.112 (p58)	rate_local3 >0.378 (p57)	58.231	0.6463	20.247	0.1248	1.013E-05
**10**	prop_preprim_edu <0.140 (p86) &prop_higher_edu <0.219 (p56) &build_area_pp <71.407 (p53)	rate_local3 >0.280 (p48)	55.81	0.8229	20.082	0.0554	6.571E-05
**11**	med_income <27961.685 (p84) &prop_grassland <0.073 (p69)	rate_local3 >0.270 (p47)	82.884	0.7109	19.956	0.099	2.542E-05
**12**	prop_higher_edu <0.219 (p56) &den_population >15540.769 (p47)	rate_local3 >0.338 (p54)	50.087	0.7667	19.896	0.1873	5.951E-08
**13**	med_area_home <19.087 (p60) &den_population >14945.078 (p46)	rate_local3 >0.326 (p52)	64.397	0.6867	19.849	0.0883	2.724E-06
**14**	prop_higher_edu <0.231 (p62) &prop_agricultural <0.025 (p71)	rate_local3 >0.340 (p54)	58.412	0.6958	19.823	0.1248	2.188E-05
**15**	med_area_home <18.689 (p57) &den_bldg >0.115 (p37)	rate_local3 >0.370 (p57)	59.54	0.6422	19.635	0.0886	4.586E-05
**16**	prop_higher_edu <0.229 (p61) &prop_transport >0.106 (p45)	rate_local3 >0.352 (p56)	48.715	0.7463	19.582	0.1886	4.488E-06
**17**	prop_preprim_edu <0.140 (p86) &med_income <17248.143 (p53)	rate_local3 >0.270 (p47)	69.41	0.7491	19.313	0.0757	3.697E-05
**18**	prop_higher_edu <0.227 (p60) &build_area_pp <74.445 (p54)	rate_local3 >0.328 (p52)	57.58	0.711	19.271	0.1211	3.74E-05
**19**	med_income <26769.030 (p83) &prop_agricultural <0.024 (p71)	rate_local3 >0.333 (p53)	73.688	0.6329	19.271	0.093	4.217E-05
**20**	prop_higher_edu <0.242 (p63) &den_road >9.163 (p44)	rate_local3 >0.356 (p56)	48.912	0.7307	19.259	0.1855	6.003E-06
**21**	prop_preprim_edu <0.141 (p86) &med_area_home <19.467 (p61)	rate_local3 >0.242 (p41)	87.62	0.7417	19.19	0.0482	9.598E-06
**22**	med_area_home <18.950 (p58) &den_road >9.184 (p44)	rate_local3 >0.344 (p55)	58.876	0.6748	19.099	0.0958	5.323E-05
**23**	prop_higher_edu <0.221 (p57) &med_area_home <15.911 (p47)	rate_local3 >0.328 (p52)	56.046	0.7159	19.005	0.0808	8.225E-05
**24**	prop_higher_edu <0.231 (p62) &den_bldg >0.115 (p37)	rate_local3 >0.372 (p57)	51.084	0.6695	18.466	0.1356	4.1E-05

**25**	prop_over65 >0.124 (p21) &prop_preprim_edu <0.140 (p86) &prop_higher_edu <0.246 (p64)	rate_local3 >0.229 (p39)	72.878	0.7954	18.166	0.0486	5.972E-06
**26**	med_income <17170.044 (p53) &prop_open_recreation >0.028 (p47)	rate_local3 >0.337 (p54)	49.771	0.7223	17.823	0.107	3.635E-05
**27**	prop_higher_edu <0.227 (p60) &prop_grassland <0.073 (p69)	rate_local3 >0.292 (p50)	57.962	0.7368	17.671	0.117	8.038E-05
**28**	med_income <16632.840 (p51)	rate_local3 >0.341 (p54)	67.301	0.6193	17.407	0.1602	6.932E-07
**29**	prop_higher_edu <0.231 (p62) &prop_open_recreation >0.026 (p45)	rate_local3 >0.340 (p54)	49.912	0.7045	17.347	0.1335	4.437E-05
**30**	med_area_home <18.551 (p56) &prop_grassland <0.080 (p70)	rate_local3 >0.256 (p44)	70.587	0.7372	17.007	0.0631	7.149E-05
**31**	prop_higher_edu <0.212 (p54) &prop_gov_insti_faci >0.027 (p38)	rate_local3 >0.328 (p52)	49.115	0.7194	16.839	0.127	8.199E-05
**32**	med_income <28352.730 (p84) &prop_gov_insti_faci >0.026 (p38)	rate_local3 >0.190 (p35)	88.713	0.8063	16.743	0.0895	7.1E-05
**33**	prop_preprim_edu <0.140 (p86) &prop_higher_edu <0.219 (p56)	rate_local3 >0.243 (p41)	70.409	0.755	16.532	0.0839	1.199E-05
**34**	med_income <25832.400 (p81) &prop_woodland <0.072 (p38)	rate_local3 >0.348 (p55)	46.924	0.6948	16.412	0.0991	5.782E-05
**35**	med_area_home <16.859 (p50) &build_area_pp <71.501 (p53)	rate_local3 >0.184 (p33)	71.528	0.8563	16.24	0.0717	4.852E-05
**36**	prop_over65 >0.140 (p35) &prop_shrubland <0.217 (p82)	rate_local3 >0.278 (p47)	79.936	0.6626	16.127	0.0581	7.125E-05
**37**	med_area_home <19.087 (p60) &prop_gov_insti_faci >0.027 (p38)	rate_local3 >0.163 (p29)	83.121	0.8575	16.111	0.0707	8.061E-05
**38**	med_area_home <13.764 (p26)	rate_local3 >0.352 (p56)	41.012	0.7346	16.09	0.2882	1.106E-07
**39**	med_area_home <18.923 (p57) &prop_woodland <0.076 (p40)	rate_local3 >0.372 (p57)	39.567	0.7029	15.477	0.1343	6.807E-05
**40**	med_income <21114.095 (p74) &POI_den__densports >351.476 (p64)	rate_local3 >0.441 (p64)	36.084	0.6332	15.38	0.1697	4.439E-05
**41**	ave_household_size <2.986 (p63) &prop_transport >0.091 (p39)	rate_local3 >0.362 (p56)	53.192	0.6134	15.226	0.0829	7.519E-05
**42**	prop_over65 >0.120 (p19) &prop_higher_edu <0.236 (p62) &prop_transport >0.122 (p50)	rate_local3 >0.226 (p38)	48.001	0.8768	15.111	0.0361	5.101E-05
**43**	prop_preprim_edu >0.092 (p41) &den_population >15540.769 (p47)	rate_local3 >0.333 (p53)	45.161	0.7004	15.019	0.1166	7.33E-06
**44**	med_income <21058.780 (p74) &POI_den_dentelecom_elec >243.501 (p65)	rate_local3 >0.437 (p64)	34.165	0.6507	14.909	0.1791	7.416E-05
**45**	prop_over65 >0.124 (p21) &prop_higher_edu <0.246 (p64) &den_road >9.607 (p45)	rate_local3 >0.229 (p39)	49.122	0.8559	14.85	0.041	7.417E-05
**46**	med_income <20623.735 (p73) &POI_den_dentransport >589.870 (p65)	rate_local3 >0.444 (p64)	33.822	0.6423	14.827	0.177	5.501E-05

**47**	med_income <21114.095 (p74) &POI_den_denmall_mkt >146.926 (p67)	rate_local3 >0.441 (p64)	32.37	0.6645	14.672	0.1833	2.13E-05
**48**	build_area_pp <61.539 (p50)	rate_local3 >0.221 (p38)	75.937	0.7511	14.461	0.143	6.155E-06
**49**	prop_over65 >0.146 (p38) &prop_preprim_edu <0.126 (p81)	rate_local3 >0.236 (p40)	71.708	0.7342	14.401	0.0797	2.827E-05
**50**	med_income <21058.780 (p74) &POI_den_denedu >198.459 (p63)	rate_local3 >0.476 (p66)	33.942	0.5714	14.261	0.1457	5.81E-05
**51**	prop_higher_edu <0.231 (p62)	rate_local3 >0.340 (p54)	73.108	0.5707	14.226	0.1111	1.139E-05
**52**	den_population >22716.207 (p54)	rate_local3 >0.265 (p47)	67.208	0.6915	13.987	0.1439	2.766E-05
**53**	prop_transport >0.142 (p55)	rate_local3 >0.378 (p57)	54.732	0.5656	13.906	0.1437	4.175E-05
**54**	prop_higher_edu <0.243 (p63) &prop_shrubland <0.184 (p78) &POI_den_densports >338.260 (p64)	rate_local3 >0.379 (p57)	29.293	0.7881	13.651	0.0607	3.135E-05
**55**	prop_higher_edu <0.247 (p64) &den_public_trans >18.752 (p53)	rate_local3 >0.410 (p59)	38.55	0.5997	13.309	0.1175	7.118E-05
**56**	ave_household_size <2.666 (p29) &den_population >15287.373 (p47)	rate_local3 >0.424 (p63)	28.82	0.6969	13.133	0.1758	6.917E-05
**57**	prop_preprim_edu >0.092 (p41) &den_road >9.987 (p47)	rate_local3 >0.333 (p53)	41.762	0.6812	13.103	0.1298	5.529E-05
**58**	prop_higher_edu <0.312 (p77) &POI_den_denmall_mkt >184.953 (p69)	rate_local3 >0.406 (p59)	28.335	0.726	12.863	0.1983	6.937E-05
**59**	ave_household_size <2.639 (p29) &prop_rural_set <0.001 (p46)	rate_local3 >0.493 (p68)	25.062	0.6449	12.809	0.164	5.449E-05
**60**	prop_higher_edu <0.243 (p63) &POI_den_densports >338.260 (p64)	rate_local3 >0.379 (p57)	29.898	0.7275	12.602	0.2101	4.547E-05
**61**	prop_shrubland <0.100 (p56)	rate_local3 >0.420 (p61)	58.694	0.4877	12.546	0.1042	7.135E-05
**62**	prop_higher_edu <0.241 (p63) &POI_den_dentransport >517.252 (p64)	rate_local3 >0.376 (p57)	30.219	0.7204	12.44	0.1985	4.547E-05
**63**	ave_household_size <2.632 (p29) &den_road >10.043 (p47)	rate_local3 >0.451 (p64)	27.119	0.6507	12.379	0.1398	5.595E-05
**64**	prop_publicResid >0.086 (p79)	rate_local3 >0.297 (p50)	35.12	0.7799	12.359	0.2744	7.805E-06
**65**	POI_den__mall_mkt >307.693 (p78)	rate_local3 >0.547 (p72)	24.39	0.53	12.21	0.26	5.239E-06
**66**	ave_household_size <2.654 (p29) &den_bldg >0.166 (p48)	rate_local3 >0.437 (p64)	28.373	0.6379	12.058	0.1254	7.177E-05
**67**	prop_higher_edu <0.331 (p79) &prop_shrubland <0.158 (p72) &POI_den_dentelecom_elec >577.491 (p80)	rate_local3 >0.384 (p57)	23.54	0.849	11.979	0.0438	2.269E-05
**68**	prop_preprim_edu <0.141 (p86) &ave_household_size <2.654 (p29)	rate_local3 >0.437 (p64)	30.61	0.6026	11.976	0.09	6.777E-05
**69**	POI_den__sports >882.075 (p78)	rate_local3 >0.528 (p71)	24.72	0.55	11.96	0.26	4.033E-06
**70**	POI_den__telecom_elec >540.287 (p78)	rate_local3 >0.539 (p71)	24.30	0.53	11.95	0.26	2.434E-06

**71**	prop_higher_edu <0.301 (p76) &prop_woodland <0.142 (p56) &POI_den_denmall_mkt >307.589 (p78)	rate_local3 >0.376 (p57)	22.661	0.8891	11.859	0.0676	4.499E-05
**72**	prop_woodland <0.076 (p40)	rate_local3 >0.403 (p59)	45.867	0.5375	11.798	0.1383	3.989E-05
**73**	prop_higher_edu <0.331 (p79) &prop_woodland <0.198 (p65) &POI_den_dentelecom_elec >577.491 (p80)	rate_local3 >0.384 (p57)	22.916	0.854	11.727	0.0488	2.269E-05
**74**	POI_den__transport >1382.534 (p79)	rate_local3 >0.530 (p71)	24.57	0.54	11.70	0.26	1.176E-05
**75**	med_income <18952.447 (p62) &prop_industrial <0.008 (p69) &POI_den_dentransport >1251.423 (p77)	rate_local3 >0.405 (p59)	21.379	0.8708	11.618	0.1091	7.391E-05
**76**	prop_higher_edu <0.239 (p63) &prop_shrubland <0.176 (p77) &POI_den_denedu >293.660 (p70)	rate_local3 >0.386 (p57)	22.678	0.8509	11.553	0.0642	5.989E-05
**77**	prop_higher_edu <0.333 (p79) &prop_woodland <0.198 (p65) &POI_den_dentransport >1420.849 (p79)	rate_local3 >0.384 (p57)	23.389	0.8206	11.505	0.0452	6.691E-05
**78**	den_road >9.163 (p44)	rate_local3 >0.407 (p59)	59.287	0.4876	11.241	0.0924	3.309E-05
**79**	prop_higher_edu <0.239 (p63) &POI_den_denedu >293.660 (p70)	rate_local3 >0.380 (p57)	23.313	0.8068	11.171	0.2879	5.411E-05
**80**	ave_household_size <2.629 (p29) &prop_gov_insti_faci >0.025 (p36)	rate_local3 >0.426 (p63)	25.038	0.678	11.114	0.1474	6.054E-06
**81**	prop_grassland <0.000 (p15)	rate_local3 >0.498 (p68)	21.316	0.6383	10.941	0.3276	1.079E-05
**82**	POI_den__edu >611.274 (p81)	rate_local3 >0.558 (p74)	21.47	0.51	10.87	0.26	2.039E-05
**83**	prop_higher_edu <0.245 (p64) &prop_private_resid >0.098 (p66) &prop_grassland <0.061 (p64)	rate_local3 >0.371 (p57)	22.095	0.8364	10.76	0.0468	6.369E-05
**84**	prop_over65 >0.140 (p35)	rate_local3 >0.278 (p47)	85.505	0.6045	10.69	0.0756	7.794E-05
**85**	med_area_home <20.975 (p73) &POI_den_dentelecom_elec >683.674 (p83)	rate_local3 >0.345 (p55)	23.236	0.8174	10.32	0.1267	8.115E-05
**86**	prop_higher_edu <0.245 (p64) &den_bldg >0.222 (p60) &prop_private_resid >0.098 (p66)	rate_local3 >0.371 (p57)	19.5	0.8835	10.029	0.0939	4.221E-05
**87**	ave_household_size <2.766 (p37) &prop_publicResid >0.047 (p71)	rate_local3 >0.410 (p59)	18.842	0.8082	9.6904	0.2521	4.405E-05
**88**	ave_household_size <2.587 (p17)	rate_local3 >0.610 (p79)	19.369	0.4304	9.5905	0.2131	2.656E-05
**89**	prop_preprim_edu >0.093 (p43) &prop_grassland <0.052 (p62)	rate_local3 >0.247 (p42)	48.762	0.7103	9.4833	0.0818	7.965E-05
**90**	prop_higher_edu <0.221 (p57) &prop_private_resid >0.117 (p70)	rate_local3 >0.374 (p57)	16.71	0.9523	9.2353	0.4128	6.486E-05
**91**	den_bldg >0.414 (p83)	rate_local3 >0.518 (p71)	20.018	0.5331	9.0521	0.2411	4.192E-05
**92**	prop_business >0.037 (p83)	rate_local3 >0.510 (p71)	20.041	0.5433	8.9966	0.2439	2.061E-05
**93**	prop_higher_edu <0.208 (p52) &POI_den_dentelecom_elec >359.967 (p71)	rate_local3 >0.376 (p57)	17.272	0.8841	8.9912	0.3308	8.324E-05

**94**	gender_ratio <86.703 (p11)	rate_local3 >0.610 (p79)	11.686	0.5118	6.8158	0.2985	7.161E-05
**95**	den_public_trans >89.817 (p91)	rate_local3 >0.515 (p71)	12.632	0.6389	6.7963	0.3438	7.157E-05
**96**	den_population >16512.251 (p49) &POI_den_dentelecom_elec <179.880 (p60)	rate_local3 =0.289-0.529 (p50-71)	19.684	0.4457	9.1098	0.1495	4.236E-05
**97**	den_population >16512.251 (p49) &POI_den_denedu <220.422 (p64)	rate_local3 =0.289-0.529 (p50-71)	20.008	0.4123	8.3889	0.128	3.501E-05
**98**	prop_publicResid <0.078 (p77) &prop_business <0.050 (p86) &prop_industrial <0.004 (p60)	rate_local3 <0.359 (p56)	63.65	0.8362	21.049	0.121	2.425E-05
**99**	prop_publicResid <0.027 (p66) &POI_den_denmall_mkt <335.783 (p79)	rate_local3 <0.338 (p54)	80.313	0.7252	20.329	0.0893	2.637E-05
**100**	prop_publicResid <0.082 (p79) &prop_industrial <0.003 (p58) &POI_den_dentransport <1307.010 (p77)	rate_local3 <0.376 (p57)	59.258	0.872	20.148	0.1131	2.347E-06
**101**	prop_publicResid <0.096 (p81) &prop_industrial <0.003 (p57) &POI_den_denmall_mkt <321.686 (p79)	rate_local3 <0.380 (p57)	60.133	0.8696	20.113	0.1103	3.695E-06
**102**	prop_publicResid <0.076 (p77) &prop_industrial <0.003 (p58) &POI_den_dentelecom_elec <540.287 (p78)	rate_local3 <0.377 (p57)	59.001	0.8732	20.098	0.1137	3.134E-06
**103**	prop_publicResid <0.025 (p66) &prop_transport <0.214 (p67)	rate_local3 <0.368 (p57)	74.76	0.7745	20.01	0.1152	1.42E-05
**104**	prop_publicResid <0.027 (p66) &POI_den_dentelecom_elec <499.985 (p78)	rate_local3 <0.348 (p55)	79.512	0.7339	19.935	0.0898	2.517E-05
**105**	prop_publicResid <0.027 (p66) &POI_den_densports <958.264 (p81)	rate_local3 <0.338 (p54)	80.368	0.7197	19.885	0.0838	1.178E-05
**106**	prop_publicResid <0.076 (p77) &prop_industrial <0.003 (p59) &POI_den_densports <938.704 (p79)	rate_local3 <0.362 (p56)	59.586	0.8429	19.864	0.114	5.506E-05
**107**	den_public_trans <63.592 (p84) &prop_publicResid <0.078 (p77) &prop_industrial <0.003 (p58)	rate_local3 <0.379 (p57)	62.586	0.8457	19.835	0.1098	2.006E-06
**108**	med_area_home >14.812 (p39) &prop_business <0.048 (p86) &prop_industrial <0.003 (p58)	rate_local3 <0.371 (p57)	57.102	0.8741	19.812	0.1032	4.923E-06
**109**	prop_publicResid <0.091 (p80) &prop_industrial <0.003 (p58) &prop_transport <0.252 (p74)	rate_local3 <0.377 (p57)	59.365	0.8578	19.513	0.13	2.977E-06
**110**	prop_publicResid <0.027 (p66) &POI_den_dentransport <1286.158 (p77)	rate_local3 <0.338 (p54)	77.575	0.7219	19.37	0.086	5.139E-05
**111**	prop_publicResid <0.027 (p66) &POI_den_denedu <650.564 (p81)	rate_local3 <0.338 (p54)	80.476	0.7063	18.762	0.0704	7.714E-05
**112**	med_area_home >14.812 (p39) &prop_industrial <0.003 (p58) &POI_den_dentelecom_elec <540.287 (p78)	rate_local3 <0.371 (p57)	54.582	0.8665	18.625	0.0956	3.882E-05

**113**	prop_publicResid <0.078 (p77) &prop_industrial <0.003 (p58) &POI_den_denedu <499.915 (p79)	rate_local3 <0.379 (p57)	57.499	0.8479	18.326	0.1037	9.271E-06
**114**	med_area_home >15.209 (p44) &den_public_trans <59.341 (p83) &prop_industrial <0.003 (p58)	rate_local3 <0.370 (p57)	55.454	0.8462	18.118	0.0721	4.243E-05
**115**	prop_publicResid <0.025 (p66) &prop_business <0.038 (p84)	rate_local3 <0.408 (p59)	84.856	0.7668	18.055	0.0818	3.678E-05
**116**	den_population <36301.913 (p67) &prop_industrial <0.003 (p58)	rate_local3 <0.377 (p57)	63.728	0.8027	18.018	0.1409	3.453E-06
**117**	den_public_trans <47.515 (p78) &prop_publicResid <0.025 (p66)	rate_local3 <0.408 (p59)	84.845	0.7664	18.012	0.0814	3.678E-05
**118**	prop_publicResid <0.024 (p66) &prop_woodland >0.077 (p40)	rate_local3 <0.374 (p57)	64.31	0.7953	17.942	0.1277	2.094E-05
**119**	prop_higher_edu >0.199 (p49) &prop_business <0.044 (p85)	rate_local3 <0.375 (p57)	66.554	0.7748	17.175	0.0846	5.406E-05
**120**	prop_higher_edu >0.197 (p49) &prop_woodland >0.067 (p37)	rate_local3 <0.399 (p58)	56.449	0.8429	16.521	0.1376	1.936E-06
**121**	prop_industrial <0.003 (p58) &POI_den_dentelecom_elec <540.287 (p78)	rate_local3 <0.377 (p57)	67.511	0.7595	16.333	0.1232	5.593E-05
**122**	prop_higher_edu >0.170 (p37) &prop_business <0.051 (p86) &prop_industrial <0.003 (p57)	rate_local3 <0.410 (p59)	57.42	0.8453	16.223	0.103	3.901E-05
**123**	prop_industrial <0.003 (p58) &POI_den_dentransport <1307.010 (p77)	rate_local3 <0.376 (p57)	67.135	0.7589	16.222	0.124	5.965E-05
**124**	prop_higher_edu >0.225 (p59) &ave_household_size >2.579 (p17)	rate_local3 <0.321 (p52)	54.23	0.7515	16.186	0.0766	5.451E-05
**125**	build_area_pp >65.200 (p51) &prop_business <0.069 (p88) &prop_industrial <0.003 (p59)	rate_local3 <0.376 (p57)	43.03	0.9145	15.966	0.1292	1.063E-05
**126**	prop_industrial <0.003 (p58) &POI_den_densports <741.357 (p78)	rate_local3 <0.376 (p57)	65.763	0.7583	15.905	0.1224	8.074E-05
**127**	med_area_home >19.743 (p65)	rate_local3 <0.352 (p56)	58.77	0.7604	15.899	0.2057	3.995E-07
**128**	prop_publicResid <0.013 (p62) &prop_shrubland >0.047 (p43)	rate_local3 <0.376 (p57)	60.643	0.7787	15.887	0.1061	5.787E-05
**129**	prop_industrial <0.003 (p58) &POI_den_denmall_mkt <294.792 (p78)	rate_local3 <0.377 (p57)	65.554	0.752	15.361	0.1168	8.074E-05
**130**	ave_household_size >2.657 (p29) &prop_industrial <0.003 (p58)	rate_local3 <0.376 (p57)	62.382	0.7615	15.304	0.1294	7.073E-06
**131**	med_income >22184.362 (p77)	rate_local3 <0.341 (p54)	43.895	0.8221	14.847	0.2781	2.542E-07
**132**	ave_household_size >2.850 (p49) &prop_publicResid <0.095 (p81) &prop_industrial <0.003 (p58)	rate_local3 <0.336 (p53)	43.015	0.8194	14.695	0.09	7.53E-05
**133**	den_public_trans <32.922 (p67) &prop_industrial <0.003 (p58)	rate_local3 <0.377 (p57)	59.244	0.7654	14.678	0.1277	4.84E-05
**134**	build_area_pp >61.539 (p50)	rate_local3 <0.221 (p38)	58.629	0.515	14.367	0.1262	6.155E-06
**135**	den_population <22716.207 (p54)	rate_local3 <0.265 (p47)	66.938	0.5745	14.356	0.1232	2.766E-05

**136**	prop_gov_insti_faci <0.023 (p33)	rate_local3 <0.011 (p15)	25.789	0.3578	14.338	0.1989	5.041E-09
**137**	prop_higher_edu >0.231 (p62)	rate_local3 <0.340 (p54)	60.577	0.7058	13.932	0.1623	1.139E-05
**138**	prop_transport <0.142 (p55)	rate_local3 <0.378 (p57)	81.392	0.695	13.789	0.1178	4.175E-05
**139**	prop_shrubland >0.100 (p56)	rate_local3 <0.420 (p61)	70.629	0.7522	12.762	0.1359	7.135E-05
**140**	prop_publicResid <0.086 (p79)	rate_local3 <0.297 (p50)	95.928	0.5684	12.553	0.0744	7.805E-06
**141**	prop_industrial <0.003 (p58) &POI_den_denedu <70.598 (p46)	rate_local3 <0.380 (p57)	38.606	0.8501	12.324	0.1834	3.615E-05
**142**	POI_den_denmall_mkt <307.693 (p78)	rate_local3 <0.547 (p72)	135.67	0.8082	12.286	0.0732	5.239E-06
**143**	POI_den__telecom_elec <540.287 (p78)	rate_local3 <0.539 (p71)	134.81	0.80	12.01	0.07	2.434E-06
**144**	POI_den__sports <882.075 (p78)	rate_local3 <0.528 (p71)	133.09	0.79	11.89	0.07	4.033E-06
**145**	POI_den__transport <1382.534 (p79)	rate_local3 <0.530 (p71)	132.54	0.79	11.58	0.07	1.176E-05
**146**	den_road <9.163 (p44)	rate_local3 <0.407 (p59)	67.18	0.7267	11.336	0.1226	3.309E-05
**147**	prop_open_recreation <0.099 (p78) &prop_woodland >0.173 (p62)	rate_local3 <0.508 (p70)	64.928	0.8366	11.129	0.0321	3.972E-05
**148**	prop_open_recreation <0.016 (p36)	rate_local3 <0.013 (p15)	22.835	0.3012	10.789	0.1423	1.963E-05
**149**	POI_den_denedu <611.274 (p81)	rate_local3 <0.558 (p74)	139.02	0.807	10.742	0.0624	2.039E-05
**150**	prop_woodland >0.312 (p81)	rate_local3 <0.403 (p59)	35.086	0.8626	10.684	0.2627	7.471E-06
**151**	prop_over65 <0.140 (p35)	rate_local3 <0.278 (p47)	44.728	0.6149	10.591	0.1456	7.794E-05
**152**	prop_private_resid <0.022 (p37)	rate_local3 <0.013 (p15)	23	0.2924	10.502	0.1335	5.149E-05
**153**	ave_household_size >3.008 (p74)	rate_local3 <0.610 (p79)	60.542	0.936	9.8406	0.1521	6.605E-05
**154**	den_bldg <0.414 (p83)	rate_local3 <0.518 (p71)	133.97	0.7577	8.7898	0.0497	4.192E-05
**155**	prop_business <0.037 (p83)	rate_local3 <0.510 (p71)	132.89	0.7501	8.7862	0.0496	2.061E-05
**156**	prop_industrial <0.003 (p57) &prop_agricultural >0.017 (p64)	rate_local3 <0.486 (p67)	31.377	0.9309	8.4727	0.1713	4.243E-05
**157**	prop_preprim_edu >0.146 (p89)	rate_local3 <0.021 (p15)	11.836	0.523	8.2401	0.3641	4.683E-07
**158**	prop_household_3gen >0.058 (p84)	rate_local3 <0.013 (p15)	13.594	0.3959	8.1391	0.2371	1.984E-05
**159**	prop_private_resid >0.104 (p66) &prop_transport <0.221 (p68)	rate_local3 <0.284 (p49)	22.581	0.7428	7.9125	0.194	7.598E-05
**160**	den_public_trans <89.817 (p91)	rate_local3 <0.515 (p71)	143.72	0.7394	6.7434	0.0347	7.157E-05

(i) Rules for wave-3 local case rate; POI density was used

No.	Antecedent	Consequent	Supp	Conf	Lev	Imp	*P*
**1**	med_income <23455.645 (p78) &den_road >8.039 (p38) &LU_entropy <0.548 (p48)	rate_local4 >0.788 (p77)	31.38	0.77	21.68	0.20	2.075E-06
**2**	med_income <23474.184 (p78) &prop_grassland <0.022 (p47) &LU_entropy <0.554 (p50)	rate_local4 >0.796 (p77)	31.02	0.76	21.50	0.19	3.727E-06
**3**	den_population >18592.692 (p50) &prop_agricultural <0.010 (p59) &prop_shrubland <0.097 (p55)	rate_local4 >0.466 (p46)	60.83	0.81	21.48	0.05	8.88E-06
**4**	med_income <23183.658 (p78) &prop_transport >0.087 (p38) &LU_entropy <0.547 (p47)	rate_local4 >0.817 (p78)	30.05	0.78	21.45	0.20	1.32E-05
**5**	med_income <23814.728 (p79) &prop_shrubland <0.020 (p36) &LU_entropy <0.553 (p49)	rate_local4 >0.787 (p77)	30.18	0.79	21.01	0.18	2.951E-06
**6**	prop_over65 >0.109 (p11) &prop_transport >0.142 (p55) &prop_agricultural <0.012 (p60)	rate_local4 >0.431 (p43)	63.92	0.84	21.01	0.05	1.296E-05
**7**	med_income <23474.184 (p78) &prop_agricultural <0.010 (p59) &LU_entropy <0.554 (p50)	rate_local4 >0.792 (p77)	32.26	0.68	21.00	0.18	2.647E-06
**8**	med_income <23474.184 (p78) &prop_woodland <0.028 (p29) &LU_entropy <0.554 (p50)	rate_local4 >0.817 (p78)	29.45	0.78	20.96	0.12	1.17E-05
**9**	prop_transport >0.128 (p52) &LU_entropy <0.547 (p47)	rate_local4 >0.789 (p77)	33.42	0.64	20.86	0.25	2.149E-08
**10**	den_population >18592.692 (p50) &den_road >10.356 (p49) &prop_agricultural <0.010 (p59)	rate_local4 >0.466 (p46)	61.21	0.79	20.82	0.05	3.644E-06
**11**	med_income <22034.302 (p77) &den_bldg >0.134 (p42) &LU_entropy <0.547 (p47)	rate_local4 >0.806 (p78)	29.56	0.77	20.78	0.21	2.809E-05
**12**	med_income <24553.043 (p79) &LU_entropy <0.548 (p47) &POI_den_telecom_elec >223.612 (p65)	rate_local4 >0.811 (p78)	29.89	0.74	20.74	0.18	1.844E-05
**13**	den_population >17099.042 (p50) &prop_rural_set <0.039 (p71) &prop_shrubland <0.097 (p55)	rate_local4 >0.466 (p46)	59.77	0.80	20.74	0.05	3.983E-06
**14**	prop_grassland <0.017 (p43) &LU_entropy <0.547 (p47)	rate_local4 >0.803 (p78)	33.11	0.61	20.56	0.20	3.356E-07
**15**	prop_higher_edu <0.363 (p85) &den_road >8.189 (p38) &LU_entropy <0.548 (p48)	rate_local4 >0.808 (p78)	30.14	0.71	20.41	0.14	5.557E-06
**16**	med_income <22534.375 (p78) &LU_entropy <0.549 (p48) &POI_den_sports >368.773 (p64)	rate_local4 >0.821 (p79)	28.85	0.76	20.39	0.19	5.263E-05
**17**	den_road >9.940 (p47) &LU_entropy <0.548 (p48)	rate_local4 >0.788 (p77)	34.15	0.59	20.36	0.21	1.063E-07
**18**	med_income <23794.776 (p79) &prop_agricultural <0.017 (p64) &prop_shrubland <0.118 (p60)	rate_local4 >0.442 (p43)	61.86	0.82	20.34	0.09	6.161E-05

**19**	med_income <27018.204 (p84) &prop_rural_set <0.024 (p65) &prop_shrubland <0.118 (p60)	rate_local4 >0.443 (p43)	58.54	0.84	20.32	0.09	3.687E-05
**20**	med_income <24553.043 (p79) &POI_den_mall_mkt >163.832 (p69)	rate_local4 >0.788 (p77)	32.02	0.65	20.24	0.14	4.493E-05
**21**	med_income <21397.583 (p75) &prop_rural_set <0.002 (p49) &LU_entropy <0.549 (p48)	rate_local4 >0.791 (p77)	29.43	0.76	20.21	0.21	1.275E-05
**22**	den_bldg >0.162 (p47) &LU_entropy <0.546 (p47)	rate_local4 >0.786 (p77)	34.07	0.59	20.17	0.21	6.677E-08
**23**	ave_household_size <2.934 (p63) &prop_agricultural <0.010 (p59) &LU_entropy <0.558 (p50)	rate_local4 >0.824 (p79)	31.12	0.62	20.12	0.13	3.923E-05
**24**	med_area_home <18.625 (p56) &prop_grassland <0.018 (p45) &LU_entropy <0.550 (p49)	rate_local4 >0.807 (p78)	27.81	0.82	20.10	0.23	5.435E-06
**25**	prop_rural_set <0.024 (p65) &prop_shrubland <0.110 (p58)	rate_local4 >0.548 (p58)	59.45	0.66	20.09	0.10	1.347E-05
**26**	med_area_home <19.189 (p61) &den_road >8.189 (p38) &LU_entropy <0.548 (p48)	rate_local4 >0.808 (p78)	28.67	0.76	20.06	0.19	2.302E-05
**27**	prop_shrubland <0.021 (p37) &LU_entropy <0.543 (p46)	rate_local4 >0.787 (p77)	32.28	0.63	20.02	0.19	6.655E-08
**28**	med_income <24602.040 (p79) &POI_den_transport >860.459 (p70)	rate_local4 >0.783 (p76)	31.15	0.68	19.98	0.13	4.866E-05
**29**	med_income <24606.472 (p79) &POI_den_telecom_elec >297.186 (p68)	rate_local4 >0.792 (p77)	31.64	0.64	19.95	0.14	4.316E-05
**30**	med_income <28797.450 (p84) &prop_grassland <0.005 (p29)	rate_local4 >0.823 (p79)	29.69	0.67	19.94	0.14	7.623E-05
**31**	ave_household_size <2.934 (p63) &prop_grassland <0.009 (p34) &LU_entropy <0.558 (p50)	rate_local4 >0.824 (p79)	28.39	0.74	19.93	0.14	6.342E-05
**32**	prop_rural_set <0.002 (p49) &LU_entropy <0.549 (p48)	rate_local4 >0.791 (p77)	35.32	0.55	19.91	0.17	3.676E-07
**33**	den_population >18592.692 (p50) &prop_shrubland <0.097 (p55)	rate_local4 >0.526 (p56)	58.58	0.69	19.91	0.09	2.575E-05
**34**	med_income <23474.184 (p78) &prop_woodland <0.015 (p24)	rate_local4 >0.817 (p78)	29.11	0.71	19.91	0.13	3.975E-05
**35**	med_income <23243.595 (p78) &POI_den_sports >464.930 (p68)	rate_local4 >0.804 (p78)	31.32	0.63	19.90	0.14	5.095E-05
**36**	med_income <23878.187 (p79) &LU_entropy <0.551 (p49) &POI_den_edu >204.703 (p63)	rate_local4 >0.822 (p79)	28.56	0.73	19.87	0.18	1.417E-05
**37**	den_population >20156.904 (p52) &den_bldg >0.213 (p59)	rate_local4 >0.525 (p55)	51.35	0.75	19.81	0.08	2.44E-05
**38**	med_income <23474.184 (p78) &den_public_trans >16.405 (p50) &LU_entropy <0.555 (p50)	rate_local4 >0.817 (p78)	29.02	0.70	19.79	0.15	5.972E-05
**39**	med_income <27485.399 (p84) &prop_transport >0.077 (p36) &prop_agricultural <0.012 (p60)	rate_local4 >0.394 (p39)	70.68	0.84	19.73	0.06	4.522E-05

**40**	den_population >18592.692 (p50) &prop_agricultural <0.010 (p59)	rate_local4 >0.447 (p43)	68.58	0.76	19.67	0.06	5.54E-06
**41**	prop_transport >0.106 (p45) &prop_agricultural <0.010 (p59)	rate_local4 >0.442 (p43)	73.70	0.75	19.65	0.06	7.072E-05
**42**	med_income <27485.399 (p84) &den_road >10.530 (p49) &prop_agricultural <0.009 (p59)	rate_local4 >0.394 (p39)	63.34	0.88	19.65	0.07	5.317E-05
**43**	prop_agricultural <0.013 (p61) &prop_shrubland <0.114 (p58)	rate_local4 >0.549 (p58)	62.04	0.63	19.64	0.09	6.993E-05
**44**	med_area_home <18.625 (p56) &prop_shrubland <0.137 (p65) &LU_entropy <0.550 (p49)	rate_local4 >0.807 (p78)	27.63	0.79	19.59	0.20	2.112E-06
**45**	prop_woodland <0.098 (p47) &LU_entropy <0.543 (p46)	rate_local4 >0.806 (p78)	31.56	0.60	19.48	0.22	7.047E-07
**46**	ave_household_size <2.942 (p63) &prop_shrubland <0.146 (p67) &LU_entropy <0.553 (p49)	rate_local4 >0.801 (p78)	30.53	0.64	19.48	0.13	4.112E-05
**47**	med_area_home <18.434 (p56) &den_bldg >0.140 (p43) &LU_entropy <0.554 (p50)	rate_local4 >0.804 (p78)	27.68	0.77	19.44	0.19	2.949E-05
**48**	LU_entropy <0.546 (p47) &POI_den_telecom_elec >223.612 (p65)	rate_local4 >0.817 (p78)	32.28	0.56	19.43	0.13	4.9E-05
**49**	POI_den_transport >904.959 (p71)	rate_local4 >0.726 (p73)	36.27	0.60	19.27	0.32	1.954E-14
**50**	ave_household_size <2.826 (p49) &prop_rural_set <0.022 (p64) &prop_shrubland <0.125 (p61)	rate_local4 >0.722 (p72)	34.34	0.64	19.25	0.10	6.966E-05
**51**	den_road >16.034 (p70)	rate_local4 >0.759 (p74)	35.84	0.56	19.14	0.30	2.191E-12
**52**	POI_den_mall_mkt >208.752 (p73)	rate_local4 >0.753 (p74)	34.64	0.58	19.11	0.32	1.821E-11
**53**	POI_den_sports >598.644 (p72)	rate_local4 >0.751 (p74)	34.76	0.58	19.11	0.32	9.183E-12
**54**	med_area_home <17.698 (p54) &prop_transport >0.103 (p44) &LU_entropy <0.553 (p49)	rate_local4 >0.797 (p77)	27.09	0.80	19.10	0.18	6.196E-05
**55**	med_area_home <18.625 (p56) &prop_woodland <0.084 (p42) &LU_entropy <0.550 (p49)	rate_local4 >0.807 (p78)	26.53	0.81	19.00	0.22	1.544E-05
**56**	LU_entropy <0.548 (p48) &POI_den_edu >204.703 (p63)	rate_local4 >0.788 (p77)	32.48	0.57	18.90	0.14	5.243E-05
**57**	med_income <27504.973 (p84) &den_public_trans >16.600 (p50) &prop_shrubland <0.099 (p55)	rate_local4 >0.437 (p43)	60.87	0.81	18.88	0.07	4.031E-05
**58**	prop_woodland <0.111 (p50) &POI_den_edu >253.687 (p65)	rate_local4 >0.761 (p74)	32.92	0.60	18.77	0.10	7.123E-05
**59**	den_population >19181.514 (p50) &LU_entropy <0.547 (p47)	rate_local4 >0.748 (p74)	31.41	0.66	18.75	0.25	2.886E-08
**60**	prop_agricultural <0.002 (p47) &LU_entropy <0.543 (p46)	rate_local4 >0.811 (p78)	32.48	0.53	18.69	0.16	1.313E-05
**61**	prop_agricultural <0.000 (p37)	rate_local4 >0.709 (p71)	41.79	0.53	18.66	0.24	1.304E-10
**62**	POI_den_telecom_elec >374.340 (p72)	rate_local4 >0.741 (p74)	34.54	0.58	18.63	0.31	9.927E-12
**63**	den_public_trans >16.405 (p50) &LU_entropy <0.555 (p50)	rate_local4 >0.790 (p77)	32.08	0.57	18.56	0.20	2.016E-08
**64**	prop_transport >0.103 (p43) &prop_woodland <0.119 (p52)	rate_local4 >0.760 (p74)	40.00	0.48	18.52	0.10	2.999E-05

**65**	ave_household_size <2.826 (p49) &prop_rural_set <0.047 (p73) &prop_grassland <0.030 (p54)	rate_local4 >0.722 (p72)	33.27	0.63	18.42	0.10	3.786E-05
**66**	den_bldg >0.240 (p64)	rate_local4 >0.681 (p70)	42.91	0.55	18.42	0.24	2.469E-09
**67**	med_income <24159.180 (p79) &den_bldg >0.291 (p72)	rate_local4 >0.784 (p76)	29.39	0.63	18.17	0.13	2.873E-05
**68**	prop_woodland <0.013 (p23)	rate_local4 >0.786 (p77)	29.80	0.62	18.15	0.38	9.845E-13
**69**	med_income <24553.043 (p79) &POI_den_edu >218.294 (p64)	rate_local4 >0.847 (p80)	30.50	0.52	18.11	0.11	6.863E-05
**70**	prop_higher_edu <0.357 (p84) &den_bldg >0.189 (p53) &LU_entropy <0.549 (p48)	rate_local4 >0.878 (p80)	25.79	0.65	17.98	0.13	7.241E-05
**71**	prop_shrubland <0.000 (p0)	rate_local4 >0.777 (p76)	27.26	0.72	17.87	0.47	1.265E-13
**72**	prop_over65 >0.114 (p15) &den_public_trans >26.736 (p59) &prop_shrubland <0.099 (p55)	rate_local4 >0.437 (p43)	55.51	0.82	17.87	0.05	4.406E-05
**73**	med_income <28872.090 (p84) &prop_rural_set <0.050 (p74) &prop_transport >0.064 (p35)	rate_local4 >0.342 (p36)	77.49	0.86	17.80	0.06	5.14E-05
**74**	prop_over65 >0.109 (p11) &den_road >18.717 (p77)	rate_local4 >0.573 (p61)	35.49	0.82	17.75	0.08	3.421E-05
**75**	med_area_home <16.794 (p50) &LU_entropy <0.555 (p50)	rate_local4 >0.804 (p78)	27.61	0.65	17.74	0.28	3.167E-08
**76**	prop_grassland <0.002 (p20)	rate_local4 >0.810 (p78)	27.51	0.64	17.74	0.41	4.673E-13
**77**	prop_agricultural <0.014 (p61) &prop_woodland <0.092 (p45)	rate_local4 >0.719 (p71)	38.48	0.53	17.71	0.09	7.405E-05
**78**	prop_rural_set <0.017 (p62) &prop_woodland <0.093 (p45)	rate_local4 >0.706 (p71)	38.12	0.55	17.71	0.11	7.082E-05
**79**	prop_over65 >0.109 (p11) &prop_agricultural <0.000 (p38)	rate_local4 >0.450 (p44)	56.12	0.79	17.69	0.06	6.022E-05
**80**	den_road >8.024 (p38) &prop_agricultural <0.012 (p60)	rate_local4 >0.537 (p56)	65.85	0.61	17.68	0.06	6.882E-05
**81**	med_income <28502.705 (p84) &prop_agricultural <0.010 (p59)	rate_local4 >0.394 (p39)	76.88	0.78	17.58	0.07	5.216E-05
**82**	med_income <23832.199 (p79) &prop_transport >0.245 (p72)	rate_local4 >0.801 (p78)	28.25	0.61	17.56	0.10	7.895E-05
**83**	prop_rural_set <0.013 (p59) &prop_grassland <0.027 (p51)	rate_local4 >0.529 (p56)	56.67	0.66	17.50	0.07	5.015E-05
**84**	den_public_trans >17.793 (p52) &prop_woodland <0.107 (p50)	rate_local4 >0.783 (p76)	34.17	0.50	17.45	0.12	7.135E-05
**85**	den_bldg >0.109 (p36) &prop_woodland <0.111 (p50)	rate_local4 >0.749 (p74)	40.58	0.46	17.45	0.07	8.156E-05
**86**	prop_rural_set <0.051 (p75) &prop_transport >0.079 (p36)	rate_local4 >0.422 (p42)	79.96	0.73	17.40	0.06	2.295E-05
**87**	med_area_home <17.020 (p52) &prop_agricultural <0.012 (p60) &prop_shrubland <0.241 (p86)	rate_local4 >0.366 (p36)	65.92	0.86	17.36	0.03	5.065E-05
**88**	den_population >18592.692 (p50) &den_road >15.388 (p69)	rate_local4 >0.466 (p46)	47.11	0.83	17.35	0.06	1.854E-05
**89**	ave_household_size <2.904 (p63) &LU_entropy <0.553 (p49)	rate_local4 >0.818 (p78)	30.27	0.52	17.30	0.16	7.853E-05
**90**	prop_private_resid >0.086 (p63) &prop_woodland <0.093 (p45)	rate_local4 >0.780 (p76)	30.13	0.57	17.25	0.16	1.712E-05
**91**	med_income <23474.184 (p78) &prop_private_resid >0.049 (p50) &LU_entropy <0.551 (p49)	rate_local4 >0.817 (p78)	24.80	0.73	17.20	0.20	2.791E-05

**92**	med_income <26724.437 (p83) &den_road >18.654 (p77)	rate_local4 >0.801 (p78)	26.01	0.68	17.16	0.11	6.371E-05
**93**	ave_household_size <2.779 (p37) &prop_rural_set <0.004 (p51)	rate_local4 >0.708 (p71)	32.35	0.62	17.16	0.19	5.292E-05
**94**	med_income <21422.604 (p75) &prop_shrubland <0.121 (p60) &POI_den_sports >492.479 (p69)	rate_local4 >0.545 (p57)	36.54	0.83	17.15	0.05	8.078E-05
**95**	prop_transport >0.224 (p69)	rate_local4 >0.799 (p77)	32.52	0.49	16.97	0.26	5.913E-11
**96**	med_income <23474.184 (p78) &prop_open_recreation >0.029 (p47) &LU_entropy <0.554 (p50)	rate_local4 >0.792 (p77)	25.02	0.73	16.85	0.18	6.076E-05
**97**	ave_household_size <2.749 (p37) &prop_grassland <0.008 (p32)	rate_local4 >0.847 (p80)	24.33	0.67	16.74	0.20	2.298E-05
**98**	prop_private_resid >0.060 (p55) &LU_entropy <0.549 (p48)	rate_local4 >0.787 (p77)	29.01	0.57	16.73	0.19	4.317E-06
**99**	ave_household_size <2.749 (p37) &prop_agricultural <0.001 (p44)	rate_local4 >0.720 (p71)	29.90	0.64	16.71	0.20	6.566E-05
**100**	ave_household_size <2.779 (p37) &prop_shrubland <0.076 (p51)	rate_local4 >0.708 (p71)	32.87	0.59	16.64	0.16	7.191E-05
**101**	med_income <23824.720 (p79) &prop_private_resid >0.097 (p65)	rate_local4 >0.799 (p77)	27.80	0.58	16.64	0.16	4.271E-05
**102**	den_public_trans >22.586 (p56) &prop_shrubland <0.099 (p55)	rate_local4 >0.437 (p43)	62.74	0.76	16.63	0.05	4.496E-05
**103**	ave_household_size <2.792 (p37) &den_population >15122.431 (p46) &prop_rural_set <0.008 (p56)	rate_local4 >0.565 (p60)	35.31	0.78	16.43	0.10	2.842E-06
**104**	med_income <27224.417 (p84) &den_bldg >0.169 (p50) &prop_industrial <0.000 (p43)	rate_local4 >0.900 (p81)	23.02	0.65	16.40	0.22	9.264E-06
**105**	den_population >29701.369 (p63)	rate_local4 >0.428 (p42)	63.16	0.76	16.36	0.20	4.413E-07
**106**	med_income <24215.184 (p79) &prop_industrial <0.000 (p0) &POI_den_edu >218.294 (p64)	rate_local4 >0.910 (p81)	22.07	0.71	16.36	0.23	7.184E-05
**107**	build_area_pp <190.427 (p88) &prop_agricultural <0.014 (p61)	rate_local4 >0.304 (p29)	98.10	0.84	16.22	0.04	8.088E-05
**108**	med_area_home <19.541 (p61) &prop_agricultural <0.007 (p55) &LU_entropy <0.528 (p40)	rate_local4 >0.686 (p70)	27.32	0.76	16.17	0.13	7.723E-05
**109**	med_income <24831.420 (p79) &prop_publicResid <0.037 (p70) &prop_transport >0.123 (p50)	rate_local4 >0.829 (p79)	25.01	0.61	16.10	0.17	5.443E-05
**110**	med_income <26859.289 (p83) &den_bldg >0.104 (p35) &prop_shrubland <0.125 (p61)	rate_local4 >0.336 (p35)	78.48	0.84	16.08	0.05	7.191E-05
**111**	med_income <24553.043 (p79) &prop_industrial <0.000 (p0) &prop_woodland <0.070 (p37)	rate_local4 >0.869 (p80)	21.39	0.80	16.04	0.19	7.757E-06
**112**	med_income <24553.043 (p79) &den_road >12.323 (p57) &prop_industrial <0.000 (p0)	rate_local4 >0.893 (p81)	21.91	0.71	16.04	0.23	6.314E-05

**113**	ave_household_size <2.904 (p63) &prop_private_resid >0.075 (p60) &LU_entropy <0.553 (p49)	rate_local4 >0.809 (p78)	22.81	0.77	16.02	0.21	3.412E-05
**114**	med_income <24831.420 (p79) &prop_publicResid <0.037 (p70) &prop_shrubland <0.021 (p37)	rate_local4 >0.846 (p80)	23.43	0.66	16.01	0.17	7.987E-05
**115**	prop_open_recreation >0.029 (p47) &LU_entropy <0.554 (p50)	rate_local4 >0.792 (p77)	27.94	0.55	15.87	0.18	2.402E-05
**116**	med_income <24553.043 (p79) &prop_industrial <0.000 (p0) &LU_entropy <0.548 (p48)	rate_local4 >0.910 (p81)	21.56	0.68	15.74	0.21	6.327E-05
**117**	ave_household_size <2.707 (p37) &den_bldg >0.180 (p51)	rate_local4 >0.828 (p79)	25.94	0.55	15.69	0.15	7.309E-05
**118**	med_income <23837.723 (p79) &prop_industrial <0.000 (p0) &prop_shrubland <0.037 (p41)	rate_local4 >0.869 (p80)	21.47	0.73	15.61	0.21	8.15E-05
**119**	prop_industrial <0.000 (p0) &prop_woodland <0.094 (p45)	rate_local4 >0.856 (p80)	23.98	0.59	15.58	0.22	1.603E-05
**120**	med_income <27750.705 (p84) &den_public_trans >18.296 (p53)	rate_local4 >0.366 (p36)	72.86	0.81	15.57	0.05	6.584E-05
**121**	med_income <27423.226 (p84) &prop_industrial <0.001 (p48) &prop_transport >0.089 (p39)	rate_local4 >0.919 (p81)	23.02	0.55	15.47	0.17	2.014E-05
**122**	med_income <28872.090 (p84) &den_road >9.412 (p44) &prop_rural_set <0.050 (p74)	rate_local4 >0.297 (p29)	70.24	0.90	15.44	0.05	2.952E-05
**123**	med_area_home <17.020 (p52) &prop_rural_set <0.040 (p71) &prop_shrubland <0.241 (p86)	rate_local4 >0.309 (p30)	69.21	0.89	15.41	0.05	7.014E-06
**124**	med_income <23475.477 (p78) &den_road >10.922 (p51) &prop_publicResid <0.024 (p66)	rate_local4 >0.878 (p80)	22.54	0.62	15.40	0.21	5.402E-05
**125**	prop_shrubland <0.149 (p68) &POI_den_edu >230.921 (p64)	rate_local4 >0.437 (p43)	56.18	0.77	15.39	0.04	7.177E-05
**126**	den_population >18592.692 (p50) &prop_grassland <0.011 (p36)	rate_local4 >0.466 (p46)	47.10	0.78	15.39	0.07	1.701E-05
**127**	med_income <24792.419 (p79) &prop_industrial <0.001 (p50) &prop_grassland <0.036 (p58)	rate_local4 >0.964 (p84)	21.99	0.54	15.38	0.18	3.553E-05
**128**	med_income <28003.284 (p84) &prop_rural_set <0.001 (p48)	rate_local4 >0.339 (p35)	64.53	0.87	15.35	0.08	5.209E-05
**129**	med_income <24054.127 (p79) &den_public_trans >18.631 (p53) &prop_publicResid <0.024 (p66)	rate_local4 >0.774 (p75)	24.08	0.68	15.33	0.19	4.364E-05
**130**	med_area_home <17.020 (p52) &prop_agricultural <0.012 (p60)	rate_local4 >0.329 (p33)	70.04	0.86	15.30	0.09	6.1E-05
**131**	ave_household_size <2.904 (p63) &prop_private_resid >0.090 (p64)	rate_local4 >0.792 (p77)	25.32	0.60	15.28	0.18	4.788E-05
**132**	ave_household_size <2.792 (p37) &den_population >15122.431 (p46)	rate_local4 >0.753 (p74)	29.46	0.54	15.27	0.15	7.53E-05

**133**	med_income <23878.187 (p79) &den_population >20544.461 (p52) &LU_entropy <0.514 (p38)	rate_local4 >0.811 (p78)	21.80	0.76	15.16	0.13	4.218E-05
**134**	ave_household_size <2.749 (p37) &prop_industrial <0.000 (p0)	rate_local4 >0.844 (p80)	22.60	0.62	14.96	0.25	1.409E-05
**135**	prop_higher_edu <0.331 (p79) &prop_rural_set <0.039 (p71) &prop_shrubland <0.122 (p60)	rate_local4 >0.311 (p30)	61.36	0.91	14.70	0.07	1.427E-05
**136**	den_road >8.269 (p39) &prop_shrubland <0.145 (p67)	rate_local4 >0.391 (p39)	86.89	0.73	14.69	0.03	7.999E-05
**137**	prop_over65 >0.114 (p15) &den_public_trans >29.911 (p62)	rate_local4 >0.356 (p36)	59.72	0.85	14.53	0.04	8.011E-05
**138**	prop_household_3gen <0.078 (p91) &prop_transport >0.093 (p39)	rate_local4 >0.241 (p21)	102.18	0.88	14.45	0.02	4.721E-05
**139**	med_income <24793.016 (p79) &den_bldg >0.109 (p36) &prop_publicResid <0.023 (p66)	rate_local4 >0.866 (p80)	24.10	0.50	14.45	0.16	4.661E-05
**140**	prop_higher_edu <0.379 (p87) &prop_shrubland <0.181 (p78) &prop_grassland <0.047 (p61)	rate_local4 >0.297 (p29)	87.77	0.84	14.44	0.02	4.164E-05
**141**	den_bldg >0.104 (p35) &prop_shrubland <0.145 (p67)	rate_local4 >0.391 (p39)	90.66	0.72	14.42	0.04	1.077E-06
**142**	prop_preprim_edu <0.148 (p90) &med_income <23790.502 (p79) &prop_grassland <0.040 (p59)	rate_local4 >0.234 (p21)	81.46	0.92	14.38	0.03	4.948E-05
**143**	build_area_pp <104.067 (p69) &prop_rural_set <0.046 (p73) &prop_shrubland <0.213 (p82)	rate_local4 >0.299 (p29)	74.57	0.87	14.29	0.05	5.959E-05
**144**	prop_private_resid >0.091 (p64)	rate_local4 >0.753 (p74)	34.63	0.44	14.26	0.18	1.071E-05
**145**	prop_higher_edu <0.361 (p84) &den_bldg >0.197 (p55) &prop_industrial <0.000 (p0)	rate_local4 >0.883 (p81)	20.45	0.63	14.12	0.15	1.826E-05
**146**	med_income <21397.583 (p75) &prop_publicResid <0.011 (p61) &prop_rural_set <0.002 (p49)	rate_local4 >0.791 (p77)	20.92	0.73	14.08	0.25	8.326E-05
**147**	med_income <31100.793 (p88) &den_road >11.705 (p54) &prop_shrubland <0.149 (p68)	rate_local4 >0.294 (p29)	69.48	0.89	14.08	0.01	7.267E-05
**148**	med_area_home <16.868 (p50) &prop_grassland <0.003 (p23)	rate_local4 >0.879 (p81)	19.64	0.69	14.07	0.15	2.445E-05
**149**	med_income <28097.287 (p84) &prop_shrubland <0.181 (p78) &prop_grassland <0.047 (p61)	rate_local4 >0.274 (p28)	86.48	0.87	14.03	0.02	1.325E-05
**150**	LU_entropy <0.545 (p46)	rate_local4 >0.840 (p79)	35.12	0.36	14.02	0.14	1.637E-08
**151**	med_area_home <17.698 (p54) &den_public_trans >17.842 (p52) &prop_shrubland <0.226 (p84)	rate_local4 >0.275 (p28)	69.63	0.90	13.83	0.01	4.31E-06
**152**	prop_higher_edu <0.379 (p87) &den_road >10.254 (p48)	rate_local4 >0.282 (p28)	79.35	0.87	13.71	0.04	3.768E-05
**153**	den_road >10.435 (p49) &prop_utilities <0.003 (p36)	rate_local4 >0.770 (p75)	22.41	0.64	13.64	0.25	6.063E-05
**154**	den_public_trans >23.156 (p57) &prop_publicResid <0.014 (p62)	rate_local4 >0.850 (p80)	23.02	0.52	13.63	0.17	4.873E-05

**155**	prop_higher_edu <0.322 (p79) &den_bldg >0.167 (p49) &prop_shrubland <0.156 (p70)	rate_local4 >0.282 (p28)	67.06	0.89	13.35	0.01	1.947E-05
**156**	med_income <28423.363 (p84) &med_area_home <17.098 (p52) &prop_rural_set <0.009 (p57)	rate_local4 >0.260 (p25)	65.56	0.92	13.18	0.01	7.784E-05
**157**	med_area_home <17.698 (p54) &den_public_trans >17.842 (p52)	rate_local4 >0.275 (p28)	71.04	0.89	13.15	0.05	5.01E-05
**158**	ave_household_size <2.681 (p29)	rate_local4 >0.823 (p79)	27.71	0.42	13.00	0.20	3.338E-06
**159**	med_income <24270.629 (p79) &prop_publicResid <0.000 (p0) &prop_agricultural <0.012 (p60)	rate_local4 >0.870 (p80)	18.55	0.67	13.00	0.21	3.075E-05
**160**	prop_higher_edu <0.379 (p87) &prop_grassland <0.047 (p61)	rate_local4 >0.282 (p28)	90.36	0.84	12.99	0.03	5.879E-05
**161**	med_area_home <17.698 (p54) &prop_rural_set <0.010 (p59)	rate_local4 >0.274 (p28)	68.29	0.89	12.95	0.09	1.139E-05
**162**	prop_transport >0.072 (p36) &prop_utilities <0.003 (p33)	rate_local4 >0.737 (p74)	23.15	0.62	12.94	0.24	1.544E-05
**163**	prop_over65 >0.114 (p15) &prop_transport >0.268 (p77)	rate_local4 >0.437 (p43)	36.25	0.86	12.84	0.09	2.202E-05
**164**	build_area_pp <58.358 (p47) &prop_rural_set <0.043 (p72)	rate_local4 >0.302 (p29)	65.93	0.87	12.79	0.06	2.328E-05
**165**	prop_industrial <0.000 (p0)	rate_local4 >1.030 (p86)	23.89	0.31	12.74	0.17	2.01E-07
**166**	prop_higher_edu <0.322 (p79) &den_bldg >0.167 (p49)	rate_local4 >0.282 (p28)	68.11	0.88	12.72	0.04	2.493E-05
**167**	prop_over65 >0.114 (p15) &den_bldg >0.337 (p77)	rate_local4 >0.437 (p43)	36.14	0.85	12.63	0.07	7.259E-05
**168**	prop_utilities <0.003 (p33) &prop_agricultural <0.008 (p56)	rate_local4 >0.737 (p74)	21.15	0.66	12.49	0.29	4.188E-06
**169**	med_income <14713.230 (p25) &LU_entropy <0.523 (p40)	rate_local4 >0.824 (p79)	16.81	0.84	12.42	0.47	4.118E-07
**170**	prop_higher_edu <0.356 (p84) &den_public_trans >26.707 (p59)	rate_local4 >0.262 (p25)	65.42	0.91	12.39	0.04	5.906E-05
**171**	den_bldg >0.187 (p53) &prop_utilities <0.003 (p36)	rate_local4 >0.793 (p77)	20.32	0.61	12.29	0.21	2.597E-05
**172**	med_income <24553.043 (p79) &prop_shrubland <0.177 (p77) &POI_den_edu >218.294 (p64)	rate_local4 >0.300 (p29)	51.18	0.92	12.23	0.03	5.311E-06
**173**	den_population >24308.570 (p56) &prop_publicResid <0.006 (p57)	rate_local4 >0.827 (p79)	19.33	0.59	12.19	0.24	5.111E-05
**174**	med_area_home <16.794 (p50) &prop_private_resid >0.153 (p76)	rate_local4 >0.799 (p77)	17.87	0.73	12.15	0.28	5.208E-05
**175**	prop_rural_set <0.005 (p54) &prop_utilities <0.003 (p33)	rate_local4 >0.737 (p74)	20.96	0.64	12.10	0.25	5.319E-05
**176**	den_population >46473.903 (p74) &prop_rural_set <0.047 (p73)	rate_local4 >0.302 (p29)	51.20	0.92	12.08	0.02	4.581E-05
**177**	med_area_home <17.039 (p52) &prop_shrubland <0.277 (p88)	rate_local4 >0.278 (p28)	86.24	0.84	11.89	0.03	8.105E-05
**178**	med_income <23814.728 (p79) &prop_shrubland <0.020 (p36)	rate_local4 >0.289 (p29)	53.79	0.91	11.87	0.08	8.878E-06
**179**	den_population >46473.903 (p74) &prop_rural_set <0.045 (p73) &LU_entropy <0.669 (p85)	rate_local4 >0.302 (p29)	48.67	0.92	11.77	0.01	1.533E-05
**180**	prop_rural_set <0.023 (p65)	rate_local4 >0.534 (p56)	73.40	0.53	11.48	0.08	5.687E-05

**181**	prop_utilities <0.003 (p36) &prop_grassland <0.019 (p45)	rate_local4 >0.843 (p80)	18.12	0.57	11.36	0.20	8.076E-05
**182**	med_income <28905.229 (p84) &prop_private_resid >0.093 (p65) &prop_shrubland <0.149 (p68)	rate_local4 >0.294 (p29)	48.03	0.92	11.10	0.03	5.517E-05
**183**	ave_household_size <2.794 (p37) &prop_gov_insti_faci >0.025 (p36)	rate_local4 >0.565 (p60)	35.12	0.61	11.00	0.11	3.835E-05
**184**	den_population >52299.793 (p79) &build_area_pp <93.205 (p63)	rate_local4 >0.263 (p26)	45.01	0.94	9.93	0.01	7.148E-05
**185**	med_area_home <15.396 (p45)	rate_local4 >0.295 (p29)	72.01	0.81	9.65	0.11	7.299E-05
**186**	POI_den_edu =448.921-1372.584 (p78-94)	rate_local4 >0.751 (p74)	19.67	0.50	9.43	0.24	2.323E-05
**187**	med_area_home <17.327 (p52) &prop_publicResid <0.000 (p0)	rate_local4 >0.903 (p81)	13.61	0.51	8.66	0.26	9.869E-06
**188**	den_public_trans >99.082 (p92)	rate_local4 >0.840 (p79)	11.85	0.66	8.04	0.45	4.248E-07
**189**	prop_household_3gen <0.080 (p92)	rate_local4 >0.224 (p21)	157.09	0.80	7.21	0.04	1.041E-07
**190**	prop_preprim_edu <0.143 (p88)	rate_local4 >0.449 (p44)	108.54	0.58	7.11	0.04	5.375E-05
**191**	POI_den_edu >1372.584 (p94)	rate_local4 >0.751 (p74)	10.59	0.79	7.09	0.53	5.283E-07
**192**	prop_business >0.137 (p93)	rate_local4 >0.874 (p80)	8.82	0.62	6.02	0.42	1.349E-05
**193**	med_income <12583.023 (p7)	rate_local4 >0.847 (p80)	8.69	0.63	5.77	0.42	1.349E-05
**194**	den_public_trans =70.104-99.082 (p86-92)	rate_local4 >0.840 (p79)	8.10	0.67	5.54	0.46	4.749E-05
**195**	prop_business =0.055-0.137 (p86-93)	rate_local4 >0.874 (p80)	8.72	0.48	5.11	0.28	4.451E-05
**196**	gender_ratio <87.319 (p11)	rate_local4 >1.248 (p90)	7.67	0.26	4.68	0.16	7.93E-05
**197**	prop_preprim_edu <0.155 (p91) &LU_entropy >0.544 (p46)	rate_local4 =0.174-0.799 (p16-77)	83.75	0.81	21.42	0.06	1.084E-06
**198**	gender_ratio >87.319 (p11) &prop_preprim_edu <0.146 (p89) &LU_entropy >0.524 (p40)	rate_local4 =0.182-0.915 (p16-81)	86.55	0.84	20.68	0.02	4.368E-05
**199**	prop_preprim_edu <0.155 (p91) &POI_den_sports <650.387 (p75)	rate_local4 =0.099-0.831 (p13-79)	110.07	0.79	19.87	0.05	3.307E-06
**200**	prop_preprim_edu <0.155 (p91) &POI_den_transport <1026.641 (p75)	rate_local4 =0.099-0.831 (p13-79)	109.37	0.79	19.51	0.05	8.209E-06
**201**	prop_preprim_edu <0.155 (p91) &den_road <16.849 (p72)	rate_local4 =0.099-0.831 (p13-79)	107.56	0.79	19.26	0.05	6.61E-06
**202**	prop_preprim_edu <0.155 (p91) &POI_den_telecom_elec <326.218 (p70)	rate_local4 =0.174-0.799 (p16-77)	97.59	0.75	19.26	0.05	1.489E-05
**203**	prop_preprim_edu <0.139 (p86) &POI_den_mall_mkt <265.556 (p77)	rate_local4 =0.087-0.979 (p13-85)	114.62	0.85	19.17	0.07	8.331E-07
**204**	prop_household_3gen <0.080 (p92) &POI_den_sports <650.387 (p75)	rate_local4 =0.099-0.831 (p13-79)	112.45	0.78	18.83	0.04	9.361E-06
**205**	gender_ratio >87.639 (p11) &prop_preprim_edu <0.155 (p91) &den_public_trans <80.171 (p89)	rate_local4 =0.099-0.831 (p13-79)	113.96	0.77	18.48	0.04	7.297E-05

**206**	LU_entropy >0.545 (p46)	rate_local4 =0.183-0.840 (p16-79)	88.77	0.77	17.65	0.15	2.551E-09
**207**	prop_preprim_edu <0.155 (p91) &POI_den_edu <451.677 (p78)	rate_local4 =0.099-0.831 (p13-79)	110.24	0.77	17.39	0.05	1.909E-05
**208**	prop_preprim_edu <0.155 (p91) &ave_household_size >2.699 (p29)	rate_local4 =0.108-0.823 (p13-79)	103.33	0.77	17.19	0.03	6.644E-05
**209**	prop_preprim_edu <0.155 (p91) &den_public_trans <57.487 (p82)	rate_local4 =0.099-0.831 (p13-79)	119.01	0.76	17.18	0.04	7.471E-05
**210**	gender_ratio >87.341 (p11) &LU_entropy >0.510 (p36)	rate_local4 =0.132-0.854 (p14-80)	95.90	0.79	17.17	0.04	2.066E-05
**211**	prop_household_3gen <0.080 (p92) &POI_den_edu <450.512 (p78)	rate_local4 =0.099-0.831 (p13-79)	112.64	0.76	16.51	0.04	3.931E-05
**212**	gender_ratio >87.319 (p11) &ave_household_size >2.693 (p29)	rate_local4 =0.150-0.840 (p15-79)	97.65	0.75	16.43	0.04	5.746E-05
**213**	prop_preprim_edu <0.155 (p91) &den_bldg <0.401 (p82)	rate_local4 =0.099-0.831 (p13-79)	116.85	0.74	15.50	0.04	8.169E-05
**214**	prop_preprim_edu <0.126 (p82) &prop_business <0.038 (p84)	rate_local4 =0.087-0.979 (p13-85)	114.27	0.81	15.33	0.06	1.502E-05
**215**	gender_ratio >87.236 (p11) &den_public_trans <68.524 (p86)	rate_local4 =0.127-0.819 (p14-78)	117.02	0.73	15.28	0.04	2.348E-05
**216**	gender_ratio >87.511 (p11) &den_road <25.616 (p91)	rate_local4 =0.123-0.723 (p14-72)	111.68	0.67	15.26	0.04	3.822E-05
**217**	ave_household_size >2.681 (p29)	rate_local4 =0.108-0.823 (p13-79)	108.28	0.73	13.76	0.09	6.903E-05
**218**	build_area_pp <154.967 (p85)	rate_local4 =0.254-1.043 (p24-86)	121.92	0.68	12.49	0.07	1.507E-06
**219**	den_road =9.806-16.034 (p46-70)	rate_local4 =0.157-0.759 (p15-74)	41.65	0.81	11.52	0.22	6.043E-05
**220**	prop_publicResid >0.054 (p72)	rate_local4 =0.248-1.078 (p23-87)	47.53	0.81	10.54	0.18	2.482E-05
**221**	prop_transport =0.104-0.224 (p44-69)	rate_local4 =0.174-0.799 (p16-77)	40.99	0.79	9.72	0.19	6.883E-05
**222**	den_bldg =0.135-0.240 (p42-64)	rate_local4 =0.186-0.681 (p16-70)	34.08	0.73	9.65	0.21	4.157E-05
**223**	den_public_trans >16.405 (p50) &LU_entropy >0.625 (p67)	rate_local4 =0.282-0.790 (p28-77)	22.41	0.66	5.94	0.11	5.73E-05
**224**	den_public_trans <44.105 (p77) &POI_den_mall_mkt <249.510 (p76)	rate_local4 <0.720 (p71)	124.37	0.87	22.13	0.04	1.753E-05
**225**	den_public_trans <40.100 (p73) &POI_den_sports <807.470 (p78)	rate_local4 <0.711 (p71)	120.16	0.87	21.85	0.05	2.101E-06
**226**	den_road <17.821 (p75) &den_public_trans <51.003 (p79)	rate_local4 <0.698 (p70)	126.17	0.85	21.85	0.03	5.487E-06
**227**	den_public_trans <46.718 (p78) &POI_den_telecom_elec <494.553 (p78)	rate_local4 <0.711 (p71)	125.44	0.86	21.83	0.05	9.692E-06
**228**	den_public_trans <40.100 (p73) &POI_den_transport <1274.302 (p77)	rate_local4 <0.711 (p71)	120.28	0.87	21.81	0.05	7.691E-06
**229**	den_public_trans <49.010 (p79) &POI_den_edu <598.811 (p80)	rate_local4 <0.735 (p74)	131.57	0.87	21.49	0.05	2.107E-05
**230**	den_bldg <0.312 (p75) &den_public_trans <49.010 (p79)	rate_local4 <0.715 (p71)	124.37	0.85	20.42	0.04	3.894E-05
**231**	den_bldg <0.313 (p75) &POI_den_edu <448.921 (p78)	rate_local4 <0.753 (p74)	126.52	0.88	19.83	0.03	6.557E-05

**232**	den_road <18.030 (p76) &prop_business <0.063 (p87)	rate_local4 <0.698 (p70)	128.89	0.83	19.75	0.01	2.157E-05
**233**	prop_business <0.063 (p87) &POI_den_edu <506.706 (p79)	rate_local4 <0.784 (p76)	139.11	0.88	19.54	0.03	4.939E-07
**234**	den_bldg <0.330 (p77) &POI_den_sports <958.635 (p81)	rate_local4 <0.766 (p74)	132.80	0.88	19.53	0.03	5.38E-05
**235**	POI_den_transport <904.959 (p71)	rate_local4 <0.726 (p73)	129.24	0.85	19.32	0.13	1.954E-14
**236**	POI_den_sports <598.644 (p72)	rate_local4 <0.751 (p74)	132.66	0.86	19.25	0.12	9.183E-12
**237**	POI_den_mall_mkt <208.752 (p73)	rate_local4 <0.753 (p74)	132.99	0.86	19.24	0.12	1.821E-11
**238**	den_bldg <0.330 (p77) &POI_den_transport <1601.585 (p81)	rate_local4 <0.766 (p74)	132.28	0.88	19.22	0.04	4.932E-05
**239**	den_population <35216.647 (p66) &den_public_trans <40.100 (p73)	rate_local4 <0.715 (p71)	108.59	0.87	19.22	0.05	7.172E-05
**240**	den_bldg <0.330 (p77) &den_road <20.312 (p81)	rate_local4 <0.766 (p74)	134.97	0.87	18.97	0.03	6.657E-05
**241**	POI_den_telecom_elec <374.340 (p72)	rate_local4 <0.741 (p74)	131.51	0.85	18.85	0.12	9.927E-12
**242**	den_bldg <0.312 (p75) &POI_den_mall_mkt <474.871 (p86)	rate_local4 <0.767 (p74)	132.92	0.87	18.79	0.03	4.724E-06
**243**	den_bldg <0.341 (p77) &POI_den_telecom_elec <755.639 (p85)	rate_local4 <0.758 (p74)	134.71	0.86	18.75	0.03	5.8E-06
**244**	POI_den_sports <820.348 (p78) &POI_den_edu <442.307 (p78)	rate_local4 <0.755 (p74)	134.82	0.86	18.42	0.01	3.309E-05
**245**	prop_business <0.063 (p87) &POI_den_sports <1059.151 (p82)	rate_local4 <0.784 (p76)	141.87	0.87	18.21	0.03	4.824E-06
**246**	prop_business <0.051 (p86) &POI_den_transport <1601.585 (p81)	rate_local4 <0.766 (p74)	136.33	0.86	17.94	0.02	5.973E-05
**247**	den_population <36883.552 (p68) &prop_business <0.065 (p87)	rate_local4 <0.736 (p74)	113.45	0.86	17.86	0.04	2.739E-06
**248**	prop_business <0.057 (p86) &POI_den_telecom_elec <653.258 (p82)	rate_local4 <0.825 (p79)	143.85	0.89	17.44	0.03	4.72E-05
**249**	den_population <7339.493 (p37)	rate_local4 <0.177 (p16)	30.40	0.37	17.43	0.21	2.35E-11
**250**	den_road <25.616 (p91) &POI_den_edu <396.631 (p76)	rate_local4 <0.835 (p79)	138.92	0.89	16.88	0.01	3.239E-05
**251**	POI_den_edu <448.921 (p78)	rate_local4 <0.751 (p74)	135.83	0.84	16.60	0.10	3.487E-11
**252**	POI_den_edu <448.921 (p78) &POI_den_mall_mkt <311.791 (p78)	rate_local4 <0.725 (p73)	124.79	0.79	15.88	0.01	4.992E-05
**253**	prop_shrubland >0.064 (p49)	rate_local4 <0.777 (p76)	99.56	0.90	15.80	0.14	7.92E-07
**254**	prop_private_resid <0.114 (p69) &prop_publicResid <0.033 (p69)	rate_local4 <0.183 (p16)	30.49	0.33	15.53	0.10	2.403E-05
**255**	ave_household_size >2.492 (p11) &POI_den_transport <1730.985 (p83)	rate_local4 <0.884 (p81)	144.02	0.89	14.97	0.02	3.175E-05
**256**	prop_woodland >0.097 (p46)	rate_local4 <0.786 (p77)	103.57	0.89	14.94	0.13	2.724E-07
**257**	prop_publicResid <0.056 (p73) &prop_business <0.008 (p59)	rate_local4 <0.720 (p71)	86.79	0.87	14.90	0.06	1.305E-05

**258**	prop_grassland >0.040 (p59)	rate_local4 <0.517 (p54)	64.87	0.69	14.64	0.16	2.082E-05
**259**	prop_private_resid <0.091 (p64)	rate_local4 <0.753 (p74)	114.54	0.84	14.17	0.10	1.071E-05
**260**	prop_agricultural >0.021 (p67)	rate_local4 <0.416 (p42)	43.29	0.60	13.19	0.18	4.033E-05
**261**	den_bldg <0.135 (p42)	rate_local4 <0.186 (p16)	27.77	0.31	12.92	0.14	2.104E-07
**262**	prop_private_resid <0.156 (p76) &POI_den_edu <972.143 (p91)	rate_local4 <0.957 (p83)	141.44	0.91	12.84	0.02	7.996E-05
**263**	den_public_trans <4.829 (p30)	rate_local4 <0.183 (p16)	22.78	0.36	12.65	0.20	2.702E-08
**264**	gender_ratio >87.511 (p11) &den_bldg <0.299 (p74) &den_road <25.616 (p91)	rate_local4 <1.127 (p88)	130.78	0.97	12.20	0.00	4.271E-05
**265**	prop_transport <0.104 (p44)	rate_local4 <0.174 (p16)	27.43	0.29	11.96	0.13	1.188E-06
**266**	den_road <9.806 (p46)	rate_local4 <0.157 (p15)	26.95	0.27	11.89	0.12	6.292E-06
**267**	prop_rural_set >0.023 (p65)	rate_local4 <0.534 (p56)	53.70	0.70	11.73	0.15	5.687E-05
**268**	prop_gov_insti_faci <0.025 (p36)	rate_local4 <0.050 (p13)	21.12	0.28	11.13	0.15	2.825E-06
**269**	prop_business <0.055 (p86)	rate_local4 <0.874 (p80)	157.87	0.86	11.10	0.06	6.039E-10
**270**	gender_ratio >87.659 (p11) &prop_business <0.024 (p79)	rate_local4 <1.153 (p89)	138.19	0.95	10.34	0.02	7.725E-05
**271**	med_area_home >15.396 (p45)	rate_local4 <0.295 (p29)	46.19	0.37	9.64	0.08	7.299E-05
**272**	build_area_pp >154.967 (p85)	rate_local4 <0.254 (p24)	18.13	0.51	9.60	0.27	6.704E-06
**273**	prop_preprim_edu >0.143 (p88)	rate_local4 <0.170 (p16)	13.75	0.52	9.51	0.36	1.615E-08
**274**	prop_higher_edu <0.167 (p35) &med_area_home >15.171 (p43)	rate_local4 <0.423 (p42)	22.51	0.74	9.49	0.24	5.538E-05
**275**	gender_ratio >87.527 (p11) &POI_den_transport <2117.352 (p88)	rate_local4 <1.358 (p91)	158.44	0.97	9.44	0.01	3.657E-05
**276**	gender_ratio >87.319 (p11) &prop_private_resid <0.172 (p80)	rate_local4 <1.248 (p90)	141.62	0.96	9.39	0.02	5.141E-05
**277**	prop_publicResid <0.054 (p72)	rate_local4 <0.248 (p23)	45.96	0.30	9.39	0.06	2.787E-05
**278**	prop_open_recreation <0.006 (p25)	rate_local4 <0.037 (p13)	16.19	0.31	9.31	0.18	2.281E-06
**279**	prop_higher_edu <0.167 (p35) &build_area_pp >69.457 (p52)	rate_local4 <0.423 (p42)	20.64	0.74	8.69	0.24	7.052E-05
**280**	gender_ratio >87.623 (p11) &prop_woodland >0.069 (p37)	rate_local4 <1.358 (p91)	117.54	0.98	8.62	0.01	7.927E-05
**281**	prop_household_3gen >0.080 (p92)	rate_local4 <0.224 (p21)	11.57	0.63	7.38	0.40	1.041E-07
**282**	den_bldg <0.104 (p35) &prop_shrubland <0.145 (p67)	rate_local4 <0.391 (p39)	13.44	0.70	5.89	0.17	4.552E-05
**283**	gender_ratio <87.871 (p11) &den_population <8952.749 (p40)	rate_local4 <0.040 (p13)	6.73	0.86	5.71	0.53	8.126E-05
**284**	med_income <15000.000 (p27) &med_area_home >15.497 (p45)	rate_local4 <0.050 (p13)	6.71	0.44	4.72	0.25	7.584E-06
**285**	gender_ratio <86.955 (p11) &prop_grassland >0.066 (p66)	rate_local4 <0.039 (p13)	5.47	0.94	4.70	0.68	1.044E-05
**286**	gender_ratio >87.319 (p11)	rate_local4 <1.248 (p90)	171.58	0.92	4.55	0.02	7.93E-05
**287**	gender_ratio <86.981 (p11) &den_bldg <0.112 (p37)	rate_local4 <0.141 (p14)	5.11	1.00	4.37	0.71	7.005E-05

(j) Rules for median waiting period; POI accessibility was used

No.	Antecedent	Consequent	Supp	Conf	Lev	Imp	*P*
**1**	med_area_home >18.602 (p52)	med_waiting_period >4.392 (p67)	31.27	0.38	10.07	0.12	0.000202
**2**	med_income >19680.813 (p60)	med_waiting_period >5.030 (p82)	17.80	0.27	7.39	0.11	0.000202
**3**	build_area_pp >57.721 (p46)	med_waiting_period >5.317 (p82)	19.22	0.18	6.54	0.06	0.0002
**4**	den_public_trans <3.053 (p17)	med_waiting_period >5.251 (p82)	10.79	0.29	6.50	0.18	0.000137
**5**	gender_ratio >102.032 (p82)	med_waiting_period >5.276 (p82)	8.70	0.35	5.85	0.24	1.18E-05
**6**	prop_open_recreation <0.008 (p24)	med_waiting_period >5.544 (p83)	10.99	0.22	5.64	0.11	0.000221
**7**	prop_preprim_edu <0.065 (p7)	med_waiting_period >5.076 (p82)	6.58	0.47	4.52	0.32	0.000154
**8**	prop_household_3gen >0.060 (p82) &den_population =11418.151-37853.952 (p35-61)	med_waiting_period >5.250 (p82)	4.79	0.67	3.92	0.41	0.000196
**9**	gender_ratio <88.232 (p12) &prop_transport <0.109 (p40)	med_waiting_period >5.669 (p83)	2.73	0.61	2.27	0.45	0.000177
**10**	gender_ratio <102.188 (p82) &build_area_pp <137.537 (p77)	med_waiting_period =3.497-5.221 (p26-82)	104.86	0.70	18.16	0.05	9.99E-05
**11**	gender_ratio <100.812 (p79) &prop_higher_edu <0.300 (p70) &build_area_pp <148.430 (p79)	med_waiting_period =2.993-5.300 (p5-82)	102.09	0.85	17.17	0.04	1.24E-05
**12**	gender_ratio <102.255 (p82) &med_income <21279.411 (p70) &build_area_pp <160.924 (p81)	med_waiting_period =2.965-5.099 (p5-82)	103.25	0.83	17.16	0.03	4.5E-06
**13**	gender_ratio <102.242 (p82) &med_area_home <20.171 (p63)	med_waiting_period =3.663-5.258 (p30-82)	83.75	0.69	16.91	0.05	0.000159
**14**	med_area_home <17.835 (p51) &build_area_pp <162.989 (p81)	med_waiting_period =2.949-5.253 (p5-82)	91.84	0.89	16.85	0.01	4.15E-06
**15**	med_area_home <18.602 (p52)	med_waiting_period =2.769-4.392 (p5-67)	95.05	0.82	15.51	0.13	1.34E-07
**16**	den_population >41209.065 (p63)	med_waiting_period =3.734-4.292 (p30-67)	40.95	0.64	15.41	0.24	1.48E-07
**17**	gender_ratio <101.525 (p81) &den_population >38042.584 (p61)	med_waiting_period =3.753-5.345 (p30-82)	51.39	0.79	15.39	0.03	5.99E-05
**18**	med_income <20988.125 (p68) &med_area_home <18.979 (p54) &build_area_pp <137.247 (p77)	med_waiting_period =2.987-5.260 (p5-82)	88.90	0.86	15.37	0.01	5.91E-05
**19**	gender_ratio <102.307 (p82) &med_area_home <20.874 (p65) &build_area_pp <162.781 (p81)	med_waiting_period =2.976-5.655 (p5-83)	102.55	0.86	15.32	0.02	0.00016
**20**	build_area_pp <57.721 (p46)	med_waiting_period =3.840-5.317 (p30-82)	67.27	0.72	15.23	0.16	2.18E-06
**21**	med_income <20841.767 (p68) &build_area_pp <155.834 (p81)	med_waiting_period =2.935-4.660 (p5-72)	97.48	0.74	15.04	0.04	0.000236

**22**	prop_higher_edu <0.365 (p79) &med_income <21006.819 (p69) &build_area_pp <163.106 (p81)	med_waiting_period =2.985-5.123 (p5-82)	106.06	0.80	14.99	0.00	0.000104
**23**	gender_ratio <100.600 (p79) &med_income <30435.197 (p80)	med_waiting_period =3.425-5.176 (p26-82)	98.23	0.68	14.65	0.05	1.23E-05
**24**	prop_higher_edu <0.297 (p69) &build_area_pp <150.096 (p79)	med_waiting_period =2.993-5.300 (p5-82)	108.28	0.82	14.42	0.05	6.06E-06
**25**	gender_ratio <101.408 (p80) &prop_household_3gen <0.064 (p84) &build_area_pp <179.586 (p83)	med_waiting_period =2.944-5.225 (p5-82)	117.69	0.83	14.40	0.02	2.27E-05
**26**	med_area_home <20.679 (p64) &den_population >15042.218 (p39) &build_area_pp <143.553 (p78)	med_waiting_period =2.998-5.244 (p5-82)	81.12	0.86	14.37	0.03	1.18E-05
**27**	prop_preprim_edu <0.143 (p84) &med_income <18372.979 (p56)	med_waiting_period =2.965-5.190 (p5-82)	86.88	0.85	14.16	0.04	4.89E-05
**28**	med_income <22638.607 (p72) &den_population >15042.218 (p39) &build_area_pp <143.553 (p78)	med_waiting_period =2.998-5.244 (p5-82)	78.33	0.87	14.10	0.02	0.000117
**29**	med_income <20507.105 (p68) &med_area_home <19.618 (p58)	med_waiting_period =3.000-5.137 (p5-82)	90.67	0.81	13.96	0.03	9.72E-05
**30**	gender_ratio <97.560 (p72) &prop_preprim_edu <0.131 (p79) &med_income <18372.979 (p56)	med_waiting_period =2.993-5.300 (p5-82)	73.77	0.88	13.86	0.02	2.55E-05
**31**	gender_ratio <97.711 (p72) &med_income <18919.315 (p57) &med_area_home <21.437 (p70)	med_waiting_period =2.948-5.371 (p5-82)	84.60	0.87	13.49	0.01	0.000209
**32**	gender_ratio <105.121 (p85) &prop_preprim_edu <0.129 (p79) &prop_higher_edu <0.285 (p67)	med_waiting_period =2.931-5.353 (p5-82)	94.36	0.86	13.44	0.01	0.000183
**33**	med_income <20696.281 (p68) &den_population >15042.218 (p39)	med_waiting_period =2.979-5.242 (p5-82)	79.30	0.86	13.35	0.07	4.1E-07
**34**	gender_ratio <103.325 (p83) &med_income <22645.700 (p72) &den_population >15364.577 (p39)	med_waiting_period =2.969-5.368 (p5-82)	80.42	0.87	13.31	0.01	0.0002
**35**	prop_preprim_edu <0.128 (p78) &prop_higher_edu <0.365 (p79) &med_income <21006.819 (p69)	med_waiting_period =2.823-5.112 (p5-82)	104.79	0.91	13.19	0.00	4.6E-05
**36**	med_income <21279.411 (p70) &build_area_pp <158.852 (p81) &prop_open_recreation >0.027 (p41)	med_waiting_period =2.965-5.099 (p5-82)	70.18	0.86	13.11	0.03	0.000229
**37**	gender_ratio <104.531 (p83) &med_income <20891.100 (p68) &prop_grassland <0.039 (p58)	med_waiting_period =2.562-5.081 (p5-82)	80.61	0.94	13.09	0.01	0.000242
**38**	prop_higher_edu <0.275 (p64) &prop_open_recreation >0.027 (p41)	med_waiting_period =3.840-5.317 (p30-82)	55.60	0.72	12.84	0.10	0.000122
**39**	med_income <20729.622 (p68) &build_area_pp <164.775 (p81) &prop_agricultural <0.012 (p58)	med_waiting_period =2.949-5.253 (p5-82)	70.48	0.89	12.81	0.03	0.000235

**40**	gender_ratio <100.733 (p79) &prop_household_3gen <0.039 (p61)	med_waiting_period =2.744-5.150 (p5-82)	98.59	0.92	12.80	0.06	2.25E-06
**41**	med_income <20891.100 (p68) &prop_grassland <0.039 (p58)	med_waiting_period =2.562-5.081 (p5-82)	82.15	0.93	12.78	0.08	2.55E-05
**42**	prop_higher_edu <0.315 (p71) &den_population >16609.467 (p41) &build_area_pp <160.647 (p81)	med_waiting_period =2.874-5.368 (p5-82)	78.94	0.92	12.73	0.01	0.000173
**43**	med_area_home <20.679 (p64) &den_population >15042.218 (p39)	med_waiting_period =2.998-5.244 (p5-82)	83.43	0.84	12.73	0.05	2.28E-05
**44**	prop_higher_edu <0.355 (p78) &med_income <21006.819 (p69) &den_road >5.569 (p21)	med_waiting_period =2.654-5.020 (p5-82)	98.14	0.89	12.72	0.01	1.19E-05
**45**	prop_preprim_edu <0.130 (p79) &prop_higher_edu <0.300 (p70)	med_waiting_period =2.993-5.300 (p5-82)	97.37	0.82	12.67	0.05	3.8E-05
**46**	gender_ratio <98.273 (p73) &prop_higher_edu <0.348 (p77) &med_income <21698.571 (p70)	med_waiting_period =2.993-5.328 (p5-82)	97.73	0.82	12.59	0.00	0.00015
**47**	prop_preprim_edu >0.076 (p14) &den_population >14982.236 (p39) &build_area_pp <139.950 (p78)	med_waiting_period =2.957-5.374 (p5-82)	80.92	0.86	12.56	0.02	4.73E-05
**48**	gender_ratio <97.517 (p72) &med_area_home <20.101 (p63) &den_population >15060.055 (p39)	med_waiting_period =3.000-5.504 (p5-83)	74.66	0.86	12.55	0.02	0.000133
**49**	prop_higher_edu <0.365 (p79) &med_income <21006.819 (p69) &prop_grassland <0.106 (p71)	med_waiting_period =2.823-5.112 (p5-82)	99.33	0.91	12.54	0.00	0.000222
**50**	gender_ratio <98.716 (p74) &prop_household_3gen <0.051 (p79) &med_area_home <20.922 (p65)	med_waiting_period =2.773-5.116 (p5-82)	97.08	0.91	12.49	0.02	0.000165
**51**	prop_higher_edu <0.316 (p72) &den_population >15042.218 (p39)	med_waiting_period =2.998-5.244 (p5-82)	77.34	0.85	12.47	0.06	6.31E-05
**52**	gender_ratio <101.408 (p80) &prop_household_3gen <0.064 (p84) &den_population >14982.236 (p39)	med_waiting_period =2.944-5.225 (p5-82)	86.74	0.85	12.40	0.02	0.000138
**53**	prop_higher_edu <0.344 (p76) &prop_household_3gen <0.064 (p84) &den_population >14982.236 (p39)	med_waiting_period =2.944-5.225 (p5-82)	77.95	0.86	12.34	0.01	6.53E-05
**54**	med_income <21006.819 (p69) &prop_transport >0.112 (p41)	med_waiting_period =2.930-5.163 (p5-82)	73.13	0.87	12.33	0.08	3.16E-05
**55**	med_income <21006.819 (p69) &den_road >5.569 (p21)	med_waiting_period =2.654-5.020 (p5-82)	99.04	0.88	12.31	0.05	7.79E-06
**56**	prop_higher_edu <0.340 (p75) &med_income <22901.777 (p72) &den_population >15847.175 (p40)	med_waiting_period =2.542-5.535 (p5-83)	87.86	0.96	12.07	0.01	0.000129
**57**	gender_ratio <98.663 (p74) &prop_higher_edu <0.305 (p70)	med_waiting_period =2.993-5.300 (p5-82)	99.14	0.81	12.06	0.04	9.67E-05

**58**	prop_higher_edu <0.355 (p78) &med_area_home <20.923 (p65) &den_road >5.569 (p21)	med_waiting_period =2.654-5.020 (p5-82)	96.31	0.88	11.91	0.02	9.05E-05
**59**	gender_ratio <105.315 (p85) &den_population >19560.599 (p43) &build_area_pp <122.002 (p73)	med_waiting_period =2.921-5.465 (p5-82)	84.91	0.89	11.84	0.01	0.00016
**60**	den_population >17331.400 (p42) &build_area_pp <143.553 (p78)	med_waiting_period =2.984-5.370 (p5-82)	85.16	0.83	11.76	0.03	0.000159
**61**	med_income <21279.411 (p70) &prop_open_recreation >0.027 (p42)	med_waiting_period =2.989-5.416 (p5-82)	74.09	0.85	11.74	0.08	0.000106
**62**	med_income <21163.180 (p69) &prop_agricultural <0.012 (p58)	med_waiting_period =2.949-5.253 (p5-82)	75.09	0.86	11.70	0.08	5.71E-05
**63**	prop_higher_edu <0.289 (p67)	med_waiting_period =3.284-4.158 (p26-67)	75.30	0.52	11.62	0.08	2.98E-05
**64**	med_income <22670.905 (p72) &build_area_pp <121.123 (p72) &prop_rural_set <0.009 (p55)	med_waiting_period =2.977-5.261 (p5-82)	61.22	0.88	11.61	0.05	0.000215
**65**	prop_preprim_edu >0.076 (p14) &den_population >14982.236 (p39)	med_waiting_period =2.957-5.374 (p5-82)	84.77	0.85	11.59	0.04	2.48E-05
**66**	med_income <21006.819 (p69) &den_public_trans >17.695 (p45)	med_waiting_period =2.771-5.133 (p5-82)	78.65	0.93	11.52	0.07	2.38E-05
**67**	prop_higher_edu <0.353 (p78) &med_income <22901.777 (p72) &prop_transport >0.097 (p35)	med_waiting_period =2.585-5.004 (p5-82)	82.05	0.90	11.45	0.00	8.34E-05
**68**	gender_ratio <95.004 (p61) &med_income <21309.129 (p70) &prop_household_3gen <0.042 (p67)	med_waiting_period =2.663-5.044 (p5-82)	70.65	0.92	11.14	0.04	0.000169
**69**	med_income <19680.813 (p60)	med_waiting_period =2.999-5.030 (p5-82)	97.53	0.75	10.99	0.08	0.000144
**70**	prop_higher_edu <0.312 (p71) &prop_transport >0.106 (p38)	med_waiting_period =2.979-5.225 (p5-82)	72.25	0.84	10.76	0.07	0.000106
**71**	gender_ratio <97.609 (p72) &prop_higher_edu <0.315 (p71) &den_population >16609.467 (p41)	med_waiting_period =2.808-5.534 (p5-83)	74.96	0.98	10.68	0.01	6.17E-05
**72**	prop_higher_edu <0.302 (p70) &prop_grassland <0.063 (p63)	med_waiting_period =2.941-5.368 (p5-82)	81.40	0.85	10.63	0.06	9.24E-05
**73**	med_area_home <20.923 (p65) &den_road >5.569 (p21)	med_waiting_period =2.654-5.020 (p5-82)	101.66	0.86	10.53	0.03	0.000121
**74**	med_income <19701.735 (p60) &prop_publicResid >0.033 (p61)	med_waiting_period =2.995-5.251 (p5-82)	57.49	0.87	10.47	0.03	0.000247
**75**	prop_preprim_edu >0.075 (p14) &den_public_trans >17.478 (p45)	med_waiting_period =2.737-5.211 (p5-82)	83.88	0.91	10.44	0.05	1.13E-05

**76**	med_area_home <21.618 (p71) &prop_transport >0.096 (p34)	med_waiting_period =2.974-5.233 (p5-82)	82.57	0.82	10.36	0.05	0.00022
**77**	prop_higher_edu <0.285 (p67) &prop_publicResid >0.033 (p61)	med_waiting_period =2.931-5.353 (p5-82)	59.24	0.90	10.27	0.03	5.38E-05
**78**	prop_household_3gen <0.038 (p61) &build_area_pp <173.428 (p82)	med_waiting_period =2.747-5.137 (p5-82)	102.41	0.89	10.13	0.03	0.000232
**79**	prop_higher_edu <0.305 (p70) &den_road >5.864 (p22)	med_waiting_period =2.648-5.044 (p5-82)	94.77	0.87	10.13	0.05	6.56E-05
**80**	gender_ratio <96.126 (p67) &prop_preprim_edu >0.078 (p15) &prop_household_3gen <0.037 (p56)	med_waiting_period =2.931-5.353 (p5-82)	61.42	0.89	10.07	0.02	0.000227
**81**	prop_preprim_edu >0.075 (p14) &den_public_trans >17.478 (p45) &build_area_pp <115.172 (p71)	med_waiting_period =2.737-5.211 (p5-82)	72.96	0.93	10.06	0.01	7.18E-05
**82**	med_area_home <20.754 (p64) &prop_publicResid >0.034 (p62)	med_waiting_period =2.857-5.380 (p5-82)	63.15	0.93	9.85	0.02	0.000196
**83**	prop_preprim_edu >0.077 (p15) &prop_publicResid >0.033 (p61)	med_waiting_period =2.931-5.353 (p5-82)	58.66	0.89	9.71	0.02	6.06E-05
**84**	prop_preprim_edu >0.077 (p15) &prop_agricultural <0.012 (p58)	med_waiting_period =2.793-5.327 (p5-82)	90.42	0.91	9.66	0.07	0.000201
**85**	prop_preprim_edu >0.079 (p15) &prop_transport >0.079 (p30)	med_waiting_period =2.731-5.219 (p5-82)	94.75	0.89	9.64	0.06	0.000196
**86**	gender_ratio <102.032 (p82)	med_waiting_period =3.525-5.276 (p30-82)	109.15	0.63	9.18	0.05	8.93E-05
**87**	prop_publicResid >0.039 (p62)	med_waiting_period =2.992-5.317 (p5-82)	56.16	0.85	8.95	0.14	6.52E-05
**88**	prop_preprim_edu >0.089 (p31) &prop_grassland <0.060 (p62)	med_waiting_period =2.744-5.004 (p5-82)	70.15	0.86	7.50	0.07	0.000123
**89**	prop_household_3gen >0.057 (p81)	med_waiting_period =5.125-6.524 (p82-87)	7.04	0.29	3.87	0.16	9.16E-06
**90**	den_population =8798.848-38778.099 (p32-62) &den_public_trans >27.062 (p52)	med_waiting_period <3.731 (p30)	15.80	0.59	7.06	0.18	8.27E-05
**91**	med_area_home >18.602 (p52)	med_waiting_period <2.769 (p5)	10.67	0.13	5.36	0.06	0.000213

(k) Rules for average waiting period; POI accessibility was used

No.	Antecedent	Consequent	Supp	Conf	Lev	Imp	*P*
**1**	POI_pp_transport >39.683 (p60)	ave_waiting_period >5.790 (p75)	22.98	0.34	9.81	0.14	6.655E-05
**2**	POI_pp_telecom_elec >15.395 (p57)	ave_waiting_period >5.765 (p74)	24.76	0.33	9.65	0.13	7.16E-05
**3**	POI_pp_mall_mkt >8.676 (p64)	ave_waiting_period >5.822 (p75)	20.26	0.33	8.90	0.14	0.0001032
**4**	med_area_home >17.644 (p50)	ave_waiting_period >5.807 (p75)	26.33	0.29	8.89	0.10	1.144E-05
**5**	med_income >19979.413 (p60)	ave_waiting_period >5.751 (p74)	21.18	0.33	8.39	0.13	9.479E-05
**6**	POI_pp_sports >29.352 (p65)	ave_waiting_period >6.163 (p81)	16.16	0.27	8.26	0.14	1.534E-07
**7**	POI_pp_edu >14.015 (p63)	ave_waiting_period >5.788 (p75)	20.68	0.31	7.75	0.12	0.0001774
**8**	prop_over65 <0.099 (p7)	ave_waiting_period >5.508 (p69)	10.2	0.71	6.6	0.46	5.042E-05
**9**	den_public_trans <2.671 (p16)	ave_waiting_period >6.179 (p81)	11.0	0.31	6.6	0.19	0.0001147
**10**	prop_higher_edu >0.351 (p78)	ave_waiting_period >5.750 (p74)	12.9	0.39	6.2	0.18	0.0001866
**11**	prop_over65 <0.114 (p14) &prop_gov_insti_faci <0.027 (p31)	ave_waiting_period >5.506 (p69)	9.4	0.70	6.0	0.25	0.0001759
**12**	prop_higher_edu >0.344 (p75) &prop_publicResid =0.009-0.072 (p53-69)	ave_waiting_period >5.720 (p74)	7.3	0.96	5.7	0.55	0.0001092
**13**	gender_ratio >102.339 (p82)	ave_waiting_period >5.720 (p74)	10.2	0.43	5.3	0.22	0.0002224
**14**	med_income >21927.588 (p70) &prop_publicResid =0.010-0.078 (p53-70)	ave_waiting_period >5.899 (p77)	5.2	0.69	3.9	0.40	0.0001353
**15**	med_area_home >22.579 (p75) &prop_publicResid =0.011-0.091 (p54-73)	ave_waiting_period >5.895 (p77)	4.0	1.00	3.3	0.76	0.0001875
**16**	prop_preprim_edu <0.132 (p79) &med_area_home <18.769 (p53) &POI_pp_mall_mkt <10.185 (p70)	ave_waiting_period =4.142-5.588 (p24-71)	67.4	0.76	23.1	0.05	7.493E-05
**17**	gender_ratio <101.246 (p80) &med_area_home <18.533 (p52) &POI_pp_mall_mkt <10.746 (p72)	ave_waiting_period =4.082-5.672 (p22-72)	74.3	0.78	23.0	0.03	0.0002124
**18**	gender_ratio <101.246 (p80) &med_area_home <18.381 (p51) &POI_pp_telecom_elec <18.589 (p65)	ave_waiting_period =3.754-5.828 (p16-75)	83.1	0.88	23.0	0.02	1.404E-05
**19**	build_area_pp <76.920 (p54) &POI_pp_transport <27.446 (p50)	ave_waiting_period =4.522-6.179 (p40-81)	59.0	0.72	22.8	0.10	3.021E-05
**20**	med_area_home <19.444 (p57) &POI_pp_telecom_elec <15.554 (p57)	ave_waiting_period =4.116-5.839 (p23-76)	76.6	0.80	22.6	0.09	1.939E-05
**21**	gender_ratio <101.187 (p80) &med_area_home <18.325 (p51) &POI_pp_transport <49.039 (p69)	ave_waiting_period =3.801-5.674 (p16-72)	79.3	0.83	22.1	0.02	0.0001352
**22**	med_area_home <18.586 (p52) &build_area_pp <85.357 (p57)	ave_waiting_period =4.097-5.822 (p22-75)	73.6	0.81	22.1	0.07	0.0001032
**23**	med_area_home <18.586 (p52) &POI_pp_sports <29.583 (p65)	ave_waiting_period =4.097-5.822 (p22-75)	76.7	0.80	22.0	0.06	0.0001595

**24**	med_area_home <19.338 (p57) &POI_pp_transport <35.640 (p56)	ave_waiting_period =4.146-5.838 (p24-76)	73.4	0.79	21.9	0.09	4.352E-05
**25**	gender_ratio <101.136 (p80) &prop_preprim_edu <0.131 (p79) &POI_pp_sports <26.199 (p63)	ave_waiting_period =3.627-5.895 (p14-77)	98.6	0.87	21.8	0.02	0.0001209
**26**	build_area_pp <76.052 (p53) &POI_pp_mall_mkt <5.418 (p50)	ave_waiting_period =4.522-6.179 (p40-81)	57.8	0.71	21.8	0.11	9.808E-06
**27**	gender_ratio <101.246 (p80) &med_area_home <19.086 (p56)	ave_waiting_period =3.754-5.828 (p16-75)	92.1	0.83	21.8	0.04	7.792E-07
**28**	prop_over65 <0.184 (p69) &gender_ratio <98.915 (p74) &med_area_home <19.448 (p57)	ave_waiting_period =4.001-5.761 (p22-74)	65.4	0.86	21.7	0.07	4.203E-06
**29**	prop_preprim_edu <0.124 (p77) &prop_higher_edu <0.360 (p78) &POI_pp_telecom_elec <19.306 (p65)	ave_waiting_period =4.083-5.914 (p22-77)	85.7	0.78	21.5	0.04	5.302E-05
**30**	prop_preprim_edu <0.136 (p81) &med_income <19568.062 (p60) &POI_pp_telecom_elec <19.371 (p65)	ave_waiting_period =4.119-5.872 (p23-76)	75.3	0.80	21.2	0.04	0.0001229
**31**	prop_preprim_edu <0.131 (p79) &med_area_home <18.042 (p51) &POI_pp_sports <26.199 (p63)	ave_waiting_period =4.158-5.591 (p25-71)	61.5	0.75	21.1	0.04	0.0002291
**32**	gender_ratio <101.698 (p81) &prop_higher_edu <0.355 (p78) &med_area_home <19.914 (p61)	ave_waiting_period =3.825-5.877 (p16-77)	93.4	0.82	21.0	0.01	8.77E-05
**33**	build_area_pp <70.173 (p52) &POI_pp_edu <9.364 (p50)	ave_waiting_period =4.522-6.179 (p40-81)	55.2	0.71	21.0	0.11	3.614E-05
**34**	gender_ratio <100.126 (p79) &med_income <20155.135 (p68) &POI_pp_telecom_elec <12.529 (p50)	ave_waiting_period =4.099-5.873 (p22-76)	67.3	0.84	20.9	0.03	0.0001119
**35**	gender_ratio <101.246 (p80) &prop_preprim_edu <0.128 (p79) &med_income <22728.923 (p72)	ave_waiting_period =3.754-5.828 (p16-75)	93.1	0.82	20.9	0.04	0.0002417
**36**	prop_over65 <0.184 (p69) &med_area_home <19.338 (p57) &POI_pp_telecom_elec <18.675 (p65)	ave_waiting_period =4.001-5.761 (p22-74)	64.0	0.85	20.9	0.06	9.959E-05
**37**	med_area_home <19.914 (p61) &POI_pp_mall_mkt <10.111 (p69)	ave_waiting_period =4.037-5.725 (p22-74)	80.9	0.75	20.9	0.07	0.0001365
**38**	prop_preprim_edu <0.125 (p77) &med_income <19096.021 (p58) &POI_pp_mall_mkt <10.359 (p70)	ave_waiting_period =4.148-5.622 (p24-71)	63.7	0.75	20.8	0.05	0.0001529
**39**	gender_ratio <101.386 (p80) &prop_preprim_edu <0.138 (p81) &POI_pp_telecom_elec <15.153 (p57)	ave_waiting_period =3.608-5.942 (p14-78)	95.7	0.88	20.7	0.02	7.052E-05
**40**	prop_over65 <0.207 (p82) &gender_ratio <98.783 (p74) &med_income <19568.062 (p60)	ave_waiting_period =4.015-5.776 (p22-74)	73.3	0.79	20.5	0.06	0.0001903
**41**	gender_ratio <100.607 (p79) &med_area_home <19.338 (p57) &POI_pp_edu <20.523 (p74)	ave_waiting_period =3.799-6.109 (p16-81)	89.5	0.88	20.4	0.03	0.0001358

**42**	prop_preprim_edu <0.137 (p81) &prop_higher_edu <0.360 (p78) &POI_pp_sports <23.041 (p58)	ave_waiting_period =4.083-5.920 (p22-77)	80.9	0.79	20.4	0.03	0.0001199
**43**	gender_ratio <98.227 (p73) &build_area_pp <151.826 (p80) &POI_pp_edu <19.179 (p72)	ave_waiting_period =4.106-5.861 (p23-76)	86.4	0.75	20.3	0.05	0.0002449
**44**	gender_ratio <102.327 (p82) &med_income <19349.019 (p58) &build_area_pp <139.103 (p78)	ave_waiting_period =4.115-5.891 (p23-77)	80.9	0.77	20.3	0.04	0.0001412
**45**	gender_ratio <101.386 (p80) &prop_preprim_edu <0.136 (p81) &POI_pp_mall_mkt <8.764 (p64)	ave_waiting_period =3.608-5.942 (p14-78)	99.8	0.87	20.2	0.02	7.367E-05
**46**	gender_ratio <101.386 (p80) &prop_preprim_edu <0.136 (p81) &POI_pp_transport <35.602 (p56)	ave_waiting_period =3.608-5.942 (p14-78)	93.4	0.88	20.1	0.02	2.543E-05
**47**	prop_preprim_edu <0.124 (p77) &POI_pp_mall_mkt <9.862 (p67)	ave_waiting_period =3.611-5.939 (p14-78)	102.0	0.85	20.0	0.07	3.769E-07
**48**	med_area_home <19.338 (p57) &POI_pp_edu <20.523 (p74)	ave_waiting_period =3.999-5.827 (p20-75)	83.3	0.77	19.8	0.04	0.000222
**49**	prop_preprim_edu <0.134 (p80) &POI_pp_transport <40.374 (p62)	ave_waiting_period =3.611-5.939 (p14-78)	100.4	0.86	19.8	0.04	0.0001354
**50**	prop_preprim_edu <0.128 (p79) &med_income <19994.590 (p60) &build_area_pp <142.936 (p78)	ave_waiting_period =4.177-5.826 (p25-75)	72.9	0.74	19.7	0.04	7.151E-05
**51**	prop_preprim_edu <0.121 (p75) &med_income <19349.019 (p58) &POI_pp_edu <20.242 (p73)	ave_waiting_period =4.097-5.822 (p22-75)	68.9	0.80	19.7	0.04	5.974E-05
**52**	prop_over65 <0.179 (p65) &med_area_home <17.509 (p50)	ave_waiting_period =4.034-5.792 (p22-75)	59.7	0.85	19.7	0.10	2.691E-05
**53**	gender_ratio <102.594 (p82) &prop_higher_edu <0.293 (p69) &build_area_pp <140.696 (p78)	ave_waiting_period =4.117-5.849 (p23-76)	85.1	0.74	19.6	0.04	0.0002139
**54**	prop_preprim_edu <0.126 (p78) &POI_pp_telecom_elec <19.306 (p65)	ave_waiting_period =3.611-5.939 (p14-78)	104.5	0.84	19.5	0.03	0.000211
**55**	gender_ratio <101.386 (p80) &POI_pp_telecom_elec <12.570 (p50)	ave_waiting_period =3.608-5.942 (p14-78)	91.6	0.88	19.5	0.02	2.31E-06
**56**	prop_preprim_edu <0.137 (p81) &med_area_home <18.543 (p52)	ave_waiting_period =4.119-5.872 (p23-76)	80.4	0.75	19.5	0.03	0.0001647
**57**	prop_preprim_edu <0.137 (p81) &POI_pp_sports <23.041 (p58)	ave_waiting_period =4.158-5.571 (p25-70)	74.9	0.66	19.3	0.04	0.0002297
**58**	build_area_pp <77.760 (p54) &POI_pp_telecom_elec <12.658 (p50)	ave_waiting_period =4.463-6.255 (p36-82)	60.3	0.72	19.3	0.09	0.0002241
**59**	prop_preprim_edu <0.137 (p81) &med_area_home <21.280 (p70) &POI_pp_edu <21.599 (p76)	ave_waiting_period =4.119-5.872 (p23-76)	85.6	0.74	19.3	0.04	0.000199

**60**	POI_pp_sports <11.624 (p40)	ave_waiting_period =4.517-6.163 (p40-81)	56.2	0.67	19.3	0.23	1.956E-09
**61**	gender_ratio <101.136 (p80) &POI_pp_sports <26.199 (p63)	ave_waiting_period =3.587-6.044 (p13-80)	107.0	0.86	19.0	0.03	9.064E-05
**62**	prop_higher_edu <0.354 (p78) &POI_pp_telecom_elec <17.856 (p63)	ave_waiting_period =3.950-5.917 (p20-77)	93.0	0.78	19.0	0.03	3.467E-05
**63**	build_area_pp <81.717 (p55) &POI_pp_sports <16.987 (p50)	ave_waiting_period =4.489-6.252 (p37-82)	57.8	0.70	19.0	0.08	8.432E-05
**64**	gender_ratio <102.331 (p82) &prop_preprim_edu <0.124 (p77) &prop_higher_edu <0.360 (p78)	ave_waiting_period =4.083-5.914 (p22-77)	92.5	0.74	19.0	0.03	0.0002457
**65**	prop_preprim_edu <0.127 (p78) &med_income <19096.021 (p58)	ave_waiting_period =4.148-5.622 (p24-71)	69.4	0.70	18.9	0.09	0.0002132
**66**	gender_ratio <101.407 (p80) &build_area_pp <151.678 (p80) &POI_pp_transport <50.379 (p70)	ave_waiting_period =3.639-5.816 (p14-75)	101.2	0.81	18.8	0.02	0.0001082
**67**	gender_ratio <99.654 (p77) &med_income <18510.449 (p57)	ave_waiting_period =3.892-5.810 (p19-75)	81.8	0.78	18.8	0.06	8.888E-05
**68**	prop_preprim_edu <0.140 (p82) &build_area_pp <141.705 (p78) &POI_pp_edu <18.548 (p71)	ave_waiting_period =3.611-5.939 (p14-78)	100.6	0.84	18.8	0.03	9.595E-05
**69**	prop_preprim_edu <0.126 (p78) &POI_pp_edu <17.144 (p68)	ave_waiting_period =3.611-5.939 (p14-78)	104.2	0.84	18.6	0.05	1.849E-05
**70**	POI_pp_telecom_elec <15.395 (p57)	ave_waiting_period =3.650-5.765 (p14-74)	97.7	0.80	18.4	0.15	3.654E-11
**71**	gender_ratio <102.296 (p82) &POI_pp_transport <31.274 (p52)	ave_waiting_period =3.642-6.079 (p14-81)	94.9	0.87	18.4	0.02	4.147E-05
**72**	prop_preprim_edu <0.124 (p77) &prop_higher_edu <0.302 (p70)	ave_waiting_period =4.081-5.450 (p22-68)	74.8	0.65	18.3	0.09	1.07E-05
**73**	med_area_home <17.644 (p50)	ave_waiting_period =4.102-5.807 (p23-75)	77.6	0.72	18.2	0.17	1.443E-11
**74**	prop_higher_edu <0.356 (p78) &med_area_home <19.914 (p61)	ave_waiting_period =3.825-5.877 (p16-77)	96.3	0.78	18.1	0.02	0.0001507
**75**	den_population >19400.293 (p43) &POI_pp_mall_mkt <5.588 (p51)	ave_waiting_period =4.526-6.160 (p40-81)	47.4	0.71	18.1	0.12	0.0001115
**76**	med_income <19588.403 (p60) &build_area_pp <126.455 (p75)	ave_waiting_period =4.069-5.628 (p22-71)	75.4	0.69	17.5	0.07	0.0001283
**77**	gender_ratio <101.386 (p80) &POI_pp_mall_mkt <8.976 (p65)	ave_waiting_period =3.608-5.942 (p14-78)	106.3	0.83	17.5	0.03	6.824E-05

**78**	med_income <19509.546 (p60) &prop_transport >0.068 (p29)	ave_waiting_period =4.071-5.970 (p22-78)	74.2	0.78	17.5	0.10	3.684E-05
**79**	prop_higher_edu <0.293 (p69) &den_population >18412.543 (p43)	ave_waiting_period =4.117-5.849 (p23-76)	63.8	0.78	17.5	0.08	0.0001523
**80**	med_income <19568.062 (p60) &POI_pp_transport <29.494 (p51)	ave_waiting_period =4.119-5.872 (p23-76)	64.7	0.78	17.5	0.06	0.0001698
**81**	build_area_pp <59.911 (p49) &prop_gov_insti_faci >0.027 (p31)	ave_waiting_period =4.174-5.857 (p25-76)	58.5	0.77	17.4	0.08	0.000101
**82**	prop_over65 <0.207 (p82) &med_income <19568.062 (p60) &prop_open_recreation >0.027 (p41)	ave_waiting_period =4.096-5.905 (p22-77)	57.7	0.83	17.2	0.05	0.0001196
**83**	POI_pp_transport <39.683 (p60)	ave_waiting_period =3.633-5.790 (p14-75)	102.4	0.79	17.1	0.13	1.298E-09
**84**	prop_gov_insti_faci >0.028 (p31) &POI_pp_mall_mkt <8.762 (p64)	ave_waiting_period =4.177-5.826 (p25-75)	67.8	0.72	17.1	0.09	3.756E-05
**85**	gender_ratio <103.079 (p83) &POI_pp_sports <30.800 (p67) &POI_pp_transport <31.274 (p52)	ave_waiting_period =3.399-6.268 (p9-82)	100.4	0.93	16.9	0.00	3.149E-05
**86**	gender_ratio <96.204 (p67) &POI_pp_edu <19.100 (p72)	ave_waiting_period =4.089-5.878 (p22-77)	84.8	0.72	16.8	0.07	0.0001529
**87**	prop_transport >0.079 (p30) &POI_pp_mall_mkt <10.478 (p71)	ave_waiting_period =4.039-5.936 (p22-78)	76.7	0.76	16.7	0.09	4.933E-05
**88**	med_income <18877.334 (p57) &prop_open_recreation >0.028 (p43)	ave_waiting_period =4.039-6.004 (p22-80)	63.0	0.82	16.6	0.12	8.371E-06
**89**	prop_higher_edu <0.302 (p70) &prop_transport >0.086 (p32)	ave_waiting_period =4.078-5.915 (p22-77)	70.9	0.77	16.5	0.10	6.928E-06
**90**	gender_ratio <101.724 (p81) &den_population >25055.300 (p49)	ave_waiting_period =4.117-5.849 (p23-76)	65.9	0.75	16.3	0.03	0.0002242
**91**	build_area_pp <77.130 (p54)	ave_waiting_period =4.526-6.160 (p40-81)	67.0	0.58	16.3	0.14	5.406E-07
**92**	prop_preprim_edu <0.143 (p85) &POI_pp_edu <14.015 (p63) &POI_pp_transport <15.096 (p35)	ave_waiting_period =3.631-6.100 (p14-81)	66.1	0.93	15.9	0.00	8.233E-05
**93**	POI_pp_edu <12.794 (p58) &POI_pp_transport <15.096 (p35)	ave_waiting_period =3.631-6.100 (p14-81)	67.5	0.92	15.7	0.01	8.436E-06
**94**	gender_ratio <101.407 (p80) &build_area_pp <151.678 (p80)	ave_waiting_period =4.008-5.745 (p22-74)	97.4	0.66	15.4	0.04	3.101E-05
**95**	med_income <20215.433 (p68) &prop_gov_insti_faci >0.027 (p31)	ave_waiting_period =4.174-5.857 (p25-76)	64.7	0.70	15.0	0.08	0.0001548

**96**	POI_pp_mall_mkt <2.416 (p28)	ave_waiting_period =4.401-5.822 (p34-75)	41.2	0.72	15.0	0.26	2.638E-07
**97**	gender_ratio <101.661 (p81) &prop_higher_edu <0.332 (p73)	ave_waiting_period =3.825-5.877 (p16-77)	104.7	0.74	14.9	0.05	0.0002342
**98**	prop_publicResid >0.048 (p64) &POI_pp_mall_mkt <9.418 (p65)	ave_waiting_period =4.033-6.072 (p22-81)	48.9	0.87	14.7	0.04	3.573E-05
**99**	prop_higher_edu <0.219 (p51) &prop_open_recreation >0.027 (p41)	ave_waiting_period =4.102-5.890 (p23-77)	51.0	0.81	14.4	0.15	8.691E-06
**100**	POI_pp_edu <14.015 (p63)	ave_waiting_period =3.640-5.788 (p14-75)	99.9	0.77	14.1	0.11	3.847E-09
**101**	prop_publicResid >0.053 (p65)	ave_waiting_period =4.097-5.657 (p22-72)	45.0	0.76	13.7	0.23	1.57E-06
**102**	den_population >39592.585 (p62)	ave_waiting_period =4.075-5.980 (p22-78)	52.3	0.79	13.5	0.20	1.659E-05
**103**	med_income <19979.413 (p60)	ave_waiting_period =3.689-5.751 (p15-74)	96.8	0.72	11.5	0.09	9.251E-06
**104**	prop_gov_insti_faci >0.028 (p31)	ave_waiting_period =4.177-5.826 (p25-75)	80.7	0.62	11.0	0.09	1.855E-05
**105**	prop_preprim_edu <0.125 (p77)	ave_waiting_period =4.148-5.622 (p24-71)	93.0	0.56	9.5	0.06	0.0001761
**106**	gender_ratio <102.339 (p82)	ave_waiting_period =3.686-5.720 (p15-74)	119.1	0.68	9.3	0.05	2.591E-06
**107**	prop_higher_edu <0.351 (p78)	ave_waiting_period =3.724-5.750 (p15-74)	112.0	0.68	8.4	0.05	2.264E-05
**108**	build_area_pp >77.760 (p54) &POI_pp_telecom_elec <12.658 (p50)	ave_waiting_period =3.736-4.463 (p15-36)	12.5	0.49	6.8	0.24	1.595E-05
**109**	POI_pp_sports =14.823-29.352 (p46-65)	ave_waiting_period =4.058-4.517 (p22-40)	13.6	0.34	6.1	0.15	4.769E-05
**110**	build_area_pp >81.717 (p55) &POI_pp_sports <16.987 (p50)	ave_waiting_period =3.711-4.489 (p15-37)	9.9	0.48	4.9	0.20	0.0001676
**111**	prop_gov_insti_faci <0.028 (p31)	ave_waiting_period =3.992-4.177 (p20-25)	7.0	0.10	3.4	0.05	0.000185
**112**	den_population =6601.148-27429.878 (p30-53) &prop_transport <0.064 (p28)	ave_waiting_period =4.658-5.633 (p45-71)	4.5	0.57	2.3	0.29	0.0001078
**113**	build_area_pp >77.130 (p54)	ave_waiting_period <4.113 (p23)	32.6	0.39	11.4	0.14	7.052E-06
**114**	prop_publicResid <0.006 (p50)	ave_waiting_period <4.097 (p22)	37.7	0.35	10.7	0.10	8.287E-05

**115**	den_population <39592.585 (p62)	ave_waiting_period <4.075 (p22)	42.7	0.32	10.6	0.08	4.796E-05
**116**	prop_over65 >0.163 (p51) &den_population <28746.747 (p55)	ave_waiting_period <4.183 (p26)	21.5	0.53	10.4	0.17	0.0002337
**117**	med_area_home >17.644 (p50)	ave_waiting_period <4.102 (p23)	32.2	0.35	9.2	0.10	0.0001486
**118**	POI_pp_telecom_elec >15.395 (p57)	ave_waiting_period <3.650 (p14)	20.2	0.27	8.7	0.11	9.833E-06
**119**	POI_pp_transport >39.683 (p60)	ave_waiting_period <3.633 (p14)	17.2	0.25	7.2	0.11	0.0002353
**120**	POI_pp_edu >14.015 (p63)	ave_waiting_period <3.640 (p14)	16.3	0.24	6.4	0.10	0.0002096
